# The global kinetic–thermodynamic relationship derived from first principles

**DOI:** 10.1039/d5sc04829j

**Published:** 2025-08-21

**Authors:** Eduardo Garcia-Padilla, Guanqi Qiu

**Affiliations:** a Max-Planck-Institut für Kohlenforschung Kaiser-Wilhelm-Platz 1 45470 Mülheim an der Ruhr Germany garciapadilla@kofo.mpg.de gqiu@kofo.mpg.de

## Abstract

What governs the relationship between the reaction rate and thermodynamic driving force? Despite decades of rate theory, no general physically grounded equation exists to relate rate and driving force across all regimes. Classical models, such as the Marcus equation and Leffler equations, either rely on under-realistic assumptions or only capture the local behaviour, failing outside narrow regimes. We derive a general, non-linear equation from microscopic reversibility, arriving at three physically meaningful parameters: a minimum preorganisational barrier (*E*_min_), a reaction symmetry offset (*E*_eq_), and a kinetic curvature factor (*θ*). This model captures global behaviour, recovers known limits, and explains why classical models like Leffler equation exhibit the rate–driving force responsiveness (the Brønsted slope) as they do, by revealing their physical origin, not by fitting them. The model enables a causal reinterpretation of experimentally observed curved rate–driving force plots, such as in hydrogen atom transfer to Fe(iv)=O. Importantly, this model does not require replacing existing models, it explains their physical foundations, enabling chemists to continue using them while understanding when and why they apply, and where they break down. Beyond case studies including hydride shifts, rearrangements, and cyclisations, the framework's strength lies in its deductive foundation enabling the physically grounded design of reactions with desired kinetics across diverse chemical systems. By revealing the global structure of the rate–driving force relationship, this framework enables chemists to recognise, predict, and design reactivity that would otherwise appear anomalous or inaccessible, better clarifying the unknowns. Examples include highly exergonic regimes near *E*_min_, where further increases in exergonicity offer little rate improvement, and control shifts to structural factors; even when the rate–driving force plot appears linear, the model uncovers hidden curvature and deeper physical meaning.

## Abstract 2

What governs the relationship between the reaction rate and thermodynamic driving force? We present a physically grounded equation that causally relates energy barriers with reaction energies.

## Graphical abstract

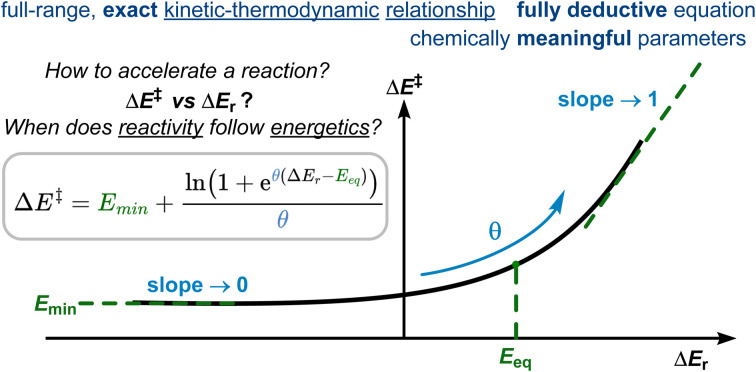

## 1 Introduction

What determines how reaction rates respond to a thermodynamic driving force? Why do some reactions speed up dramatically with slight changes in driving force, while some others appear much less responsive? The interplay between kinetics and thermodynamic driving forces has been a cornerstone of physical organic chemistry. The most elegant aspect of Marcus theory lies in dissecting the reaction barrier into thermodynamic-independent and thermodynamic-dependent components.^1^ This dissection immediately raises a critical question: what are the “thermodynamic-independent” parameters defining the inherent nature of a family of reactions?^2^ This question forms the foundation of different attempts to describing the interplay, leading to different models. However, many previous models rely either on excessively strong assumptions,^1–3^ or, in attempts to be more comprehensive, on extensive parametrisation of reaction-specific concepts,^4^ which greatly restricts the scope and the ability to derive meaningful physical insights from these frameworks due to their more statistical nature. These include models that incorporate bond breaking and bond formation to estimate the energy of the transition state, which often require treatments very specific to one reaction class.^4^ Existing models interpret these cases as breakdowns or edge effects, but perhaps they are evidence of a deeper, universal relationship. Addressing these limitations requires a model that captures the global relationship between activation and reaction energies across the entire thermodynamic range that relies on minimal assumptions. Existing models are often thermodynamically narrow, too empirical, or too dependent on system-specific parametrisation. What has been missing is a unified, physically interpretable framework that recovers known behaviour as limits, and explains why existing models succeed or fail.^2^

**Fig. 1:**
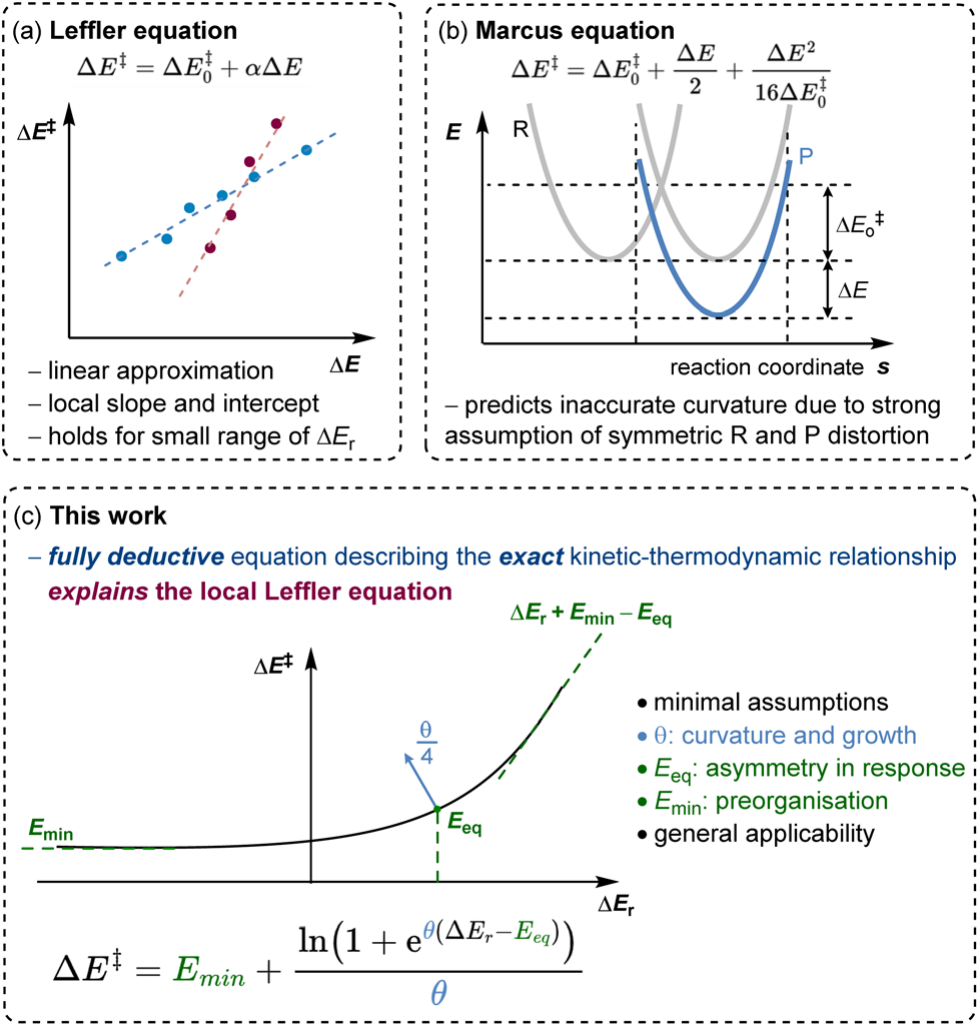
Models in describing the interplay between thermodynamic-independent and thermodynamic-dependent components of chemical reactivity: (a) Leffler equation as a local linear fitting with an empirically derived slope *α* treated as a constant, (b) Marcus model predicting a curved relationship, but with inaccurate curvature and limiting behaviour, (c) our derived exact model yielding three key parameters with physical meaning: a minimum preorganisational barrier (*E*_min_), a reaction symmetry offset (*E*_eq_), and an energetic coupling factor (*θ*). Our causal model explains why the Leffler equation yields the Brønsted slope value as it does and clarifies the physical basis of the observed curvature.

For example, the most experimentally relevant is the Leffler equation (Fig. 1a), which expresses the energy of activation Δ*E*^‡^ by a combination of the reaction energy Δ*E* and the intrinsic barrier Δ*E*^‡^_0_, which corresponds to Δ*E*^‡^ for Δ*E* = 0, in the linear approximation, Δ*E*^‡^ = Δ*E*^‡^_0_ + *α*Δ*E*.^2^ The Brønsted slope *α* indicates the sensitivity of the reaction rates to changes in driving force. Δ*E*^‡^_0_ and *α* are intrinsic properties of each family of reactions, presumed to be independent of the thermodynamic driving force. For constant Δ*E*^‡^_0_ and *α*, the Leffler equation is reduced to the Bell–Evans–Polanyi (BEP) principle, describing the kinetic–thermodynamic relationship within the same family of reactions.^5^ The value of *α* is often used to probe how early or late in a reaction the transition state develops, with values lower than 0.5 corresponding to an earlier transition state. Although the linear kinetic–thermodynamic relationship is commonly observed experimentally, it is the approximation over a limited range of thermodynamic driving force. As the driving force becomes highly exergonic or endergonic, the linearity breaks down.^6^ In the extreme limits, the behaviour of *α* reveals distinct kinetic regimes. For highly exergonic reactions, *α* approaches 0; conversely, for very endergonic reactions, *α* approaches 1.^7^ BEP plots often show deviations from linearity.^8^ These outliers prompt a critical question: do they indicate curvature due to driving forces exceeding the linear range, or do they reflect reactions from distinct families with different intrinsic properties? Analysing energy relationships in their exact non-linear form is essential to addressing this, as true chemical information can only be found when moving beyond parameters for a local interpolation. A universal, deductive relationship between rate and reaction energy remains conspicuously absent. This absence has not gone unnoticed, but it is often obscured by models that appear to work locally while failing to generalise, with all current models either empirical, local, or reliant on idealised reaction assumptions. Marcus derived a curved equation from the analysis of crossing the reactant state parabola and the product state parabola (Fig. 1b), and a follow-up work offers a thermodynamically rigorous description.^9^ This model predicts a Brønsted slope *α* near 0.5 for reactions close to zero driving force. A serious assumption in the Marcus formation of the curve is that the parabolas of the reactant and the product states have identical shape. These shapes are influenced by factors such as bonding, conjugation, steric properties, solvation, and non-covalent interactions.^10^ This oversimplification obscures the true origins of deviations from linear BEP relationships. Koeppl and Kresge demonstrated that allowing bond strengths and reaction distances to vary realistically produces a sigmoid Brønsted slope, better reflecting real systems,^11^ which would deviate from Marcus predictions.^12^ Similarly, Richard and Jencks found significant deviations in Brønsted slopes for general base catalysis, attributing them to differential charge development in the transition state and reactant-product asymmetry.^13^ These studies introduce reaction-specific corrections. Each of these formulations and models rely on distinct assumptions and, while having advanced our understanding of kinetic–thermodynamic relationships, still often address aspects in isolation.

Herein, we present a model that originates from a distinct foundation and goes significantly beyond the scope of existing frameworks. In this study (Fig. 1c), we derive a non-linear equation grounded in the principle of microscopic reversibility, with minimal assumptions, which captures (or “arrives at”, given its deductive nature) all these essential physical features: reaction asymmetry, reactant preorganisation, product reorganisation, and the sensitivity of kinetic–thermodynamic response at each thermodynamic driving force. Together, these portray the full reactivity landscape. In comparison to *G*^‡^_0_ and *α* that represent the local thermodynamic-independent properties of a family of reactions, we introduce a new set of thermodynamic-independent parameters overarching the global thermodynamic range. Each parameter has a distinct physical interpretation, grounded in common chemical meaning (in contrast to statistical in nature). We demonstrate the applicability of this equation through diverse chemical reactions, illustrating its capacity to describe non-linearity, identify valid linear approximations, and elucidate the role of each thermodynamic-independent parameter. Experimental observations show that some families of reactions exhibit high sensitivity to rate–driving force responsiveness, while others display relatively insensitive correlations.^14^ Some sets of reactions exhibit crossing behaviour: one is more reactive in the endergonic region, while the other is more favoured in the exergonic region of thermodynamic driving forces. These distinctions raise practical questions: What should we do to accelerate a given reaction? To what extent will making a reaction more thermodynamically downhill improve its rate? Our derived equation also provides a lens through which to examine these questions. This framework reveals the physical origins of observed Brønsted slopes, showing why they have the values they do across different regimes, and recovers them as limiting behaviour under a broader kinetic–thermodynamic relationship.

## 2 Results and discussion

Finding a global model for the kinetic–thermodynamic relationship generalisable for any chemical reaction must arise from the most fundamental characteristics. Any approach from linearity or parametrisations specific to one reaction class, even when not *ad hoc*, hamper both the physicality and generality of the resulting equations. For these reasons, we investigated what mathematical features should be present in the global energy responses, followed by a deductive approach in agreement with the former and stemming from an interpretation of microscopic reversibility.

### 2.1. Derivation of the constraints and properties of the non-linear relationship

**Fig. 2:**
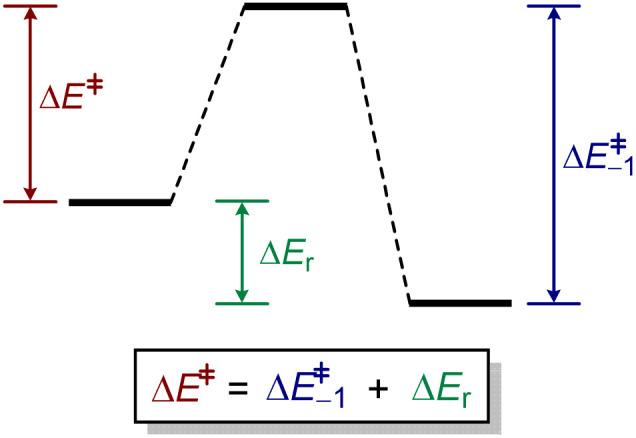
The activation energies and reaction energy of a reverse reaction are fundamentally connected to the forward reaction.

In order to reach a model dependent on physically meaningful parameters, the question must be approached in the most general way possible by considering the properties common to any kind of chemical reaction. Through this, we should deductively arrive at a set of constraints from characteristics general to all energy responses, rather than assuming that a particular behaviour should apply to the non-linear relationship. For a given family of reactions, it would be possible to determine Δ*E*^‡^ as a function of Δ*E*_r_, where Δ*E*_r_ is the reaction energy and Δ*E*^‡^ is the activation energy. In any chemical reaction, the reaction energy can be expressed as the difference between the forward and backward energy barriers (Fig. 2).

This non-linear function would have to satisfy a number of physically meaningful requirements that define its properties, particularly those concerning the symmetry of the function and its derivatives. As there is a fundamental relationship between the energies of a given reaction and its reverse, the function linking the reaction energy to the forward energy barrier, Δ*E*^‡^(Δ*E*_r_) and the one linking the (negative) reaction energy to the reverse energy barrier, Δ*E*^‡^_−1_(−Δ*E*_r_), have to be connected somehow. This feature of all reactions constrains the nature of the function. By considering how the energy barriers and reaction energies invert between a general forward and reverse reaction, the following constraints on the nature of the non-linear relationship are obtained:

1 Δ*E*^‡^_−1_(−Δ*E*_r_) = Δ*E*^‡^(Δ*E*_r_) − Δ*E*_r_

2 −[Δ*E*^‡^_−1_]′(−Δ*E*_r_) = −1 + [Δ*E*^‡^]′(Δ*E*_r_)

3 [Δ*E*^‡^_−1_]′′(−Δ*E*_r_) = [Δ*E*^‡^]′′(Δ*E*_r_)

**Fig. 3:**
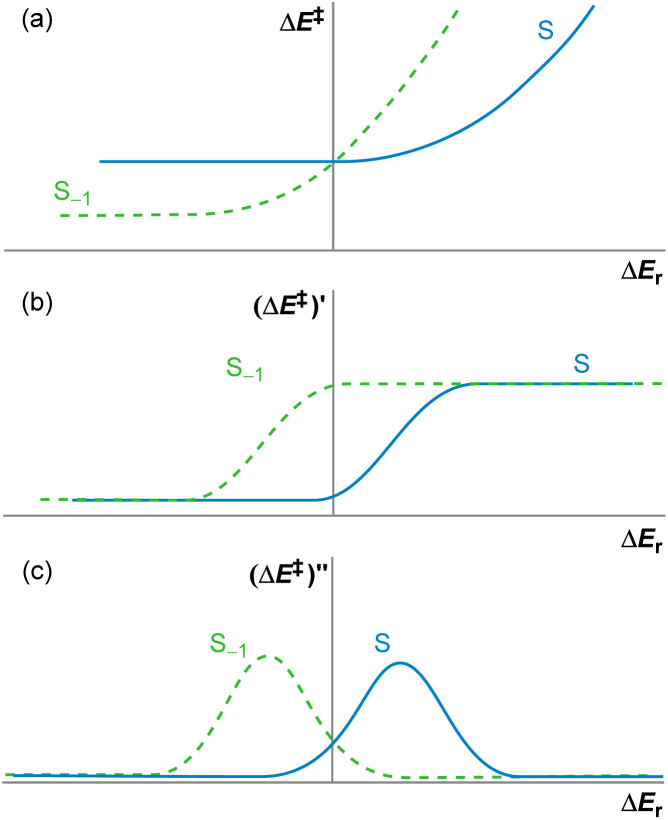
Graphical representation of (a) the non-linear kinetic–thermodynamic response, eqn (1); (b) its gradient, eqn (2); and (c) its curvature, eqn (3); for a set of forward reactions *S* and the set of reverse reactions *S*_−1_.

Eqn (1) relates the activation energy of a reverse process to the activation energy of the forward process, which are always different by the reaction energy, Δ*E*_r_. Therefore, given a constant horizontal displacement in Δ*E*^‡^_−1_, a vertical displacement of the same magnitude would appear in Δ*E*^‡^. By differentiating eqn (1) once and twice, we obtain eqn (2) and (3), respectively. The first derivative shows that the gradients of the forward and reverse reactions are complementary, adding up to one. The second derivative, the curvature, of the backward reaction is the mirror image about the *y* axis of that of the forward reaction (as −Δ*E*_r,−1_ = Δ*E*_r_) (Fig. 3).

The additional conceptually derivable constraints on a function that models the kinetic–thermodynamic relationship involve the limits at extreme driving forces. Due to the TS interpolating between the minima, the regimes at extreme driving forces must be asymptotic to the energies of the minimum closest in energy. As the reaction energy approaches negative infinity, the factors stabilising the product cannot be present in the TS and so the energy of the TS must only depend on that of the reactant. In this limit, the gradient 
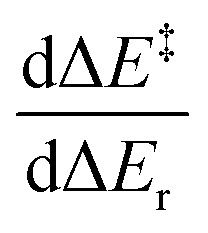
 must approach zero, with the barrier approaching 
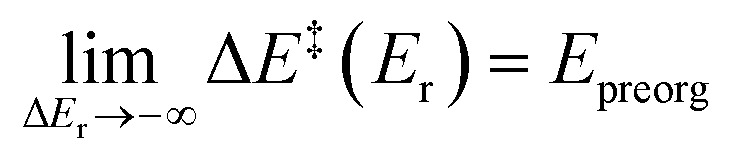
, where *E*_preorg_ represents the Δ*E*_r_-independent minimum barrier to be overcome regardless of the driving force. The reverse is also true for extremely endergonic reactions. In the limit, the barrier must be equal to the reaction energy plus a reactant-independent offset and, hence, independent of Δ*E*_r_. This is represented as Δ*E*_r_ + *E*_reorg_, where *E*_reorg_ is the Δ*E*_r_-independent reorganisation energy of the product. As these limits are linear, the gradient of the non-linear relationship must transition between its asymptotic limits at 0 and 1. For an idealised reaction following Hammond's postulate, and so in which the geometry of the TS is fully and exclusively defined by its connected minima, only the factors influencing the TS are present to various degrees in the connected minima, and so the response of Δ*E*^‡^ must vary with the energetic proximity to the minima. As the position of the energy barrier stems from an interpolation of stabilising factors that change monotonically in elementary processes, the response of Δ*E*^‡^ thereto must be of the same sign as the response of Δ*E*_r_. In cases where this does not hold, the TS cannot be seen as an interpolation of factors. This situation only occurs if the potential energy surface shows extensive asynchronicity, caldera-like regions or bifurcation-like behaviour.^15^ These effects of asynchronicity can lead to incorporation of extraneous stabilisation factors, as one of the minima would be less geometrically related to the TS, and presumably significant changes in reaction energy unrelated to the factors affecting the barrier. Therefore, for any single synchronous elementary process, 
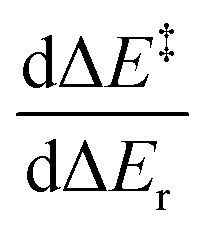
 must always be positive and monotonically increasing, with the second derivative positive for all values of Δ*E*_r_.

We now consider an identity reaction *r*^i^, with its reactants and products being identical. There are many examples of such identity reactions, including degenerate Cope rearrangements, degenerate deprotonations with a conjugate base, and type I and type II dyotropic reactions. This reaction belongs to a family of analogous reactions, A, such that the corresponding family of backward reactions A_−1_ is equivalent. Such reactions may be labelled isodromic, as the reverse reactions show the same features as the forward reactions, stemming from how the reactants form products of similar nature.^16^ The energy response of these systems perfectly aligns with the notion of early or late TS, with exergonic reactions showing earlier transition states and thermoneutral reactions having the TS at the middle of the process. Being so closely related to the identity reaction, these isodromic reactions would energetically behave like the identity reaction upon the appearance of a driving force due to changes in reaction energy, the only difference between forward and backward reactions being the reaction energy. As these are indistinguishable, the kinetic–thermodynamic relationship of the forward and backward isodromic reactions, where an identity reaction belongs by definition to both sets, must be modelled by the same function as by definition they show the same energy-related features. The identity reaction is, by definition, thermoneutral, and its reverse is itself, and so Δ*E*^‡^(Δ*E*_r_) = Δ*E*^‡^_−1_(Δ*E*_r_) for any idealised isodromic family containing *r*^*i*^. Following eqn (2), the gradient [Δ*E*^‡^]′(0) = 0.5 and so the function is symmetric about (0, 0.5).^17^

**Fig. 4:**
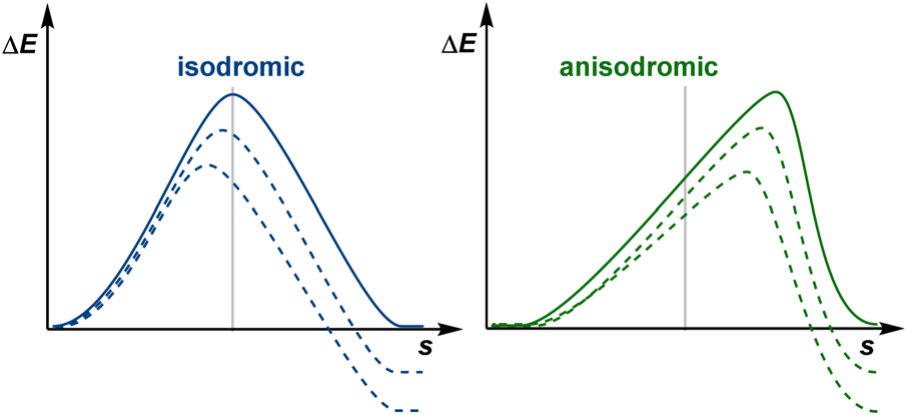
Reaction coordinate for thermoneutral (plain) and non-thermoneutral (dashed) reactions of differing isodromicity. A vertical line shows the perfect symmetry for the thermoneutral isodromic reaction, as it would be an identity reaction.

**Fig. 5:**
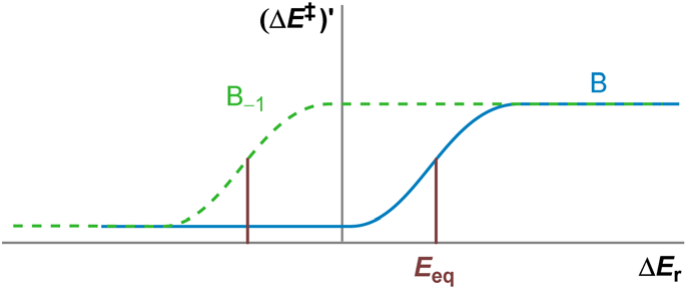
Relationship between the gradient of the kinetic–thermodynamic response of a set of anisodromic reactions B and the set of their reverse counterparts B_−1_. The points where their gradient is 0.5 are separated from the *y* axis by *E*_eq_.

Conversely, for a family of anisodromic reactions, B, such that no reaction of B_−1_ belongs to B, the curves Δ*E*^‡^ and Δ*E*^‡^_−1_ would intersect at Δ*E*_r_ = 0 by definition. However, their gradients at Δ*E*_r_ = 0 could be different from 0.5. This is clearly identifiable as thermoneutral reactions would still be found to be early or late in spite of having no apparent thermodynamic bias, resulting in a mismatch with any symmetric model.^18^ The TS of thermoneutral anisodromic reactions could thus in principle be displaced from the centre of the reaction coordinate and so early or late TSs switch at a reaction energy different from 0 (Fig. 4). Applying eqn (2), which provides the symmetry constraints of the gradient, shows that there is an energy *E*_eq_ for which [Δ*E*^‡^]′(Δ*E*_r_ + *E*_eq_) = [Δ*E*^‡^_−1_]′(Δ*E*_r_ − *E*_eq_) = 0.5 (Fig. 5). Hence, [Δ*E*^‡^]′ and [Δ*E*^‡^_−1_]′ are symmetric about 
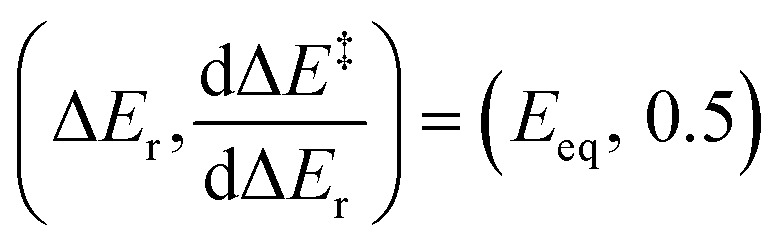
 and (−*E*_eq_, 0.5), respectively. *E*_eq_ would represent the point at which the influence of reactants and products on the energy barrier is equal. This is already unaccounted for in the Marcus quadratic equation, for which the gradient at 0 is invariant since, by construction, the Marcus equation assumes perfectly isodromic behaviour. This can cause large deviations for anisodromic reactions.^17^

Consequently, as both [Δ*E*^‡^]′(Δ*E*_r_ + *E*_eq_) and [Δ*E*^‡^_−1_]′(Δ*E*_r_ − *E*_eq_) are symmetric about (0, 0.5), and their curvature is identical (eqn (3)), they must be equal. We can of course integrate these with respect to the reaction energy to recover the kinetic–thermodynamic relationship. This means that the kinetic–thermodynamic relationship functions themselves, Δ*E*^‡^(Δ*E*_r_ + *E*_eq_) and Δ*E*^‡^_−1_(Δ*E*_r_ − *E*_eq_), can only differ by a single constant (the integration constant) for any Δ*E*_r_. The difference between their limits at negative infinity, *E*_preorg_ − *E*_preorg,−1_, and at positive infinity, (*E*_reorg_ + *E*_eq_) = (*E*_reorg,−1_ − *E*_eq_), must be equal. As the reorganisation of the product from the TS is, by definition, equivalent to the preorganisation of the reverse reaction, *E*_reorg_ = *E*_preorg,−1_, *E*_eq_ is obtained from the limits of the function:

4 *E*_eq_ = *E*_preorg_ − *E*_reorg_

The value of the barrier at *E*_eq_ can only depend on the curvature, [Δ*E*^‡^]′, which itself controls the transition between the limiting linear behaviour of the energy barrier at extreme driving forces. Owing to the symmetry constraints (eqn (3)), the curvature reaches a maximum at *E*_eq_, while the gradient of the function was shown to be limited to the range between 0 and 1. For a self-similar curvature profile, invariant under rescaling as would be expected for a single-mechanism interpolation, the value of Δ*E*^‡^(*E*_eq_) is therefore inversely proportional to the curvature at that point.^19^ The curvature is responsible for describing the coupling of the two minima to the TS and is the defining feature of the non-linear equation, with the barrier at *E*_eq_ being a direct consequence thereof.

### 2.2. Derivation of equation

We return to ideal isodromic processes, where the stabilising factors affecting the reactants and products would be expected to impact the energy of the transition state proportionately to their influence on the geometry of the transition state. From the previous explored constraints, this influence must be dependent on the energy of the minima, consequently coupling the energy of the TS to those of the intermediates. Ultimately, an exponential dependence on energy can be expected, as it is found in analogous questions such as transition probabilities, rate constants and other aspects in non-equilibrium transition-state theory.^20^ This would be derived from the energy difference, not unlike a Boltzmann distribution, albeit with a scaling parameter different to inverse temperature. For an isodromic reaction set, in which the reactants and products are of the same nature, any changes in energy on either of the minima would result in an identical response on the transition state (TS). Drawing an analogy to microscopic reversibility, as the forward and backward factor transmission influences constitute the total origin of all factors acting on the TS from either minimum, they must add to 1. This can be expressed as a normalised sum of exponentials, where *θ* is the reaction-specific scaling factor related to the responsiveness of the barrier (eqn (5)):5
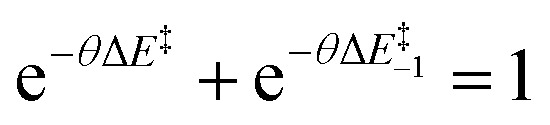


The value of *θ* captures the partition of influences and, hence, must be a positive real number. Larger values would correspond to a stronger coupling, where the TS is highly influenced by the minima, whereas values closer to zero would mean that the transition state responds less rapidly to thermodynamic changes. This can be rationalised as the stabilisation of the TS arising from the competing factors modifying the reactants and the products. The ease of transmission of stabilising factors to the TS would thus be modulated by *θ*: higher values would lead to a TS more responsive to the factors that stabilise the minima. Conceptually, this would represent the information about the reaction hitherto usually ascribed to the Brønsted slope. Re-expressing Δ*E*^‡^_−1_ in eqn (5) as (Δ*E*^‡^ − Δ*E*_r_) and rearranging^20^ shows the energetic dependence of the barrier with the minima it connects as an explicit function, for the idealised set of reactions:6
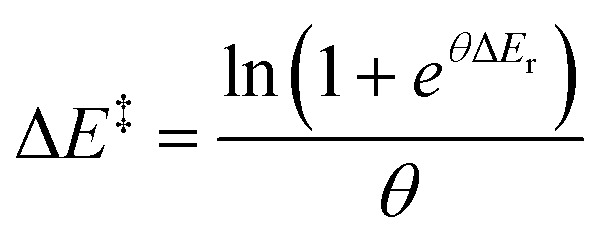


From eqn (5), for the thermoneutral isodromic identity reaction (with *E*_eq_ = 0), the forward and backward barriers are the same, 
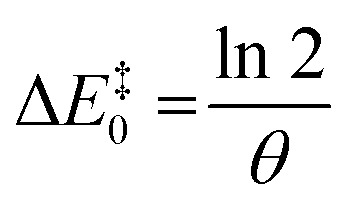
. This inverse proportionality to *θ* matches the constraint we found regarding the connection between curvature and the barrier at *E*_eq_, with the curvature at that point being 
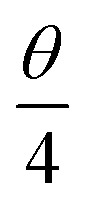
. As explored earlier, the asymptotic minimum energy of preorganisation could be non-zero. In addition, for a set of anisodromic reactions B, the expression would be displaced by *E*_eq_, and so by the point with a gradient of 0.5 for reactions with unequal responses, giving rise to the final expression for the function:7
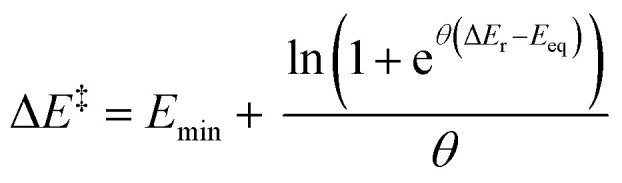


**Fig. 6:**
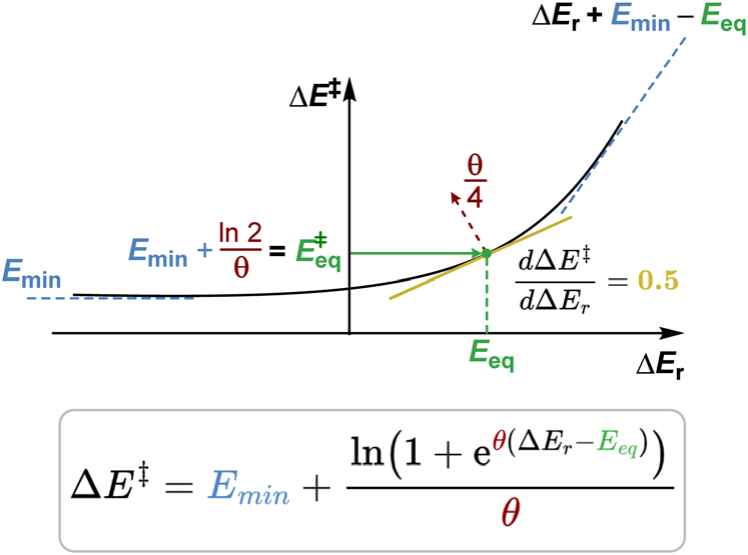
Graphical representation of the effects of each parameter on the behaviour of the equation. Note that the gradient at *E*_eq_ is always equal to 0.5.

The limits of this function at negative and positive infinity are *E*_min_ and Δ*E*_r_ + (*E*_min_ − *E*_eq_) respectively, which match the derived constraints for the asymptotic preorganisation and reorganisation energies, as well as the definition of *E*_eq_. Note that the constraints were derived from the general behaviour of forward and backward reactions (*vide supra*), whereas the equation was derived independently from the balance of influences in eqn (7). Hence, both the correct asymptotic behaviour and the symmetry of the gradient expected from the constraints are reached separately and serve as additional validation of the energy relationship. The effects of each parameter energy response are shown in Fig. 6.

While the kinetic–thermodynamic plots of some reactions exhibit observed slopes outside of the range 0–1 (such as the nitroalkane anomaly or the Marcus inverted region),^21^ these observed local gradients do not contradict our model but instead arise from parallel system-dependent effects. In most known examples, the disruption of the monotonicity of the kinetic–thermodynamic response occurs because of the weakening of Hammond's postulate, with the outcome of such a treatment akin to a Hammett-type correlation instead of a causal relationship, which sometimes results in the inverted region. Experimental evidence supports this, as in most observed inverted regions, the inverted Brønsted slopes vary significantly in magnitude,^22^ deviating from the expected same-magnitude-inverse-sign predicted by the quadratic equation in Fig. 1b. In fact, and in any case as explored in Section I, a non-monotonic energy response cannot be compatible with Hammond's postulate. In ET and many other reactions, asynchronicity is common,^23^ which limits the validity of Hammond's postulate for the particular reaction, while in intersystem crossings there is no required smooth interpolation of the potential energy surface. With asynchronicity, as more than one process occurs, such trends are context-dependent and have also been related to the nitroalkane anomaly,^24^ reinforcing the Hammett-like behaviour observed in these cases. Only in this scenario, perhaps closer in nature to a collection of independent steps than to a concerted process, would the TS correlate weakly enough for monotonicity not to be preserved: this can lead to cases where local observed Brønsted slopes were negative or higher than 1, a few examples of which have been presented as outliers in the literature.^25^ In this light, our equation must always hold unless the reaction is highly asynchronous or the reaction class is ill-defined due to external factors. It offers a more fundamental framework for distinguishing when additional factors shape reaction behaviour and prompts the need to capture the evolving phases of a reaction, which in turn also enables conceptually more accurate and predictive kinetic analysis. Anomalous kinetic behaviour can only then be systematically interpreted, instead of heuristically fitted.

### 2.3. Demonstrations on chemical reactions

**Fig. 7:**
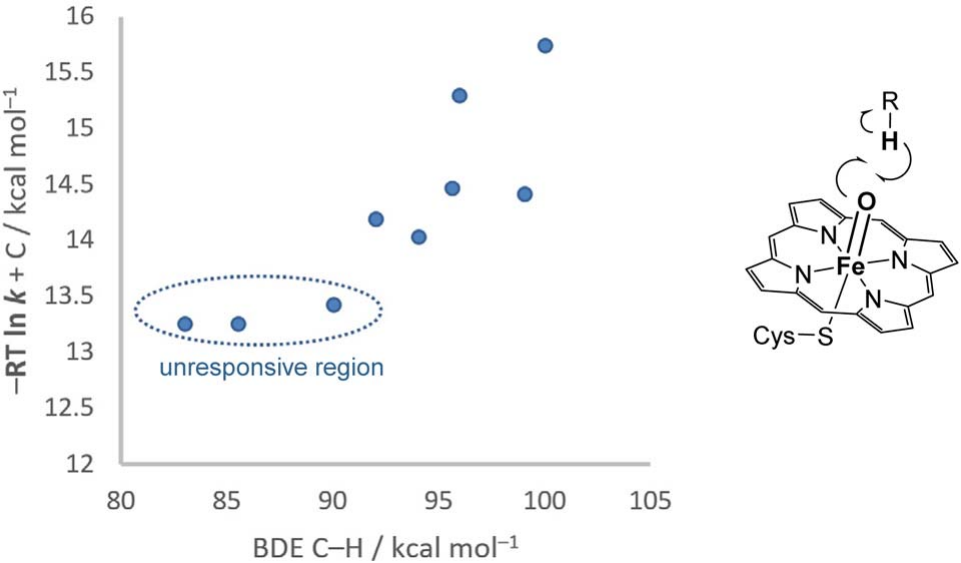
Experimental observation of a non-linear kinetic–thermodynamic response in hydrogen atom transfer to an iron(iv) oxo complex.^23^ The horizontal axis is the C–H bond dissociation energy (BDE) of substrates, the difference of which gives the variation in reaction enthalpy. The vertical axis is the pseudo-barrier obtained by the rate constant, where *C* = *RT* ln(*k*_B_*T*/h), with *R* as the gas constant, *k*_B_ as the Boltzmann constant, *h* as Planck's constant, and *T* = 277 K in this example.

A particularly clear example of experimentally found non-linearity in kinetic–thermodynamic relationships is that by Groves and coworkers involving the hydrogen atom transfer from a number of organic molecules to a key iron(iv) oxo complex in a peroxygenase (Fig. 7).^26^ While treating all C–H bonds amenable to activation in each substrate as equivalent and observing the average effect, as well as including substrates of a very divergent nature, with the consequent spread in the values, the results displayed visible non-linearity. The pseudo-barriers derived from the rate constants shifted from an essentially asymptotic unresponsive region to a more responsive section. Our derived equation provides a key causal link that accounts for these prior experimental findings. Practically, this implies that further increasing the thermodynamic driving force would no longer accelerate the reaction in this unresponsive region, reflecting an insight that the Leffler's equation cannot tell.

**Scheme 1:**
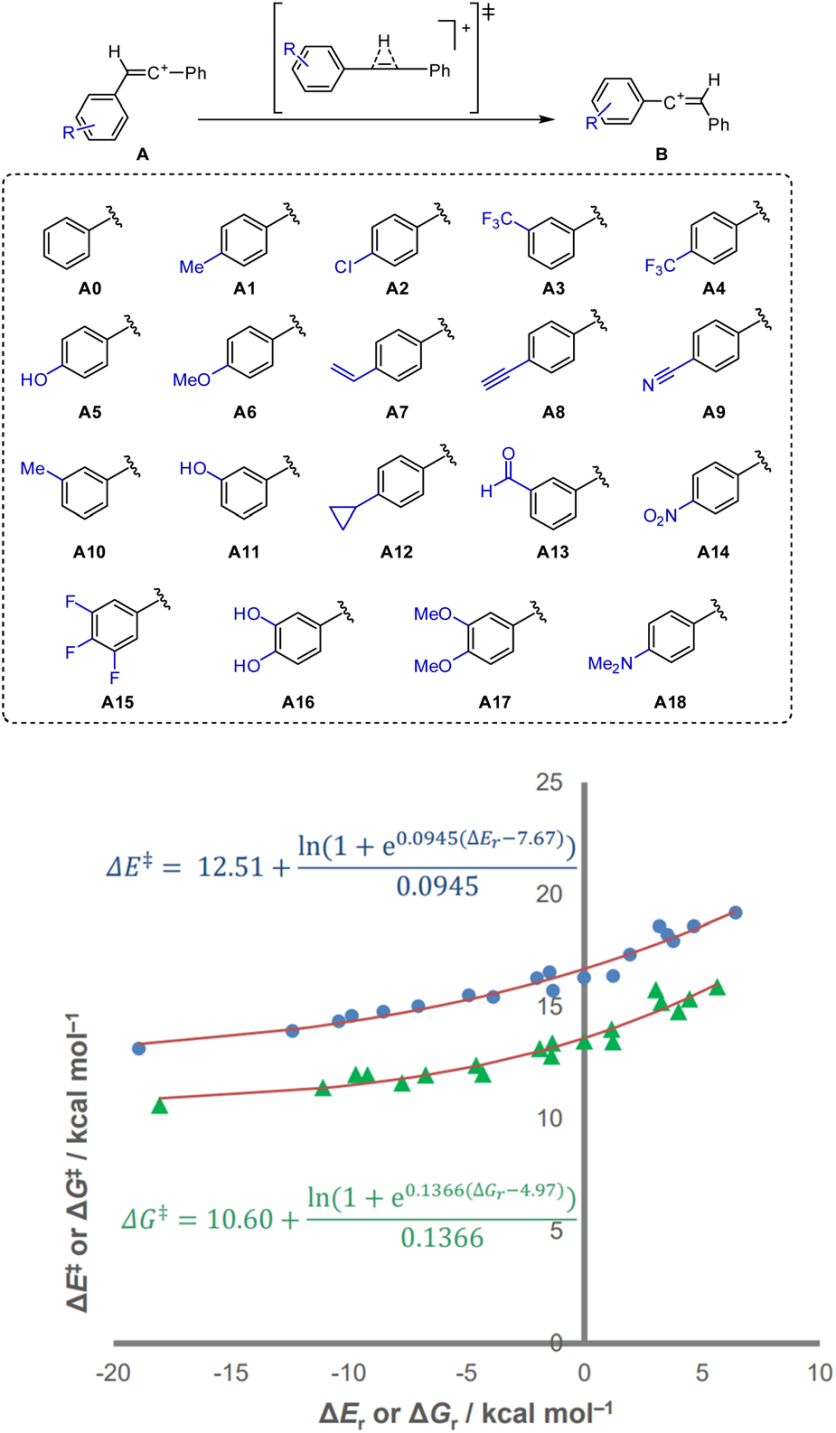
Substrates studied in the 1,2-hydride shift of 1,2-diarylethenyl cations. Non-linear relationship between the activation and reaction potential energies (circles) and Gibbs energies (triangles) of 1,2-hydride shifts on diarylethenyl cations listed in Scheme 1. *R*^2^ = 0.9625 (Δ*E*), 0.945 (Δ*G*).

In order to better explore the non-linear responses to demonstrate the emergence of the derived behaviour, we studied a series of model systems with tunable reaction energies while retaining a closer structural similarity among substrates. In this way, the kinetic–thermodynamic relationship of the family of reactions would clearly manifest the three parameters we defined. To see non-linearity clearly, the reactions should display easily modulated driving forces over a wide range of energies while retaining the same orbital makeup. For these reasons, we first explored a 1,2-hydride shift on a vinyl cation, a system adapted from a reported reaction.^27^ In order to determine the energies associated with this process, we performed density functional theory (DFT) calculations on the system. The reported energies were calculated, in the gas phase, in ORCA 6.0 (ref. 28) using the dispersion-corrected^29^ ωB97X-D3 functional^30^ and the def2-TZVPPD basis set^31^ for all atoms, with the RIJCOSX^32^ approximation.^33^ A family of 1,2-diarylethenyl cations was calculated with substitution on one of the aryl rings, together with the corresponding 1,2-hydride shift transition states and their products (Scheme 1).

The geometry of the transition state showed an essentially linear geometry of both central carbon atoms, best represented as formal *η*^2^ coordination of a diarylacetylene to the migrating hydrogen atom. In accordance with Hammond's postulate, the more favourable reactions (substrates A17 and A18) showed an earlier transition state,^30^ with the C–H distances reflecting the position of the transition state across all substrates. The corresponding plot showed a well-defined curve in a driving force range of around 25 kcal mol^−1^ (Scheme 1b, circles).^34^

The relationship evidences a large, thermodynamically independent contribution to the energy barrier of 12.5 kcal mol^−1^. This is presumably linked to the change in hybridisation and consequent weakening of the C–H bond. In addition, the curvature of the plot, defined by *θ*, is markedly visible. This would stem from the geometric proximity between the TS and the minima and an ensuing stronger impact of the same factors on the three stationary points. Such chemical proximity is captured by the coupling of the energy of the transition state to that of the connecting minima (*θ* = 0.0945 mol kcal^−1^), which is stronger than in the other studied reactions in this work. Finally, the *E*_eq_ of 7.7 kcal mol^−1^ hints at a degree of asymmetry in influence, showing how a hydrogen migration from a variable aryl-substituted sp^2^ carbon to a phenyl-substituted one responds differently because of the modified fragment. This results in the reorganisation, or the *E*_min_ of the reverse reaction (*E*_min_ − *E*_eq_), being only 4.8 kcal mol^−1^, showing that the thermodynamic dependence of 1,2-hydride shifts depends less on the substitution of the product carbocation and more on that of the reactant carbocation. Additional insights can be gained: if the *E*_min_ is lower when the migration occurs from the variable aryl, this suggests that the distortion will vary with the substituents more strongly (aligning better with thermodynamics) than if the substituents are located on a different ring, matching general chemical intuition. Through the thermodynamic-independent minimum preorganisation and reorganisation energies, *E*_min_ and *E*_eq_ can be instrumental in explaining whether the initial distortion is eased by the chemical modifications.

Plotting the same results with Gibbs energy also lead to an analysable plot (Fig. 4, triangles), although the dispersion of the data points was slightly higher, presumably owing to the additional non-trivial effects of the entropy and zero-point energy. The change in the parameters shows *G*_min_ (the Gibbs energy analogue of *E*_min_) to be 1.9 kcal mol^−1^ lower than *E*_min_, *G*_eq_ (the Gibbs energy analogue of *E*_eq_) 2.7 kcal mol^−1^ lower than *E*_eq_, and the resulting reorganisation energy (*G*_min_ − *G*_eq_) 0.8 kcal mol^−1^ higher than its potential energy analogue. These changes suggest that the inclusion of thermal terms, likely with the changes related to zero-point energy as the greatest contribution, significantly reduces the barriers at higher driving forces, and with the shift of *G*_eq_ – towards a more equal response – also conditions how the Δ*G*^‡^(Δ*G*_r_) function grows. However, the change in *θ* makes the activation energy much more responsive to changes in the thermodynamic driving force. On its own, *θ*_G_ accounts for a reduction of up to 2.5 kcal mol^−1^ at *G*_eq_ with respect to *θ*_*E*_, and a reduction of at least 1 kcal mol^−1^ between around Δ*G*_r_ = −15 and 25 kcal mol^−1^. Had *θ* remained the same, the stabilisation would have been lower than 1 kcal for Δ*G*_r_ ≥ 0 kcal mol^−1^, and actually destabilising for barriers above Δ*G*_r_ = 15.7 kcal mol^−1^. This exemplifies how the observed energy barriers arise from a complex interplay of the parameters, even when studying the effect of thermal corrections. The influence of entropy is known to explain patterns in selectivity,^35^ reaction kinetics,^36^ and reaction outcomes,^37^ with consequences on the free energy surface including the appearance of entropic intermediates.^38^ Here, entropy can be seen to change not just the absolute value of the activation energy itself, but also its responsiveness to reaction energy. The changes in the non-linear parameters can enable the analysis of this effect.

**Scheme 2:**
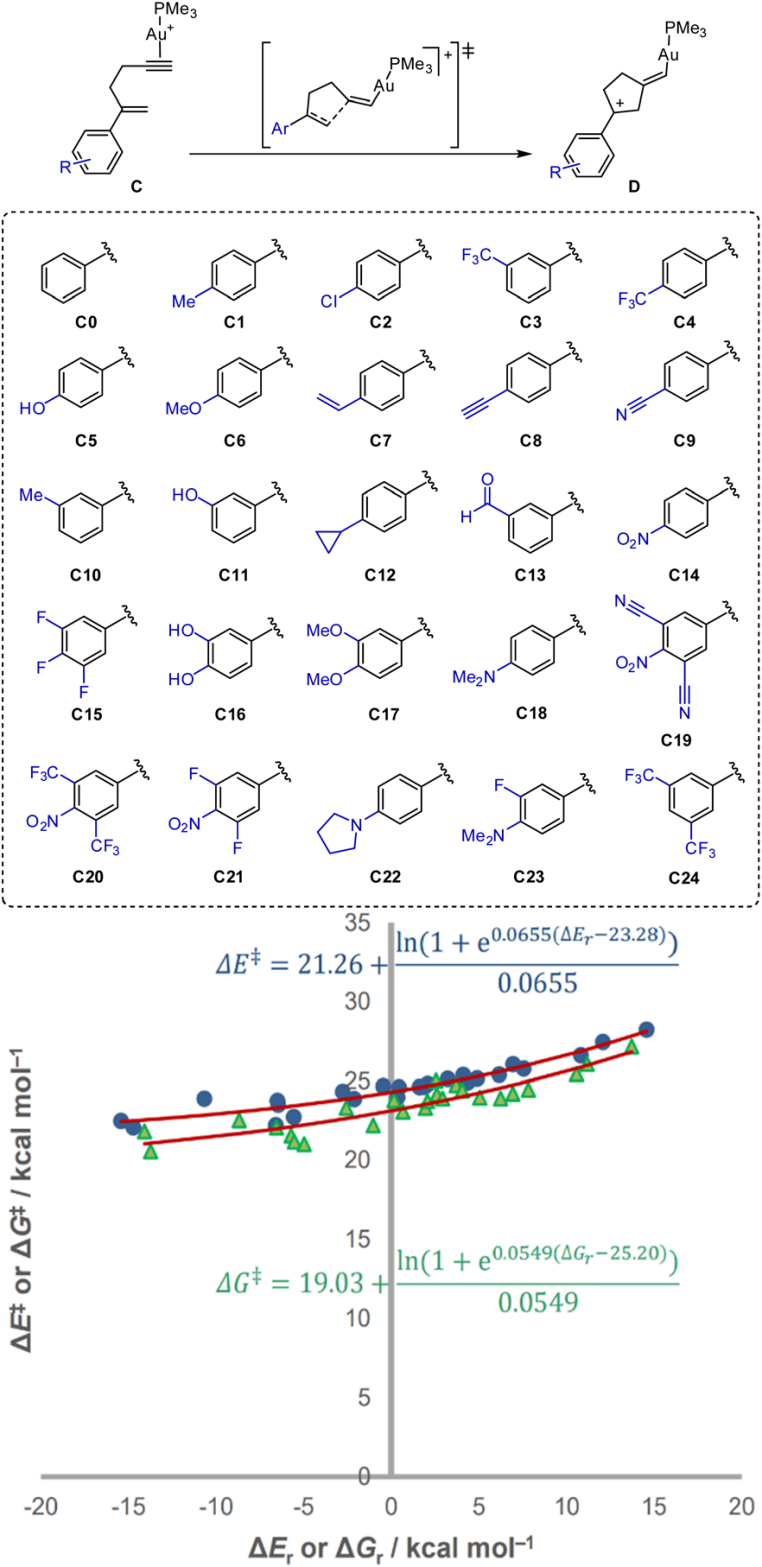
Substrates studied in the gold(i)-mediated 5-*exo*-dig cyclisation of 2-aryl-1,5-enynes. Non-linear relationship between the activation and reaction potential energies (circles) and Gibbs energies (triangles) of 5-*exo*-dig cyclisations of 1,5-enynes listed in Scheme 2. *R*^2^ = 0.9216 (Δ*E*), 0.8391 (Δ*G*).

We then investigated the kinetic–thermodynamic response in the gold(i)-catalysed 5-*exo*-dig cyclisation of a set of 2-aryl-1,5-enynes C, affording vinylgold(i) species D (Scheme 2). Using a trimethylphosphine ligand, as a reasonable model for most experimentally used phosphines, the aim of showing how the global kinetic–thermodynamic relationship can also be realised in synthetically relevant metal-catalysed transformations. Varying the substitution on the phenyl ring with a total of 25 different examples allowed modulating the reactivity of the alkene, as well as changing the stability of the product. This resulted in a reaction energy range of about 30 kcal mol^−1^, allowing the observation of the curvature in spite of the inherent complexity of the reaction and the inclusion of implicit solvation.

These reactions showed a shallower curvature, as evidenced by its lower value of *θ*, at 0.0655 mol kcal^−1^. In addition, the minimum preorganisation energy *E*_min_ was also particularly high, at 21.3 kcal mol^−1^. This was expected for this reaction, as even with highly stabilising factors in the product, the TS must be reached by significant distortion of the alkyne and the alkene. This distortion is independent of the stabilisation of the final vinylgold(i) species and is not as strongly affected by the electronics as the reaction energy itself. The parent enyne is always intrinsically robust, as electronic factors do not radically alter the stability of the styrene fragment. However, due to the benzylic cation, the stability of the product is very strongly affected by the electronics. For that reason, the reverse barrier should be generally much more dependent on thermodynamics than the forward reaction. Thus, in very disfavoured reactions, the energetic cost to reach the TS is much lower. That intuitive behaviour is observed in the notably lower reorganisation energy for the process (*E*_min_ − *E*_eq_) of −2.0 kcal mol^−1^. This would mean that the barrier would be zero for any reaction with a reaction energy higher than +53.2 kcal mol^−1^, were it to remain well-behaved and, in those, the product would return barrierlessly to the reactant configuration as the barrier would cease to exist. We can see that the parent enyne is intrinsically robust (it remains a stationary point at any driving force), at least with respect to modification on the ring, whereas the product carbocation is not intrinsically robust as it is not stable at all energy ranges (it would become a non-stationary geometry given a large enough destabilisation). Since the reactant and product of these reactions respond so differently to the same effect, consequently, the reaction is highly anisodromic, with an *E*_eq_ of 23.3 kcal mol^−1^. The Gibbs energy analogue similarly led to a plot with a higher variability. However, there was a slight decrease in the minimum preorganisation, with *G*_min_ = 19.0 kcal mol^−1^, accompanied by a slightly higher asymmetry in response (*G*_eq_ = 25.2 kcal mol^−1^) and a marginally shallower curvature, *θ*_G_ = 0.0549 mol kcal^−1^. Due to the greater scatter of the data and low local gradient, the significance of these small variations is harder to assess conclusively.

**Fig. 8:**
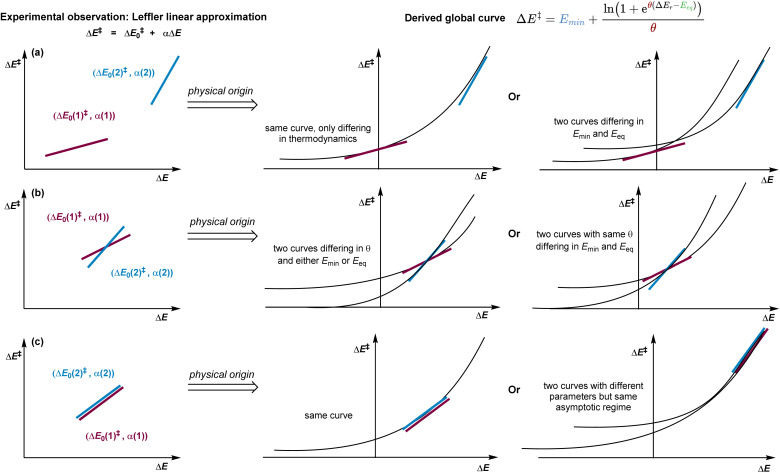
Conceptual map showing how classical rate–driving force relationships, particularly the Leffler equation, emerge as limiting cases of the global kinetic–thermodynamic model derived here. The figure illustrates how to causally interpret apparent linear relationships between the reaction rate and thermodynamic driving force within the broader, non-linear energy landscape governed by *E*_min_, *E*_eq_, and *θ*. (a) Two distinct linear plots may belong to the same curve, and so share all three parameters, or to two different ones. (b) Two linear plots that intersect may curve at similar rates, with only a minor difference in preorganisation, or respond very differently over a greater range of reaction energy. (c) Two apparently coincident linear plots may indeed belong to the same global non-linear energy relationship or happen to match an unrelated curve due to close asymptotic behaviour.

Wherever an apparent linear relationship is observed, leading to the use of the Leffler equation, the true physical origin of the observation can lead to conceptually very different interpretations of what happens in a given chemical system. Several of these examples are shown in Fig. 8, showing locally linear approximations may originate from different curves. Our model explains why rate–driving force relationships display the Brønsted slopes they do. The chemical insights gained from studying their origins are greater than from the simpler linear model because it enables the approach to rather complex questions. For instance, two non-crossing Leffler plots with different slopes are typically interpreted as representing distinct classes of reactions, each with its own set of thermodynamic-independent parameters. However, our model shows that such behaviour may also arise from a single reaction class sampled at different thermodynamic regions (Fig. 8a). Is the minimum preorganisation of the system always high, regardless of driving force (Fig. 8b)? Two apparently coincident linear plots may indeed belong to the same global non-linear energy relationship or happen to match an unrelated curve due to close asymptotic behaviour (Fig. 8c). These reinterpretations show that even when linear models like Leffler's appear valid, they reflect only a local view of a deeper structure. Our equation explains why they succeed where they do, providing a global context that transforms heuristics into physical understanding. Does the reaction ever become barrierless and perhaps only limited by the diffusion rate? Will extrapolating to more exergonic reactions allow a large improvement in selectivity between two competing barriers? As evidenced in the analysis of the reactions in this work, the magnitude of the thermodynamic-independent component in the preorganisation or reorganisation can be fully consistent with chemical intuition. Furthermore, in any case, the values of the parameters may explain patterns that are not immediately obvious, but are critical to explaining why an energy barrier has a particular energy. Understanding the origin of a given linear approximation can provide many more insights into a system of interest. Having examined systems with clearly visible curvature, we now turn to cases where the response appears deceptively linear and show how our model can still extract mechanistic insight.

**Scheme 3:**
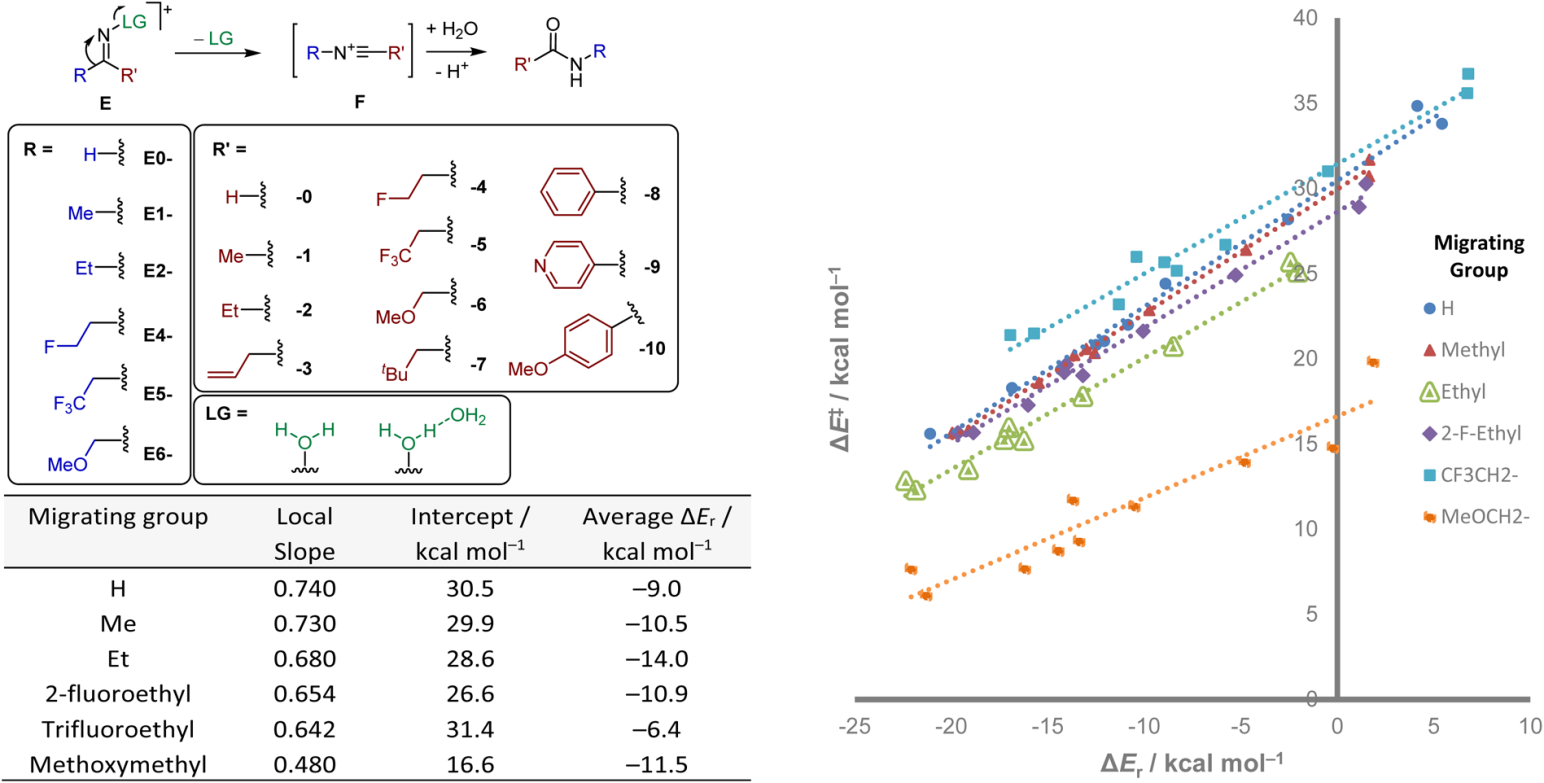
Substrates studied in the initial step of the Beckmann rearrangement, with a pair of numbers defining the substituents. Plots of six different reaction sets, each with a different migrating *R* group (labelled), with linear fitting shown. Each point is a reaction with a perturbation on the non-migrating *R*′ group. The leaving group is modelled as a water dimer. Observed Brønsted slopes and intercepts (intrinsic barriers) from a linear fitting.

We considered the Beckmann rearrangement,^39^ a classical reaction which would support a similar 1,2-migration to the hydride shift albeit concerted with the concomitant cleavage of a leaving group. In order to find several related curves, we calculated six different sets of reactions, each of them with a different migrating group. Within each set, the migrating group was kept constant while varying the adjacent substituent of the imine (Scheme 3). As the reaction involves the initial formation of a nitrilium species (F), these were used as the thermodynamic reference for the products.

For each migrating group, an essentially linear plot was obtained, suggesting that the value of *θ* was significantly lower in all cases (Scheme 3). However, each of the observed barriers at Δ*E*_r_ = 0 and Brønsted slopes were significantly different, the latter varying from 0.48 to 0.74. While the curvature is not directly observable, the general trend that higher intercepts display steeper gradients would be a result of the similarities of the reaction sets. Methoxymethyl substitution (E6-0 through E6-10) is the only example where several points (corresponding to E6-6, E6-9 and E6-10) appear significantly separated from the rest of the points, suggesting either the influence of non-perfect synchronisation in the process or the existence of markedly different stabilisation factors in some substrates.^40^ As all linear plots constitute the local tangents to the corresponding curves at the average of the reaction energies of the data points, they cannot be directly compared without accounting for reaction energy. However, if one parameter is fixed, the other two become solvable with a linear plot. Studying what values of *E*_min_, *E*_eq_ and *θ* could match the observed linear fits by keeping one constant allowed determination of the trifluoroethyl migrating group as an outlier, displaying a higher *E*_min_ than any other reaction class. Similarly, the only main difference with the methoxymethyl migrating group, and responsible for its much lower activation energies across the board, seems to arise from a lower reorganisation energy, and so its more isodromic energy response. This is precisely where the value of a physically derived model emerges: even where behaviour appears linear, the framework reveals the hidden structure beneath. This analysis shows how misleading apparent linearity can be, and how only a global model reveals what parameters actually control reactivity. In practical terms, this enables synthetic chemists to tune barriers through design principles derived from physical causality, not regression.

To the question of how one may increase or lower the rate and selectivity of a given reaction, the answer involves the interplay of the exergonicity of the reaction with respect to *G*_eq_, the curvature of the non-linear Δ*G*^‡^(Δ*G*_r_) function and the proximity to its asymptotic behaviour. This contributes to understanding the patterns in general chemical intuition, where different reactions respond differently to modifications, while providing a measure of which aspects are unnecessary to modify. The non-linear equation, even when only implicitly observed due to local linearity, provides insights into how reactivity can be modulated. In a highly exergonic regime, while a simpler linear plot might suggest modifications could be futile, our equation would suggest that an increase in *θ* could be very effective, attempting to make the barrier more sensitive to the stabilising factors through changes such as steric environment or strain. This breaks away from the reaction energy-based approach with a linear model (and mostly intractable outliers), instead providing alternatives for synthetic chemists to improve even already very exergonic reactions and rationally lower their barriers. This approach can be of utility regardless of the exergonicity of the reaction. Its application in new methodology will be ultimately determined by the understanding of how different factors affect the parameters that guide the non-linear kinetic–thermodynamic relationship. The answers to these questions should lie closer to the chemical properties, as the meaning of *θ*, *E*_min_ and *E*_eq_ is inherently fundamentally distinct from heuristic interpolation. Full accommodation of asynchronous processes is ongoing in our laboratory and would further broaden the scope of applicability.

## 3 Conclusions

We have derived a universal non-linear relationship between the reaction energies and activation energies of a reaction set that not only captures rate–driving force relationships across thermodynamic regimes but also explains why classical models like Bell–Evans–Polanyi relationships and Marcus equation succeed or fail in specific limits. The derivation is grounded in a formulation stemming from the principle of microscopic reversibility, with an exponential dependence on the energies, and otherwise minimal assumptions about the reactions themselves. Consequently, the observed Brønsted slopes and intercepts of the linear approximations of a system constitute local descriptors, dependent on the underlying parameters and the studied thermodynamic range. The full non-linear understanding allows global comparisons between very different ranges of reaction energies and reaction families. We expect that factors that affect the sensitivity of energy barriers to the reaction energies to correlate strongly with the parameters (*θ*, *E*_min_ and *E*_eq_) governing the non-linear relationship, and therefore only indirectly with the local slopes and intercepts. This deductively reached global non-linear model allows direct mechanistic comparisons between different reaction classes across wide ranges of reaction energies. In systems with visible curvature, such as 1,2-hydride shifts and gold(i)-catalysed cyclisations, the model enables the extraction of the three parameters and how energy barriers depend on their combined effects. Even where linear fitting may appear adequate, this is deceptively simple and the non-linear model clarifies how differences in parameters, not just thermodynamic range, govern barrier variation. This reframing offers a more unified and practical understanding of reactivity, particularly for identifying outlier behaviour and guiding new reaction discovery, by moving from local interpolation to derived reactivity parameters. While the model can reinterpret past observations, such as the non-linear rate–driving force behaviour in iron(iv) oxo-mediated reactions, its greater value lies in guiding new mechanistic design and uncovering reactivity principles that remain hidden under traditional frameworks. It remains open for future work to discern what chemical properties impact the values of *θ*, *E*_min_ and *E*_eq_, together with how their rational modulation can lead to its adoption in organic and organometallic chemistry. Through the exploration of the kinetic–thermodynamic relationship with the new overarching model, we acquire a better perspective on the importance of driving force in reaction kinetics. This work invites to rethink conventional strategies and intuition on the effects of different types of perturbation on chemical reactions: what appears locally adequate may conceal global inconsistency, and this model equips chemists to see beyond the curve, to recognise structure where none was thought to exist.

## Author contributions

E.G.-P. derived the equation and led the study. G.Q. supervised the project. Both authors jointly wrote the manuscript.

## Note after first publication

This article replaces the version published on 3rd September 2025, where eqn (5) was appearing incorrectly, this has now been resolved.

## Conflicts of interest

There are no conflicts to declare.

## Supplementary Material

SC-OLF-D5SC04829J-s001

## Data Availability

All computed data are available within the paper and its SI. See DOI: https://doi.org/10.1039/d5sc04829j. A collection of the computational data underlying this work has been made available in the ioChem-BD repository at https://doi.org/10.19061/iochem-bd-6-443.^41^
