# Supplementary material for: The global kinetic–thermodynamic relationship derived from first principles
Source: Chem Sci. 2025 Aug 21;16(37):17494–505. doi: 10.1039/d5sc04829j (PMC12406046; doi:10.1039/d5sc04829j)
Supplement: SC-016-D5SC04829J-s001 [file SC-016-D5SC04829J-s001.pdf]

Supplementary Information (SI) for Chemical Science.
This journal is © The Royal Society of Chemistry 2025

Supporting Information

Global Kinetic-Thermodynamic Responses:

Derivation of a General Non-Linear Equation and Demonstrations on Chemical Reactions

Eduardo Garcia-Padilla* and Guanqi Qiu*

*Eduardo Garcia-Padilla − Max-Planck-Institut für Kohlenforschung,
Kaiser-Wilhelm-Platz 1,
45470, Mülheim an der Ruhr, Germany;
orcid.org/0000-0001-9715-7992; Email: garciapadilla@kofo.mpg.de

*Guanqi Qiu − Max-Planck-Institut für Kohlenforschung,
Kaiser-Wilhelm-Platz 1,
45470, Mülheim an der Ruhr, Germany;
orcid.org/0000-0003-3818-3896; Email: gqiu@kofo.mpg.de

Table of Contents

1. Methods and Additional Calculations 3

1.1. General Methods 3

1.2. Additional Considerations in the Derived Constraints and Equations . . 3

1.3. On Monotonicity and Observed Inverted Regions 6

1.4. Vinyl Cation 1,2-Hydride Shift 6

1.5. Statistical Analysis of Curvature 10

1.6. Beckmann Rearrangements 11

2. Computed Structures and Energies 16

3. References 25

4. Coordinates of Stationary Points 26

1. Methods and Additional Calculations

1.1. General Methods

The calculations in this paper were performed with Gaussian 09,[1] and ORCA 6.0.[2] For geometry optimisations and frequency calculations of all organic structures (structures **A**, **B**, **E** and **F** and transition states), Gaussian09 was used. These geometry optimisations were done using the ωB97X-D functional and the 6-31+G(d,p) basis set[3] on all atoms. The geometry optimisations and frequencies of all gold(I) species (**C**, **D** and transition states) were carried out in ORCA 6.0 using the ωB97X-D3 functional, the def2-SVP basis set on all non-metal atoms and the def2-TZVP basis set on gold (with def2-ECP),[4] in addition to the autoaux auxiliary basis sets.[5] All calculations were carried out in the gas phase with the exception of all calculations on **C**, **D** and their transition states, where implicit solvation was modelled with the SMD model[6] for dichloromethane. For all single-point energy calculations at a higher level of theory, ORCA 6.0 was used. Higher level of theory single-point calculations were performed using ωB97X-D3,[7] using RIJCOSX,[8] with the def2-TZVPPD basis set and autoaux auxiliary basis sets, in all cases employing tight convergence criteria for the SCF. Frequency analysis on all minima confirmed the absence of imaginary frequencies and transition states were relaxed by IRC calculations. Free energies as reported in the manuscript are reported at 298 K and 1 atm. A collection of all computational data in ioChem-BD is freely accessible at https://doi.org/10.19061/iochem-bd-6-443.[9]

1.2. Additional Considerations in the Derived Constraints and Equations

For a given set S of similar reactions, the kinetic-thermodynamic function should map every reaction energy in the set to its activation energy: $\forall\, r \in S,\ f_S : \Delta E_r(r) \rightarrow \Delta E^{\ddagger}(r)$. Such a reaction family only requires the differences between reactions to primarily cause a change in reaction energy, with the responsiveness to such energy changes preserved across the reactions. Isodromic reactions preserve the same responsiveness for both the forward and backward reactions, with the reactants and products being analogous or identical and with a thermoneutral identity reaction in the set: $(A \sim A_{-1} \wedge \exists\, \mathrm{r}^i \in A \mid \mathrm{r}^i = \mathrm{r}^i_{-1}) \implies f_A = f_{A,-1}$.

The origin of E_{eq} from the anisodromic reaction families Upon integrating $[\Delta E^{\ddagger}]'(\Delta E_r + E_{eq})$ and $[\Delta E^{\ddagger}_{-1}]'(\Delta E_r - E_{eq})$, which are known to be equal, the difference must only be an integration constant. The difference between their limits at negative infinity, $E_{preorg} - E_{preorg,-1}$, and at positive infinity, $(E_{reorg} + E_{eq}) - (E_{reorg,-1} - E_{eq})$, are equal and satisfy Equation S1:

$$E_{preorg} - E_{preorg,-1} - E_{reorg} + E_{reorg,-1} = 2\,E_{eq} \qquad \text{(S1)}$$

By simple substitution of $E_{preorg,-1} = E_{reorg}$ and $E_{reorg,-1} = E_{preorg}$, one obtains the definition of E_{eq} shown in Equation 5 from the manuscript.

The barrier at E_{eq} can be shown to depend on the curvature, as the gradient is bound to (0,1) and the curvature is self-similar in a single-factor interpolation. As the curvature, $[\Delta E^{\ddagger}]''$, is evenly symmetric about E_{eq}, all terms higher than second order of the general Taylor expansion of $\Delta E^{\ddagger}$ around E_{eq} must be even exponents (Equation S2):

$$\Delta E^{\ddagger}(\Delta E_r - E_{eq}) = (E_{preorg} + \Delta E^{\ddagger}_{eq}) + \frac{1}{2}(\Delta E_r - E_{eq}) + \frac{1}{2}[\Delta E^{\ddagger}]''(E_{eq})(\Delta E_r - E_{eq})^2 + O(\Delta E_r^4) \qquad \text{(S2)}$$

The zeroth order term must be related to how quickly the function grows from its asymptote at negative infinity. As the function is monotonically increasing, its value at 0 must be strictly larger than E_{preorg}, and this difference (shown as $\Delta E^{\ddagger}_{eq}$) can be shown to depend on the curvature of the function. As $[\Delta E^{\ddagger}]'$ is bounded by two horizontal asymptotes, the integral of $[\Delta E^{\ddagger}]''$ is constrained over its domain. However, the integral of $[\Delta E^{\ddagger}]'$ is subject only to a linear growth limit and so, in principle, $\Delta E^{\ddagger}_{eq}$ can take any value. As $[\Delta E^{\ddagger}]''(E_{eq})$

approaches infinity, the transition between the limits of $[\Delta E^{\ddagger}]'(E_{eq})$ approaches a step function between 0 and 1. At the extreme, the point $(E_{eq}, \Delta E^{\ddagger}(E_{eq}))$ would become non-differentiable, and would be equal to the horizontal asymptote ($\Delta E^{\ddagger}_{eq}$ = 0). In the reverse scenario, as $[\Delta E^{\ddagger}]''(E_{eq})$ approaches 0, $[\Delta E^{\ddagger}]'$ would approach the value of 0.5 across its domain. Hence, the integral of $[\Delta E^{\ddagger}]'$ from –∞ to 0, and therefore the value of $\Delta E^{\ddagger}_{eq}$, would approach positive infinity, confirming that the curvature of the function must be strictly larger than zero and that the value of $\Delta E^{\ddagger}_{eq}$ and the curvature at that point are not independent.

In the derivation of the equation, expressing as the energy differences and rearranging affords half the harmonic mean as the relationship between the exponentials of the TS and the minima (Equation S5). This shows exactly how θ describes the coupling between the two minima and the transition state as a single-factor interpolation with an exponential dependence on energy.

$$\mathrm{e}^{-\theta\Delta E^{\ddagger}} + \mathrm{e}^{-\theta\Delta E^{\ddagger}_{-1}} = 1 \quad \text{(S3)}$$

$$\mathrm{e}^{-\theta(E_{TS}-E_1)} + \mathrm{e}^{-\theta(E_{TS}-E_2)} = 1 \quad \text{(S4)}$$

$$\frac{1}{\mathrm{e}^{-\theta E_1}} + \frac{1}{\mathrm{e}^{-\theta E_2}} = \frac{1}{\mathrm{e}^{-\theta E_{TS}}} \quad \text{(S5)}$$

From Equation S$$, however, re-expressing $\mathrm{e}^{-\theta\Delta E^{\ddagger}_{-1}}$ as $\mathrm{e}^{-\theta(\Delta E^{\ddagger}-\Delta E_r)}$, an explicit form for the kinetic-thermodynamic relationship can be reached through basic algebraic manipulations (Equations S6–S9)

$$\mathrm{e}^{-\theta\Delta E^{\ddagger}} + \mathrm{e}^{-\theta(\Delta E^{\ddagger}-\Delta E_r)} = 1 \quad \text{(S6)}$$

$$\mathrm{e}^{-\theta\Delta E^{\ddagger}} + \mathrm{e}^{-\theta\Delta E^{\ddagger}}\mathrm{e}^{\theta\Delta E_r} = 1 \quad \text{(S7)}$$

Multiplying both sides by $\mathrm{e}^{\theta\Delta E^{\ddagger}}$:

$$1 + \mathrm{e}^{\theta\Delta E_r} = \mathrm{e}^{\theta\Delta E^{\ddagger}} \quad \text{(S8)}$$

$$\Delta E^{\ddagger} = \frac{\ln(1+\mathrm{e}^{\theta\Delta E_r})}{\theta} \quad \text{(S9)}$$

Note that from Equation S9 one can also show the difference between the barrier and reaction as being dependent on the coupling between the barrier and the reaction energy, in a different form:

$$\mathrm{e}^{-\theta\Delta E_r}\mathrm{e}^{-\theta\Delta E^{\ddagger}} + \mathrm{e}^{-\theta\Delta E^{\ddagger}} = \mathrm{e}^{-\theta\Delta E_r} \quad \text{(S10)}$$

$$\mathrm{e}^{-\theta\Delta E^{\ddagger}} - \mathrm{e}^{-\theta\Delta E_r} = -\mathrm{e}^{-\theta\Delta E_r}\mathrm{e}^{-\theta\Delta E^{\ddagger}} \quad \text{(S11)}$$

The derivative of the general kinetic-thermodynamic relationship with respect to reaction energy is a sigmoid curve (Equation S12), which is oddly symmetric about (E_{eq}, 0.5) and satisfies all derived constraints required by the kinetic-thermodynamic relationship, including its limits at negative and positive infinity.

$$\frac{d\Delta E^{\ddagger}}{d\Delta E_{\mathrm{r}}} = \frac{1}{1+\mathrm{e}^{-\theta(\Delta E_r - E_{eq})}} \quad \text{(S12)}$$

From studying the general equation, the meaning of the curvature in particular can be assessed. While E_{min} and E_{eq} were directly addressed in the derivation of the constraints, and the curvature was known to be non-zero, the way in which θ modulates the barriers only arises from the derived equation. Replacing θ by $\frac{\ln 2}{E^{\ddagger}_{eq}}$ in shows that the global kinetic-thermodynamic relationship is equivalent to Equation S13, where $E^{\ddagger}_{eq}$ is the value of $\Delta E^{\ddagger}$ at the equilibrium energy.

$$\Delta E^{\ddagger} = E_{min} + E^{\ddagger}_{eq}\log_2\left(1 + 2^{\frac{\Delta E_r - E_{eq}}{E^{\ddagger}_{eq}}}\right) \quad \text{(S13)}$$

In an isodromic reaction where E_{eq} = 0, the second derivative with respect to ΔE_r of this function would be the following:

$$\frac{d^2\Delta E^{\ddagger}}{d\Delta E_r^2} = \frac{\ln 2}{\Delta E_0^{\ddagger}} \frac{2^{\frac{\Delta E_r}{E_0^{\ddagger}}}}{(1 + 2^{\frac{\Delta E_r}{E_0^{\ddagger}}})^2} = \theta \frac{2^{\frac{\Delta E_r}{E_0^{\ddagger}}}}{(1 + 2^{\frac{\Delta E_r}{E_0^{\ddagger}}})^2} \qquad \text{(S14)}$$

Note that the first terms of the series expansion around ΔE_r = 0 of the curvature are $\frac{\ln 2}{4E_0^{\ddagger}} + \frac{\ln^3 2}{16\left(E_0^{\ddagger}\right)^3}\Delta E_r^2 + O(\Delta E_r^4)$ and in particular, the first term shows how the curvature at ΔE_r = 0 (or at E_{eq} for anisodromic reactions) is proportional to θ. As the associated barrier at that point, $E_{eq}^{\ddagger}$, is itself inversely proportional to θ, it can be seen that the second derivative of the global kinetic-thermodynamic response is closely linked to the value of the barrier (Equation S15). It is the dependence on θ that determines the value of the barrier $E_{eq}^{\ddagger}$, and so θ is the more fundamental parameter, defining the curvature of the energy relationship and as a result determining the activation energy at E_{eq}. The specific characteristics of a process that lead it to respond more or less sharply to changes in the reaction energy are the origin of this barrier.

$$\frac{d^2\Delta E^{\ddagger}}{d\Delta E_r^2} = \theta e^{-\theta\Delta E^{\ddagger}}\left(1 - e^{-\theta\Delta E^{\ddagger}}\right) = \theta e^{-\theta\Delta E^{\ddagger}} e^{-\theta\Delta E_{-1}^{\ddagger}} \qquad \text{(S15)}$$

This substitution helps to understand the nature of θ, which defines not only the curvature but the additional barrier associated to the thermoneutral process (or at E_{eq} for an anisodromic process). Nonetheless, the re-expression of the normalised transition probabilities as a function not of θ but of $E_{eq}^{\ddagger}$ (equation S16, from equation 7) –for an idealised isodromic system with no ΔE_r-independent behaviour– shows that the energy response can also be thought of as an $E_{eq}^{\ddagger}$-normalised binary partitioning between the forward and backward reaction. The anisodromic equivalent would be analogous, but only acts on the ΔE_r-dependent part of the barrier (equation S5). This new expression highlights how the implicit role of θ modulates energy barriers with respect to that at E_{eq}.

$$e^{-\theta\Delta E^{\ddagger}} + e^{-\theta\Delta E_{-1}^{\ddagger}} = 2^{\frac{\Delta E^{\ddagger}}{E_{eq}^{\ddagger}}} + 2^{\frac{\Delta E_{-1}^{\ddagger}}{E_{eq}^{\ddagger}}} = 1 \qquad \text{(S16)}$$

We can also represent the base 2 relationship for the generalised anisodromic systems. This would be analogous to the isodromic case but operating on the reaction energies displaced by E_{eq} and acting only on the thermodynamic-dependent component of the barrier (subtracting the thermodynamic-independent E_{min}). Thus, with non-zero E_{min} and E_{eq}, the binary partitioning dependence only acts on the ΔE_r-dependent part of the barrier (equation S13, note that both $\Delta E^{\ddagger}$ and $\Delta E^{\ddagger}_{-1}$ are presented as functions, where ΔE_r is the reaction energy of the forward reaction). The total sum is conserved at 1, and is intrinsically dependent on the proportions between the barriers and the barrier at equilibrium as a result of the symmetry constraint.

$$e^{-\theta\left(\Delta E^{\ddagger}(\Delta E_r - E_{eq}) - E_{min}\right)} + e^{-\theta\left(\Delta E_{-1}^{\ddagger}(-\Delta E_r + E_{eq}) - (E_{min} - E_{eq})\right)}$$

$$= 2^{\frac{\Delta E^{\ddagger}(\Delta E_r - E_{eq}) - E_{min}}{E_{eq}^{\ddagger}}} + 2^{\frac{\Delta E_{-1}^{\ddagger}(-\Delta E_r + E_{eq}) - (E_{min} - E_{eq})}{E_{eq}^{\ddagger}}} = 1 \qquad \text{(S17)}$$

The behaviour of the kinetic-thermodynamic relationship is, hence, described by the combined effects of three parameters. E_{min}, corresponds to a driving force-independent preorganisation energy; E_{eq}, corresponding to the point at which the influence of the reactant and product stabilisation factors act equally on the TS; and θ, which modulates how strongly the barrier couples to the energy of the minima through the curvature (Figure 6). (E_{min} – E_{eq}) is the reorganisation energy, equivalent to the E_{min} of the reverse process. The physical meaning of θ would seem to be related to the distance between the TS and the minima along the reaction coordinate.

1.3. On Monotonicity and Observed Inverted Regions

Even in more challenging cases, such as when local behaviour of the kinetic-thermodynamic responses appears erratic, or shows very divergent gradients outside of the range from 0 to 1, understanding why non-linear kinetic-thermodynamic responses exist can prove to be valuable. Erratic or noisy behaviour originates from non-trivial changes in the thermodynamic-independent reaction parameters.

When perturbations define a set of similar reactions, the perturbations may induce non-thermodynamic changes that happen to be proportional to the thermodynamic force changes, thereby disrupting the expected monotonic barrier-energy relationship. The observed rate-driving force relationship thereby no longer follows the direct determination of the energy barrier from the reaction energy. Instead, it transitions into a system-dependent relationship, where reactant, TS, and product no longer interpolate directly. Monotonicity is lost, meaning that the kinetic-thermodynamic response can no longer be causally defined. Instead, the outcome becomes non-interpolative and system-dependent, not unlike the Hammett-type correlation, where the observed kinetic trends emerge from indirect effects rather than from a fundamental energy response. This can be seen, for example, in nonadiabatic electron transfer (ET), where *concomitant* changes in factors such as the Franck–Condon integral with thermodynamic driving force disrupt the monotonicity of the kinetic-thermodynamic Hammond interpolation, and may result in an observed inverted region.

Similarly, in apparent outliers caused by asynchronicity, the activation barrier is no longer solely determined by the reaction energy but mediated by other factors indirectly or even coincidentally affected by reaction energy, leading to non-monotonic and/or context-dependent trends (this has been explored in the past for the nitroalkane anomaly). Our derived equation for the kinetic-thermodynamic response remains essential in defining the nature of the energy relationships. As reaction phase asynchronicity may complicate reaction behaviour and disrupts global monotonicity, it makes it even more critical to precisely determine what is happening. Unlike heuristic slopes and intercepts, these parameters offer a causal architecture for reactivity. Knowing one does not merely predict a barrier, it explains why it behaves as it does.

Very large or negative local gradients would suggest that external factors such as asynchronicity are affecting the degree to which Hammond's postulate holds, thus hindering the analysis of the TS as an interpolation of factors from the minima. Alternatively, the reactions may be too distinct in nature, with different factors stabilising their TS geometries and so not being directly comparable. Other experimentally relevant questions would include the consequences in reaction rates, where additional contributions parallel to those arising from the energy barrier, such as diffusion rate limit or the quantum yield of a photochemical process, must also be taken into account when studying the overall observed rate.

1.4. Vinyl Cation 1,2-Hydride Shift

In all calculations on the 1,2-hydride shift (Table S1), the conformation used for the product was that stemming directly from the transition state, as any bond rotation would constitute a (subsequent) secondary process and could add some minor noise to the data.

Table S1. Reaction energies and activation energies for the 1,2-hydride shifts.

Reactant	ΔE_r / kcal mol^{-1}	$\Delta E^{\ddagger}$ / kcal mol^{-1}	ΔG_r / kcal mol^{-1}	$\Delta G^{\ddagger}$ / kcal mol^{-1}
**A0**	0.0	16.3	0.0	13.5
**A1**	-3.9	15.4	-4.3	12.0
**A2**	-1.5	16.5	-1.4	13.4
**A3**	3.5	18.2	3.3	15.2
**A4**	3.8	17.9	4.0	14.8
**A5**	-8.5	14.8	-7.7	11.6
**A6**	-10.4	14.3	-9.7	12.0
**A7**	-4.9	15.5	-4.6	12.4

**A8**	-2.0	16.3	-1.9	13.1
**A9**	4.7	18.6	4.5	15.4
**A10**	-1.3	15.7	-1.4	12.8
**A11**	1.2	16.4	1.2	13.4
**A12**	-7.0	15.0	-6.7	12.0
**A13**	1.9	17.3	1.2	14.0
**A14**	6.4	19.2	5.7	15.9
**A15**	3.2	18.6	3.0	15.8
**A16**	-9.9	14.6	-9.2	12.0
**A17**	-12.4	13.9	-11.1	11.4
**A18**	-18.9	13.1	-18.0	10.6

Table S2. C–H distances in each of the TS of dehydrostilbenium.

	**r_1, ArC–H / Å**	**r_2, PhC–H / Å**	**$r_2 - r_1$ / Å**
**TSAB0**	1.291	1.292	0.001
**TSAB1**	1.272	1.316	0.044
**TSAB2**	1.289	1.298	0.009
**TSAB3**	1.321	1.269	-0.052
**TSAB4**	1.325	1.264	-0.061
**TSAB5**	1.255	1.339	0.084
**TSAB6**	1.251	1.346	0.095
**TSAB7**	1.268	1.322	0.054
**TSAB8**	1.284	1.303	0.019
**TSAB9**	1.331	1.261	-0.070
**TSAB10**	1.284	1.302	0.018
**TSAB11**	1.301	1.284	-0.017
**TSAB12**	1.257	1.336	0.079
**TSAB13**	1.310	1.278	-0.032
**TSAB14**	1.351	1.246	-0.105
**TSAB15**	1.322	1.269	-0.053
**TSAB16**	1.253	1.342	0.089
**TSAB17**	1.248	1.350	0.102
**TSAB18**	1.236	1.367	0.131

In agreement with Hammond's postulate, the position of the hydrogen atom correlated strongly with $\Delta E^{\ddagger}$, showing (at least in this range) a strong linear correlation (Table S2, Figure S1). Note that, with the same set of data, plotting ΔE_r instead against the difference of C–H distances already displays very significant non-linearity. As the energy is a function of molecular geometry, this is consistent with the position of the hydrogen atom, and so the geometry of the TS, undergoing the same non-linear transformation as the energies themselves. A linear correlation can also be found by plotting against the absolute value of the C–H distance (Figure S2).

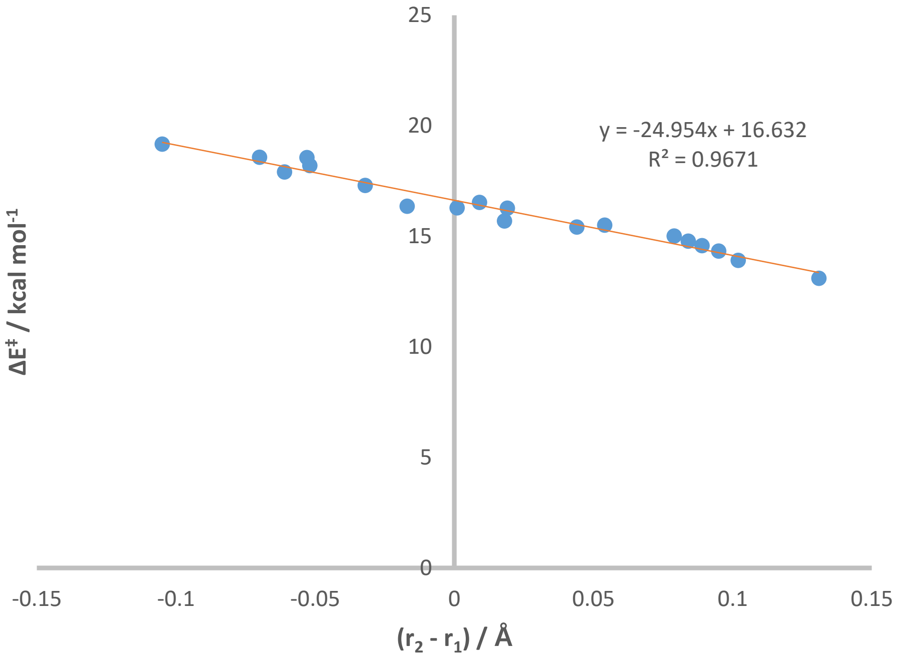

Figure S1. Linear correlation between $\Delta E^{‡}$ and the difference of the two C–H distances.

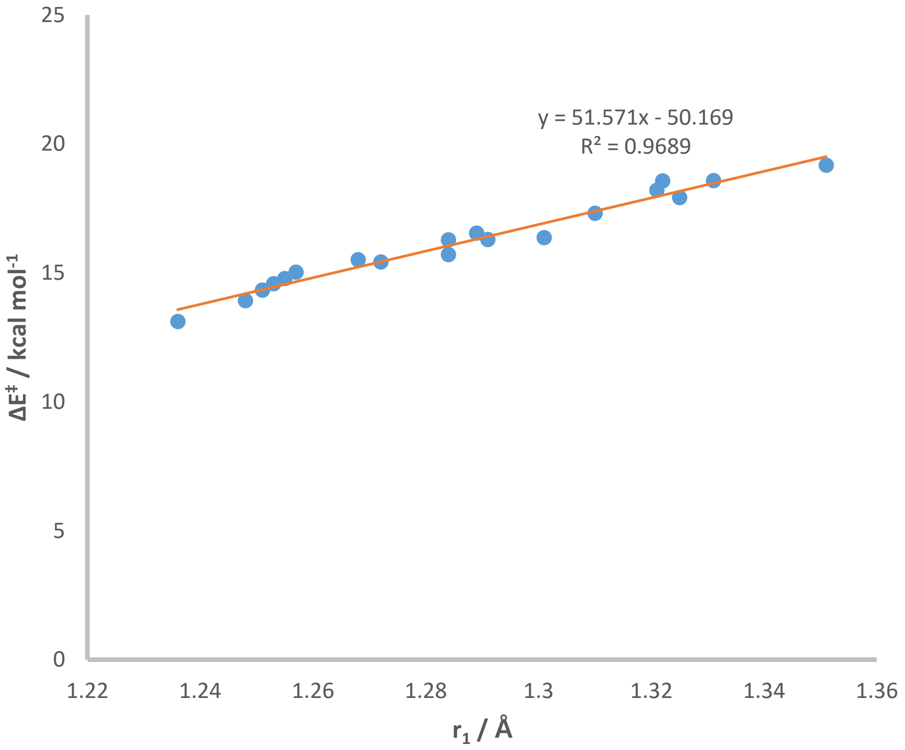

Figure S2. Linear correlation between $\Delta E^{‡}$ and the absolute C–H distance r_1 (ArCH).

We also naïvely fitted the dataset for the 1,2-hydride shift with a linear relationship (Leffler equation, Figure S3), with the standard Marcus equation (Figure S4) and with a quadratic relationship (asymmetric Marcus equation, Figure S5). From the Leffler equation, the deviation from linearity is apparent. The slope lower than 0.5 reflects the fact that E_{eq} is higher than the average energy of the data points.

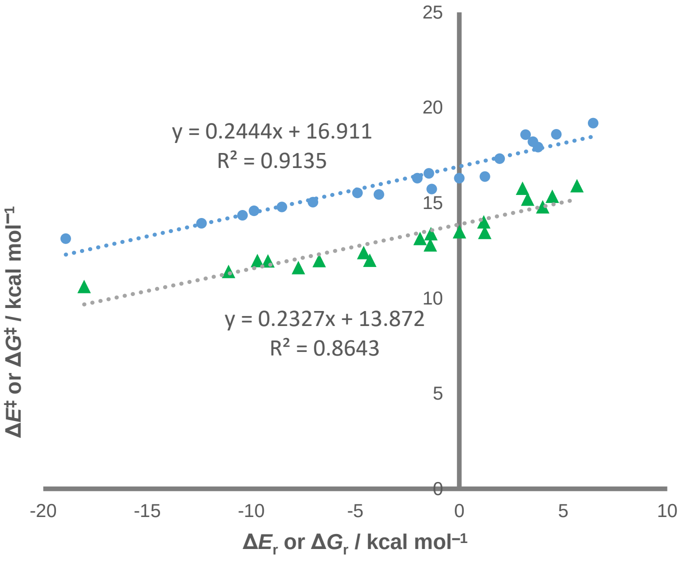

Figure S3. Fitting a linear relationship to the energy data of the 1,2-hydride shift.

Note how the standard Marcus equation, while curved, fails to fit the data due to its requirement that the slope is 0.5 at $\Delta E_r = 0$, which assumes the reactions are isodromic. The corresponding Marcus intrinsic barriers would be $\Delta E_0^‡ = 17.45$ kcal mol^{-1} and $\Delta G_0^‡ = 14.39$ kcal mol^{-1}. These values significantly deviate from the known values at 0, given that the reaction is anisodromic. Instead, our non-linear equation predicts the values at 0 to be 16.69 kcal mol^{-1} and 13.60 kcal mol^{-1} (thermoneutral reaction calculated with DFT: 16.29 kcal mol^{-1} and 13.48 kcal mol^{-1}).

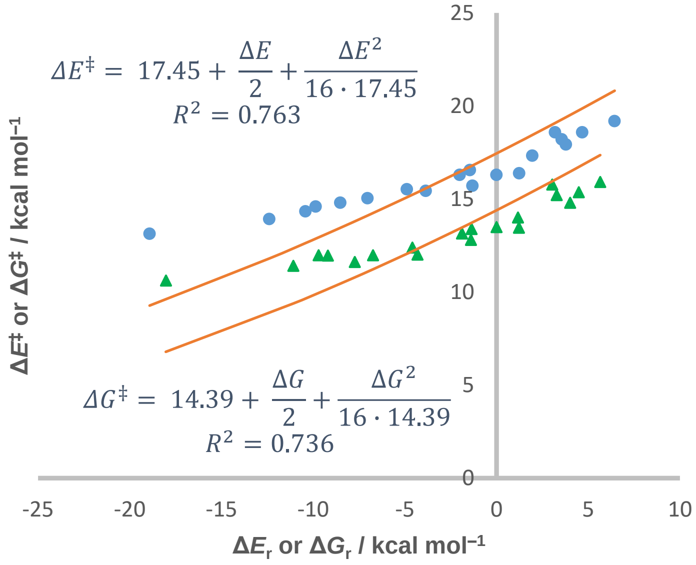

Figure S4. Fitting the Marcus equation to the energy data of the 1,2-hydride shift.

If we instead use a free quadratic fit, which accounts for anisodromic reactions, the fit is substantially better with an R^2 value marginally worse than our non-linear equation. The intercepts at 0 are also much closer to the real values, as expected. If we now consider that the "anisodromic Marcus intrinsic barrier" occurs where the tangent has a slope of 0.5, the values would be $\Delta E^{\ddagger}_{eq,Quad}$ = 21.74 kcal mol^{-1} and $\Delta G^{\ddagger}_{eq,Quad}$ = 17.48 kcal mol^{-1}, with the values derived from our non-linear equation [$\Delta E^{\ddagger}_{eq} = E_{min} + \ln(2)/\theta$] being $\Delta E^{\ddagger}_{eq}$ = 19.84 kcal mol^{-1} and $\Delta G^{\ddagger}_{eq}$ = 15.67 kcal mol^{-1}. The discrepancy is minor although the quadratic fit has $E_{eq,Quad}$ = 12.16 kcal mol^{-1} (actual E_{eq} = 7.67 kcal mol^{-1}) and $G_{eq,Quad}$ = 9.17 kcal mol^{-1} (actual G_{eq} = 4.97 kcal mol^{-1}).

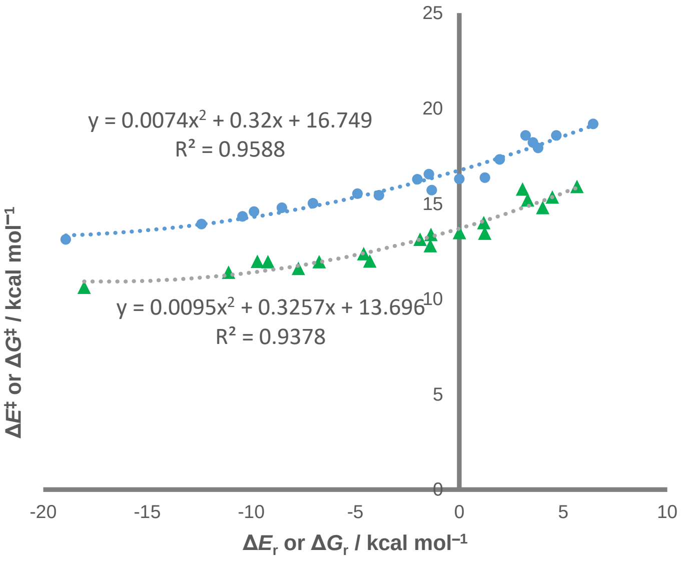

Figure S5. Fitting a quadratic relationship to the energy data of the 1,2-hydride shift.

1.5. Statistical Analysis of Curvature

We performed some basic statistical analysis to confirm that the visible curvature in the plot of $\Delta E^{\ddagger}$ against ΔE_r (or its free energy analogue) was significant enough to directly fit a non-linear function. In order to do this, we followed two complementary approaches: (1) multilinear regression with ΔE_r and $(\Delta E_r)^2$ to see if inclusion of the latter term is statistically significant, and (2) analysis of covariance (ANCOVA) by dividing the data in two sets ($\Delta E_r < 0$ and $\Delta E_r \geq 0$). We then repeated the same procedure with the corresponding free energies. The coefficients and p values are tabulated in Tables S3 and S4, both for potential energy or Gibbs free energy. In both cases, the inclusion of a quadratic term was found to be statistically significant. The groupings also showed statistically significant changes in the local linear slope and, as expected, the vertical displacement of the two lines was not significant (as their intercept with the y axis should be the same for the two groups).

Table S3. Results of the regression with the energies of 1,2-hydride shifts including a quadratic term.

	**Δ*E***		**Δ*G***	
	**Coefficient**	***p* value**	**Coefficient**	***p* value**
**Intercept**	16.75	$2.7 \cdot 10^{-27}$	13.70	$3.9 \cdot 10^{-25}$
**ΔE_r or ΔG_r**	0.320	$1.4 \cdot 10^{-10}$	0.326	$1.4 \cdot 10^{-9}$
**ΔE_r^2 or ΔG_r^2**	$7.37 \cdot 10^{-3}$	$6.8 \cdot 10^{-4}$	$9.52 \cdot 10^{-3}$	$5.0 \cdot 10^{-4}$

Table S4. Results of the analysis of covariance with the energies of 1,2-hydride shifts. M is the grouping term (0 if $\Delta E_r < 0$ and 1 if $\Delta E_r \geq 0$).

	**Δ*E***		**Δ*G***	
	**Coefficient**	***p* value**	**Coefficient**	***p* value**
**Intercept**	16.35	$2.0{\cdot}10^{-22}$	13.12	$1.65{\cdot}10^{-15}$
**ΔE_r or ΔG_r**	0.182	$7.3{\cdot}10^{-8}$	0.146	$3.3{\cdot}10^{-5}$
**M**	-0.0729	0.79	0.326	0.35
**(ΔE_r or ΔG_r)·M**	0.308	$1.9{\cdot}10^{-4}$	0.307	$2.1{\cdot}10^{-3}$

These results strongly supported that the curvature was pronounced enough for the data to be fitted quadratically. As the non-linear equation is similarly defined by three parameters, directly fitting our non-linear model is equally justified.

1.6. Beckmann Rearrangements

IRC analysis of the Beckmann rearrangement with some R' groups, using a water dimer leaving group, showed initial migration of the R group with concomitant dissociation of the leaving group, only then followed by a second step: a barrierless water attack on the intermediate nitrilium cation. Constituting two separate steps with independent enough nature to constitute an entropic intermediate, and likely a substrate- and model-dependent stationary point (implicit solvation, water cluster, etc.), we studied the first step exclusively, the formation of the nitrilium ion, for the determination of kinetic-thermodynamic parameters (Table S5).

Table S5. Reaction and activation energies for Beckmann rearrangements with a water dimer leaving group.

**Reactant**	**ΔE_r / kcal mol^{-1}**	**$\Delta E^‡$ / kcal mol^{-1}**	**ΔG_r / kcal mol^{-1}**	**$\Delta G^‡$ / kcal mol^{-1}**
**E0-0b**	5.4	33.8	-13.8	28.1
**E1-0b**	1.7	31.7	-20.7	26.6
**E2-0b**	-2.4	25.7	-25.7	20.1
**E3-0b**	-1.6	23.9	-23.9	18.2
**E4-0b**	1.5	30.3	-21.7	24.7
**E5-0b**	6.8	36.7	-16.0	31.2
**E6-0b**	-0.2	14.8	-24.0	10.0
**E7-0b**	-4.9	22.8	-27.8	17.2
**E0-1b**	-12.1	21.0	-34.9	15.4
**E0-2b**	-14.3	19.4	-37.6	13.8
**E0-3b**	-8.9	24.4	-33.6	17.3
**E0-4b**	-10.8	22.0	-33.9	18.2
**E0-5b**	-2.5	28.2	-27.1	21.1
**E0-6b**	4.1	34.8	-20.5	27.2
**E0-8b**	-16.9	18.3	-40.4	12.3
**E0-9b**	-12.5	20.8	-36.6	14.1
**E0-10b**	-21.1	15.6	-45.9	9.7
**E1-1b**	-13.6	20.2	-36.5	15.3
**E1-2b**	-15.5	18.6	-38.8	13.3
**E1-3b**	-9.7	22.9	-34.6	15.4
**E1-4b**	-12.6	20.3	-36.2	16.9
**E1-5b**	-4.7	26.4	-29.6	20.0
**E1-6b**	1.6	30.7	-23.4	24.5
**E1-8b**	-19.1	15.8	-43.0	9.9
**E1-9b**	-13.0	20.6	-37.5	14.5
**E1-10b**	-19.9	15.7	-43.7	10.3

**E2-1b**	-17.3	15.3	-40.5	10.6
**E2-2b**	-19.1	13.5	-41.9	8.1
**E2-3b**	-13.2	17.8	-38.6	11.1
**E2-4b**	-16.2	15.2	-39.4	11.6
**E2-5b**	-8.5	20.7	-32.5	14.8
**E2-6b**	-2.1	25.1	-27.3	18.2
**E2-8b**	-21.8	12.3	-45.1	7.7
**E2-9b**	-17.0	16.0	-41.0	11.4
**E2-10b**	-22.4	12.8	-46.5	8.4
**E4-1b**	-14.1	19.2	-37.1	14.3
**E4-2b**	-16.0	17.3	-38.5	12.9
**E4-3b**	-10.1	21.6	-34.1	14.2
**E4-4b**	-13.2	19.0	-36.8	15.6
**E4-5b**	-5.3	24.9	-29.0	18.9
**E4-6b**	1.1	28.9	-23.9	22.3
**E4-8b**	-18.9	15.7	-42.6	10.9
**E4-9b**	-14.0	19.7	-37.9	14.2
**E4-10b**	-19.7	15.7	-43.6	11.0
**E5-1b**	-9.0	25.7	-33.0	19.8
**E5-2b**	-11.3	23.2	-33.6	18.7
**E5-3b**	-5.8	26.7	-29.7	20.7
**E5-4b**	-8.3	25.2	-32.2	21.0
**E5-5b**	-0.5	31.0	-24.6	25.0
**E5-6b**	6.7	35.6	-18.0	29.7
**E5-8b**	-15.7	21.5	-40.2	16.5
**E5-9b**	-10.4	26.0	-35.2	21.1
**E5-10b**	-16.9	21.4	-40.4	17.1
**E6-1b**	-14.5	8.8	-38.0	4.8
**E6-2b**	-16.2	7.7	-39.5	4.4
**E6-3b**	-10.5	11.4	-35.5	6.8
**E6-4b**	-13.4	9.3	-35.7	7.1
**E6-5b**	-4.8	14.0	-28.7	10.0
**E6-6b**	1.9	19.8	-24.4	14.8
**E6-8b**	-21.3	6.1	-45.9	3.6
**E6-9b**	-13.7	11.7	-37.8	9.9
**E6-10b**	-22.1	7.7	-45.9	5.1

Are these slopes and intercepts completely independent of each other? While technically representing six different reactions due to having different migrating groups, there could be underlying patterns in their energy responses that allow a greater understanding than exclusive linear fitting. The underlying parameters that are the cause of these local observations are likely to show some broad similarities. Given a group of similar reaction sets, approximating the non-linear relationship through assuming common or closely related parameters is feasible. As E_{min} is likely associated to preorganisation changes of the reaction distinct from the migration itself, it should remain similar across all reactions. Thus, assuming that the E_{min} values are equal to 0, in order to aid us in understanding how the other parameters change, solving for θ and E_{eq} was now possible, with the results collected in Table S6.

Table S6. Approximated parameters for the Beckmann rearrangement assuming E_{min} = 0 kcal mol^{-1}.

Migrating group	θ / mol kcal$^{–1}$	E_{eq} / kcal mol$^{–1}$
H	0.0575	–27.1
Me	0.0604	–27.0
Et	0.0658	–23.7

2-fluoroethyl	0.0554	–24.5
Trifluoroethyl	0.0379	–21.9
Methoxymethyl	0.0650	–10.3
Trifluoroethyl[a]	0.0570	–16.7

[a]Assuming E_{min} = 9.0 kcal mol^{-1}.

In the case of the Beckmann rearrangement, the underlying assumption that the values of both E_{min} and θ should both remain similar across the different curves could be confirmed with values around E_{min} = 0 and θ = 0.06. The application of the equation in these two families of reactions sheds light on the important role of the parameters in describing their characteristics: some barriers may be higher or lower, more or less sensitive to thermodynamics due to unique combinations of factors. Overall, these explorations of the Beckmann rearrangement exemplify how even when essentially only local linearity is found, the interplay of the parameters can be studied if several sets of reactions of the same family are compared to each other.

When assuming the same E_{min} value for each reaction set, the main difference between each curve was determined by E_{eq}, whereas the value of θ remained roughly around the same range. The main difference in θ was found for trifluoroethyl, suggesting that the destabilisation of the Beckmann rearrangement transition states also carried an additional constant component. This suggests that no matter whether one tries to match the value of θ or the value of E_{min} for all reaction sets, the trifluoroethyl migrations always remain an outlier with respect to the other reactions. The reason for the high electronic energy barriers associated to most sets of Beckmann rearrangements are primarily due to E_{eq} being large and negative. Reactants such as methoxymethyl, have significantly lower barriers precisely due to the more equal response. This is kinetically extremely important, as among all reaction sets, and regardless of the specific model used for the protonated leaving group, thermoneutral reactions of **E6** would proceed at much lower temperatures than any of the other counterparts. That provides more information about the Beckmann rearrangement than would otherwise be understood from the linear fitting alone. Inevitably, defining three parameters from a local linear approximation cannot be achieved without further information on the non-linear response. Nevertheless, as knowing any parameter affords the others, it is possible to identify outliers in reaction sets and obtain more meaningful information from the sets of reactions than would otherwise be inferable from local slopes alone. Therefore, even when rate–driving force relationships appear nearly linear, the full curvature can be resolved through comparative analysis, revealing hidden mechanistic structure that local models obscure. The new insight paves the way for deeper understanding of activation energy modulation.

We studied whether the choice of guessed parameter made any difference to the result of the analysis. Instead of fixing the value of E_{min} to 0, we made θ constant for all reactions at 0.0608 mol kcal^{-1}, i.e. the average of all other reaction sets. Calculating E_{min} and E_{eq} shows the trifluoroethyl group as an outlier with the only significantly large E_{min} (Table S7), albeit the reverse reaction is unaffected and would show a typical preorganisation ($E_{min} - E_{eq}$). In addition, the methoxymethyl group is found to have very low preorganisation and reorganisation energy contributions, likely due to the very high stabilisation of the migrating fragment in the TS. The fact that these conclusions are analogous with the previous assumptions shows that the starting points to approximate the non-linear parameters are interchangeable and given a known group of sets known to be more substantially closely related (in this case: hydrogen, methyl and ethyl), insights can be reproducibly derived from the approximated parameters.

Table S7. Approximated parameters for the Beckmann rearrangement assuming θ = 0.0608 mol kcal^{-1}.

Migrating group	E_{min} / kcal mol^{-1}	E_{eq} / kcal mol^{-1}	$E_{min} - E_{eq}$ / kcal mol^{-1}
H	1.3	–26.2	27.4
Me	0.1	–26.9	27.0
Et	–1.2	–24.5	23.3

2-fluoroethyl	1.8	–23.3	25.0
Trifluoroethyl	10.1	–16.1	26.2
Methoxymethyl	–0.6	–10.2	9.6

While the negative E_{min} values in some entries, in this case, could be the result of minor error, a negative E_{min} is not aphysical, corresponding to reactions that become barrierless beyond a certain thermodynamic driving force due to the disappearance of the saddle point.**Error! Bookmark not defined.** Such behaviour might initially seem unexpected for the Beckmann rearrangement; it is worth noting that this is not predicted to occur in the chemically relevant ΔE_r range. In the case of ethyl, for example, extrapolated barrierless reactions would only be observed for reactions perfectly analogous to the fitted ones but with ΔE_r below −66 kcal mol^{-1}, which is presumably unattainable with standard substituent modifications.

We also explored what would happen if a much smaller θ was used to model the data. Assuming θ = 0.0300 resulted in a greater spread of the values of E_{min}, with the values (excluding the known E_{min} outlier, trifluoroethyl) spreading over a range of over 10 kcal mol^{-1} (Table S8). Without an additional deeper exploration of whether E_{min} are expected to vary so much, it cannot be ascertained whether this could be closer to the real parameters. However, given how an alternative solution resulted in apparently almost constant E_{min} and θ across the reactions, we consider θ = 0.0608 to be more likely in the absence of more evidence. Critically, however, this does not affect the detection of trifluoroethyl as an outlier in E_{min}, being around the order of 10 kcal mol^{-1} higher than expected regardless of the chosen θ when compared to the rest of the data.

Table S8. Approximated parameters for the Beckmann rearrangement assuming θ = 0.0300 mol kcal^{-1}.

Migrating group	E_{min} / kcal mol^{-1}	E_{eq} / kcal mol^{-1}	$E_{min} - E_{eq}$ / kcal mol^{-1}
**H**	-21.3	-43.8	22.5
**Me**	-21.7	-43.7	22.0
**Et**	-18.6	-35.3	16.7
**2-fluoroethyl**	-17.1	-36.0	18.9
**Trifluoroethyl**	-7.1	-26.0	18.8
**Methoxymethyl**	-11.2	-8.8	-2.3

Modelling a protonated substrate is much more correct to be shown with a coordinated water, as in all previous examples in Table S8 and those discussed in the paper, or an alternative coordinated base. The Gibbs free energy of coordination of a water molecule to **E0-0** through **E6-10** always resulted in a stabilisation greater than 11 kcal mol^{-1} and an average of 15.8 kcal mol^{-1} (greater for potential energy, as expected, at 25.9 kcal mol^{-1}). This leads to a water dimer formally being the leaving group, even if the reported energies are referenced to two separate molecules of water.

For this reason, we also calculated sets with a single molecule of water as a leaving group, with the energies tabulated in Table S9.

Table S9. Reaction and activation energies for Beckmann rearrangements with H_2O leaving group.

Reactant	ΔE_r / kcal mol^{-1}	$\Delta E^‡$ / kcal mol^{-1}	ΔG_r / kcal mol^{-1}	$\Delta G^‡$ / kcal mol^{-1}
**E0-0**	1.9	19.7	-10.5	13.9
**E0-1**	-13.3	9.4	-28.6	4.2
**E0-2**	-14.9	8.1	-30.9	3.1
**E0-3**	-9.1	13.5	-26.6	7.2
**E0-4**	-12.8	9.8	-28.7	4.7
**E0-5**	-5.1	15.4	-22.6	8.4
**E0-6**	-9.0	10.8	-25.0	5.8
**E0-8**	-17.6	6.3	-33.5	1.9

**E0-9**	-14.8	7.8	-30.1	3.7
**E0-10**	-19.6	5.4	-36.6	1.3
**E1-0**	0.1	17.2	-15.3	12.1
**E1-1**	-13.5	7.5	-28.8	3.9
**E1-2**	-15.1	5.9	-30.9	1.6
**E1-3**	-8.7	10.8	-25.7	5.0
**E1-4**	-13.3	7.9	-29.7	3.0
**E1-5**	-5.7	12.9	-23.1	7.4
**E1-6**	-8.4	7.6	-25.0	2.8
**E1-8**	-16.0	5.8	-32.2	2.8
**E1-9**	-13.4	7.2	-29.5	3.6
**E1-10**	-18.7	4.0	-34.9	0.9
**E2-0**	-3.4	11.4	-19.0	6.1
**E2-1**	-16.4	3.6	-32.1	-0.1
**E2-2**	-17.7	2.3	-33.5	-1.1
**E2-3**	-11.6	6.7	-29.3	1.3
**E2-4**	-16.0	3.8	-30.7	1.0
**E2-5**	-8.9	8.1	-25.2	3.3
**E2-6**	-11.6	2.7	-28.1	-1.4
**E2-8**	-18.2	4.0	-34.1	1.7
**E2-9**	-15.8	4.6	-31.5	2.5
**E2-10**	-20.7	2.8	-36.4	2.0
**E4-0**	-0.6	15.3	-16.3	10.0
**E4-1**	-14.4	6.2	-30.2	2.0
**E4-2**	-15.7	4.7	-31.9	0.5
**E4-3**	-9.4	9.4	-26.4	3.8
**E4-4**	-14.1	6.5	-30.4	1.9
**E4-5**	-6.6	11.1	-23.4	5.0
**E4-6**	-9.2	5.7	-25.3	1.2
**E4-8**	-16.3	5.6	-32.6	2.3
**E4-9**	-13.7	6.6	-29.2	3.4
**E4-10**	-18.8	4.0	-35.6	1.0

Similarly estimating the parameters assuming that the value of θ had remained constant at 0.0608 afforded essentially unchanged E_{eq} but with a now negative E_{min} (Table S10). This means that for a sufficiently unstable starting material, the N-O bond would not be present in a stationary geometry and so would undergo spontaneous dissociation. The values of E_{min} are around 11.5 kcal mol^{-1} lower than with the water dimer, with similar E_{eq}, although the ethyl migration seems slightly more different to the rest; nevertheless, this was probably due to the much noisier data for ethyl migrations (**C2-n**). In all four curves, the point for the methoxymethyl spectator group (R') was an outlier.

Table S10. Observed Brønsted slopes and intercepts (intrinsic barriers) from a linear fitting with H_2O leaving group. Approximated parameters for the Beckmann rearrangement assuming θ = 0.0608 mol kcal^{-1}.

Migrating group	Local Slope	Intercept / kcal mol^{-1}	Average ΔE_r / kcal mol^{-1}	E_{min} / kcal mol^{-1}	E_{eq} / kcal mol^{-1}
**H**	0.697	18.8	-11.7	-9.8	-25.4
**Me**	0.708	17.0	-11.6	-12.2	-26.1
**Et**	0.535	12.9	-14.3	-8.8	-16.6
**2-fluoroethyl**	0.635	15.5	-12.2	-9.8	-21.3

2. Computed Structures and Energies

Table S11. Hydride shifts. Low level of theory potential and free energies reported at ωB97X-D/6-31+G(d,p). HLT energies reported at ωB97X-D3/def2-TZVPPD.

Code	Filename	E / Ha	G / Ha	E_{HLT} / Ha
**A0**	SM0dhs	-539.63548	-539.46954	-539.82306
**A1**	SM1dhs	-578.94770	-578.75634	-579.14792
**A2**	SM2dhs	-999.21087	-999.05696	-999.43302
**A3**	SM3dhs	-876.59950	-876.43444	-876.93497
**A4**	SM4dhs	-876.59800	-876.43442	-876.93354
**A5**	SM5dhs	-614.84219	-614.67367	-615.06466
**A6**	SM6dhs	-654.13405	-653.93854	-654.36828
**A7**	SM7dhs	-617.01409	-616.81838	-617.22826
**A8**	SM8dhs	-615.75074	-615.57869	-615.96836
**A9**	SM9dhs	-631.83286	-631.67138	-632.05846
**A10**	SM10dhs	-578.94679	-578.75508	-579.14699
**A11**	SM11dhs	-614.84030	-614.67145	-615.06250
**A12**	SM12dhs	-656.31637	-656.09143	-656.54391
**A13**	SM13dhs	-652.91956	-652.74695	-653.15399
**A14**	SM14dhs	-744.06642	-743.90210	-744.34525
**A15**	SM15dhs	-837.25049	-837.11292	-837.57509
**A16**	SM16dhs	-690.04626	-689.87470	-690.30262
**A17**	SM17dhs	-768.62382	-768.39910	-768.90461
**A18**	SM18dhs	-673.58523	-673.35143	-673.81931
**B0**	Prod0dhs	-539.63548	-539.46954	-539.82306
**B1**	Prod1dhs	-578.95367	-578.76304	-579.15407
**B2**	Prod2dhs	-999.21247	-999.05841	-999.43535
**B3**	Prod3dhs	-876.59308	-876.42845	-876.92932
**B4**	Prod4dhs	-876.59124	-876.42731	-876.92751
**B5**	Prod5dhs	-614.85522	-614.68545	-615.07825
**B6**	Prod6dhs	-654.15022	-653.95359	-654.38489
**B7**	Prod7dhs	-617.02204	-616.82585	-617.23606
**B8**	Prod8dhs	-615.75410	-615.58187	-615.97156
**B9**	Prod9dhs	-631.82530	-631.66413	-632.05103
**B10**	Prod10dhs	-578.94888	-578.75729	-579.14910
**B11**	Prod11dhs	-614.83821	-614.66937	-615.06053
**B12**	Prod12dhs	-656.32770	-656.10229	-656.55513
**B13**	Prod13dhs	-652.91602	-652.74464	-653.15090
**B14**	Prod14dhs	-744.05526	-743.89219	-744.33499
**B15**	Prod15dhs	-837.24400	-837.10669	-837.57000
**B16**	Prod16dhs	-690.06141	-689.88882	-690.31833
**B17**	Prod17dhs	-768.64326	-768.41648	-768.92435
**B18**	Prod18dhs	-673.61489	-673.37969	-673.84946
**TSAB0**	TS0dhs	-539.60525	-539.44379	-539.79709
**TSAB1**	TS1dhs	-578.91885	-578.73296	-579.12333
**TSAB2**	TS2dhs	-999.18001	-999.03113	-999.40666
**TSAB3**	TS3dhs	-876.56594	-876.40567	-876.90596
**TSAB4**	TS4dhs	-876.56487	-876.40628	-876.90498
**TSAB5**	TS5dhs	-614.81456	-614.65112	-615.04110
**TSAB6**	TS6dhs	-654.10720	-653.91545	-654.34544
**TSAB7**	TS7dhs	-616.98510	-616.79437	-617.20354
**TSAB8**	TS8dhs	-615.72045	-615.55342	-615.94241
**TSAB9**	TS9dhs	-631.79896	-631.64262	-632.02885
**TSAB10**	TS10dhs	-578.91742	-578.73034	-579.12196

**TSAB11**	TS11dhs	-614.80982	-614.64561	-615.03642
**TSAB12**	TS12dhs	-656.28828	-656.06821	-656.51997
**TSAB13**	TS13dhs	-652.88747	-652.72014	-653.12640
**TSAB14**	TS14dhs	-744.03131	-743.87220	-744.31468
**TSAB15**	TS15dhs	-837.21614	-837.08304	-837.54549
**TSAB16**	TS16dhs	-690.01890	-689.85152	-690.27939
**TSAB17**	TS17dhs	-768.59767	-768.37696	-768.88242
**TSAB18**	TS18dhs	-673.56056	-673.33075	-673.79841

Table S12. Gold(I)-catalysed 5-*exo*-dig cyclisations. Low level of theory potential and free energies reported at ωB97X-D3/def2-SVP+def2-TZVP[Au]. HLT energies reported at ωB97X-D3/def2-TZVPPD. Implicit solvation (dichloromethane, SMD) is included in all energies.

Code / Filename	E / Ha	G / Ha	E_{HLT} / Ha
**C0**	-1060.56759	-1060.29803	-1061.31870
**C1**	-1099.84958	-1099.55406	-1100.64202
**C2**	-1520.02453	-1519.76579	-1520.93379
**C3**	-1397.28218	-1397.00995	-1398.43744
**C4**	-1397.28051	-1397.00903	-1398.43675
**C5**	-1135.71929	-1135.44518	-1136.56065
**C6**	-1174.97965	-1174.67856	-1175.86152
**C7**	-1137.89232	-1137.59134	-1138.72594
**C8**	-1136.63504	-1136.35782	-1137.46877
**C9**	-1152.71299	-1152.44631	-1153.56940
**C10**	-1099.85027	-1099.55348	-1100.64270
**C11**	-1135.72079	-1135.44655	-1136.56200
**C12**	-1177.16861	-1176.83858	-1178.04117
**C13**	-1173.77898	-1173.50074	-1174.66073
**C14**	-1264.86156	-1264.59149	-1265.85623
**C15**	-1357.95916	-1357.71517	-1359.07822
**C16**	-1210.87068	-1210.59272	-1211.80128
**C17**	-1289.38708	-1289.05546	-1290.39895
**C18**	-1194.41554	-1194.07592	-1195.30893
**C19**	-1449.12847	-1448.86366	-1450.33160
**C20**	-1938.26392	-1937.99001	-1940.06555
**C21**	-1463.11457	-1462.86151	-1464.35195
**C22**	-1271.77703	-1271.40077	-1272.74956
**C23**	-1293.54412	-1293.21431	-1294.56161
**C24**	-1733.99167	-1733.71772	-1735.55268
**D0**	-1060.57193	-1060.29906	-1061.31812
**D1**	-1099.85754	-1099.56032	-1100.64538
**D2**	-1520.02643	-1519.76620	-1520.93120
**D3**	-1397.27862	-1397.00709	-1398.43086
**D4**	-1397.27672	-1397.00502	-1398.42891
**D5**	-1135.73371	-1135.45795	-1136.57114
**D6**	-1174.99404	-1174.69181	-1175.87177
**D7**	-1137.89792	-1137.59595	-1138.72667
**D8**	-1136.63659	-1136.35853	-1137.46544
**D9**	-1152.70838	-1152.44043	-1153.55958
**D10**	-1099.85430	-1099.55716	-1100.64197
**D11**	-1135.72087	-1135.44656	-1136.55896
**D12**	-1177.17730	-1176.84699	-1178.04556
**D13**	-1173.77881	-1173.50104	-1174.65557
**D14**	-1264.85437	-1264.58392	-1265.84415

**D15**	-1357.95432	-1357.71085	-1359.07125
**D16**	-1210.88161	-1210.60273	-1211.81013
**D17**	-1289.39975	-1289.06824	-1290.40930
**D18**	-1194.44279	-1194.10158	-1195.33240
**D19**	-1449.10960	-1448.84615	-1450.30836
**D20**	-1938.24736	-1937.97489	-1940.04628
**D21**	-1463.09921	-1462.84657	-1464.33468
**D22**	-1271.80512	-1271.42672	-1272.77414
**D23**	-1293.56679	-1293.23379	-1294.57861
**D24**	-1733.98365	-1733.71083	-1735.54159
**TSCD0**	-1060.52889	-1060.25968	-1061.28053
**TSCD1**	-1099.81090	-1099.51800	-1100.60405
**TSCD2**	-1519.98433	-1519.72492	-1520.89458
**TSCD3**	-1397.23927	-1396.96803	-1398.39704
**TSCD4**	-1397.23885	-1396.96924	-1398.39673
**TSCD5**	-1135.68178	-1135.40932	-1136.52529
**TSCD6**	-1174.94087	-1174.64288	-1175.82409
**TSCD7**	-1137.85241	-1137.55283	-1138.68667
**TSCD8**	-1136.59504	-1136.31881	-1137.42930
**TSCD9**	-1152.67216	-1152.40738	-1153.52896
**TSCD10**	-1099.81041	-1099.51601	-1100.60358
**TSCD11**	-1135.67912	-1135.40695	-1136.52279
**TSCD12**	-1177.12925	-1176.80082	-1178.00245
**TSCD13**	-1173.73831	-1173.46203	-1174.62071
**TSCD14**	-1264.81971	-1264.55176	-1265.81520
**TSCD15**	-1357.91611	-1357.67288	-1359.03858
**TSCD16**	-1210.82997	-1210.55473	-1211.76511
**TSCD17**	-1289.34587	-1289.01691	-1290.36114
**TSCD18**	-1194.37920	-1194.04200	-1195.27378
**TSCD19**	-1449.08229	-1448.81938	-1450.28663
**TSCD20**	-1938.21753	-1937.94578	-1940.02187
**TSCD21**	-1463.06895	-1462.81774	-1464.30958
**TSCD22**	-1271.74013	-1271.36488	-1272.71379
**TSCD23**	-1293.50634	-1293.17866	-1294.52360
**TSCD24**	-1733.94758	-1733.67703	-1735.51121

Table S13. Beckmann rearrangements. Low level of theory potential and free energies reported at ωB97X-D/6-31+G(d,p). HLT energies reported at ωB97X-D3/def2-TZVPPD.

Code	Filename	E / Ha	G / Ha	E_{HLT} / Ha
	H2Obeckm	-76.40953	-76.40542	-76.44478
**E0-0**	SM0-0beckm	-170.05378	-170.02229	-170.12300
**E1-0**	SM1-0beckm	-209.38056	-209.32303	-209.46271
**E2-0**	SM2-0beckm	-248.68905	-248.60502	-248.78361
**E3-0**	SM3-0beckm	-286.75216	-286.66483	-286.86112
**E4-0**	SM4-0beckm	-347.88729	-347.81220	-348.02724
**E5-0**	SM5-0beckm	-546.33574	-546.27907	-546.56689
**E6-0**	SM6-0beckm	-323.86038	-323.77470	-323.98895
**E7-0**	SM7-0beckm	-366.61497	-366.45121	-366.74621
**E0-1**	SM0-1beckm	-209.37974	-209.32314	-209.46161
**E0-2**	SM0-2beckm	-248.68779	-248.60387	-248.78216
**E0-3**	SM0-3beckm	-286.75840	-286.66878	-286.86759
**E0-4**	SM0-4beckm	-347.88632	-347.81141	-348.02602
**E0-5**	SM0-5beckm	-546.33895	-546.27967	-546.57031

**E0-6**	SM0-6beckm	-323.85951	-323.77358	-323.98811
**E0-8**	SM0-8beckm	-401.06389	-400.95822	-401.21235
**E0-9**	SM0-9beckm	-417.07892	-416.98603	-417.23595
**E0-10**	SM0-10beckm	-515.56730	-515.43186	-515.76254
**E1-1**	SM1-1beckm	-248.70269	-248.62045	-248.79713
**E1-2**	SM1-2beckm	-288.01011	-287.90006	-288.11664
**E1-3**	SM1-3beckm	-326.08112	-325.96550	-326.20299
**E1-4**	SM1-4beckm	-387.20944	-387.10756	-387.36128
**E1-5**	SM1-5beckm	-585.66393	-585.57832	-585.90754
**E1-6**	SM1-6beckm	-363.18469	-363.07199	-363.32569
**E1-8**	SM1-8beckm	-440.38462	-440.25267	-440.54707
**E1-9**	SM1-9beckm	-456.40177	-456.28186	-456.57268
**E1-10**	SM1-10beckm	-554.88508	-554.72308	-555.09412
**E2-1**	SM2-1beckm	-288.01123	-287.90152	-288.11777
**E2-2**	SM2-2beckm	-327.31914	-327.18183	-327.43760
**E2-3**	SM2-3beckm	-365.38922	-365.24625	-365.52332
**E2-4**	SM2-4beckm	-426.51845	-426.39191	-426.68230
**E2-5**	SM2-5beckm	-624.97228	-624.86032	-625.22813
**E2-6**	SM2-6beckm	-402.49302	-402.35378	-402.64596
**E2-8**	SM2-8beckm	-479.69351	-479.53414	-479.86817
**E2-9**	SM2-9beckm	-495.71104	-495.56469	-495.89389
**E2-10**	SM2-10beckm	-594.19378	-594.00545	-594.41508
**E4-1**	SM4-1beckm	-387.20984	-387.10907	-387.36174
**E4-2**	SM4-2beckm	-426.51804	-426.38933	-426.68186
**E4-3**	SM4-3beckm	-464.58891	-464.45491	-464.76812
**E4-4**	SM4-4beckm	-525.71691	-525.59719	-525.92610
**E4-5**	SM4-5beckm	-724.17067	-724.06708	-724.47189
**E4-6**	SM4-6beckm	-501.69268	-501.56200	-501.89082
**E4-8**	SM4-8beckm	-578.89407	-578.74323	-579.11354
**E4-9**	SM4-9beckm	-594.91065	-594.77344	-595.13858
**E4-10**	SM4-10beckm	-693.39506	-693.21464	-693.66109
**E5-1**	SM5-1beckm	-585.65906	-585.57534	-585.90214
**E5-2**	SM5-2beckm	-624.96703	-624.85632	-625.22224
**E5-3**	SM5-3beckm	-663.03873	-662.92266	-663.30897
**E5-4**	SM5-4beckm	-724.16570	-724.06335	-724.46613
**E5-5**	SM5-5beckm	-922.62011	-922.53476	-923.01242
**E5-6**	SM5-6beckm	-700.14062	-700.02712	-700.42993
**E5-8**	SM5-8beckm	-777.34279	-777.21016	-777.65350
**E5-9**	SM5-9beckm	-793.35911	-793.23915	-793.67840
**E5-10**	SM5-10beckm	-891.84418	-891.68137	-892.20139
**E6-1**	SM6-1beckm	-363.18490	-363.07334	-363.32554
**E6-2**	SM6-2beckm	-402.49288	-402.35355	-402.64566
**E6-3**	SM6-3beckm	-440.56359	-440.41814	-440.73159
**E6-4**	SM6-4beckm	-501.69266	-501.56206	-501.89053
**E6-5**	SM6-5beckm	-700.14759	-700.03275	-700.43728
**E6-6**	SM6-6beckm	-477.66452	-477.52410	-477.85138
**E6-8**	SM6-8beckm	-554.86519	-554.70395	-555.07408
**E6-9**	SM6-9beckm	-570.88273	-570.73367	-571.10006
**E6-10**	SM6-10beckm	-669.36594	-669.17443	-669.62133
**E0-0b**	SM0-0bbeckm	-246.51164	-246.46111	-246.61272
**E1-0b**	SM1-0bbeckm	-285.83530	-285.75839	-285.94936
**E2-0b**	SM2-0bbeckm	-325.14270	-325.03823	-325.26930
**E3-0b**	SM3-0bbeckm	-363.20560	-363.09890	-363.34665
**E4-0b**	SM4-0bbeckm	-424.34298	-424.24775	-424.51472
**E5-0b**	SM5-0bbeckm	-622.79302	-622.71642	-623.05590

**E6-0b**	SM6-0bbeckm	-400.31497	-400.20842	-400.47550
**E7-0b**	SM7-0bbeckm	-443.06730	-442.88355	-443.23057
**E0-1b**	SM0-1bbeckm	-285.83390	-285.75705	-285.94771
**E0-2b**	SM0-2bbeckm	-325.14093	-325.03702	-325.26727
**E0-3b**	SM0-3bbeckm	-363.21080	-363.10144	-363.35207
**E0-4b**	SM0-4bbeckm	-424.34173	-424.24722	-424.51323
**E0-5b**	SM0-5bbeckm	-622.79566	-622.71691	-623.05854
**E0-6b**	SM0-6bbeckm	-400.33336	-400.22546	-400.49316
**E0-8b**	SM0-8bbeckm	-477.51609	-477.39005	-477.69772
**E0-9b**	SM0-9bbeckm	-493.53424	-493.41911	-493.72374
**E0-10b**	SM0-10bbeckm	-592.01713	-591.86109	-592.24432
**E1-1b**	SM1-1bbeckm	-325.15446	-325.05195	-325.28103
**E1-2b**	SM1-2bbeckm	-364.46128	-364.33092	-364.60020
**E1-3b**	SM1-3bbeckm	-402.53152	-402.39519	-402.68547
**E1-4b**	SM1-4bbeckm	-463.66248	-463.54074	-463.84659
**E1-5b**	SM1-5bbeckm	-662.11791	-662.01221	-662.39322
**E1-6b**	SM1-6bbeckm	-439.65342	-439.51893	-439.82590
**E1-8b**	SM1-8bbeckm	-516.83231	-516.67986	-517.02631
**E1-9b**	SM1-9bbeckm	-532.85508	-532.71362	-533.05742
**E1-10b**	SM1-10bbeckm	-631.33515	-631.15292	-631.57623
**E2-1b**	SM2-1bbeckm	-364.46180	-364.33181	-364.60051
**E2-2b**	SM2-2bbeckm	-403.76820	-403.61158	-403.91945
**E2-3b**	SM2-3bbeckm	-441.83873	-441.67525	-442.00500
**E2-4b**	SM2-4bbeckm	-502.96981	-502.82139	-503.16613
**E2-5b**	SM2-5bbeckm	-701.42527	-701.29285	-701.71284
**E2-6b**	SM2-6bbeckm	-478.96060	-478.79930	-479.14528
**E2-8b**	SM2-8bbeckm	-556.14045	-555.96106	-556.34659
**E2-9b**	SM2-9bbeckm	-572.16150	-571.99380	-572.37608
**E2-10b**	SM2-10bbeckm	-670.64330	-670.43335	-670.89654
**E4-1b**	SM4-1bbeckm	-463.66239	-463.54198	-463.84630
**E4-2b**	SM4-2bbeckm	-502.96927	-502.82223	-503.16557
**E4-3b**	SM4-3bbeckm	-541.04003	-540.88654	-541.25127
**E4-4b**	SM4-4bbeckm	-602.17022	-602.03060	-602.41171
**E4-5b**	SM4-5bbeckm	-800.62541	-800.50244	-800.95817
**E4-6b**	SM4-6bbeckm	-578.16171	-578.00855	-578.39143
**E4-8b**	SM4-8bbeckm	-655.34271	-655.17193	-655.59355
**E4-9b**	SM4-9bbeckm	-671.36266	-671.20400	-671.62220
**E4-10b**	SM4-10bbeckm	-769.84596	-769.64593	-770.14385
**E5-1b**	SM5-1bbeckm	-662.11442	-662.01006	-662.38935
**E5-2b**	SM5-2bbeckm	-701.42091	-701.29246	-701.70817
**E5-3b**	SM5-3bbeckm	-739.49090	-739.35613	-739.79317
**E5-4b**	SM5-4bbeckm	-800.62173	-800.50035	-800.95415
**E5-5b**	SM5-5bbeckm	-999.07648	-998.97194	-999.50017
**E5-6b**	SM5-6bbeckm	-776.61430	-776.48028	-776.93480
**E5-8b**	SM5-8bbeckm	-853.79286	-853.63971	-854.13459
**E5-9b**	SM5-9bbeckm	-869.81294	-869.67213	-870.16327
**E5-10b**	SM5-10bbeckm	-968.29618	-968.11489	-968.68507
**E6-1b**	SM6-1bbeckm	-439.63662	-439.50427	-439.80936
**E6-2b**	SM6-2bbeckm	-478.94363	-478.78401	-479.12862
**E6-3b**	SM6-3bbeckm	-517.01370	-516.84839	-517.21384
**E6-4b**	SM6-4bbeckm	-578.14460	-577.99528	-578.37484
**E6-5b**	SM6-5bbeckm	-776.60138	-776.46801	-776.92263
**E6-6b**	SM6-6bbeckm	-554.13738	-553.97271	-554.35571
**E6-8b**	SM6-8bbeckm	-631.31270	-631.13065	-631.55285
**E6-9b**	SM6-9bbeckm	-647.33780	-647.16908	-647.58611

**E6-10b**	SM6-10bbeckm	-745.81585	-745.60421	-746.10320
**F0-0**	Prod0-0beckm	-93.67425	-93.66258	-93.71450
**F1-0**	Prod1-0beckm	-133.00547	-132.97238	-133.05715
**F2-0**	Prod2-0beckm	-172.31919	-172.25999	-172.38360
**F3-0**	Prod3-0beckm	-210.38088	-210.31800	-210.45960
**F4-0**	Prod4-0beckm	-271.51298	-271.46289	-271.62279
**F5-0**	Prod5-0beckm	-469.95410	-469.92204	-470.15551
**F6-0**	Prod6-0beckm	-247.48710	-247.42662	-247.58630
**F7-0**	Prod7-0beckm	-290.24767	-290.10854	-290.34888
**F0-1**	Prod0-1beckm	-133.02389	-132.99161	-133.07739
**F0-2**	Prod0-2beckm	-172.33433	-172.27580	-172.40052
**F0-3**	Prod0-3beckm	-210.39611	-210.33441	-210.47669
**F0-4**	Prod0-4beckm	-271.52917	-271.47971	-271.64094
**F0-5**	Prod0-5beckm	-469.96958	-469.93819	-470.17303
**F0-6**	Prod0-6beckm	-247.49629	-247.43590	-247.59699
**F0-8**	Prod0-8beckm	-324.71445	-324.63415	-324.83501
**F0-9**	Prod0-9beckm	-340.72497	-340.65655	-340.85408
**F0-10**	Prod0-10beckm	-439.22103	-439.11272	-439.38837
**F1-1**	Prod1-1beckm	-172.34815	-172.29038	-172.41319
**F1-2**	Prod1-2beckm	-211.65753	-211.57262	-211.73528
**F1-3**	Prod1-3beckm	-249.71922	-249.63078	-249.81142
**F1-4**	Prod1-4beckm	-310.85385	-310.77803	-310.97705
**F1-5**	Prod1-5beckm	-509.29658	-509.23870	-509.51118
**F1-6**	Prod1-6beckm	-286.82166	-286.73530	-286.93371
**F1-8**	Prod1-8beckm	-364.03489	-363.92865	-364.16721
**F1-9**	Prod1-9beckm	-380.04780	-379.95359	-380.18858
**F1-10**	Prod1-10beckm	-478.53928	-478.40313	-478.71845
**F2-1**	Prod2-1beckm	-211.66072	-211.57603	-211.73845
**F2-2**	Prod2-2beckm	-250.96994	-250.85792	-251.06035
**F2-3**	Prod2-3beckm	-289.03161	-288.91684	-289.13645
**F2-4**	Prod2-4beckm	-350.16670	-350.06346	-350.30245
**F2-5**	Prod2-5beckm	-548.60976	-548.52386	-548.83682
**F2-6**	Prod2-6beckm	-326.13432	-326.02146	-326.25898
**F2-8**	Prod2-8beckm	-403.34695	-403.21292	-403.49182
**F2-9**	Prod2-9beckm	-419.36028	-419.23894	-419.51363
**F2-10**	Prod2-10beckm	-517.85089	-517.68761	-518.04262
**F4-1**	Prod4-1beckm	-310.85621	-310.78067	-310.97928
**F4-2**	Prod4-2beckm	-350.16585	-350.06285	-350.30154
**F4-3**	Prod4-3beckm	-388.22768	-388.12076	-388.37774
**F4-4**	Prod4-4beckm	-449.36196	-449.26827	-449.54315
**F4-5**	Prod4-5beckm	-647.80450	-647.72763	-648.07699
**F4-6**	Prod4-6beckm	-425.33017	-425.22511	-425.50007
**F4-8**	Prod4-8beckm	-502.54407	-502.41931	-502.73404
**F4-9**	Prod4-9beckm	-518.55641	-518.44394	-518.75502
**F4-10**	Prod4-10beckm	-617.04884	-616.89512	-617.28562
**F5-1**	Prod5-1beckm	-509.29959	-509.24176	-509.51407
**F5-2**	Prod5-2beckm	-548.60965	-548.52494	-548.83664
**F5-3**	Prod5-3beckm	-586.67147	-586.58307	-586.91283
**F5-4**	Prod5-4beckm	-647.80524	-647.73021	-648.07784
**F5-5**	Prod5-5beckm	-846.24742	-846.18961	-846.61138
**F5-6**	Prod5-6beckm	-623.77309	-623.68672	-624.03449
**F5-8**	Prod5-8beckm	-700.98878	-700.88296	-701.27006
**F5-9**	Prod5-9beckm	-717.00032	-716.90731	-717.29027
**F5-10**	Prod5-10beckm	-815.49444	-815.35884	-815.82249
**F6-1**	Prod6-1beckm	-286.83044	-286.74387	-286.94283

**F6-2**	Prod6-2beckm	-326.13996	-326.02564	-326.26487
**F6-3**	Prod6-3beckm	-364.20166	-364.08441	-364.34105
**F6-4**	Prod6-4beckm	-425.33624	-425.23071	-425.50660
**F6-5**	Prod6-5beckm	-623.77894	-623.69188	-624.04073
**F6-6**	Prod6-6beckm	-401.30386	-401.18928	-401.46319
**F6-8**	Prod6-8beckm	-478.51789	-478.38327	-478.69723
**F6-9**	Prod6-9beckm	-494.53046	-494.40833	-494.71839
**F6-10**	Prod6-10beckm	-593.02284	-592.85736	-593.24882
**TSEF0-0**	TS0-0beckm	-170.02092	-169.99872	-170.09161
**TSEF1-0**	TS1-0beckm	-209.35220	-209.30279	-209.43530
**TSEF2-0**	TS2-0beckm	-248.66997	-248.59446	-248.76537
**TSEF3-0**	TS3-0beckm	-286.73708	-286.65806	-286.84580
**TSEF4-0**	TS4-0beckm	-347.86183	-347.79520	-348.00279
**TSEF5-0**	TS5-0beckm	-546.30050	-546.25230	-546.53329
**TSEF6-0**	TS6-0beckm	-323.85835	-323.77604	-323.98494
**TSEF7-0**	TS7-0beckm	-366.59998	-366.44446	-366.73196
**TSEF0-1**	TS0-1beckm	-209.36353	-209.31531	-209.44655
**TSEF0-2**	TS0-2beckm	-248.67361	-248.59773	-248.76917
**TSEF0-3**	TS0-3beckm	-286.73621	-286.65662	-286.84610
**TSEF0-4**	TS0-4beckm	-347.86923	-347.80236	-348.01041
**TSEF0-5**	TS0-5beckm	-546.31317	-546.26515	-546.54571
**TSEF0-6**	TS0-6beckm	-323.84101	-323.76305	-323.97093
**TSEF0-8**	TS0-8beckm	-401.05246	-400.95374	-401.20235
**TSEF0-9**	TS0-9beckm	-417.06499	-416.97876	-417.22347
**TSEF0-10**	TS0-10beckm	-515.55729	-515.42850	-515.75388
**TSEF1-1**	TS1-1beckm	-248.68983	-248.61338	-248.78516
**TSEF1-2**	TS1-2beckm	-287.99968	-287.89650	-288.10716
**TSEF1-3**	TS1-3beckm	-326.06383	-325.95742	-326.18574
**TSEF1-4**	TS1-4beckm	-387.19560	-387.10149	-387.34868
**TSEF1-5**	TS1-5beckm	-585.64282	-585.56601	-585.88700
**TSEF1-6**	TS1-6beckm	-363.17170	-363.06671	-363.31354
**TSEF1-8**	TS1-8beckm	-440.37655	-440.24945	-440.53775
**TSEF1-9**	TS1-9beckm	-456.39112	-456.27694	-456.56121
**TSEF1-10**	TS1-10beckm	-554.88013	-554.72299	-555.08778
**TSEF2-1**	TS2-1beckm	-288.00511	-287.90122	-288.11204
**TSEF2-2**	TS2-2beckm	-327.31509	-327.18330	-327.43391
**TSEF2-3**	TS2-3beckm	-365.37929	-365.24503	-365.51260
**TSEF2-4**	TS2-4beckm	-426.51179	-426.38962	-426.67626
**TSEF2-5**	TS2-5beckm	-624.95958	-624.85522	-625.21520
**TSEF2-6**	TS2-6beckm	-402.48829	-402.35559	-402.64162
**TSEF2-8**	TS2-8beckm	-479.68903	-479.53335	-479.86183
**TSEF2-9**	TS2-9beckm	-495.70525	-495.56221	-495.88659
**TSEF2-10**	TS2-10beckm	-594.19137	-594.00436	-594.41059
**TSEF4-1**	TS4-1beckm	-387.19893	-387.10494	-387.35179
**TSEF4-2**	TS4-2beckm	-426.50959	-426.38766	-426.67434
**TSEF4-3**	TS4-3beckm	-464.57399	-464.44897	-464.75314
**TSEF4-4**	TS4-4beckm	-525.70530	-525.59282	-525.91575
**TSEF4-5**	TS4-5beckm	-724.15256	-724.05872	-724.45423
**TSEF4-6**	TS4-6beckm	-501.68273	-501.55920	-501.88172
**TSEF4-8**	TS4-8beckm	-578.88647	-578.74090	-579.10463
**TSEF4-9**	TS4-9beckm	-594.90085	-594.76869	-595.12807
**TSEF4-10**	TS4-10beckm	-693.39005	-693.21432	-693.65475
**TSEF0-0b**	TS0-0bbeckm	-246.45543	-246.41398	-246.55885
**TSEF1-0b**	TS1-0bbeckm	-285.78319	-285.71433	-285.89885
**TSEF2-0b**	TS2-0bbeckm	-325.10021	-325.00469	-325.22837

**TSEF3-0b**	TS3-0bbeckm	-363.16665	-363.06911	-363.30855
**TSEF4-0b**	TS4-0bbeckm	-424.29296	-424.20669	-424.46646
**TSEF5-0b**	TS5-0bbeckm	-622.73216	-622.66431	-622.99738
**TSEF6-0b**	TS6-0bbeckm	-400.29172	-400.19281	-400.45192
**TSEF7-0b**	TS7-0bbeckm	-443.02948	-442.85470	-443.19426
**TSEF0-1b**	TS0-1bbeckm	-285.79823	-285.73037	-285.91418
**TSEF0-2b**	TS0-2bbeckm	-325.10795	-325.01291	-325.23642
**TSEF0-3b**	TS0-3bbeckm	-363.17031	-363.07225	-363.31315
**TSEF0-4b**	TS0-4bbeckm	-424.30504	-424.21654	-424.47817
**TSEF0-5b**	TS0-5bbeckm	-622.74829	-622.68078	-623.01362
**TSEF0-6b**	TS0-6bbeckm	-400.27488	-400.17920	-400.43763
**TSEF0-8b**	TS0-8bbeckm	-477.48566	-477.36919	-477.66858
**TSEF0-9b**	TS0-9bbeckm	-493.49915	-493.39478	-493.69057
**TSEF0-10b**	TS0-10bbeckm	-591.98977	-591.84314	-592.21944
**TSEF1-1b**	TS1-1bbeckm	-325.12028	-325.02562	-325.24881
**TSEF1-2b**	TS1-2bbeckm	-364.42986	-364.30785	-364.57058
**TSEF1-3b**	TS1-3bbeckm	-402.49389	-402.36945	-402.64903
**TSEF1-4b**	TS1-4bbeckm	-463.62920	-463.51287	-463.81418
**TSEF1-5b**	TS1-5bbeckm	-662.07393	-661.97838	-662.35112
**TSEF1-6b**	TS1-6bbeckm	-439.60197	-439.47743	-439.77694
**TSEF1-8b**	TS1-8bbeckm	-516.80571	-516.66259	-517.00113
**TSEF1-9b**	TS1-9bbeckm	-532.82072	-532.68900	-533.02463
**TSEF1-10b**	TS1-10bbeckm	-631.30908	-631.13534	-631.55126
**TSEF2-1b**	TS2-1bbeckm	-364.43565	-364.31313	-364.57618
**TSEF2-2b**	TS2-2bbeckm	-403.74535	-403.59729	-403.89794
**TSEF2-3b**	TS2-3bbeckm	-441.80966	-441.65678	-441.97666
**TSEF2-4b**	TS2-4bbeckm	-502.94498	-502.80230	-503.14190
**TSEF2-5b**	TS2-5bbeckm	-701.39077	-701.26780	-701.67982
**TSEF2-6b**	TS2-6bbeckm	-478.91863	-478.76826	-479.10529
**TSEF2-8b**	TS2-8bbeckm	-556.12006	-555.94793	-556.32700
**TSEF2-9b**	TS2-9bbeckm	-572.13482	-571.97440	-572.35060
**TSEF2-10b**	TS2-10bbeckm	-670.62265	-670.41972	-670.87607
**TSEF4-1b**	TS4-1bbeckm	-463.62968	-463.51711	-463.81568
**TSEF4-2b**	TS4-2bbeckm	-502.93998	-502.79995	-503.13800
**TSEF4-3b**	TS4-3bbeckm	-541.00445	-540.86274	-541.21680
**TSEF4-4b**	TS4-4bbeckm	-602.13899	-602.00486	-602.38139
**TSEF4-5b**	TS4-5bbeckm	-800.58386	-800.47055	-800.91844
**TSEF4-6b**	TS4-6bbeckm	-578.11339	-577.97084	-578.34534
**TSEF4-8b**	TS4-8bbeckm	-655.31636	-655.15313	-655.56855
**TSEF4-9b**	TS4-9bbeckm	-671.32968	-671.17967	-671.59087
**TSEF4-10b**	TS4-10bbeckm	-769.81985	-769.62722	-770.11887
**TSEF5-1b**	TS5-1bbeckm	-662.07118	-661.97622	-662.34842
**TSEF5-2b**	TS5-2bbeckm	-701.38176	-701.26050	-701.67119
**TSEF5-3b**	TS5-3bbeckm	-739.44695	-739.32180	-739.75061
**TSEF5-4b**	TS5-4bbeckm	-800.57989	-800.46512	-800.91402
**TSEF5-5b**	TS5-5bbeckm	-999.02495	-998.92994	-999.45076
**TSEF5-6b**	TS5-6bbeckm	-776.55501	-776.43032	-776.87806
**TSEF5-8b**	TS5-8bbeckm	-853.75669	-853.61150	-854.10034
**TSEF5-9b**	TS5-9bbeckm	-869.76940	-869.63640	-870.12183
**TSEF5-10b**	TS5-10bbeckm	-968.26073	-968.08636	-968.65097
**TSEF6-1b**	TS6-1bbeckm	-439.62264	-439.49665	-439.79539
**TSEF6-2b**	TS6-2bbeckm	-478.93128	-478.77692	-479.11634
**TSEF6-3b**	TS6-3bbeckm	-516.99672	-516.83873	-517.19575
**TSEF6-4b**	TS6-4bbeckm	-578.13076	-577.98499	-578.36002
**TSEF6-5b**	TS6-5bbeckm	-776.57928	-776.45225	-776.90036

**TSEF6-6b**	TS6-6bbeckm	-554.10615	-553.94945	-554.32411
**TSEF6-8b**	TS6-8bbeckm	-631.30416	-631.12623	-631.54306
**TSEF6-9b**	TS6-9bbeckm	-647.31938	-647.15364	-647.56744
**TSEF6-10b**	TS6-10bbeckm	-745.80541	-745.59782	-746.09096

3. REFERENCES

(1) Gaussian 09, Revision B.1, Frisch, M. J., Trucks, G. W., Schlegel, H. B., Scuseria, G. E., Robb, M. A., Cheeseman, J. R., Scalmani, G., Barone, V., Mennucci, B., Petersson, G. A., Nakatsuji, H., Caricato, M., Li, X., Hratchian, H. P., Izmaylov, A. F., Bloino, J., Zheng, G., Sonnenberg, J. L., Hada, M., Ehara, M., Toyota, K., Fukuda, R., Hasegawa, J., Ishida, M., Nakajima, T., Honda, Y., Kitao, O., Nakai, H., Vreven, T., Montgomery, J. A., Peralta, Jr. J. E., Ogliaro, F., Bearpark, M., Heyd, J. J., Brothers, E., Kudin, K. N., Staroverov, V. N., Kobayashi, R., Normand, J., Raghavachari, K., Rendell, A., Burant, J. C., Iyengar, S. S., Tomasi, J., Cossi, M., Rega, N., Millam, J. M., Klene, M., Knox, J. E., Cross, J. B., Bakken, V., Adamo, C., Jaramillo, J., Gomperts, R., Stratmann, R. E., Yazyev, O., Austin, A. J., Cammi, R., Pomelli, C., Ochterski, J. W., Martin, R. L., Morokuma, K., Zakrzewski, V. G., Voth, G. A., Salvador, P., Dannenberg, J. J., Dapprich, S., Daniels, A. D., Farkas, Ö., Foresman, J. B., Ortiz, J. V., Cioslowski, J., Fox, D. J. Gaussian, Inc., Wallingford CT 2009.

(2) Neese, F. Software update: the ORCA program system, version 5.0. *WIREs Comput. Molec. Sci.*, **2022**, 12(1)e1606.

(3) Hehre, W. J.; Ditchfield, R.; Pople, J. A. Self–Consistent Molecular Orbital Methods. XII. Further Extensions of Gaussian–Type Basis Sets for Use in Molecular Orbital Studies of Organic Molecules. *J. Chem. Phys.* **1972**, *56*, 2257–2261.

(4) (a) Weigend, F.; Ahlrichs, R. Balanced basis sets of split valence, triple zeta valence and quadruple zeta valence quality for H to Rn: Design and assessment of accuracy. *Phys. Chem. Chem. Phys.* **2005**, *7*, 3297–3305; (b) D. Andrae, D.; Haeussermann, U.; Dolg, M.; Stoll, H.; Preuss, H. Energy-adjusted ab initio pseudopotentials for the second and third row transition elements. *Theor. Chim. Acta*, **1990**, *77*, 123-141.

(5) Stoychev, G. L.; Auer, A.; Neese, F. Automatic Generation of Auxiliary Basis Sets. *J. Chem. Theory Comput.* **2017**, *13*, 554–562.

(6) Marenich, A. V.; Cramer, C. J.; Truhlar, D.G. Universal Solvation Model Based on Solute Electron Density and on a Continuum Model of the Solvent Defined by the Bulk Dielectric Constant and Atomic Surface Tensions. *J. Phys. Chem. B*, **2009**, *113*, 6378–6396.

(7) Lin, Y.-S.; Li, G.-D.; Mao, S.-P.; Chai, J.-D. Long-Range Corrected Hybrid Density Functionals with Improved Dispersion Corrections. *J. Chem. Theory Comput.* **2013**, *9*, 263–272.

(8) Neese, F. An improvement of the resolution of the identity approximation for the formation of the Coulomb matrix. *J. Comp. Chem.*, **2003**, *24*, 1740–1747.

(9) Álvarez-Moreno, M.; de Graaf, C.; Lopez, N.; Maseras, F.; Poblet, J. M.; Bo, C. Managing the Computational Chemistry Big Data Problem: The ioChem-BD Platform. *J. Chem. Inf. Model.* **2015**, *55*, 95−103.

4. Coordinates of Stationary Points

prod10dhs

C	-0.30861100	-4.04157800	-0.55765500
C	-0.25103800	-3.51657900	0.75043800
C	0.16981900	-2.20873300	0.89074500
C	0.52599700	-1.43065700	-0.25780500
C	0.45456700	-2.00162000	-1.56644200
C	0.03518900	-3.30657900	-1.69963500
H	0.23741900	-1.74601300	1.87058600
H	0.72881200	-1.39948100	-2.42596400
H	-0.03112700	-3.76791600	-2.67809600
C	0.91503800	-0.13634600	-0.10452300
H	2.37023900	1.21506400	0.18253700
C	0.45894800	2.31012700	0.04838700
C	-0.92935000	2.24588100	-0.13213300
C	1.08499600	3.54601200	0.23772400
C	-1.67960000	3.41271400	-0.12234600
H	-1.42232000	1.28887200	-0.28074800
C	0.32558100	4.71191400	0.24639400
H	2.16043600	3.59915500	0.37841800
C	-1.05333100	4.64610600	0.06679000
H	-2.75380900	3.36335800	-0.26322400
H	0.81228200	5.66973800	0.39312700
H	-1.64331900	5.55638300	0.07327300
C	1.29146200	1.09359400	0.04407300
C	-0.64412000	-4.36571100	1.92863000
H	-1.70334900	-4.63318800	1.87123100
H	-0.06785100	-5.29483300	1.94679600
H	-0.47720500	-3.84325900	2.87203500
H	-0.63698800	-5.07045800	-0.68454800

*

prod11dhs

C	-0.21562800	-4.09952300	-0.58203900
C	-0.31604100	-3.54798400	0.70856100
C	0.05611600	-2.22818000	0.90477600
C	0.52300700	-1.46316400	-0.20355600
C	0.61370600	-2.03884100	-1.50786700
C	0.24186500	-3.35579400	-1.67545200
H	-0.01131500	-1.78285800	1.89109000
H	0.97123500	-1.43639200	-2.33500200
H	0.30200600	-3.82551700	-2.65021000
C	0.88323100	-0.16089400	-0.01551000
H	2.30775600	1.17704400	0.40954600
C	0.42967900	2.29257600	0.09938800
C	-0.94697600	2.23994300	-0.15817300
C	1.06066400	3.52442700	0.29913500
C	-1.68100400	3.41563800	-0.21480800
H	-1.44369600	1.28546900	-0.31022600
C	0.31736000	4.69893500	0.23992800
H	2.12712000	3.56755800	0.49911400
C	-1.05000500	4.64520400	-0.01632600
H	-2.74674800	3.37618600	-0.41173100
H	0.80711200	5.65391200	0.39466000
H	-1.62756300	5.56230700	-0.06086400
C	1.24490300	1.06731500	0.16933900
H	-0.50142100	-5.13711000	-0.73766100
O	-0.75390400	-4.23286500	1.77627200
H	-0.99131600	-5.14164100	1.56306400

*

prod12dhs

C	0.09742400	-3.28616100	0.39035100
C	0.43024100	-2.51208800	1.53434900
C	0.72022500	-1.18102500	1.42395000
C	0.69302900	-0.54377600	0.13646000
C	0.34699900	-1.32868800	-1.01733600
C	0.05691700	-2.65707600	-0.88413500
H	0.45558700	-2.99433900	2.50543300
H	0.97727200	-0.58994500	2.29677800
H	0.32266900	-0.84626900	-1.98876300
H	-0.20135700	-3.23338100	-1.76577400
C	0.99168700	0.76735000	0.01118200
H	2.36776000	2.20563000	-0.27744700
C	0.41070400	3.19306000	-0.05789500
C	-0.97139500	3.05651800	0.12733600
C	0.97346800	4.46515400	-0.19999100
C	-1.77734400	4.18553200	0.16800500
H	-1.41710500	2.07165400	0.23870800
C	0.15978000	5.59323300	-0.15790600
H	2.04417500	4.57545800	-0.34394400
C	-1.21309100	5.45442200	0.02516600
H	-2.84710200	4.07847700	0.31094800
H	0.60018300	6.57795800	-0.26945000
H	-1.84696600	6.33425200	0.05628600
C	1.30269600	2.02094500	-0.11154400
C	-0.18574600	-4.70333900	0.56446100
C	-1.22756900	-5.41364200	-0.31123400
C	0.20128600	-5.71771500	-0.52146900
H	-0.11176200	-5.06044400	1.58580000
H	-1.75253100	-4.80882800	-1.04333500
H	-1.84780900	-6.13482700	0.20890200
H	0.59971300	-6.65556300	-0.15122100
H	0.69929700	-5.33007500	-1.40410500

*

prod13dhs

C	-0.28576800	-3.74372500	-0.87633700
C	-0.03120600	-3.25865900	0.42171800
C	0.35206100	-1.94699300	0.59344700
C	0.47930400	-1.09579900	-0.54940000
C	0.20817400	-1.61138400	-1.85654800
C	-0.16965100	-2.92992900	-2.00749800
H	0.55743200	-1.54242600	1.58109300
H	0.30740000	-0.95117600	-2.71192600
H	-0.37298500	-3.33547600	-2.99165700
C	0.85703000	0.20393100	-0.39219900
H	2.32691400	1.55659800	-0.30072500
C	0.42665600	2.63338200	0.00362600
C	-0.96856500	2.55995200	0.11807400
C	1.07852700	3.86479800	0.12573800
C	-1.70011600	3.71430800	0.35456100
H	-1.48078500	1.60617900	0.02358500
C	0.33715600	5.01788500	0.36248400
H	2.15904400	3.92427400	0.03563200
C	-1.04831700	4.94306700	0.47753400
H	-2.77930500	3.65901400	0.44593600
H	0.84237000	5.97247100	0.45840600
H	-1.62364100	5.84354100	0.66372700
C	1.24051700	1.43069100	-0.23891500
H	-0.58030700	-4.78576600	-0.97575100

C	-0.17856700	-4.18290300	1.59501900
O	-0.49924300	-5.33615400	1.46276700
H	0.02283400	-3.73924200	2.58801200

*

prod14dhs

C	0.00364000	-3.28720000	-0.06837800
C	0.59240900	-2.66283500	1.03068700
C	0.88852500	-1.31907800	0.92944700
C	0.58366200	-0.61808100	-0.27606600
C	-0.02105200	-1.30058400	-1.37432600
C	-0.30913000	-2.64549500	-1.26614100
H	0.80312400	-3.23385900	1.92684300
H	1.34863800	-0.78678400	1.75485800
H	-0.24569300	-0.75428000	-2.28396400
H	-0.76460000	-3.20420600	-2.07479300
C	0.86286000	0.71835400	-0.37547600
H	2.15798600	2.14381600	-0.88202900
C	0.34794300	3.15615000	-0.13071300
C	-0.94708800	3.03023900	0.39158200
C	0.90839900	4.42208600	-0.33127100
C	-1.67106500	4.16935100	0.71030800
H	-1.38856500	2.04968600	0.54812300
C	0.17480600	5.55891500	-0.00845800
H	1.91037100	4.52079600	-0.73783600
C	-1.11055000	5.43298600	0.51151100
H	-2.67217200	4.07541000	1.11641400
H	0.60913500	6.54033800	-0.16240800
H	-1.68018400	6.32106300	0.76342900
C	1.15347300	1.97390600	-0.47636500
N	-0.31233400	-4.74163800	0.04474200
O	-0.79119000	-5.27184400	-0.93850200
O	-0.06431300	-5.27053400	1.11055900

*

prod15dhs

C	-0.09861300	-3.49805200	-0.12637300
C	-0.27779300	-2.85237300	1.11115700
C	0.04706400	-1.52879000	1.25525400
C	0.56796400	-0.82230100	0.12657200
C	0.75053100	-1.47901600	-1.13025800
C	0.41484600	-2.80343800	-1.23719700
H	-0.09067000	-1.03158000	2.20869900
H	1.14657800	-0.94396900	-1.98584700
C	0.88666800	0.49727700	0.24487300
H	2.25756300	1.89352600	0.65192600
C	0.39531000	2.94529000	0.12076600
C	-0.95704100	2.85092000	-0.23727500
C	0.99955000	4.19853700	0.26659500
C	-1.69329000	4.00717200	-0.44786700
H	-1.43322100	1.88057200	-0.34975400
C	0.25392300	5.35301000	0.05238600
H	2.04694500	4.27351900	0.54309900
C	-1.08872100	5.25776600	-0.30356800
H	-2.73999000	3.93689500	-0.72264500
H	0.72231800	6.32434700	0.16509000
H	-1.66798800	6.15963400	-0.46970200
C	1.21469000	1.74488200	0.35026700
F	-0.41309200	-4.75496600	-0.24512900
F	-0.76392800	-3.56058200	2.11364400
F	0.55886500	-3.46653800	-2.36961800

*

prod16dhs

C	0.19038900	-3.70653100	0.40098400
C	1.22760900	-2.97212800	1.01004400
C	1.39563300	-1.65670700	0.67874900
C	0.52425300	-1.03040400	-0.27818800
C	-0.53340200	-1.79644800	-0.89035100
C	-0.69322300	-3.10631000	-0.55469600
H	1.86618600	-3.47701200	1.72511500
H	2.18624000	-1.06647600	1.12878800
H	-1.18545900	-1.31235000	-1.61036400
C	0.70218500	0.26704500	-0.59909100
H	1.55883800	1.66542400	-1.77031700
C	0.29321000	2.70807600	-0.30017500
C	-0.50048200	2.62223000	0.85145100
C	0.53729600	3.95358700	-0.88734200
C	-1.04412500	3.77363200	1.40303100
H	-0.68635400	1.65947600	1.32002200
C	-0.01297000	5.10397800	-0.33097300
H	1.15359800	4.02440000	-1.77877400
C	-0.80289200	5.01520400	0.81182900
H	-1.65488300	3.70701400	2.29690500
H	0.17633000	6.06775500	-0.79062100
H	-1.23001700	5.91254000	1.24649200
C	0.88361300	1.51073000	-0.92435700
O	0.03805300	-4.96606600	0.72256000
H	-0.70518000	-5.36391300	0.23918200
O	-1.62030400	-3.96935300	-1.02373300
H	-2.21387500	-3.57743700	-1.67294000

*

prod17dhs

C	0.00388400	-3.13622100	-0.48111100
C	-0.06964700	-2.52212400	0.82644100
C	0.25326600	-1.20035800	0.95546600
C	0.65620100	-0.43342100	-0.19859200
C	0.71369300	-1.06566300	-1.48424800
C	0.39346000	-2.38873600	-1.61830000
H	0.21462900	-0.70097600	1.91547200
H	1.01582300	-0.47881300	-2.34486300
H	0.43980700	-2.86375800	-2.58955600
C	0.98499700	0.86796000	-0.08963100
H	2.37818500	2.29769100	0.20982300
C	0.44090400	3.29970200	-0.09335300
C	-0.93476600	3.17528800	-0.32787900
C	1.01100500	4.56882600	0.04510500
C	-1.72632400	4.31162600	-0.42064000
H	-1.38656300	2.19298000	-0.43741100
C	0.21239300	5.70472800	-0.04914500
H	2.07701900	4.67073500	0.22591200
C	-1.15413700	5.57727300	-0.28109900
H	-2.79127200	4.21276300	-0.60107900
H	0.65950800	6.68673600	0.05980500
H	-1.77611800	6.46310200	-0.35332300
C	1.31751800	2.11903200	0.01588400
O	-0.31399600	-4.39894300	-0.50700500
C	-0.28769200	-5.14884800	-1.73212300
H	-1.00981600	-4.73737200	-2.44139700
H	0.72063400	-5.15160300	-2.15252900
H	-0.57620900	-6.15827100	-1.45001000
O	-0.45730000	-3.34823600	1.79818500
C	-0.57021700	-2.82823600	3.11925100

H	0.39847800	-2.46394500	3.47795900
H	-1.31648000	-2.02749000	3.15642800
H	-0.89763300	-3.66314000	3.73490800

*

prod18dhs

C	0.02432100	-3.22841600	0.01821800
C	0.95916700	-2.49914100	0.84072500
C	1.20652900	-1.18655200	0.60998100
C	0.54294900	-0.47392800	-0.45887600
C	-0.38570500	-1.21423800	-1.28308900
C	-0.63185300	-2.52828700	-1.05959900
H	1.47574100	-2.99957500	1.64847800
H	1.91075200	-0.64476900	1.23249300
H	-0.88696000	-0.69435500	-2.09275700
H	-1.33299200	-3.04897800	-1.69736900
C	0.77737000	0.82757800	-0.67110700
H	1.82436900	2.31494400	-1.56673100
C	0.31094600	3.23603500	-0.25995100
C	-0.72261900	3.05589300	0.66853800
C	0.70029000	4.52963600	-0.62034200
C	-1.35470500	4.15850000	1.22633800
H	-1.03098800	2.05407600	0.95556000
C	0.06314500	5.63209700	-0.05857900
H	1.50033800	4.67539600	-1.34034300
C	-0.96250700	5.44836000	0.86433900
H	-2.15286100	4.01562700	1.94699500
H	0.36976200	6.63340100	-0.34117000
H	-1.45682400	6.30823000	1.30366900
C	1.01322500	2.09117300	-0.87066500
N	-0.22777200	-4.51386500	0.24787200
C	0.41619700	-5.22358100	1.35832300
C	-1.15284200	-5.26593400	-0.60710800
H	0.00650400	-6.22888200	1.41891800
H	1.49551400	-5.29856200	1.19916800
H	0.21716600	-4.71962700	2.30709000
H	-1.18410600	-6.29932100	-0.27074900
H	-2.16264100	-4.85170800	-0.54277100
H	-0.81500100	-5.25524500	-1.64651600

*

prod1dhs

C	-0.24653600	-4.02291200	0.12291000
C	0.28086400	-3.32953500	1.23586900
C	0.65311300	-2.01380300	1.12745700
C	0.50373500	-1.33255000	-0.12530000
C	-0.03267000	-2.04167300	-1.25021700
C	-0.39779100	-3.35682200	-1.11466200
H	0.38977800	-3.84841300	2.18230700
H	1.05931900	-1.47311300	1.97577200
H	-0.14443200	-1.52222700	-2.19612100
H	-0.80754700	-3.89660200	-1.96176600
C	0.85961300	-0.02938400	-0.23960300
H	2.26357200	1.35117800	-0.63364300
C	0.40743300	2.42056500	-0.11204700
C	-0.94879100	2.33925800	0.23160900
C	1.02224900	3.66948300	-0.24455900
C	-1.67742300	3.50133600	0.44055800
H	-1.43422100	1.37244200	0.33440800
C	0.28550700	4.83051600	-0.03255100
H	2.07320800	3.73605600	-0.50979800
C	-1.06154400	4.74731000	0.30882600

H	-2.72762100	3.43841900	0.70402100
H	0.76442400	5.79787300	-0.13600800
H	-1.63422200	5.65378200	0.47295000
C	1.21852700	1.21169800	-0.34132000
C	-0.61401400	-5.46507900	0.24518500
H	-1.41756100	-5.73761800	-0.44179800
H	0.26057000	-6.07558300	-0.01466500
H	-0.90294300	-5.72378900	1.26559600

*

prod2dhs

C	-0.07031700	-3.58842000	-0.11415700
C	0.29776400	-2.95481100	1.08950000
C	0.63466300	-1.62510700	1.06531500
C	0.60814600	-0.89902600	-0.17013400
C	0.22923000	-1.57337200	-1.37671800
C	-0.10565900	-2.90346900	-1.34522900
H	0.31092200	-3.52048700	2.01370600
H	0.92234900	-1.10907200	1.97511800
H	0.21144600	-1.01898200	-2.30901600
H	-0.39415100	-3.43125800	-2.24661500
C	0.93246800	0.41927500	-0.19492600
H	2.32690400	1.84564100	-0.39601800
C	0.39808600	2.85357900	-0.04565600
C	-0.97523000	2.72810900	0.20471700
C	0.98146900	4.12141700	-0.13642200
C	-1.75296500	3.86624400	0.36136300
H	-1.43557000	1.74655700	0.27961200
C	0.19477800	5.25808000	0.02127000
H	2.04545500	4.22188800	-0.32873300
C	-1.16916900	5.13126800	0.26913200
H	-2.81566100	3.76972900	0.55490700
H	0.64887600	6.24009600	-0.05073900
H	-1.78081100	6.01857200	0.39210600
C	1.26057900	1.67227200	-0.21951100
Cl	-0.48877200	-5.24144100	-0.08003000

*

prod3dhs

C	-0.31167700	-3.02567600	-1.50970600
C	-0.06908200	-2.68740900	-0.16712000
C	0.33277000	-1.41296700	0.15789200
C	0.49157200	-0.44761700	-0.88404600
C	0.23572800	-0.80759400	-2.24292000
C	-0.16164400	-2.09418900	-2.54186500
H	0.52686800	-1.12968300	1.18672200
H	0.35894300	-0.05976500	-3.01916300
H	-0.35821800	-2.38614600	-3.56665400
C	0.87702700	0.82506500	-0.57903000
H	2.35124800	2.15225600	-0.33323100
C	0.44921400	3.20961300	0.03045800
C	-0.94328500	3.12076000	0.16555500
C	1.09765700	4.43552100	0.21196800
C	-1.67633200	4.25554600	0.47921500
H	-1.45160200	2.16955400	0.03224500
C	0.35434700	5.56908100	0.52463700
H	2.17614400	4.50634400	0.10753800
C	-1.02862100	5.47963500	0.65816500
H	-2.75313200	4.18810900	0.58840200
H	0.85621200	6.51992800	0.66500100
H	-1.60557500	6.36461600	0.90431700
C	1.26294900	2.02891800	-0.30375700

C	-0.24539500	-3.75183900	0.89856200
H	-0.62544300	-4.03811200	-1.74865500
F	-0.09581900	-3.24268400	2.12621800
F	-1.46432700	-4.30150600	0.81504100
F	0.65703200	-4.72889900	0.73549300

*

prod4dhs

C	0.07682800	-2.88889800	-0.07055600
C	0.33429500	-2.25448200	1.14985400
C	0.64118900	-0.90948900	1.15587300
C	0.68804900	-0.19108100	-0.07646400
C	0.42121900	-0.86401500	-1.30902200
C	0.11850900	-2.20636700	-1.29617300
H	0.29296200	-2.81642000	2.07509000
H	0.84628900	-0.38715000	2.08416400
H	0.46076800	-0.30669500	-2.23868900
H	-0.08589100	-2.73501100	-2.22048400
C	0.97773900	1.14350400	-0.07759400
H	2.34856000	2.58881900	-0.20041200
C	0.38349700	3.57133800	-0.00093200
C	-0.99695200	3.42474500	0.19389700
C	0.94740700	4.84621800	-0.11525300
C	-1.80255000	4.55135000	0.27038900
H	-1.44091700	2.43752200	0.29009600
C	0.13170800	5.97051800	-0.03911800
H	2.01657200	4.96170100	-0.26515200
C	-1.23932900	5.82373000	0.15279000
H	-2.87039300	4.44031100	0.42293700
H	0.56909400	6.95850200	-0.13007000
H	-1.87370200	6.70159700	0.21243200
C	1.27381200	2.40244800	-0.09487400
C	-0.27063100	-4.37275000	-0.10231000
F	-1.49751200	-4.53755400	-0.61207700
F	-0.24152500	-4.91775400	1.11456200
F	0.59873100	-5.02684700	-0.88138800

*

prod5dhs

C	-0.24734300	-4.01998900	0.08293700
C	0.29368600	-3.34715100	1.20779000
C	0.66227100	-2.04018700	1.08857700
C	0.50300800	-1.34779400	-0.16476700
C	-0.04884500	-2.05775000	-1.28771600
C	-0.41691200	-3.36670400	-1.16521500
H	0.40074300	-3.89399000	2.13715500
H	1.07966800	-1.50287500	1.93334500
H	-0.16746500	-1.53580900	-2.23129900
H	-0.83430800	-3.90703100	-2.00914600
C	0.85967800	-0.05151300	-0.28115900
H	2.26164500	1.33856200	-0.67815400
C	0.40993300	2.39527400	-0.12320900
C	-0.93940100	2.30800100	0.24521200
C	1.01728100	3.64764800	-0.25787600
C	-1.66793800	3.46598200	0.47656200
H	-1.41942700	1.33878100	0.35094600
C	0.28124800	4.80494900	-0.02347300
H	2.06294400	3.71961300	-0.54217000
C	-1.05883100	4.71504900	0.34257100
H	-2.71264600	3.39764600	0.75996000
H	0.75536300	5.77454300	-0.12842900
H	-1.63119700	5.61833700	0.52458700
C	1.22135900	1.19128700	-0.37523400
O	-0.57965900	-5.27960900	0.26944400
H	-0.94114000	-5.69912500	-0.52257400

*

prod6dhs

C	-0.00488000	-3.56659800	0.22716700
C	0.67077300	-2.84419900	1.25284300
C	0.96694800	-1.52972200	1.06972400
C	0.60045700	-0.86342400	-0.15651000
C	-0.08085700	-1.61616700	-1.17601600
C	-0.37823500	-2.93620600	-0.99244100
H	0.93136100	-3.37267800	2.16236400
H	1.48043500	-0.96318700	1.83916400
H	-0.35407600	-1.11380400	-2.09811600
H	-0.89020000	-3.49061600	-1.76845200
C	0.88451500	0.43947900	-0.34903600
H	2.14963500	1.86775700	-0.99789800
C	0.36071200	2.87155400	-0.19414500
C	-0.91404500	2.74623500	0.37391900
C	0.89048900	4.14107500	-0.44525700
C	-1.64551000	3.88305200	0.68757200
H	-1.33507900	1.76350000	0.56867000
C	0.15248200	5.27723200	-0.12749100
H	1.87711400	4.24276100	-0.88758500
C	-1.11273900	5.14927100	0.43862200
H	-2.63158100	3.78472400	1.12850800
H	0.56809700	6.25998500	-0.32105200
H	-1.68677400	6.03575900	0.68658800
C	1.17532800	1.69114800	-0.53461800
O	-0.24325200	-4.82248500	0.50112400
C	-0.92556700	-5.67622800	-0.43362800
H	-0.35000700	-5.76057000	-1.35823800
H	-0.98387500	-6.64348400	0.05878100
H	-1.93029000	-5.29342500	-0.62677500

*

prod7dhs

C	0.00933700	-3.68289800	0.21919800
C	0.64422800	-2.94417000	1.25091300
C	0.93780400	-1.61678900	1.08902500
C	0.60533400	-0.95636900	-0.14047600
C	-0.03306800	-1.70913400	-1.18391800
C	-0.31690100	-3.03430200	-1.00070200
H	0.89413300	-3.44433300	2.18048700
H	1.42057100	-1.04949300	1.87776600
H	-0.27717100	-1.20947700	-2.11538200
H	-0.78672700	-3.58954200	-1.80330900
C	0.88706400	0.35581500	-0.31449700
H	2.16519400	1.78385200	-0.91928200
C	0.35082000	2.78857100	-0.17305700
C	-0.93931300	2.66212400	0.35902800
C	0.88652200	4.05756000	-0.41409000
C	-1.68065900	3.79904000	0.64770500
H	-1.36443800	1.67950400	0.54514300
C	0.13790600	5.19339900	-0.12142900
H	1.88485400	4.15923900	-0.82922600
C	-1.14256800	5.06508100	0.40929000
H	-2.67857400	3.70082900	1.06110400
H	0.55715000	6.17611500	-0.30695700
H	-1.72453500	5.95154400	0.63792000
C	1.17582700	1.60810400	-0.48688800

C	-0.27395600	-5.09291000	0.46155800
C	-0.96829800	-5.89792100	-0.35543600
H	0.10866700	-5.49758900	1.39477200
H	-1.13574500	-6.93589800	-0.09002000
H	-1.39890100	-5.56729700	-1.29527500

*

prod8dhs

C	-0.09196900	-3.75300500	0.00937700
C	0.28131000	-3.07863400	1.19840900
C	0.61866400	-1.75095000	1.15150000
C	0.59174700	-1.04852800	-0.09842400
C	0.21040300	-1.74511400	-1.29256600
C	-0.12461000	-3.07296100	-1.23333400
H	0.29668700	-3.62432300	2.13442400
H	0.90793500	-1.21625700	2.04994200
H	0.19249100	-1.20626300	-2.23387000
H	-0.41417000	-3.61453200	-2.12611700
C	0.91865400	0.26741700	-0.14873000
H	2.31626100	1.68911800	-0.37587200
C	0.38762100	2.70434800	-0.04656100
C	-0.98649900	2.58449600	0.20118200
C	0.97164200	3.97015500	-0.15701000
C	-1.76421400	3.72569100	0.33533000
H	-1.44747300	1.60451700	0.29106800
C	0.18524900	5.11002200	-0.02181300
H	2.03637000	4.06659900	-0.34740700
C	-1.17965300	4.98863500	0.22339500
H	-2.82767200	3.63291000	0.52670900
H	0.64038700	6.09035600	-0.10918700
H	-1.79118800	5.87832400	0.32858200
C	1.25002000	1.51911700	-0.19717300
C	-0.43579000	-5.12688400	0.06372400
C	-0.72679900	-6.29881100	0.11106100
H	-0.98226400	-7.33677900	0.15403400

*

prod9dhs

C	-0.08086800	-3.76024800	-0.04633700
C	0.28156800	-3.11196200	1.15077400
C	0.61185900	-1.77637700	1.11611300
C	0.58134300	-1.07031200	-0.12603600
C	0.20994500	-1.74961900	-1.32729300
C	-0.11816100	-3.08548000	-1.28253500
H	0.29737700	-3.66739300	2.08122700
H	0.89536400	-1.24837400	2.02022100
H	0.19104700	-1.20190400	-2.26336100
H	-0.40211800	-3.62141400	-2.18067700
C	0.90008100	0.25693200	-0.16268400
H	2.29673100	1.66742200	-0.37496300
C	0.37811500	2.70039900	-0.04045100
C	-0.99735600	2.59133600	0.20773700
C	0.97746200	3.95999800	-0.14439500
C	-1.76208200	3.73982900	0.34920700
H	-1.46879900	1.61591000	0.29259200
C	0.20273300	5.10653400	-0.00171100
H	2.04291200	4.04652700	-0.33512300
C	-1.16324600	4.99696900	0.24401400
H	-2.82625600	3.65781200	0.54106200
H	0.66733300	6.08278300	-0.08360100
H	-1.76513800	5.89243500	0.35526900
C	1.22687900	1.50806400	-0.19851100
C	-0.42392700	-5.15256200	-0.00561200
N	-0.70155000	-6.27682400	0.02714500

*

sm0dhs

C	-0.44471400	-4.45405700	0.21646700
C	0.20326600	-3.80032500	1.27550500
C	0.62175900	-2.49845500	1.11294100
C	0.38343200	-1.83342600	-0.13220600
C	-0.27956000	-2.52134000	-1.19837700
C	-0.68557900	-3.82423200	-1.01391900
H	0.37149900	-4.31887000	2.21213700
H	1.12608400	-1.96239900	1.91003300
H	-0.45010100	-2.00283400	-2.13588400
H	-1.18769400	-4.36156100	-1.81001200
C	0.77216200	-0.53920100	-0.29669300
H	2.16475600	0.78504500	-0.86415600
C	0.41217800	1.92654300	-0.16201600
C	-0.88199000	1.89430300	0.37522800
C	1.03157900	3.15171900	-0.42819600
C	-1.54549200	3.08288100	0.64196300
H	-1.36939700	0.94578500	0.58424200
C	0.35925300	4.33934600	-0.15801000
H	2.03358100	3.17916500	-0.84580300
C	-0.92577200	4.30552000	0.37631800
H	-2.54603300	3.05944700	1.05973200
H	0.84070200	5.28895300	-0.36345100
H	-1.44775600	5.23273600	0.58691300
C	1.15350400	0.68758700	-0.45601300
H	-0.76974800	-5.48106400	0.35380200

*

sm10dhs

C	-0.27043500	-4.94238700	0.20594600
C	0.43376700	-4.27344200	1.21833400
C	0.76013900	-2.94593300	1.05161200
C	0.37307100	-2.27134700	-0.14985100
C	-0.34698500	-2.97435300	-1.16767000
C	-0.66081500	-4.30215600	-0.97976200
H	0.71759400	-4.80066100	2.12166200
H	1.30476000	-2.39681600	1.81242500
H	-0.63281800	-2.44727600	-2.07178000
H	-1.20448000	-4.85181200	-1.73930100
C	0.68357400	-0.95632200	-0.31979600
H	1.96802900	0.43757100	-0.96557100
C	0.23319300	1.48781400	-0.09421000
C	-1.02986400	1.38553400	0.50426300
C	0.80260100	2.74055500	-0.33234400
C	-1.70310500	2.54402600	0.85685500
H	-1.48159700	0.41500500	0.68951500
C	0.12901000	3.91329100	0.02155100
H	1.78263100	2.81051100	-0.79816200
C	-1.12737000	3.79332100	0.61827800
H	-2.68098300	2.48001200	1.32159500
H	-1.66826200	4.69108600	0.90202500
C	0.99800400	0.28912500	-0.47998300
H	-0.52020800	-5.98995500	0.34508100
C	0.75426900	5.25842500	-0.24096200
H	1.72454100	5.34380600	0.25712200
H	0.91736300	5.41044700	-1.31222400
H	0.11780800	6.06863100	0.11990600

*

sm11dhs
C -0.25003000 -4.91839400 0.30081000
C 0.49782300 -4.21313400 1.25556900
C 0.80308500 -2.88912500 1.03190400
C 0.35152200 -2.25627700 -0.17013800
C -0.41250000 -2.99580600 -1.12850200
C -0.70573000 -4.31878800 -0.88311300
H 0.83202200 -4.71004600 2.15877800
H 1.38087900 -2.31187400 1.74588600
H -0.74729100 -2.49971000 -2.03331000
H -1.28264800 -4.89648300 -1.59579100
C 0.64805800 -0.94713500 -0.39648300
H 1.90896500 0.42311600 -1.13445900
C 0.20931300 1.50327400 -0.22740600
C -1.05414500 1.42154100 0.37274900
C 0.80498200 2.73848300 -0.47701600
C -1.70758400 2.59527400 0.71950800
H -1.52315100 0.46054900 0.56003700
C 0.13532900 3.91119800 -0.12075000
H 1.78165900 2.81848900 -0.94259000
C -1.12276900 3.83715400 0.47897300
H -2.68656900 2.55016600 1.18384300
H -1.64835500 4.74644500 0.75628800
C 0.95800200 0.29115100 -0.60847500
H -0.48288000 -5.96315100 0.48368700
O 0.76853300 5.07235100 -0.38862200
H 0.24308200 5.83072400 -0.11457600
*

sm12dhs
C -0.75841600 -5.65329400 0.31009700
C -0.07859800 -5.02957700 1.36674700
C 0.45867000 -3.77491700 1.17979400
C 0.31177000 -3.12811500 -0.08797900
C -0.38554600 -3.78489500 -1.15079600
C -0.91378200 -5.03986500 -0.94186500
H 0.02213900 -5.53412500 2.32055200
H 0.98998400 -3.26269000 1.97500300
H -0.48740800 -3.28016000 -2.10562600
H -1.44433700 -5.55241600 -1.73587400
C 0.82811300 -1.88168600 -0.28038500
H 2.36218000 -0.73486300 -0.85462800
C 0.72701900 0.61258900 -0.25767900
C -0.56613900 0.75344400 0.26324900
C 1.46163400 1.75791300 -0.58591500
C -1.10572100 2.01481400 0.44526300
H -1.15270900 -0.12222100 0.52941700
C 0.91127900 3.01697900 -0.40029000
H 2.46494700 1.66505600 -0.99147200
C -0.38216000 3.17317300 0.11738900
H -2.10752400 2.11201600 0.85187400
H 1.50299700 3.88759500 -0.66509500
C 1.33591800 -0.70532800 -0.47311800
H -1.17969600 -6.64189600 0.46623600
C -1.00163200 4.50286800 0.32681900
C -0.17205500 5.68979800 0.78056100
C -0.71138700 5.65895400 -0.61127100
H -2.01235400 4.45876800 0.72107300
H 0.89121500 5.52705200 0.92820100
H -0.62673900 6.36270300 1.49918300
H -1.54091500 6.31102700 -0.86152100
H -0.02134000 5.47697100 -1.42960700
*

sm13dhs
C -0.21159400 -5.28277900 0.34636200
C 0.55134000 -4.56217100 1.27800100
C 0.84188700 -3.23874400 1.03482100
C 0.36071200 -2.62141900 -0.16515400
C -0.41748200 -3.37761400 -1.10043600
C -0.69633300 -4.69928000 -0.83459000
H 0.90836200 -5.04744200 2.17881500
H 1.43054700 -2.65025900 1.73054600
H -0.77423500 -2.89434000 -2.00382900
H -1.28390600 -5.28892400 -1.52851700
C 0.64339700 -1.31404100 -0.41036100
H 1.88388900 0.06020600 -1.17646600
C 0.19080800 1.13479000 -0.25230900
C -1.07224100 1.05404800 0.34942500
C 0.76781300 2.38443400 -0.49205600
C -1.74734200 2.21389900 0.70926500
H -1.53317600 0.08691000 0.53124300
C 0.08856400 3.54400700 -0.12640700
H 1.74657700 2.45920700 -0.96079200
C -1.16911000 3.45835700 0.47491100
H -2.72517400 2.14599300 1.17281200
H -1.67906500 4.37649700 0.74898600
C 0.94167200 -0.07449300 -0.63580200
H -0.43322000 -6.32714200 0.54497200
C 0.71122500 4.87775800 -0.37672200
O 0.18161200 5.92058600 -0.07949600
H 1.70819000 4.85674300 -0.86068800
*

sm14dhs
C -0.90004200 -5.47266100 0.18806400
C -0.46395300 -4.83898800 1.36240300
C 0.15749100 -3.61395000 1.27892700
C 0.34567400 -3.00702100 -0.00614500
C -0.10829000 -3.67386300 -1.19125800
C -0.72588500 -4.89872400 -1.08121500
H -0.61615200 -5.31472100 2.32412300
H 0.50897600 -3.09573800 2.16485700
H 0.04367200 -3.20022300 -2.15536600
H -1.07617400 -5.41935000 -1.96478400
C 0.94909300 -1.79388700 -0.09971300
H 2.62831400 -0.71493700 -0.30161800
C 0.93548300 0.69864700 -0.14480400
C -0.45098300 0.87035200 -0.03631300
C 1.78068500 1.81030000 -0.21369300
C -0.99061200 2.14599100 0.00454100
H -1.11490700 0.01238800 0.01375300
C 1.24708100 3.09321000 -0.17247200
H 2.85448700 1.68042500 -0.30017000
C -0.12766800 3.23394500 -0.06383600
H -2.05788200 2.31006400 0.08873600
H 1.87844900 3.97195500 -0.22268800
C 1.54220800 -0.64652800 -0.18593300
H -1.38831600 -6.43959400 0.26457600
N -0.70186800 4.59628100 -0.01952600
O -1.91397500 4.68481500 0.07338000
O 0.07813700 5.52997700 -0.07932500
*

sm15dhs

C	-0.53476500	-5.35036700	0.30137800
C	0.07666700	-4.69374900	1.38116200
C	0.57940500	-3.42490500	1.20462200
C	0.46988700	-2.79765800	-0.07995800
C	-0.15590500	-3.49031000	-1.16819900
C	-0.65316500	-4.75734900	-0.96567000
H	0.15045700	-5.18565100	2.34394000
H	1.05733300	-2.88738900	2.01682800
H	-0.22904200	-3.00085400	-2.13359800
H	-1.13139200	-5.29634200	-1.77515900
C	0.95079100	-1.54108000	-0.26359700
H	2.47261700	-0.32984400	-0.75940200
C	0.73276000	0.94059100	-0.28034500
C	-0.62179300	0.99640400	0.06989000
C	1.46360800	2.11335100	-0.48557800
C	-1.22640200	2.23068000	0.21039900
H	-1.21891900	0.10598900	0.23374300
C	0.82995100	3.33695600	-0.33652900
H	2.51334200	2.10075200	-0.75638300
C	-0.51493000	3.41392900	0.01073200
C	1.42476900	-0.34930400	-0.44507400
H	-0.93123600	-6.35021900	0.45135900
F	-2.51124700	2.31028400	0.54029600
F	-1.11035200	4.58416100	0.14838500
F	1.50598600	4.46371600	-0.52622000

*

sm16dhs

C	-0.69148300	-5.05686500	0.18223600
C	-0.19946000	-4.42163300	1.33213000
C	0.36529900	-3.16975400	1.22438100
C	0.43948000	-2.53679300	-0.05698100
C	-0.06692100	-3.20696300	-1.21572100
C	-0.62695600	-4.45880600	-1.08496800
H	-0.26469800	-4.91523000	2.29478600
H	0.75812500	-2.65016400	2.09203400
H	0.00115700	-2.71510600	-2.18033700
H	-1.01578900	-4.98042800	-1.95179800
C	0.98300900	-1.29192400	-0.17126900
H	2.60403300	-0.16567000	-0.46411100
C	0.92013000	1.20949400	-0.17541300
C	-0.45861400	1.37452000	0.05676600
C	1.74226100	2.33066100	-0.31187900
C	-0.98735000	2.64556700	0.14853400
H	-1.11306700	0.51242900	0.16381300
C	1.20603900	3.60890800	-0.21819400
H	2.80578000	2.20991700	-0.49073500
C	-0.15367800	3.77813100	0.01159500
H	1.82958000	4.48938200	-0.32089100
C	1.52587300	-0.11882700	-0.27532400
H	-1.13645600	-6.04297200	0.27656000
O	-2.29544500	2.93992900	0.36962500
H	-2.84121400	2.15317900	0.45360600
O	-0.66291900	5.01235300	0.10139800
H	-1.61533000	4.96853300	0.26030100

*

sm17dhs

C	5.94098100	0.45674500	0.06354900
C	5.31653100	0.36370000	-1.18884500
C	4.01084200	-0.06978600	-1.26457500
C	3.31424900	-0.42052200	-0.06535100
C	3.97403400	-0.32012900	1.20000500
C	5.27888700	0.11901900	1.25291500
H	5.85962000	0.63216400	-2.08754700
H	3.49664800	-0.15292200	-2.21615100
H	3.43127100	-0.59019200	2.09971800
H	5.79253700	0.20401200	2.20339400
C	2.01695900	-0.83635100	-0.12454900
H	0.71908200	-2.34144300	-0.29079700
C	-0.46014900	-0.49769000	-0.08285400
C	-1.67592100	-1.20356800	-0.18002900
C	-0.46874500	0.88265100	0.09625100
C	-2.88505300	-0.53275700	-0.09785000
H	-1.66128200	-2.27827200	-0.32018500
C	-1.67888500	1.56451700	0.18037200
H	0.45998000	1.44021300	0.17485900
C	-2.88911100	0.87836300	0.08742400
H	-1.67161000	2.63803000	0.32164000
C	0.79049700	-1.25505900	-0.16943000
H	6.96953100	0.80138800	0.11420700
O	-4.09624900	-1.10468100	-0.17844400
O	-4.09772500	1.44083400	0.16001100
C	-4.17459300	-2.50825500	-0.35398900
H	-3.69875800	-2.81671900	-1.29234200
H	-3.71829000	-3.03707400	0.49115200
H	-5.23725000	-2.74052300	-0.39511300
C	-4.19371000	2.84605800	0.34169600
H	-3.72662900	3.15310300	1.28377800
H	-3.73774700	3.38122800	-0.49833900
H	-5.25960900	3.06302400	0.37681800

*

sm18dhs

C	-0.85456500	-5.67142400	0.08056500
C	-0.50585200	-5.04708900	1.28651700
C	0.08182200	-3.80066100	1.25852100
C	0.32483100	-3.16212500	0.00260500
C	-0.04037200	-3.81972000	-1.21320000
C	-0.62605900	-5.06645700	-1.16345500
H	-0.69659700	-5.54418200	2.23047200
H	0.36456400	-3.28823600	2.17192700
H	0.15060400	-3.32221900	-2.15807100
H	-0.90770300	-5.57868500	-2.07610100
C	0.89744400	-1.92284600	-0.03364100
H	2.56361000	-0.83625000	-0.11004500
C	0.89623300	0.57877200	-0.02564500
C	-0.49078300	0.79775800	0.02938600
C	1.74478300	1.69597100	-0.04735000
C	-1.01047300	2.07238800	0.06224200
H	-1.17832900	-0.04436500	0.04523500
C	1.23974900	2.97943800	-0.01367700
H	2.82146500	1.55771100	-0.09269300
C	-0.15853900	3.21143200	0.04404400
H	-2.08492700	2.19413500	0.10423900
H	1.93510900	3.80811400	-0.03324300
C	1.47146300	-0.75754200	-0.05746000
H	-1.31692300	-6.65337500	0.11129800
N	-0.66664700	4.47110300	0.07999300
C	0.22533400	5.61939900	0.05458900
C	-2.10477200	4.68252200	0.11423200
H	-0.36445000	6.53291500	0.10893800

H	0.91131600	5.61099300	0.90923500
H	0.81293700	5.65100400	-0.87064300
H	-2.30750000	5.75189800	0.13700500
H	-2.59297200	4.26537900	-0.77435200
H	-2.55462900	4.23643300	1.00867500

*

sm1dhs

C	-0.67642700	-4.88429400	0.14247600
C	-0.25952300	-4.23497500	1.31400500
C	0.26709500	-2.96485700	1.23262300
C	0.37583200	-2.32760700	-0.04421400
C	-0.05477400	-3.01107700	-1.22553500
C	-0.57516300	-4.28206800	-1.12048900
H	-0.35303400	-4.73163200	2.27265300
H	0.59997700	-2.43255300	2.11737100
H	0.03737200	-2.51434000	-2.18552200
H	-0.90605100	-4.81534300	-2.00404400
C	0.87615800	-1.06351600	-0.13057800
H	2.45467500	0.14730600	-0.34746900
C	0.67702000	1.42458300	-0.10049100
C	-0.71036600	1.50593500	0.08629900
C	1.43000400	2.59873100	-0.19059500
C	-1.32189400	2.74394800	0.18355100
H	-1.31198600	0.60318600	0.15384100
C	0.80359000	3.83523700	-0.09109600
H	2.50469500	2.54924100	-0.33923600
C	-0.57725300	3.93076200	0.10049100
H	-2.39684900	2.79881900	0.32739600
H	1.39867200	4.74022800	-0.16338600
C	1.36974300	0.13191200	-0.19938100
H	-1.09225400	-5.88471800	0.21617300
C	-1.25372700	5.26665500	0.24144700
H	-1.45942200	5.47630600	1.29655600
H	-0.62913400	6.07490400	-0.14425300
H	-2.20878300	5.28493000	-0.28985600

*

sm2dhs

C	-0.84149100	-5.22757000	0.14550600
C	-0.48281400	-4.57833000	1.33669100
C	0.10477700	-3.33448800	1.27876200
C	0.33771400	-2.72462200	0.00407600
C	-0.03749400	-3.40708900	-1.19784100
C	-0.62315600	-4.65045200	-1.11477500
H	-0.66647300	-5.05628200	2.29180800
H	0.39569200	-2.80346000	2.17897000
H	0.14654600	-2.93051300	-2.15486400
H	-0.91311000	-5.18282200	-2.01315000
C	0.91302900	-1.49350300	-0.06393400
H	2.57596300	-0.38861500	-0.18231100
C	0.86767200	1.00295900	-0.11919900
C	-0.52287000	1.17146100	-0.07406100
C	1.70119600	2.12534700	-0.15855400
C	-1.07191900	2.44300500	-0.06785300
H	-1.18510200	0.31055100	-0.04596700
C	1.15670500	3.40303000	-0.15178800
H	2.77974400	2.00680500	-0.19502200
C	-0.22687400	3.55494100	-0.10610800
H	-2.14642900	2.58050500	-0.03297600
H	1.79953800	4.27506900	-0.18126800
C	1.48393500	-0.33244900	-0.12343900
H	-1.30422200	-6.20833800	0.20134100
Cl	-0.91251700	5.14391600	-0.09790800

*

sm3dhs

C	-1.09139800	-5.09525800	0.09870800
C	-0.50815400	-4.58407800	1.26857400
C	0.29286800	-3.46729100	1.19304800
C	0.51492800	-2.84663200	-0.07937000
C	-0.09186900	-3.38706700	-1.25968900
C	-0.88897300	-4.50453900	-1.15835400
H	-0.68882600	-5.06799900	2.22124700
H	0.75914500	-3.04324400	2.07600300
H	0.08645500	-2.90326800	-2.21415500
H	-1.35712500	-4.92877500	-2.03882800
C	1.29187900	-1.73505200	-0.16586500
H	3.09720500	-0.89467100	-0.42953800
C	1.63012100	0.73238900	-0.14895400
C	0.29435100	1.08253800	0.08103000
C	2.60321800	1.72572500	-0.28063700
C	-0.05262100	2.42037100	0.17773200
H	-0.47225400	0.32188400	0.18827900
C	2.24236900	3.06673900	-0.18272700
H	3.64020900	1.45749700	-0.45762200
C	0.91763400	3.41713400	0.04589100
H	2.99793700	3.83752600	-0.28278800
C	2.03848000	-0.68085500	-0.25435600
H	-1.72110900	-5.97732300	0.16837600
C	-1.49327200	2.81910900	0.39819500
F	-1.60486000	3.72681100	1.37915000
F	-2.01830200	3.36225900	-0.71364200
F	-2.25906000	1.76314800	0.72988000
H	0.63582000	4.46152300	0.12681600

*

sm4dhs

C	-0.97912500	-5.86167200	0.11178900
C	-0.60731400	-5.24414200	1.31607800
C	0.04091000	-4.03027400	1.28213900
C	0.32336500	-3.41937000	0.01729600
C	-0.06701400	-4.06884300	-1.19874700
C	-0.71486000	-5.28189700	-1.13877300
H	-0.82826700	-5.72362100	2.26249200
H	0.34376100	-3.52417800	2.19268000
H	0.15457400	-3.59131200	-2.14731300
H	-1.01770200	-5.78942000	-2.04716200
C	0.96181900	-2.22016000	-0.02917600
H	2.67952300	-1.19228800	-0.11649800
C	1.02825200	0.27389800	-0.07542500
C	-0.35589800	0.49478800	-0.05366700
C	1.90949300	1.35631900	-0.10063100
C	-0.84677800	1.78885000	-0.05690800
H	-1.05109800	-0.33963400	-0.03708200
C	1.41354200	2.65673800	-0.10286000
H	2.98218400	1.19165400	-0.11906200
C	0.04056000	2.86828800	-0.08187500
H	-1.91689400	1.96474300	-0.04185100
H	2.09637300	3.49783300	-0.12255400
C	1.59048600	-1.08958300	-0.07409700
H	-1.48807200	-6.82019600	0.14913300
C	-0.52814900	4.26982800	-0.06556100
F	-1.43580300	4.43176000	-1.04366300

F	-1.15102500	4.51636800	1.10073900
F	0.42030500	5.20122100	-0.22725100

*

sm5dhs

C	-0.65209900	-4.87712300	0.15466100
C	-0.24436400	-4.21949200	1.32471200
C	0.26687100	-2.94317600	1.24024800
C	0.36975700	-2.30752900	-0.03786600
C	-0.05119200	-3.00013400	-1.21738000
C	-0.55667300	-4.27703100	-1.10969100
H	-0.33238600	-4.71483200	2.28457300
H	0.59281900	-2.40483300	2.12394600
H	0.03678800	-2.50530100	-2.17875500
H	-0.87967900	-4.81674800	-1.99228000
C	0.85690200	-1.03802100	-0.12774800
H	2.43056600	0.17043700	-0.34771100
C	0.66336900	1.45477500	-0.11584000
C	-0.72294800	1.55152400	0.06851200
C	1.42473000	2.62895600	-0.21513400
C	-1.33464900	2.78955500	0.15337700
H	-1.33298400	0.65568100	0.14682000
C	0.82082600	3.87006700	-0.13090400
H	2.49937600	2.57009700	-0.35942200
C	-0.56319900	3.95671600	0.05471900
H	-2.40906600	2.85572100	0.29725100
H	1.39862500	4.78388800	-0.20582600
C	1.34488000	0.16081700	-0.20160400
H	-1.05565900	-5.88240700	0.23061700
O	-1.09104900	5.18988200	0.13053600
H	-2.04532700	5.16239200	0.25509900

*

sm6dhs

C	-0.81953500	-5.26316200	0.09907000
C	-0.44424800	-4.63507600	1.29571700
C	0.14788000	-3.39199300	1.25085300
C	0.36825700	-2.76113700	-0.01430300
C	-0.02273700	-3.42263000	-1.22123900
C	-0.61271100	-4.66573800	-1.15299600
H	-0.61876100	-5.12735500	2.24529400
H	0.45123300	-2.87680800	2.15610100
H	0.15246700	-2.93098200	-2.17226300
H	-0.91476300	-5.18156900	-2.05702100
C	0.94312000	-1.52608400	-0.06764700
H	2.60473000	-0.42801400	-0.18362300
C	0.91706300	0.97428200	-0.07677100
C	-0.46773400	1.16533000	-0.01035000
C	1.76119100	2.09696100	-0.10905000
C	-1.00928200	2.44071200	0.02279200
H	-1.14061400	0.31199100	0.01369200
C	1.23278200	3.37117700	-0.07501600
H	2.83817000	1.96810000	-0.16237800
C	-0.15736900	3.55675700	-0.00918000
H	-2.08420400	2.55924700	0.07414400
H	1.87267200	4.24575600	-0.09904000
C	1.51342000	-0.36236700	-0.11097200
H	-1.28568100	-6.24283100	0.14381600
O	-0.57350200	4.82813100	0.01744100
C	-1.96727200	5.10496900	0.06654500
H	-2.05133700	6.18995100	0.07375700
H	-2.47576900	4.70494300	-0.81696200
H	-2.41589400	4.69816500	0.97889400

*

sm7dhs

C	-0.89874800	-5.13210700	0.16178800
C	-0.39577300	-4.52411100	1.32168300
C	0.22417000	-3.29777400	1.22814600
C	0.34278800	-2.66388700	-0.04967300
C	-0.17716600	-3.30482600	-1.21905800
C	-0.79197000	-4.53171700	-1.10163400
H	-0.49674800	-5.01809800	2.28096200
H	0.62562900	-2.79842800	2.10356700
H	-0.07707000	-2.81100800	-2.17976700
H	-1.19270700	-5.03173100	-1.97564800
C	0.93591200	-1.44177100	-0.14836500
H	2.59410100	-0.35003200	-0.40575800
C	0.91820800	1.05217300	-0.12150000
C	-0.46042900	1.23058300	0.06758300
C	1.75345900	2.17092100	-0.19257000
C	-0.98715400	2.50373300	0.17958100
H	-1.12347200	0.37188900	0.13447000
C	1.21507000	3.44534200	-0.07772100
H	2.82276800	2.04789000	-0.33573800
C	-0.16091100	3.63893900	0.09858300
H	-2.05179700	2.62174500	0.34901500
H	1.87325300	4.30682200	-0.13339100
C	1.51480700	-0.28582700	-0.23258700
H	-1.38588100	-6.09898600	0.24506700
C	-0.68816600	5.01082500	0.20169700
C	-1.96261000	5.37189800	0.03433600
H	0.05291800	5.77961200	0.40954900
H	-2.25856800	6.41105200	0.12427500
H	-2.74738900	4.66238800	-0.21140800

*

sm8dhs

C	-0.84660700	-5.10061800	0.10078700
C	-0.49175200	-4.46965000	1.30270200
C	0.09339100	-3.22368500	1.26573500
C	0.32649300	-2.59285800	0.00171400
C	-0.04454700	-3.25676700	-1.21138600
C	-0.62696600	-4.50307200	-1.14954200
H	-0.67661800	-4.96264800	2.24990500
H	0.38090200	-2.70601900	2.17474100
H	0.13967100	-2.76434900	-2.16029800
H	-0.91371500	-5.02172500	-2.05689800
C	0.89506300	-1.35713900	-0.04529100
H	2.54820000	-0.23268700	-0.14773300
C	0.82306400	1.13720600	-0.05611400
C	-0.56941000	1.28560700	0.01074800
C	1.64248200	2.27043800	-0.09341300
C	-1.13119300	2.54944600	0.04038400
H	-1.21785100	0.41407400	0.03849400
C	1.07933900	3.53824400	-0.06322300
H	2.72162700	2.16378600	-0.14665800
C	-0.31206800	3.69160200	0.00429900
H	-2.20732400	2.66924900	0.09309000
H	1.71319000	4.41722200	-0.09113400
C	1.45586000	-0.19027800	-0.08503800
H	-1.30743100	-6.08306200	0.13995200
C	-0.89682600	4.99625000	0.03601400
C	-1.39263400	6.09642100	0.06232000

H	-1.83177800	7.06965000	0.08649800

*

sm9dhs

C	-0.84660200	-5.07680600	0.12536800
C	-0.49133400	-4.43693200	1.32319600
C	0.09476300	-3.19246800	1.27719300
C	0.32891500	-2.57187800	0.00663400
C	-0.04250000	-3.24537200	-1.20286500
C	-0.62624800	-4.48973100	-1.13048400
H	-0.67674600	-4.92306300	2.27389000
H	0.38321700	-2.66851800	2.18238700
H	0.14314400	-2.76123100	-2.15583500
H	-0.91344800	-5.01585500	-2.03345900
C	0.90111400	-1.34082800	-0.05029400
H	2.56011100	-0.22079600	-0.15982100
C	0.83226300	1.15285100	-0.08362200
C	-0.56051200	1.29801100	-0.03427600
C	1.65477900	2.28309400	-0.11584200
C	-1.12488600	2.56224000	-0.01643300
H	-1.20865300	0.42664000	-0.01158600
C	1.09280800	3.55300800	-0.09740200
H	2.73406700	2.17547800	-0.15554700
C	-0.29694100	3.69333300	-0.04732900
H	-2.20136300	2.68414000	0.02218300
H	1.72550500	4.43293700	-0.12123900
C	1.46797800	-0.17777900	-0.09995800
H	-1.30835500	-6.05858100	0.17231900
C	-0.88514600	5.00496800	-0.02783100
N	-1.36294200	6.06001000	-0.01196800

*

ts0dhs

C	4.36299300	-2.44855900	-1.19861400
C	4.43119200	-1.06337400	-1.35423900
C	3.31490600	-0.28075300	-1.09663200
C	2.12940000	-0.90536300	-0.67511000
C	2.05653700	-2.29931300	-0.51710500
C	3.18224100	-3.06506500	-0.78160200
H	5.23984600	-3.05351000	-1.40385000
H	5.35438000	-0.59508100	-1.67592500
H	3.35482200	0.79702700	-1.21244600
H	1.13449800	-2.76615900	-0.18717300
H	3.14128100	-4.14159900	-0.66112300
C	0.98121400	-0.10096500	-0.38855700
H	0.01092500	0.02425500	-1.23162700
C	-1.04662800	1.48963500	0.31662800
C	-1.96736600	0.99713800	1.25658800
C	-1.16322600	2.78805100	-0.20670500
C	-3.00420700	1.81900900	1.67411400
H	-1.86048400	-0.00679000	1.65324000
C	-2.20813400	3.59368000	0.22056200
H	-0.44174400	3.15390200	-0.92967400
C	-3.12398300	3.10935600	1.15610900
H	-3.71998600	1.45542600	2.40254600
H	-2.31088900	4.59798200	-0.17435000
H	-3.93846000	3.74547100	1.48616400
C	0.03535600	0.65256700	-0.10258500

*

ts10dhs

C	-0.53613700	-4.37562000	0.04175300
C	0.74716600	-3.81184500	-0.01986000
C	0.84854900	-2.42823100	-0.12005100
C	-0.31403000	-1.63908700	-0.15184400
C	-1.59133800	-2.21349100	-0.09346600
C	-1.68852700	-3.59471300	0.00639700
H	1.82436800	-1.95436000	-0.16663300
H	-2.47916000	-1.59107700	-0.11835000
H	-2.66407700	-4.06461200	0.05892700
C	-0.19298400	-0.21449200	-0.22552500
H	-0.07288500	0.40459500	-1.34445700
C	-0.01198500	2.44780700	-0.10270500
C	-1.17642700	3.22989900	-0.18427600
C	1.24807300	3.03613000	0.09674800
C	-1.07078100	4.60710200	-0.05793500
H	-2.14202700	2.75934600	-0.33576900
C	1.33378100	4.41506000	0.21992700
H	2.13693900	2.41750600	0.16010500
C	0.17891400	5.19523600	0.14288600
H	-1.96121100	5.22295500	-0.11342500
H	2.29853200	4.88320600	0.37847100
H	0.25436700	6.27279700	0.24313400
C	-0.11368900	1.02506000	-0.20082700
C	1.97032900	-4.68798600	0.03075900
H	1.92471600	-5.46259100	-0.73953600
H	2.88615400	-4.11305900	-0.12022800
H	2.04328300	-5.19032900	0.99997600
H	-0.63199300	-5.45477300	0.12160200

*

ts11dhs

C	-0.50378200	-4.39622400	0.16807900
C	0.76077700	-3.79875500	0.10627700
C	0.86476800	-2.41414500	-0.01022700
C	-0.30926400	-1.65236400	-0.05908800
C	-1.58218400	-2.24035400	-0.00162900
C	-1.65948200	-3.62151900	0.11511200
H	1.84550100	-1.95420000	-0.05148000
H	-2.47748900	-1.63068600	-0.03802300
H	-2.62832600	-4.10477200	0.16844400
C	-0.20321800	-0.22774100	-0.14743100
H	-0.08664400	0.38245000	-1.29069100
C	-0.03493900	2.43846600	-0.09479900
C	-1.20098200	3.21535000	-0.19133100
C	1.22321400	3.03218100	0.09679600
C	-1.09882900	4.59482400	-0.08638600
H	-2.16524800	2.74039300	-0.33744800
C	1.30523400	4.41330300	0.19865100
H	2.11372100	2.41713300	0.17095800
C	0.14883800	5.18937900	0.10743200
H	-1.99084200	5.20724700	-0.15319700
H	2.26875200	4.88586600	0.35151300
H	0.22136300	6.26856900	0.19088800
C	-0.13124900	1.01166100	-0.17212300
H	-0.58675300	-5.47538300	0.26215400
O	1.91496800	-4.48947900	0.15439400
H	1.76304600	-5.43655800	0.23828000

*

ts12dhs

C	-0.30580000	-3.51557600	0.05118200
C	0.92804400	-2.87053900	-0.14934200
C	1.00779500	-1.49488300	-0.25477200

C	-0.16340600	-0.72508300	-0.15557600
C	-1.40675400	-1.34794100	0.03615000
C	-1.46464000	-2.72633800	0.13981800
H	1.84233600	-3.45036800	-0.22087300
H	1.96851400	-1.01299200	-0.40348600
H	-2.31198600	-0.75454500	0.11031300
H	-2.42516300	-3.20646000	0.29505700
C	-0.08985700	0.70166800	-0.23837000
H	-0.05526800	1.31026100	-1.33751200
C	0.02066900	3.36247500	-0.06233900
C	-1.16026900	4.12485600	-0.09560900
C	1.27684600	3.97409900	0.09540100
C	-1.07475000	5.50277900	0.03417400
H	-2.12201200	3.63738700	-0.21457200
C	1.34242600	5.35331900	0.22252100
H	2.17806500	3.37089600	0.12337400
C	0.17118800	6.11231300	0.19200700
H	-1.97730600	6.10300700	0.01471900
H	2.30363200	5.83826400	0.34870700
H	0.23064000	7.19066200	0.29517900
C	-0.05452900	1.94268600	-0.16021200
C	-0.41654700	-4.98420500	0.17207400
C	0.48334900	-5.90320800	-0.63788400
C	0.68334900	-5.79667900	0.83478300
H	-1.42895900	-5.33989100	0.33483300
H	1.20927100	-5.43682600	-1.29695000
H	0.02754600	-6.80196600	-1.03795600
H	0.36704900	-6.62112500	1.46398000
H	1.54953500	-5.25699100	1.20504800

*

ts13dhs

C	-0.84672000	-4.01569300	0.16500800
C	0.44793800	-3.48662200	0.09159700
C	0.63258900	-2.11661400	-0.03209500
C	-0.49288400	-1.27836100	-0.07802500
C	-1.79255900	-1.80827900	-0.00777500
C	-1.96011100	-3.18166600	0.11541300
H	1.63367800	-1.69689200	-0.08504300
H	-2.65218900	-1.14721100	-0.04118700
H	-2.95805500	-3.60038300	0.17588800
C	-0.31433800	0.13693600	-0.16781600
H	-0.19093100	0.75319100	-1.31692000
C	-0.00150200	2.79030300	-0.11153800
C	-1.12338100	3.62944500	-0.21093100
C	1.28583300	3.31373600	0.09194300
C	-0.94762300	5.00046100	-0.09578800
H	-2.11081500	3.20810800	-0.36662100
C	1.44109300	4.68756700	0.20479500
H	2.14116100	2.65077500	0.16715900
C	0.32890900	5.52559500	0.11120000
H	-1.80414000	5.66138900	-0.16446400
H	2.42725400	5.10716200	0.36757700
H	0.45865700	6.59862500	0.20323100
C	-0.17469700	1.37090500	-0.19808300
H	-0.95982200	-5.09102500	0.26427400
C	1.63253000	-4.39929100	0.14787200
O	1.52932600	-5.59491300	0.26096700
H	2.62234600	-3.90703200	0.07804300

*

ts14dhs

C	0.00362000	-3.51996500	0.02525500
C	1.21775900	-2.86188700	-0.12684700
C	1.21232600	-1.47764900	-0.20392400
C	-0.01131600	-0.79148000	-0.12244600
C	-1.22753000	-1.47808400	0.03348000
C	-1.21748500	-2.86254800	0.10801800
H	2.13842800	-3.42977500	-0.18156600
H	2.14319500	-0.93354900	-0.31885100
H	-2.16274600	-0.93367100	0.10163800
H	-2.13156800	-3.43056400	0.23010900
C	-0.01637200	0.63533800	-0.16540400
H	-0.09187000	1.31726300	-1.32937200
C	-0.01930500	3.30791400	-0.08507800
C	-1.22022300	4.03001400	-0.16153000
C	1.20927200	3.95226800	0.12780800
C	-1.18350900	5.40872700	-0.00971100
H	-2.16196700	3.51752200	-0.32703800
C	1.22480300	5.33170100	0.27858800
H	2.12884500	3.37945600	0.18140300
C	0.03377100	6.05511300	0.20913400
H	-2.10379400	5.97941400	-0.06027400
H	2.16530200	5.84265600	0.45018500
H	0.05427800	7.13313800	0.32848300
C	-0.04063800	1.87668900	-0.21704900
N	0.01203900	-5.00321300	0.10593900
O	-1.06532500	-5.55231800	0.24214700
O	1.09555000	-5.55166000	0.02846600

*

ts15dhs

C	0.05903300	-3.74539600	0.06615900
C	1.22999900	-3.03004300	-0.19400300
C	1.21633300	-1.65173200	-0.28664700
C	-0.00662200	-0.98540300	-0.10829800
C	-1.19793300	-1.68062200	0.15354400
C	-1.14489800	-3.05886300	0.23791900
H	2.14051900	-1.12149500	-0.48368200
H	-2.14402300	-1.17253700	0.29736500
C	-0.03267800	0.44022400	-0.16916200
H	-0.16807300	1.09187400	-1.31101800
C	-0.04952400	3.11056200	-0.08327500
C	-1.25237800	3.83367900	-0.12780300
C	1.18651200	3.75667200	0.08253800
C	-1.20915900	5.21380600	0.00511600
H	-2.19939100	3.32020700	-0.25604100
C	1.20820900	5.13744300	0.21536200
H	2.10656200	3.18290500	0.11385600
C	0.01567000	5.86095400	0.17599900
H	-2.12975500	5.78556900	-0.02217100
H	2.15398900	5.64948200	0.35078700
H	0.04094600	6.94033400	0.28168900
C	-0.07237000	1.68156600	-0.19196100
F	-2.24171400	-3.75749800	0.48611900
F	0.09037700	-5.05612400	0.15086100
F	2.36035900	-3.70122800	-0.35062300

*

ts16dhs

C	0.42808400	-3.98659200	0.03708600
C	1.58598300	-3.24420100	-0.18911000
C	1.51550600	-1.86496400	-0.29072700
C	0.27630500	-1.22348000	-0.15921000

C	-0.90448800	-1.96351000	0.06187700
C	-0.82159900	-3.33614700	0.16344100
H	2.41769300	-1.28814800	-0.46174200
H	-1.86067900	-1.45926600	0.16051100
C	0.20282500	0.20070200	-0.24831100
H	0.31494400	0.80786300	-1.33830400
C	-0.02113600	2.85460000	-0.08427000
C	-1.23651500	3.49869200	-0.37659700
C	1.10724100	3.58553400	0.32980100
C	-1.31682200	4.87653700	-0.24644500
H	-2.09845600	2.92083900	-0.69304400
C	1.00835600	4.96325300	0.45209500
H	2.03653700	3.07324000	0.55470300
C	-0.19830500	5.60381100	0.16555900
H	-2.24875300	5.38573800	-0.46378500
H	1.86888000	5.53904800	0.77325200
H	-0.26948600	6.68159900	0.26710800
C	0.06703600	1.43643000	-0.17841500
O	0.51091000	-5.31251000	0.13485000
H	2.53024600	-3.76774400	-0.27969300
H	-0.36409400	-5.69392700	0.29327600
O	-1.86201100	-4.17713700	0.38312000
H	-2.70258300	-3.71830000	0.47242800

*

ts17dhs

C	-0.50659100	-3.37719300	0.07862800
C	0.78752200	-2.78771400	-0.06959600
C	0.89999500	-1.41501600	-0.17639100
C	-0.26430100	-0.61323900	-0.13207500
C	-1.52856100	-1.18601100	0.00473700
C	-1.64243600	-2.56730300	0.11442900
H	1.87096900	-0.94698400	-0.28197600
H	-2.41643800	-0.56437700	0.03184000
H	-2.62428100	-3.00792000	0.22914500
C	-0.15374800	0.80781600	-0.22251600
H	0.18416100	1.38544600	-1.27580100
C	-0.14386300	3.47209900	-0.07094700
C	-1.22161100	4.23233500	-0.55942200
C	0.96421200	4.09084500	0.53578400
C	-1.18413000	5.61260600	-0.43362100
H	-2.07112600	3.74058700	-1.02141800
C	0.98541000	5.47246500	0.65064400
H	1.78442800	3.48912000	0.91233200
C	-0.08472500	6.22792100	0.16803200
H	-2.00951300	6.21099300	-0.80213600
H	1.83216800	5.96198400	1.11807300
H	-0.06217400	7.30840600	0.26401600
C	-0.18292600	2.05187800	-0.16122700
O	-0.51195700	-4.70180300	0.17333600
O	1.80680600	-3.65501500	-0.08739200
C	-1.75248800	-5.38532900	0.31965400
H	-2.25579800	-5.08387400	1.24386400
H	-2.40011400	-5.20425300	-0.54417800
H	-1.49791800	-6.44186300	0.37089400
C	3.12510300	-3.15272800	-0.23266700
H	3.24317400	-2.63054000	-1.18922200
H	3.38652800	-2.48623600	0.59741100
H	3.77616900	-4.02472900	-0.21468700

*

ts18dhs

C	0.03241200	-3.48408500	-0.02042600
C	1.21973700	-2.79951100	-0.40819300
C	1.24751700	-1.42866200	-0.50439000
C	0.09893600	-0.66563600	-0.21801000
C	-1.08869500	-1.32684500	0.15344300
C	-1.12292200	-2.69781200	0.25335500
H	2.12374400	-3.35010200	-0.63092000
H	2.16997900	-0.93574100	-0.79535700
H	-1.98478600	-0.75343800	0.36724700
H	-2.05115400	-3.17097800	0.54432600
C	0.12464600	0.75429700	-0.29564200
H	0.37566400	1.35680000	-1.34565800
C	-0.03742100	3.40942300	-0.07577700
C	-1.15932600	4.12492500	-0.53250200
C	1.04560600	4.07322200	0.53018200
C	-1.19201000	5.50211300	-0.37625600
H	-1.98838200	3.59961600	-0.99477300
C	0.99815800	5.45147500	0.67420500
H	1.90095900	3.50651800	0.88206000
C	-0.11669700	6.16055100	0.22350700
H	-2.05256700	6.06454000	-0.72013800
H	1.82583900	5.97405200	1.14011500
H	-0.14927700	7.23848100	0.34292100
C	-0.00383300	1.99286300	-0.19693200
N	0.00325600	-4.83252200	0.08473800
C	-1.22396200	-5.51462900	0.47279200
C	1.19157100	-5.62017500	-0.21503800
H	-1.03435200	-6.58471000	0.53095200
H	-1.56932900	-5.18131000	1.45735100
H	-2.02125700	-5.35036400	-0.26086600
H	0.96774500	-6.67480700	-0.06665100
H	1.50866800	-5.48404800	-1.25492700
H	2.02088500	-5.35541200	0.44987600

*

ts1dhs

C	-0.00247400	-4.32372100	0.09200700
C	1.19498600	-3.59634800	0.15402800
C	1.20106200	-2.21610400	0.04380500
C	-0.01683700	-1.53760100	-0.12502100
C	-1.22647300	-2.24696900	-0.19393900
C	-1.20710400	-3.62712100	-0.08194100
H	2.13428100	-4.12147100	0.29462300
H	2.13334900	-1.66403300	0.09872000
H	-2.16607200	-1.71978000	-0.32205200
H	-2.14210200	-4.17633700	-0.12594200
C	-0.02129400	-0.10922500	-0.20806800
H	-0.02374500	0.50738700	-1.32071700
C	0.00676200	2.55756400	-0.07121100
C	-1.21331500	3.23635100	0.08974100
C	1.22669800	3.25381700	-0.11590100
C	-1.20283900	4.61798700	0.21090500
H	-2.14604000	2.68371700	0.12591100
C	1.21692400	4.63521500	0.00725600
H	2.16068800	2.71558200	-0.23747100
C	0.00713100	5.31231200	0.16950800
H	-2.13624800	5.15377000	0.34027500
H	2.15080900	5.18494800	-0.02096200
H	0.00774900	6.39278800	0.26764400
C	0.00527700	1.13269000	-0.16281600
C	0.00892800	-5.82288200	0.18685300
H	-0.93091400	-6.20577400	0.59017700

H	0.14632400	-6.25982000	-0.80837700
H	0.82738100	-6.17546600	0.81832100

*

ts2dhs

C	0.73323500	-3.78342200	0.09615500
C	1.81141800	-2.89389700	0.11396000
C	1.57300700	-1.53498800	0.00479600
C	0.25232900	-1.07176100	-0.11815400
C	-0.82637500	-1.97150300	-0.13855000
C	-0.58292300	-3.32976600	-0.02926100
H	2.82250700	-3.27017100	0.21534800
H	2.40096900	-0.83445300	0.02206400
H	-1.84346300	-1.60664200	-0.23270400
H	-1.40184000	-4.03925300	-0.03799000
C	0.00107200	0.33286300	-0.20207700
H	0.02829200	0.97072800	-1.32196000
C	-0.50514300	2.95350200	-0.10177200
C	-1.80824100	3.43487100	-0.31241800
C	0.55024600	3.82350800	0.22075400
C	-2.04882600	4.79522700	-0.19052500
H	-2.61199200	2.74926800	-0.55896400
C	0.28964500	5.18053400	0.33835300
H	1.54972900	3.43391700	0.38181600
C	-1.00404100	5.66253300	0.13319100
H	-3.04959100	5.18210500	-0.34517400
H	1.09190700	5.86386200	0.59236300
H	-1.20021600	6.72511500	0.22939100
C	-0.24574000	1.55063100	-0.19331700
Cl	1.03321200	-5.47532300	0.23355700

*

ts3dhs

C	-1.42085000	-3.28831700	0.10802300
C	-0.09199700	-2.85622600	0.14631400
C	0.20994400	-1.50884500	0.04520900
C	-0.84377200	-0.59091300	-0.09425800
C	-2.18096700	-1.01637600	-0.13582200
C	-2.45976600	-2.37201100	-0.03234700
H	1.23901100	-1.16954800	0.08041800
H	-2.98187400	-0.29231800	-0.23956400
H	-3.48669100	-2.71732900	-0.05935400
C	-0.54643700	0.80448700	-0.16612700
H	-0.29487900	1.40899800	-1.31315300
C	-0.00214400	3.42034300	-0.10018800
C	-1.03842400	4.35830900	-0.23282800
C	1.32023500	3.82254700	0.14809300
C	-0.74206500	5.70735600	-0.10392100
H	-2.05485600	4.03056200	-0.42331300
C	1.59640900	5.17621000	0.27370300
H	2.10841500	3.08392200	0.24761700
C	0.56962600	6.11275500	0.14786200
H	-1.53183300	6.44403700	-0.19701300
H	2.61133400	5.50213000	0.47066500
H	0.79237700	7.16950400	0.24996900
C	-0.29709000	2.02072900	-0.20175900
C	1.00746900	-3.88327100	0.31507900
H	-1.64168500	-4.34759700	0.19114400
F	0.90162400	-4.50187300	1.50055700
F	2.22377700	-3.32107700	0.25032400
F	0.93432000	-4.82157500	-0.64010200

*

ts4dhs

C	0.00570700	-3.10359400	0.00691000
C	1.21135800	-2.41736100	-0.15913000
C	1.20923200	-1.03546800	-0.23737500
C	-0.01377500	-0.34890600	-0.14412000
C	-1.22395200	-1.03880100	0.02096900
C	-1.20695000	-2.42462900	0.09707600
H	2.14609400	-2.96301400	-0.22507800
H	2.13881800	-0.49087200	-0.36113700
H	-2.16042600	-0.49723400	0.09634600
H	-2.13189000	-2.97301400	0.22842200
C	-0.02244600	1.07929000	-0.18899700
H	-0.08608600	1.74556200	-1.33306900
C	-0.02809700	3.75006400	-0.08145300
C	-1.23486400	4.46633400	-0.12022600
C	1.20291100	4.40182800	0.09516000
C	-1.20091100	5.84519300	0.02972800
H	-2.17816600	3.94830800	-0.25698300
C	1.21587200	5.78117300	0.24475400
H	2.12636400	3.83332900	0.12217800
C	0.01917500	6.49796800	0.21139800
H	-2.12517800	6.41125700	0.00733300
H	2.15822200	6.29724800	0.38862500
H	0.03740100	7.57612100	0.33025300
C	-0.04585600	2.32107700	-0.20795400
C	0.05037300	-4.61823900	0.08881500
F	-1.16640400	-5.14622300	0.25973000
F	0.82230700	-5.00966800	1.11382000
F	0.57342400	-5.13265500	-1.03476900

*

ts5dhs

C	0.01030000	-4.32432100	0.11262500
C	1.21025600	-3.66146700	-0.19174400
C	1.22431700	-2.28810500	-0.30594600
C	0.03677300	-1.55642900	-0.11261500
C	-1.16420100	-2.22166700	0.18459100
C	-1.17602900	-3.59912900	0.30095800
H	2.11193200	-4.24627300	-0.32983300
H	2.15222900	-1.77466100	-0.53504900
H	-2.08034100	-1.65949100	0.33024000
H	-2.10224900	-4.11368500	0.53721800
C	0.04603200	-0.13087200	-0.21299700
H	0.09174200	0.46290300	-1.31821700
C	-0.02394600	2.53336500	-0.07208000
C	-1.23736000	3.22423200	-0.23860600
C	1.17268000	3.22063900	0.19919400
C	-1.24564900	4.60616900	-0.12698500
H	-2.15299400	2.67981700	-0.44387700
C	1.14480200	4.60309100	0.30516600
H	2.10000800	2.67313800	0.32920500
C	-0.05910300	5.29055100	0.14267800
H	-2.17490600	5.15081500	-0.24890600
H	2.05932100	5.14547800	0.51630200
H	-0.07345100	6.37189400	0.22997700
C	-0.00905500	1.11106800	-0.15130200
O	0.07238000	-5.65654500	0.20950600
H	-0.78315500	-6.04533300	0.42259200

*

ts6dhs

C	0.23442800	-3.86032600	0.00074700
C	1.41223200	-3.14741800	-0.30087900
C	1.38529200	-1.77585800	-0.39757400
C	0.17482900	-1.08338600	-0.18920100
C	-1.00047000	-1.79172100	0.10228800
C	-0.97430300	-3.17255000	0.20194500
H	2.33049300	-3.70322000	-0.44980700
H	2.29634500	-1.23164400	-0.62379700
H	-1.93337000	-1.26017800	0.25769700
H	-1.88860800	-3.70339500	0.43436600
C	0.13792500	0.34272200	-0.26607100
H	0.21606900	0.95560300	-1.35336900
C	-0.04001400	2.99812700	-0.06853400
C	-1.26656700	3.65015900	-0.28773600
C	1.11346300	3.72075800	0.28631300
C	-1.33237100	5.02752000	-0.14445300
H	-2.14811500	3.07831200	-0.55777000
C	1.02898300	5.09820600	0.42209700
H	2.05123200	3.20266800	0.45592200
C	-0.18863200	5.74648800	0.20809100
H	-2.27266000	5.54222600	-0.30546500
H	1.90942000	5.66760800	0.69758000
H	-0.24814800	6.82397500	0.32006000
C	0.03000100	1.58006200	-0.17603800
O	0.36929300	-5.18239900	0.07463200
C	-0.76420400	-5.99428900	0.37232400
H	-0.39341800	-7.01704100	0.38040800
H	-1.17428400	-5.74484800	1.35587200
H	-1.53182700	-5.88782000	-0.40055200

*

ts7dhs

C	0.18199000	-3.94452800	0.09025800
C	1.36036000	-3.17941100	0.12597600
C	1.32749300	-1.80041200	0.01593900
C	0.08941900	-1.15518900	-0.12584300
C	-1.10227700	-1.90012100	-0.16375300
C	-1.04778400	-3.27611600	-0.05166200
H	2.31664000	-3.67889500	0.24246300
H	2.24663600	-1.22502400	0.04697200
H	-2.05827000	-1.39787700	-0.26807100
H	-1.97562500	-3.83604900	-0.06480800
C	0.04267700	0.27193100	-0.21236500
H	0.00223600	0.88277900	-1.32231600
C	-0.01306800	2.93732200	-0.06921200
C	-1.25473600	3.57667600	0.09005100
C	1.18405800	3.67281600	-0.11147800
C	-1.28927600	4.95769700	0.21123600
H	-2.16920700	2.99414300	0.12436700
C	1.12932700	5.05303000	0.01236700
H	2.13475200	3.16446200	-0.23193700
C	-0.10201700	5.69065700	0.17255800
H	-2.23936800	5.46366200	0.33903000
H	2.04506200	5.63268000	-0.01353200
H	-0.13689200	6.77052200	0.27112000
C	0.03032600	1.51416300	-0.16080700
C	0.28755800	-5.40696700	0.21026800
C	-0.70934100	-6.28486100	0.06939300
H	1.28567400	-5.78269200	0.42289100
H	-0.52380300	-7.34763000	0.17652100
H	-1.73112400	-5.99615500	-0.15779500

*

ts8dhs

C	3.43968200	-2.78225300	-0.63265100
C	3.86672100	-1.46740300	-0.88832800
C	2.97574900	-0.41392800	-0.79270500
C	1.64177100	-0.67070000	-0.43362500
C	1.20245500	-1.98081600	-0.17943200
C	2.10121900	-3.02746900	-0.27856400
H	4.90068900	-1.28540200	-1.15696700
H	3.30521300	0.60140500	-0.98507400
H	0.17086700	-2.16945800	0.09776000
H	1.78004400	-4.04357400	-0.08145800
C	0.73019100	0.42371700	-0.30725600
H	-0.04385100	0.78553400	-1.26590900
C	-0.83815600	2.53501500	0.15903000
C	-2.07047300	2.33779400	0.80462800
C	-0.39883000	3.82202700	-0.19524400
C	-2.85825100	3.44068900	1.09941500
H	-2.39301800	1.33748300	1.07318200
C	-1.20017600	4.91238800	0.10855700
H	0.55673900	3.95743000	-0.69057600
C	-2.42405200	4.72085900	0.75189800
H	-3.80967700	3.30466400	1.60072600
H	-0.87255300	5.91200400	-0.15348600
H	-3.04491000	5.57888900	0.98717100
C	-0.00531600	1.40813100	-0.12205600
C	4.36472100	-3.86519400	-0.73126300
C	5.14863500	-4.77912400	-0.81422900
H	5.84525900	-5.58611300	-0.88640700

*

ts9dhs

C	0.02057900	-4.02487500	0.05237000
C	1.23236900	-3.33632600	-0.09674300
C	1.22772200	-1.95428400	-0.18236800
C	0.00436000	-1.26671200	-0.11498600
C	-1.21114900	-1.95550400	0.03394500
C	-1.19917300	-3.33770900	0.11805400
H	2.16529600	-3.88589500	-0.14291600
H	2.15952300	-1.41070300	-0.29324600
H	-2.14786400	-1.41206500	0.08992800
H	-2.12535500	-3.88778400	0.23660500
C	-0.00295500	0.16053900	-0.16794800
H	-0.05425600	0.82332100	-1.32051400
C	-0.02054600	2.83220700	-0.08122800
C	-1.23085800	3.54106200	-0.14094700
C	1.20428200	3.49206500	0.10770300
C	-1.20697800	4.92089800	0.00154000
H	-2.16934500	3.01697300	-0.28744100
C	1.20702900	4.87233300	0.24940500
H	2.13071600	2.92943900	0.15028600
C	0.00674900	5.58186500	0.19582100
H	-2.13414600	5.48130300	-0.03674700
H	2.14442600	5.39470800	0.40259300
H	0.01699600	6.66077000	0.30862500
C	-0.02975500	1.40221700	-0.20067000
C	0.02953300	-5.45957700	0.14122200
N	0.03710700	-6.61540200	0.21233100

*

ts7-0beckm

C	1.17990600	-0.04268400	-0.60980400

N	1.58727300	0.87880500	0.01998400
C	-0.31279400	0.77007100	-0.64315100
H	-0.37925900	0.82266300	-1.73668900
H	-0.26366700	1.79469100	-0.26255300
H	1.33223800	-0.99972300	-1.09081400
O	3.81324300	-0.30348000	0.02943700
H	4.54392300	0.11888100	-0.43455300
H	4.18052800	-0.59994100	0.86888000
C	-1.34666200	-0.07525900	0.10540100
C	-2.68115500	0.57730200	-0.33426500
H	-3.49742900	0.06582800	0.18303900
H	-2.72782500	1.63665200	-0.06571900
H	-2.85376900	0.47511400	-1.40972100
C	-1.16265400	0.06020600	1.62005300
H	-1.95902300	-0.48444300	2.13317600
H	-0.20977600	-0.36073700	1.95589000
H	-1.21114800	1.10499500	1.94118900
C	-1.32349700	-1.54538000	-0.32680300
H	-1.36362700	-1.65512200	-1.41623300
H	-0.44547300	-2.07664400	0.05814100
H	-2.19612600	-2.05869000	0.08357100

*

ts7-0bbeckm

C	0.50534100	-0.26216700	0.01241800
N	0.80130100	0.83945100	-0.31596400
C	-0.90574200	-0.01065700	-0.91104600
H	-0.74696700	-0.88040400	-1.56019700
H	-0.87642000	0.88103900	-1.54387600
H	0.71627400	-1.18521600	0.53554100
O	2.87359500	0.11646100	1.02903200
H	3.65743700	-0.01319700	0.45809600
H	3.19448500	0.55145100	1.82425100
O	4.98159800	-0.31168500	-0.69372300
H	5.42730100	0.38758100	-1.18051400
H	5.62499100	-1.01672000	-0.57691100
C	-2.11872300	-0.01886000	0.02149800
C	-2.22909800	1.31081300	0.77455300
H	-3.14691900	1.31796900	1.36766600
H	-1.39203100	1.46161500	1.46319400
H	-2.26724900	2.16109300	0.08700600
C	-3.29886100	-0.17305700	-0.96866500
H	-3.26180400	-1.12510200	-1.50663100
H	-4.23163900	-0.14968700	-0.39889700
H	-3.33104700	0.64368300	-1.69558100
C	-2.09837900	-1.20518900	0.99160500
H	-1.92989000	-2.15587000	0.47361600
H	-1.34337100	-1.08536600	1.77705300
H	-3.06277400	-1.27846000	1.49969400

*

ts6-9bbeckm

C	0.37620800	0.79845300	-0.72449300
N	1.56477200	0.99903700	-0.66520200
C	0.27900600	0.72785700	-2.46701600
H	-0.61543400	1.34640700	-2.56970300
H	1.16651800	1.19414400	-2.89829200
O	1.97352300	1.19496000	1.40674700
H	2.25348600	0.34093700	1.80231900
H	2.74327900	1.77425300	1.38980800
O	2.34236900	-1.24775300	2.54800700
H	3.03889500	-1.88965200	2.37719100
H	2.17697800	-1.27603600	3.49632700
C	-0.82891100	0.51084200	0.05353900
C	-0.74552300	-0.42577300	1.08321300
C	-2.04075200	1.13938700	-0.22287000
C	-1.91204100	-0.69182000	1.79678200
H	0.18132400	-0.93566600	1.32140900
C	-3.13385900	0.80562700	0.57379000
H	-2.14934000	1.88364700	-1.00434100
N	-3.07898900	-0.09384200	1.55637700
H	-1.90324600	-1.42302800	2.59956400
H	-4.09541800	1.28165600	0.40803000
O	0.05162800	-0.54169700	-2.78370700
C	1.20222900	-1.32918100	-3.12835500
H	1.89965500	-1.35743600	-2.28473100
H	0.82719400	-2.32732400	-3.34109900
H	1.68984900	-0.90884800	-4.01172000

*

ts6-8bbeckm

C	0.19887800	0.84411300	-0.49959300
N	1.36198300	1.20608900	-0.48360000
C	-0.02675300	1.17572400	-2.15694400
H	-1.00875600	1.65011800	-2.08271200
H	0.73127000	1.87818700	-2.50870600
O	2.12162200	0.93426000	1.42272600
H	2.60603900	0.07585300	1.36603900
H	2.78486000	1.63398000	1.43312000
O	3.29578400	-1.48749400	1.25536400
H	4.19806200	-1.66354600	0.97202600
H	3.08830900	-2.14126600	1.93032800
C	-0.90805000	0.32091900	0.28851300
C	-0.87696600	0.47627000	1.68324400
C	-1.99418800	-0.30254900	-0.34284800
C	-1.94652900	0.01064900	2.43657800
H	-0.03069300	0.95841600	2.15571400
C	-3.04754300	-0.77385900	0.42870900
H	-2.00749000	-0.44624300	-1.41766800
C	-3.02654800	-0.61354300	1.81380500
H	-1.93697500	0.13882100	3.51330000
H	-3.88659400	-1.26465800	-0.05142500
H	-3.85729700	-0.97564300	2.41023900
O	-0.08517700	0.00404000	-2.80959200
C	1.14882700	-0.46391700	-3.36730100
H	1.88361300	-0.62504500	-2.57148300
H	0.91948000	-1.40602900	-3.85996300
H	1.52952000	0.25955400	-4.09416100

*

ts6-6bbeckm

C	-0.23107000	0.63094900	0.13538900
N	0.47356800	1.04437100	-0.74827300
C	-1.79037700	0.76000800	-0.57626800
H	-2.31175000	1.01953800	0.35086900
H	-1.77225200	1.55600300	-1.32230600
O	2.42799200	0.77311600	0.06602800
H	2.87017700	0.14606000	-0.55310200
H	2.84749300	1.63145400	-0.06255500
O	3.58774700	-1.05552200	-1.54806800
H	3.82820000	-0.93794200	-2.47189600
H	4.22078400	-1.67691300	-1.17548900
C	-0.16778500	0.01244500	1.50368800
H	-0.05808200	-1.07273100	1.34758700

H	0.74375400	0.39976800	1.97704400
O	-1.33195500	0.34304400	2.18157000
C	-1.42802100	-0.26921400	3.46589200
H	-0.59719800	0.04318700	4.10837900
H	-2.36824600	0.07061800	3.89601200
H	-1.43819600	-1.36124200	3.37122300
O	-2.11535400	-0.45798400	-1.00738500
C	-1.82582900	-0.73709400	-2.38362800
H	-0.75241400	-0.61317900	-2.56728600
H	-2.11990500	-1.77034900	-2.55187100
H	-2.40094000	-0.06774600	-3.02884700

*

ts6-5bbeckm

C	-0.13363100	0.27827100	-0.21794400
N	0.73345800	0.84938200	-0.81491900
C	-1.46921500	0.68742300	-1.33068300
H	-2.17586400	0.96846600	-0.54696700
H	-1.15203000	1.51779300	-1.96278800
O	2.47129100	0.30143800	0.38758100
H	3.17806200	0.09656800	-0.26717700
H	2.75418100	1.09307400	0.85932700
O	4.38927600	-0.35486300	-1.40323200
H	4.81077700	0.23711600	-2.03309600
H	5.03275300	-1.03833200	-1.19270100
C	-0.42593600	-0.60496600	0.93879100
H	-1.17354500	-1.35005300	0.65718400
H	0.51517100	-1.08692300	1.21430900
C	-0.94126300	0.20026400	2.13150800
F	-1.17030500	-0.61185400	3.15689400
F	-0.05744100	1.13311400	2.50313300
F	-2.09166900	0.83087900	1.81963600
O	-1.78475200	-0.44836600	-1.92769600
C	-1.17664700	-0.69911400	-3.20961900
H	-0.09212600	-0.78157500	-3.08872500
H	-1.59227700	-1.64181000	-3.55669900
H	-1.42863700	0.10714900	-3.90240600

*

ts6-4bbeckm

C	-0.10034200	0.57203200	-0.51988700
N	1.02895700	0.98608800	-0.60172200
C	-0.59601300	0.85964900	-2.14463000
H	-1.53658200	1.36873500	-1.91820600
H	0.12377300	1.51218100	-2.64237100
O	1.78551400	0.79093600	1.37193100
H	1.82583700	-0.14074000	1.68084200
H	2.68452700	1.13056300	1.30297600
O	1.28881300	-1.72754300	2.24125100
H	1.78734000	-2.54147500	2.11540700
H	0.94166000	-1.76184600	3.13960800
C	-1.05524400	-0.06205900	0.42707300
H	-1.92381300	-0.44030200	-0.11522200
H	-0.52240700	-0.90459800	0.88242800
C	-1.47017400	0.92081700	1.53355000
H	-2.11672300	1.71594700	1.15235900
H	-0.59443100	1.34846500	2.02613500
F	-2.18709000	0.18730300	2.45678100
O	-0.79571200	-0.33785400	-2.69464600
C	0.30433500	-0.89041000	-3.43568900
H	1.14989600	-1.07226000	-2.76430300
H	-0.05631800	-1.82916400	-3.84924400
H	0.59481500	-0.20778400	-4.23848500

*

ts6-3bbeckm

C	-0.26248700	0.37589900	0.25937400
N	0.47097200	1.02668400	-0.43816600
C	-1.74893400	0.69957900	-0.53094800
H	-2.33959900	0.83882900	0.38196300
H	-1.66746600	1.60990400	-1.12734200
O	2.40339800	0.64459100	0.44553200
H	2.92884900	0.19290500	-0.25407700
H	2.78293100	1.52395400	0.55065200
O	3.81318300	-0.72425500	-1.41998300
H	4.13842100	-0.38849100	-2.26043000
H	4.43949000	-1.39471100	-1.13039400
C	-0.31767700	-0.53907200	1.44641400
H	-0.80800900	-1.45956900	1.11446200
H	0.72547600	-0.74487300	1.69971900
C	-1.06736900	0.10529300	2.58334400
C	-2.26355200	-0.31233100	2.99571800
H	-0.57113100	0.93754000	3.07656900
H	-2.76192200	0.16190200	3.83438300
H	-2.77007100	-1.15502000	2.53112300
O	-2.08731300	-0.41349400	-1.18485600
C	-1.71983800	-0.46661300	-2.57125800
H	-0.63028800	-0.41263300	-2.66908500
H	-2.08463200	-1.42076400	-2.94424900
H	-2.19143300	0.35765500	-3.11312000

*

ts6-2bbeckm

C	-0.48114100	0.24965700	0.56889500
N	0.15801200	0.96413000	-0.15780000
C	-2.04536800	0.66069500	-0.05419500
H	-2.54125800	0.79023300	0.91121200
H	-1.99117200	1.58845700	-0.62606000
O	2.18299000	0.52611700	0.45434300
H	2.63332600	0.18997300	-0.35401300
H	2.56855300	1.38875500	0.64315300
O	3.40256300	-0.51952300	-1.72928300
H	3.61761800	-0.06105800	-2.54684100
H	4.07255100	-1.19951600	-1.61007100
C	-0.41463700	-0.75652300	1.66493900
H	-1.15708700	-1.52942400	1.44767700
H	0.58655100	-1.18875300	1.60568400
C	-0.65797300	-0.10922500	3.03403700
H	-1.65011600	0.34402700	3.10901300
H	-0.59282000	-0.88519500	3.79888400
H	0.09628800	0.64953900	3.24999000
O	-2.48274800	-0.41811000	-0.70205400
C	-2.24035300	-0.44979800	-2.11760500
H	-1.16224600	-0.44806000	-2.30845000
H	-2.68440900	-1.37473600	-2.47764100
H	-2.71448000	0.41102700	-2.59638000

*

ts6-1bbeckm

C	-0.35869000	0.17099700	1.10023500
N	0.17308300	0.94478500	0.34884300
C	-2.00011700	0.63439100	0.72350200
H	-2.36415200	0.66720600	1.75333500
H	-2.00875400	1.61182500	0.23882100

O	2.25588000	0.43158900	0.57698400
H	2.55408400	0.20762600	-0.33445300
H	2.69963400	1.25224100	0.81852600
O	3.03206500	-0.30647500	-1.91275000
H	3.15182600	0.27704900	-2.66784900
H	3.66957100	-1.02006400	-2.01291700
C	-0.16020900	-0.93563000	2.05907300
H	-0.45248800	-0.60411400	3.05892400
H	-0.78973800	-1.77852800	1.76461500
H	0.89579300	-1.20665800	2.04313800
O	-2.51881400	-0.38055200	0.03784400
C	-2.47871700	-0.26592100	-1.39461100
H	-1.43927100	-0.19726500	-1.73158300
H	-2.94119300	-1.17087100	-1.78096500
H	-3.04281500	0.61494500	-1.71190600

*

ts6-10bbeckm

C	1.20822000	0.84124500	-0.39664800
N	2.22876600	1.08002100	0.24076500
C	1.93438000	1.10167700	-1.89219500
H	1.17419900	1.74406500	-2.34443400
H	2.87686000	1.64121700	-1.77607100
O	1.86528600	0.84808300	2.24150600
H	2.40757100	0.04853200	2.44692900
H	2.34987700	1.60945100	2.58172400
O	3.25519600	-1.40394100	2.74142500
H	4.21005500	-1.47160100	2.83427400
H	2.87369600	-2.08344900	3.30566000
C	-0.18474300	0.47209900	-0.32589700
C	-0.84767500	0.48925600	0.91285000
C	-0.89621600	0.10876400	-1.48921500
C	-2.19161100	0.16719000	0.99308300
H	-0.30558900	0.76298000	1.80897400
C	-2.22925800	-0.22013800	-1.40933100
H	-0.40006000	0.05093000	-2.45134500
C	-2.89461300	-0.18956800	-0.16916700
H	-2.68357600	0.19834500	1.95687000
H	-2.78899300	-0.50986400	-2.29093600
O	-4.18583400	-0.51554900	-0.20128200
C	-4.94491500	-0.51026200	1.00417800
H	-4.96052100	0.48885800	1.45060100
H	-5.95420600	-0.79532200	0.71437800
H	-4.54817900	-1.24000800	1.71716300
O	2.02568900	-0.08524400	-2.52635800
C	3.25892700	-0.78892000	-2.35066800
H	3.42130000	-1.00760200	-1.28984100
H	3.15999500	-1.71656300	-2.91013400
H	4.09045400	-0.19731500	-2.74579600

*

ts6-0beckm

C	0.57643900	-0.00134500	-0.81292600
N	0.99259000	0.53849300	0.18628300
C	-0.93919800	0.65552700	-1.13073400
H	-0.84425800	0.82084100	-2.20714800
H	-1.03817000	1.59065800	-0.57428200
H	0.92064000	-0.79145200	-1.47394000
O	2.77196500	-0.38216300	0.53579900
H	3.43273700	0.32519700	0.53693000
H	2.72914400	-0.71585900	1.44323100
O	-1.83000700	-0.30057900	-0.85160400
C	-2.40536300	-0.27286100	0.46397400
H	-2.91741200	0.67897000	0.62695400
H	-3.11467600	-1.09622100	0.49762600
H	-1.62294500	-0.41883100	1.21737200

*

ts6-0bbeckm

C	-0.23375800	-0.98891500	-0.04101100
N	0.11441200	0.13350000	-0.21961100
C	-1.78977200	-0.96263300	-1.04270800
H	-1.64633200	-1.97125000	-1.43281700
H	-1.65822300	-0.15785200	-1.76668900
H	-0.03886800	-1.92192300	0.47091400
O	1.92107000	0.15544700	1.08211500
H	2.71047100	0.25989700	0.50570800
H	1.90622900	0.92104800	1.66626800
O	4.11686300	0.29897000	-0.51784200
H	4.37772400	1.00820400	-1.11225600
H	4.92156100	-0.16620100	-0.27054800
O	-2.76730400	-0.87193100	-0.18077500
C	-3.26151700	0.45083000	0.12252000
H	-4.06153400	0.30885900	0.84424500
H	-2.45222600	1.04481200	0.55766400
H	-3.64035000	0.91707700	-0.78927200

*

ts5-9bbeckm

C	0.44037100	0.79167200	-0.14411400
N	1.61177300	1.00702500	-0.30677500
C	0.62294000	0.86603700	-1.86185800
H	-0.32350700	1.39444500	-2.01702200
H	1.40606000	1.47811100	-2.31159000
O	2.16180300	1.23967100	1.99392700
H	2.43098200	0.45418900	2.50461400
H	2.85655700	1.89665000	2.09282800
O	2.30852900	-1.17363200	3.32402600
H	2.86390200	-1.91380500	3.05868900
H	2.28869800	-1.18800600	4.28644700
C	0.63564400	-0.55161100	-2.43784000
C	-0.77482500	0.49983100	0.57843200
C	-0.65654300	-0.17460000	1.79685300
C	-2.02816400	0.87829600	0.09182000
C	-1.84128800	-0.43233000	2.48440900
H	0.30078400	-0.49090100	2.19473300
C	-3.13357300	0.57141100	0.87824100
H	-2.16501600	1.39541700	-0.85127300
N	-3.04590800	-0.07042300	2.04483200
H	-1.81214200	-0.95692300	3.43431500
H	-4.13096200	0.84966500	0.55271200
F	0.37317100	-0.45164100	-3.74042100
F	1.82216700	-1.12535200	-2.26632500
F	-0.29665300	-1.31962100	-1.86446400

*

ts5-8bbeckm

C	0.14885000	0.77676200	0.06966400
N	1.28055600	1.11684000	0.32375000
C	0.83280800	1.14087500	-1.46121700
H	-0.09199400	1.57681900	-1.85611100
H	1.59996100	1.91079700	-1.55165000
O	1.31063700	0.87138300	2.70497100
H	2.00084600	0.20300600	2.89471000

H	1.59613700	1.68197100	3.13683900
O	3.18052300	-1.07428000	3.19954300
H	4.08974300	-1.09179200	2.88792900
H	3.13994000	-1.65249100	3.96660900
C	1.26715600	-0.13620400	-2.18174200
C	-1.18886400	0.33796500	0.33919600
C	-1.50503200	0.08822800	1.68845600
C	-2.14125500	0.17863500	-0.68060600
C	-2.79403700	-0.31874500	1.99954300
H	-0.74516200	0.22372800	2.45122100
C	-3.42184600	-0.22686200	-0.34291300
H	-1.89463200	0.34774600	-1.72270900
C	-3.74611800	-0.47478500	0.99263500
H	-3.05509900	-0.51363800	3.03343800
H	-4.16713200	-0.35552200	-1.11920900
H	-4.75124000	-0.79378900	1.24764900
F	1.37203200	0.15672000	-3.47976900
F	0.36966300	-1.11680300	-2.03319700
F	2.44087800	-0.56637700	-1.73101700

*

ts5-6bbeckm

C	0.32530300	0.72957000	0.28859700
N	0.75655000	1.27682500	-0.68110000
C	-1.11495700	1.02470500	-0.55533200
H	-1.66989600	1.09711400	0.39631900
H	-1.23136300	1.95769200	-1.10577700
O	2.96169200	0.93719200	0.29589200
H	3.45867100	0.25283200	-0.19870900
H	3.53594500	1.70689500	0.34599800
O	4.18530000	-1.10375600	-1.05310200
H	4.34459700	-1.11556900	-2.00150000
H	4.85929700	-1.66286600	-0.65541000
C	0.36824700	0.05398800	1.61924600
H	0.60349100	-1.00471800	1.43100100
H	1.21081400	0.52113400	2.14853000
O	-0.87089600	0.25462900	2.20775800
C	-1.02468300	-0.42653900	3.45775600
H	-0.29589300	-0.05909700	4.18717400
H	-2.03289100	-0.20207200	3.79949500
H	-0.91145000	-1.50688500	3.31835800
C	-1.51068300	-0.20091600	-1.37659200
F	-2.82346500	-0.14152600	-1.58593000
F	-1.22445900	-1.32815900	-0.70797400
F	-0.87644800	-0.23301800	-2.54375700

*

ts5-5bbeckm

C	0.36742200	0.44112900	0.07195400
N	0.94422500	1.16463300	-0.68381300
C	-0.88539000	0.95454200	-1.00694100
H	-1.64074200	1.02156800	-0.21455300
H	-0.82057800	1.93140500	-1.48912100
O	2.99826100	0.68941900	0.52288000
H	3.61564100	0.20950200	-0.06926100
H	3.48451800	1.44259000	0.87157900
O	4.56553900	-0.77500200	-1.16140100
H	4.89372200	-0.47314000	-2.01361800
H	5.18980800	-1.43560700	-0.84649100
C	0.27656300	-0.47462600	1.22252700
H	-0.01283000	-1.47067400	0.87815100
H	1.29422100	-0.48804600	1.62988100
C	-0.71812500	0.02544900	2.27359500
F	-0.66794300	-0.75194000	3.34203900
F	-0.44091700	1.28674500	2.62254400
F	-1.97224900	0.00689500	1.77959200
C	-1.14947300	-0.15847600	-2.02224100
F	-2.34868500	0.05588100	-2.55148200
F	-1.14413600	-1.35801600	-1.41993500
F	-0.22943400	-0.16481900	-2.97936500

*

ts5-4bbeckm

C	0.10101300	0.59643400	0.06980000
N	1.16540800	1.09901300	-0.15390900
C	-0.04018000	0.99284600	-1.59136400
H	-1.04120000	1.43028100	-1.52214100
H	0.62106300	1.74155800	-2.03300500
O	1.77405400	0.87665900	2.18464100
H	1.82249800	-0.02387500	2.55232000
H	2.58023600	1.33859500	2.43227400
O	1.10242700	-1.71806900	2.89074900
H	1.61899900	-2.51333700	2.72268700
H	0.70114700	-1.84325600	3.75781000
C	-0.91102100	-0.02927900	0.93417700
H	-1.73129300	-0.42515700	0.33434000
H	-0.38857100	-0.85616000	1.43723400
C	-1.42221400	0.96952200	1.99624600
H	-1.99517100	1.78422000	1.54499300
H	-0.59130600	1.35892400	2.58719300
F	-2.26135500	0.24432100	2.80969100
C	0.02751300	-0.30653600	-2.40091200
F	-0.37826700	-0.02709900	-3.63546500
F	1.26694000	-0.78331400	-2.43077700
F	-0.77650800	-1.24380200	-1.88109300

*

ts5-3bbeckm

C	0.32198300	0.47981200	0.51845600
N	0.83790300	1.19668300	-0.28942100
C	-1.03019100	1.00868300	-0.38078700
H	-1.67336500	1.05306500	0.51132900
H	-1.02999000	1.99378500	-0.84770500
O	2.99356900	0.74813700	0.69549700
H	3.52496800	0.20314500	0.07884100
H	3.52720500	1.52076100	0.90246300
O	4.33825400	-0.89231100	-1.03623800
H	4.55791200	-0.67598500	-1.94709500
H	4.98921700	-1.53655200	-0.74275900
C	0.25986500	-0.40951500	1.70396900
H	0.00486900	-1.41071100	1.34422300
H	1.29430100	-0.40890900	2.06685200
C	-0.73125600	0.10093800	2.72088500
C	-1.89153600	-0.50729900	2.96234100
H	-0.43962400	0.99024200	3.27383500
H	-2.57149600	-0.12945600	3.71832800
H	-2.18995600	-1.41133300	2.43701000
C	-1.45243500	-0.07439700	-1.37169900
F	-2.70604900	0.18142600	-1.73931600
F	-0.66772000	-0.08711000	-2.44308700
F	-1.40971800	-1.28895100	-0.80065900

*

ts5-2bbeckm

C	0.25632300	0.44801300	0.65097600
N	0.64463100	1.25543700	-0.14380200
C	-1.20727500	1.05590800	-0.01536700
H	-1.72455500	1.13876500	0.94649800
H	-1.23673000	2.03739300	-0.49142100
O	2.92620500	0.79491900	0.52275500
H	3.40447800	0.25617700	-0.14069300
H	3.46176000	1.57892400	0.67412400
O	4.12190500	-0.85927500	-1.30479300
H	4.23442900	-0.66841200	-2.24059700
H	4.82837400	-1.46525800	-1.06234200
C	0.39331100	-0.57504700	1.70630600
H	0.28323000	-1.54724300	1.21556600
H	1.44029100	-0.45889600	2.00821400
C	-0.59007200	-0.40884600	2.86648900
H	-1.62161200	-0.59505600	2.55759400
H	-0.34088400	-1.14852600	3.62861800
H	-0.51270400	0.57961900	3.32511400
C	-1.79804500	-0.00380800	-0.94710300
F	-3.06138800	0.33509500	-1.19143800
F	-1.12327000	-0.07058700	-2.08929400
F	-1.78089300	-1.21509800	-0.37103900

*

ts5-1bbeckm

C	0.25154700	0.21645200	1.08504400
N	0.61321600	1.11630400	0.38346900
C	-1.23941600	0.89495700	0.52549800
H	-1.75032800	0.76974300	1.48626800
H	-1.29602400	1.95048200	0.25564900
O	2.89360900	0.55816900	0.82487200
H	3.30380200	0.21373600	0.00384600
H	3.45816400	1.27389900	1.13131600
O	3.89784800	-0.53387100	-1.47077400
H	3.95586700	-0.09529600	-2.32464900
H	4.58161800	-1.21020500	-1.46285300
C	0.41095400	-0.91922600	1.99542100
H	-0.08027300	-0.70099500	2.94675100
H	-0.02507000	-1.81468300	1.54879300
H	1.48996800	-1.03141800	2.12960400
C	-1.80890100	0.01622000	-0.58949100
F	-3.09857500	0.31675700	-0.71752200
F	-1.18238300	0.23439000	-1.73970700
F	-1.69528200	-1.28467900	-0.28334300

*

ts5-10bbeckm

C	0.89119400	0.85679500	0.11393000
N	1.90736200	1.13753000	0.71623200
C	2.03664200	1.10567700	-1.12348800
H	1.32203100	1.57912900	-1.80621400
H	2.84562600	1.82323800	-0.98226000
O	1.16537000	0.93286200	2.98147600
H	1.71129600	0.19442400	3.32040200
H	1.42365800	1.71201800	3.48246400
O	2.65112900	-1.21975000	3.82526300
H	3.60035200	-1.32357000	3.71453100
H	2.39452200	-1.79007900	4.55546500
C	2.58944100	-0.22171300	-1.64206400
C	-0.47812300	0.52081600	-0.05040300
C	-1.24724900	0.37134100	1.12255700
C	-1.07462700	0.35240900	-1.31992800
C	-2.59150900	0.07042400	1.02728100
H	-0.77153700	0.50140900	2.08942200
C	-2.40998400	0.05298600	-1.40934400
H	-0.49691800	0.43738700	-2.23361900
C	-3.18600400	-0.08947400	-0.23801600
H	-3.17049000	-0.03788600	1.93542400
H	-2.89266700	-0.08422400	-2.36955000
O	-4.46476600	-0.37567800	-0.43680800
C	-5.34456500	-0.53423000	0.67744700
H	-5.39897600	0.39065600	1.25909800
H	-6.31824800	-0.75343500	0.24527100
H	-5.02323900	-1.36850300	1.30778000
F	3.11278700	0.00834400	-2.84955400
F	1.63222700	-1.14860500	-1.76052600
F	3.53684700	-0.69566300	-0.83817500

*

ts5-0beckm

C	1.16916600	0.17042400	-0.58441200
N	1.40500500	0.87691900	0.34292700
C	-0.32400200	0.96132800	-0.41947200
H	-0.43277900	1.25984000	-1.46789900
H	-0.39358700	1.86297800	0.19720600
H	1.38268600	-0.58913000	-1.32950200
O	3.62647100	-0.39955500	0.05062300
H	4.43734800	0.04234300	-0.22467600
H	3.89834800	-1.03547100	0.72183300
C	-1.31800500	-0.10570500	0.07151700
F	-2.53640500	0.37224700	-0.13693800
F	-1.13626500	-0.35671400	1.36058400
F	-1.15998400	-1.24201500	-0.61728100

*

ts4-9beckm

C	0.65192300	0.60574500	0.30445200
N	1.67812900	1.24985000	0.30907400
C	-0.05521000	2.06667400	0.59126900
H	-0.61593000	1.80725100	1.49650800
H	0.61386000	2.88713700	0.87231000
O	3.37306100	-0.29520700	-0.10881300
H	3.96142600	0.13374700	-0.74067700
H	3.90594000	-0.45622500	0.67840700
C	-0.86617600	2.46116100	-0.64506200
H	-0.20152600	2.69651400	-1.48054400
H	-1.58679800	1.69431900	-0.93811100
C	-0.05677000	-0.65407500	0.16208600
C	0.62909600	-1.75354600	-0.36327000
C	-1.39534300	-0.77551700	0.54570100
C	-0.08431400	-2.94504900	-0.48122300
H	1.66997600	-1.68025100	-0.65462800
C	-1.99570400	-2.02086200	0.38495200
H	-1.96553800	0.04862500	0.96030100
N	-1.36040500	-3.07998500	-0.11971400
H	0.39796500	-3.82923800	-0.88634400
H	-3.03230900	-2.16982000	0.67051200
F	-1.55223000	3.59211800	-0.27430400

*

ts4-9bbeckm

C	0.65411300	0.47026300	-0.57140500
N	1.84680100	0.58215400	-0.64819200
C	0.90391900	0.52575900	-2.26145000

H	0.14083400	1.29098000	-2.44278300
H	1.83742800	0.93332100	-2.65735200
O	2.26737700	0.96716600	1.71854000
H	2.34443200	0.33141600	2.45231600
H	3.03174300	1.54764900	1.76641000
O	1.87907200	-0.96895400	3.66062800
H	2.38729000	-1.78191500	3.74550100
H	1.69167800	-0.67713900	4.55868700
C	0.62615700	-0.86855600	-2.81455200
H	1.46147400	-1.54047100	-2.60135700
H	-0.30747300	-1.29176000	-2.43469200
C	-0.62706200	0.27657300	0.07151600
C	-0.63823500	-0.23998500	1.37024000
C	-1.82522700	0.58283400	-0.57918100
C	-1.88706900	-0.42110400	1.96207600
H	0.27142400	-0.49043900	1.90256800
C	-3.00639400	0.36508900	0.12310300
H	-1.86369800	0.98067600	-1.58728600
N	-3.04072200	-0.12839300	1.36235000
H	-1.95575300	-0.82507900	2.96735000
H	-3.96408600	0.59219800	-0.33445000
F	0.51161900	-0.69499600	-4.17384900

*

ts4-8beckm

C	0.64875700	0.62459700	0.30603400
N	1.70241300	1.24444500	0.30400900
C	-0.07674300	2.04814900	0.58250900
H	-0.62065000	1.82928300	1.50745000
H	0.60212800	2.87667200	0.81459900
O	3.35122500	-0.21384200	-0.07606200
H	3.93855900	0.28820600	-0.65366500
H	3.83424800	-0.33866100	0.74979400
C	-0.91863300	2.41697500	-0.64067600
H	-0.27977100	2.57204600	-1.51436900
H	-1.68263100	1.66836300	-0.86116200
C	-0.03560200	-0.64057000	0.15969000
C	-1.37101600	-0.77404800	0.57381200
C	0.66341000	-1.72498400	-0.39856900
C	-2.00308100	-1.99967100	0.43368400
H	-1.91285700	0.05841300	1.01049200
C	0.00937200	-2.94022300	-0.54056700
H	1.69542200	-1.60416600	-0.70482800
C	-1.31535500	-3.07785200	-0.12588600
H	-3.03100400	-2.11600000	0.75739500
H	0.53462800	-3.78352800	-0.97429800
H	-1.81707600	-4.03295500	-0.23941900
F	-1.54159900	3.59975300	-0.31909900

*

ts4-8bbeckm

C	0.23223700	0.60503700	-0.42040900
N	1.37537700	0.87978100	-0.69277800
C	0.15528300	0.73851200	-2.10987500
H	-0.75784600	1.34525400	-2.11320800
H	0.90965300	1.33079300	-2.63161000
O	2.40192900	0.82024000	1.50220500
H	3.14996000	0.19682600	1.40717800
H	2.78700100	1.66490300	1.75350800
O	4.43640800	-1.01969200	1.22035000
H	5.22253900	-0.91086800	0.67767300
H	4.68199600	-1.60380200	1.94373500
C	0.03608900	-0.66465100	-2.69609300
H	1.01867500	-1.13774600	-2.76247100
H	-0.64973900	-1.30146100	-2.13177200
C	-0.87259700	0.25974300	0.43256900
C	-0.59592000	0.06383800	1.79728000
C	-2.17382800	0.12821000	-0.07929000
C	-1.64547900	-0.26413800	2.64423200
H	0.42060000	0.18060100	2.15890800
C	-3.20485100	-0.20021100	0.78606400
H	-2.38707700	0.27463500	-1.13318100
C	-2.94006800	-0.39636000	2.14301500
H	-1.45181500	-0.41661800	3.69987400
H	-4.21381600	-0.30575500	0.40432800
H	-3.75179200	-0.65513100	2.81467400
F	-0.47040100	-0.48444600	-3.96404500

*

ts4-6beckm

C	0.49348000	0.53275300	0.17651300
N	0.65810600	1.70664000	0.34642800
C	-1.05952200	0.84169500	0.66822400
H	-1.09564300	0.04939600	1.42650500
H	-1.24271500	1.79902500	1.16655300
O	3.01662000	1.50107100	-0.28758000
H	3.27326600	2.00751900	-1.06578100
H	3.61908100	1.78073400	0.41030000
C	0.99286200	-0.82485000	-0.21112300
H	1.16829800	-0.79505000	-1.29834900
H	1.96574500	-0.93976200	0.28957000
O	0.03819900	-1.74779900	0.17842800
C	0.43698700	-3.10300200	-0.05292300
H	1.34403100	-3.33835000	0.51407800
H	-0.38637100	-3.72232300	0.29673100
H	0.60425400	-3.27782200	-1.12113100
C	-1.97381400	0.67006300	-0.54407700
H	-1.78343800	1.44639900	-1.29041000
H	-1.87882100	-0.32735500	-0.97959800
F	-3.24657700	0.81969600	-0.04956400

*

ts4-6bbeckm

C	0.02658700	0.62503600	-0.09015600
N	0.55781000	1.08315400	-1.05561500
C	-1.33127100	0.76291700	-1.08189900
H	-1.95198200	1.17727100	-0.27579200
H	-1.30646200	1.48920700	-1.89769600
O	2.65067400	0.88509800	0.31775200
H	3.25070900	0.20141500	-0.04277100
H	3.18368400	1.67759100	0.42820900
O	4.18698200	-1.15867900	-0.69452600
H	4.55991300	-1.16006400	-1.58113600
H	4.78621800	-1.67466500	-0.14700600
C	-0.01896400	0.08298100	1.30136500
H	0.56311200	-0.85123400	1.29065500
H	0.52205800	0.81305900	1.92092300
O	-1.35151500	-0.08493900	1.64266900
C	-1.53480300	-0.52078100	2.99231300
H	-1.13760500	0.22094400	3.69369500
H	-2.60894700	-0.62159300	3.13334300
H	-1.04915000	-1.48932400	3.15369100
C	-1.70716800	-0.63382700	-1.56179100
H	-0.98594400	-0.99996800	-2.29740000

H -1.80737700 -1.33504300 -0.72959500
F -2.93434000 -0.48453100 -2.16446200
*

ts4-5beckm
C -0.17078600 0.88968400 -0.01305400
N -0.59754400 1.91979400 0.42592400
C -1.59839300 0.29608500 0.62981400
H -1.17094300 -0.44771200 1.31056500
H -2.19400300 0.98298100 1.24370700
O 1.42290900 3.15961600 -0.24400500
H 1.28807700 3.88370700 -0.86505500
H 1.88000600 3.54830800 0.51007800
C 0.88565800 0.15542700 -0.74612000
H 0.52875300 -0.08668800 -1.75171500
H 1.72546100 0.85407600 -0.81526500
C 1.30624200 -1.12882600 -0.02823700
F 2.37026100 -1.64455500 -0.62156800
F 0.31091700 -2.03303100 -0.05469700
F 1.58962300 -0.87411600 1.25638600
C -2.44775900 -0.20587600 -0.54113300
H -2.67623200 0.60723000 -1.23645600
H -1.96977800 -1.04060900 -1.05965600
F -3.60967400 -0.65466000 0.03029200
*

ts4-5bbeckm
C 0.25641600 0.35613000 -0.35088900
N 0.89536900 1.02811900 -1.10147900
C -0.90715700 0.73007500 -1.55753800
H -1.65743800 1.13040300 -0.86785500
H -0.68520100 1.52203100 -2.27981600
O 2.80066200 0.75008500 0.56785100
H 3.54985700 0.22341200 0.22100800
H 3.18059900 1.56685900 0.90418200
O 4.78891200 -0.84778600 -0.44034300
H 5.32262200 -0.62788500 -1.20965200
H 5.34013500 -1.39725600 0.12486800
C 0.08954800 -0.47730400 0.85525100
H 0.09538400 -1.53397500 0.57192500
H 0.98304500 -0.25609000 1.45193300
C -1.18936900 -0.15542000 1.62706800
F -1.18587400 -0.79282900 2.78700400
F -1.28985000 1.16333800 1.84231600
F -2.27378100 -0.53594000 0.92600700
C -1.22840300 -0.57565300 -2.28194100
H -0.35155200 -0.94606300 -2.82054100
H -1.61776900 -1.33820100 -1.60281300
F -2.21201800 -0.24869100 -3.18089100
*

ts4-4beckm
C 0.21005400 0.57936800 0.10129500
N 0.04434200 1.73427300 0.38486500
C -1.36764700 0.44552700 0.64027800
H -1.21864700 -0.27840400 1.44745900
H -1.82475200 1.33481500 1.08775400
O 2.27520100 2.39911900 -0.22865300
H 2.32076300 3.02864200 -0.95662000
H 2.75576300 2.80945700 0.49868600
C 1.05120100 -0.51413300 -0.42792500
H 0.65173400 -0.80607900 -1.40552800
H 2.03961600 -0.06768800 -0.57239400
C 1.11530300 -1.74157900 0.49435000
H 0.12846600 -2.18978500 0.65055800
H 1.57159900 -1.49581900 1.45726500
F 1.90662400 -2.66020500 -0.14618900
C -2.20376500 -0.00962300 -0.55931500
H -2.20720200 0.75303100 -1.34301100
H -1.86391200 -0.96914700 -0.95921900
F -3.47386200 -0.16758300 -0.06868600
*

ts4-4bbeckm
C 0.18396200 0.38901300 -0.46222700
N 1.23533900 0.92847800 -0.66086600
C 0.02848200 0.64349800 -2.12156400
H -0.94080500 1.15225800 -2.08909700
H 0.73316900 1.32424800 -2.60853100
O 1.80771400 0.83906800 1.70762300
H 1.78470100 0.01028800 2.21807800
H 2.62458600 1.29649000 1.92594500
O 0.94653500 -1.56459500 2.80797700
H 1.45460900 -2.36582400 2.97195800
H 0.34926600 -1.47092800 3.55856900
C -0.80375200 -0.24105400 0.43203700
H -1.61102500 -0.69538600 -0.14497300
H -0.25589900 -1.01617100 0.98606000
C -1.35118700 0.78284300 1.45059100
H -2.03393300 1.49718100 0.98380500
H -0.52988000 1.29642500 1.95384000
F -2.05914600 0.04597300 2.37591500
C 0.06860600 -0.72932000 -2.79033400
H 1.06963800 -1.16365100 -2.72184000
H -0.67363800 -1.41826800 -2.37725200
F -0.23982800 -0.49449900 -4.10732100
*

ts4-3beckm
C -0.31090000 0.60635400 0.06822800
N -1.38217900 1.01344000 0.42819800
C -0.82448500 -0.84499600 0.67352200
H 0.00580200 -1.01842200 1.36862200
H -1.75357100 -0.84723100 1.25214600
O -1.00341400 3.28083500 -0.21084500
H -1.62781200 3.59237200 -0.87518800
H -1.10487800 3.87609900 0.53998600
C 1.03315700 0.83569500 -0.54008400
H 1.02804700 0.35825100 -1.52582600
H 1.08231800 1.92081900 -0.67948600
C 2.13760000 0.29875700 0.33353400
C 2.92097000 -0.71401600 -0.03120000
H 2.29324900 0.80813300 1.28148500
H 3.72740700 -1.05300600 0.60985500
H 2.80592000 -1.22009300 -0.98681600
C -0.93782600 -1.81139800 -0.50493600
H -1.69625100 -1.47528100 -1.21750500
H 0.02404800 -1.95486100 -1.00457500
F -1.32593600 -3.00941200 0.04085100
*

ts4-3bbeckm
C -0.00674000 0.36624600 0.11093500
N 0.59733300 1.04454600 -0.66716600

C	-1.27419800	0.76750300	-0.93637700
H	-1.96642600	1.03400400	-0.12595400
H	-1.17224500	1.64304800	-1.58130400
O	2.63768300	0.73452900	0.65817300
H	3.30410100	0.19159400	0.19033700
H	3.07957400	1.55445900	0.89655700
O	4.41903600	-0.90901700	-0.64112800
H	4.84916200	-0.71482000	-1.47899400
H	5.02053400	-1.47664300	-0.15022600
C	-0.15838200	-0.45566400	1.33970600
H	-0.36189000	-1.48576300	1.03017100
H	0.83896700	-0.41122600	1.79217200
C	-1.24243700	0.08696300	2.23646900
C	-2.38692100	-0.55574200	2.45962600
H	-1.03167100	1.03521800	2.72454700
H	-3.13223300	-0.14919200	3.13458300
H	-2.60907400	-1.51683400	2.00208900
C	-1.62964400	-0.47679900	-1.74128700
H	-0.85591300	-0.69661700	-2.48151500
H	-1.80536600	-1.34865800	-1.10486800
F	-2.80355900	-0.16789500	-2.38705800

*

ts4-2beckm

C	0.49730100	0.07450300	0.06956200
N	0.78323100	1.18763800	0.42435600
C	-1.00837000	0.45259100	0.64295100
H	-1.11519500	-0.33560200	1.39518700
H	-1.11779500	1.41312100	1.15757000
O	3.07050300	1.08025100	-0.17974200
H	3.31011900	1.73999500	-0.83990500
H	3.63726100	1.25433400	0.57989700
C	0.88746800	-1.22492100	-0.53534400
H	0.42802100	-1.26482100	-1.52906700
H	1.97032200	-1.14402200	-0.66578400
C	0.48078300	-2.42921000	0.31827200
H	-0.60436400	-2.52908200	0.40315100
H	0.85711300	-3.33209400	-0.16513200
H	0.91787400	-2.37723600	1.31766200
C	-1.96245800	0.40418800	-0.55229500
H	-1.68485700	1.14977400	-1.30274700
H	-2.00768600	-0.59219600	-1.00054300
F	-3.20013900	0.70859500	-0.04506100

*

ts4-2bbeckm

C	-0.15238600	0.31624900	0.37090800
N	0.28848700	1.06887000	-0.44703300
C	-1.57690900	0.75233000	-0.45795500
H	-2.13032300	1.10346000	0.41850300
H	-1.55279500	1.57370000	-1.17880900
O	2.54371200	0.71608500	0.53019900
H	3.12768400	0.23871800	-0.09268100
H	3.01573900	1.51520700	0.78061300
O	4.08006600	-0.73637400	-1.23551500
H	4.37047600	-0.41645600	-2.09468100
H	4.75317500	-1.35317700	-0.93304600
C	-0.08240100	-0.62299800	1.51029500
H	-0.15091000	-1.63267500	1.09036800
H	0.93882500	-0.48338300	1.88062100
C	-1.14988700	-0.37903100	2.57910300
H	-2.16110000	-0.54592500	2.19859200
H	-0.98525000	-1.08688700	3.39278300
H	-1.08044300	0.62929000	2.99259100
C	-2.07963800	-0.52383900	-1.12895400
H	-1.38590700	-0.85153800	-1.90810700
H	-2.25340600	-1.32842700	-0.40897900
F	-3.28227700	-0.18629300	-1.69981100

*

ts4-1beckm

C	0.55978500	-0.26170800	0.21696500
N	0.84897800	0.90402000	0.23119000
C	-0.94865000	0.31461800	0.63611300
H	-1.07565600	-0.21272200	1.58667700
H	-1.03701200	1.38662700	0.84344900
O	3.15605100	0.55229600	-0.28623600
H	3.43837000	0.97111000	-1.10680700
H	3.73933800	0.90093800	0.39695100
C	0.92045700	-1.67717300	0.03164100
H	2.00267300	-1.71029900	-0.10808000
H	0.61732100	-2.25142500	0.91002600
H	0.41379200	-2.07308900	-0.85198700
C	-1.90021400	-0.05811600	-0.50385500
H	-1.58998100	0.41352100	-1.44082800
H	-1.98871000	-1.14061000	-0.62814500
F	-3.12318600	0.43653200	-0.12951700

*

ts4-1bbeckm

C	-0.24008800	0.07146200	0.79477600
N	0.12537000	0.96492300	0.08934700
C	-1.73800000	0.63361500	0.18544800
H	-2.22431300	0.72026800	1.16283900
H	-1.78536400	1.62001800	-0.28299000
O	2.44310800	0.49561700	0.73666400
H	2.97299200	0.21290400	-0.03654400
H	2.92280500	1.22750400	1.13511000
O	3.84992700	-0.39649800	-1.44841600
H	4.05149800	0.12848100	-2.22845700
H	4.55680200	-1.04314400	-1.36396500
C	-0.05802800	-1.03139300	1.74495000
H	-0.57910800	-0.79950900	2.67699000
H	-0.45139100	-1.95995900	1.32642100
H	1.02040200	-1.10403200	1.90632100
C	-2.27687400	-0.43473800	-0.76184800
H	-1.67360600	-0.48466400	-1.67235200
H	-2.34173900	-1.41943600	-0.29156400
F	-3.54848400	-0.02221300	-1.07904400

*

ts4-10beckm

C	0.60943500	1.58055700	0.30089900
N	1.53393400	2.39669900	0.24332600
C	-0.40923800	2.79428100	0.53577900
H	-0.89204000	2.51804400	1.47866900
H	0.08110600	3.75867400	0.71085100
O	3.38080100	1.34524500	-0.09103100
H	3.81204200	1.94377000	-0.71379100
H	3.86246200	1.43408400	0.74107900
C	-1.33106800	2.92205800	-0.67796500
H	-0.75444000	3.16623700	-1.57447600
H	-1.92320700	2.02053400	-0.84891700
C	0.22406300	0.20160600	0.22366000

C	-1.06284700	-0.21016800	0.63888000
C	1.13291200	-0.75350000	-0.27035000
C	-1.42136800	-1.53325400	0.56674000
H	-1.78096300	0.50243000	1.03119400
C	0.77001400	-2.08391200	-0.35504900
H	2.12083900	-0.44321400	-0.58743100
C	-0.51100400	-2.48759700	0.06480200
H	-2.39942700	-1.87069900	0.88905000
H	1.48217000	-2.80058800	-0.74360000
O	-0.95615400	-3.73591400	0.02875900
C	-0.12010800	-4.77819800	-0.47600200
H	0.79022700	-4.86864100	0.12375900
H	-0.70816700	-5.68898100	-0.38851900
H	0.12620900	-4.59759000	-1.52647300
F	-2.18760600	3.96220700	-0.39692200

*

ts4-10bbeckm

C	1.26231700	0.60610100	-0.26649500
N	2.35198500	0.79984100	0.22546100
C	2.25764600	0.62117100	-1.63278700
H	1.63504600	1.30967600	-2.21479200
H	3.24227500	1.09003300	-1.56933000
O	1.77011400	0.83606400	2.60121000
H	2.39537500	0.20360300	3.00722900
H	1.96225200	1.69084300	2.99743400
O	3.49167200	-1.01239000	3.72317200
H	4.45187200	-0.96815800	3.71477100
H	3.24737900	-1.52371400	4.49987500
C	2.37002500	-0.80767300	-2.15336900
H	3.01837700	-1.40112200	-1.50397100
H	1.39822000	-1.29479200	-2.26168800
C	-0.14394800	0.38178200	-0.27839100
C	-0.79055200	0.21939700	0.96211900
C	-0.88420600	0.32788700	-1.48003800
C	-2.15552300	0.00660800	1.00278900
H	-0.20611400	0.27390200	1.87540200
C	-2.23974900	0.11804500	-1.43574800
H	-0.40399900	0.45053600	-2.44563600
C	-2.89190900	-0.04643800	-0.19426900
H	-2.64057200	-0.11450800	1.96282200
H	-2.83244100	0.07450000	-2.34181000
O	-4.20160600	-0.24633200	-0.26397100
C	-4.95972400	-0.42960500	0.93209900
H	-4.90252200	0.46030200	1.56594800
H	-5.98563000	-0.57986200	0.60331900
H	-4.61400200	-1.31343800	1.47629400
F	2.94752000	-0.70026500	-3.39969900

*

ts4-0beckm

C	0.75394000	0.01153100	-0.44537800
N	0.96486400	0.67717200	0.51819100
C	-0.79485100	0.62718800	-0.24084000
H	-0.89887300	1.08681400	-1.23011300
H	-0.93239200	1.42482400	0.49962100
H	1.05970700	-0.67481800	-1.22740500
O	3.32505000	-0.34358900	0.04233500
H	4.04521800	0.22719600	-0.24722400
H	3.72781400	-0.96515100	0.65839700
C	-1.69585800	-0.56592800	0.10033200
H	-1.66586000	-1.33796500	-0.67363400
H	-1.43575800	-0.98565400	1.07589000
F	-2.95715100	-0.03534100	0.14815700

*

ts4-0bbeckm

C	-0.14134200	-0.28207200	0.16308200
N	0.02996700	0.78005600	-0.34521200
C	-1.61421800	-0.16189500	-0.63798800
H	-1.54625400	-1.11137300	-1.18092700
H	-1.72585000	0.62747800	-1.39013400
H	0.17787300	-1.10977300	0.78724000
O	2.21001600	0.22494600	0.97475900
H	2.98402100	0.06473400	0.39461300
H	2.54842200	0.70388200	1.73733700
O	4.27652400	-0.26064500	-0.75296500
H	4.69355500	0.42353600	-1.28492700
H	4.93536100	-0.95002600	-0.62794600
C	-2.69418100	-0.02828500	0.43877500
H	-2.66503100	-0.85093700	1.15888200
H	-2.61254600	0.93416800	0.95107500
F	-3.87814700	-0.08464700	-0.24966600

*

ts3-0beckm

C	0.51761900	-0.00152300	-0.45840200
N	1.11395900	0.62029200	0.35651900
C	-0.96896600	0.90491200	-0.05894000
H	-1.16441700	1.21337200	-1.08962900
H	-0.72336200	1.76770500	0.55850500
H	0.47775900	-0.76459500	-1.22307600
O	3.16395400	-0.61441400	-0.09752400
H	3.89961500	-0.08769400	-0.42858000
H	3.51940500	-1.12118200	0.64072000
C	-1.85759900	-0.09071100	0.54914100
H	-1.80043900	-0.21540800	1.62603300
C	-2.75442000	-0.76158000	-0.18638100
H	-2.82456500	-0.64498300	-1.26555300
H	-3.47289000	-1.42543300	0.28294700

*

ts3-0bbeckm

C	-0.32755600	-0.13436400	0.00544000
N	0.07265100	0.80079700	-0.59935300
C	-1.82057700	-0.02004700	-0.98670800
H	-1.78036700	-1.06526800	-1.30522300
H	-1.67500400	0.64078100	-1.83994900
H	-0.24011900	-0.90418300	0.75886000
O	2.04553300	0.42107700	0.92935100
H	2.85539600	0.11767100	0.47092400
H	2.32992600	1.10321100	1.54480300
O	4.25836300	-0.51658800	-0.41295300
H	4.75236500	-0.02257300	-1.07348300
H	4.86877700	-1.15541900	-0.03375400
C	-2.86431800	0.37934500	-0.03530700
H	-3.03375700	1.44341400	0.09682900
C	-3.62321300	-0.53249400	0.58541700
H	-3.46452600	-1.60115600	0.46008900
H	-4.45211900	-0.22953200	1.21635900

*

ts2-9beckm

C	0.14614700	1.12223700	0.16289200

N	0.91033400	2.05849600	-0.01138600
C	-1.04312300	2.13231400	0.61413800
H	-1.25751300	1.74055900	1.61417100
H	-0.68545600	3.15657200	0.75510000
O	2.78477200	1.29720500	-0.70025300
H	3.04688400	1.99405100	-1.31549500
H	3.40441300	1.34545500	0.03922200
C	-2.16150200	2.06759200	-0.40998000
H	-1.83754000	2.47656800	-1.36929900
H	-2.53719200	1.05461500	-0.55969100
H	-2.97948300	2.69239100	-0.03936300
C	-0.01140500	-0.32860400	0.08591200
C	0.85450800	-1.08224700	-0.71070200
C	-1.02849600	-0.96673600	0.79983100
C	0.64978900	-2.46036100	-0.75068000
H	1.65486800	-0.61936000	-1.27276100
C	-1.12542900	-2.35105600	0.69017300
H	-1.72718600	-0.42822400	1.42994100
N	-0.31126600	-3.08464200	-0.06987500
H	1.29287600	-3.08692700	-1.36115800
H	-1.89516100	-2.88981100	1.23416700

*

ts2-9bbeckm

C	0.54006900	0.52036300	-0.98809100
N	1.69483100	0.73582300	-1.22906300
C	0.43574500	0.70042100	-2.69543400
H	-0.34292800	1.46863900	-2.65388700
H	1.29780900	1.16225500	-3.18054900
O	2.51501100	0.94214400	1.01659600
H	2.79276000	0.20716100	1.59248200
H	3.25043000	1.55897700	0.96866700
O	2.70290300	-1.29956600	2.63057200
H	3.26843100	-2.06186100	2.47155700
H	2.69141700	-1.17025900	3.58436300
C	0.08061400	-0.64719800	-3.27213100
H	0.92953900	-1.33161400	-3.22035000
H	-0.78437400	-1.10064200	-2.78587500
H	-0.15837400	-0.48895600	-4.32880100
C	-0.58098700	0.17569600	-0.13694000
C	-0.31623900	-0.48385600	1.06535200
C	-1.89436600	0.48418500	-0.49751900
C	-1.41339500	-0.80129200	1.86423700
H	0.68944200	-0.74266500	1.37398100
C	-2.90307800	0.12330100	0.39020800
H	-2.14421100	0.99118400	-1.42278700
N	-2.67310400	-0.50728000	1.54347200
H	-1.26441500	-1.31604400	2.80836100
H	-3.94026200	0.34813900	0.16189400

*

ts2-8beckm

C	0.14409000	1.13354800	0.16343000
N	0.94242600	2.05971800	0.01988900
C	-1.06514500	2.12368900	0.53345600
H	-1.34541500	1.76369400	1.52865200
H	-0.71783800	3.15256200	0.66763500
O	2.79286900	1.31369600	-0.49976200
H	3.10710000	2.04489300	-1.04798300
H	3.32005700	1.33671500	0.31009800
C	-2.13692400	2.04147400	-0.54146500
H	-1.76071400	2.41644000	-1.49583100
H	-2.51224500	1.02683100	-0.68060200
H	-2.96720100	2.68284100	-0.23291000
C	0.00069200	-0.31177900	0.08104100
C	-1.05352400	-0.94354500	0.75838900
C	0.90629600	-1.05967100	-0.68872900
C	-1.19031800	-2.32132600	0.67697700
H	-1.75925300	-0.37448300	1.35369700
C	0.74555800	-2.43597600	-0.77295400
H	1.71481800	-0.56670600	-1.21252800
C	-0.29523600	-3.06569500	-0.09151300
H	-1.99799300	-2.81395100	1.20628300
H	1.43418700	-3.01910700	-1.37394500
H	-0.41296800	-4.14170200	-0.16290100

*

ts2-8bbeckm

C	0.28643900	0.43907500	-0.78336700
N	1.46616800	0.65562800	-0.91147100
C	0.37572500	0.36510200	-2.48076000
H	-0.44580100	1.06881300	-2.65142800
H	1.26108100	0.82087400	-2.92779200
O	2.23686700	0.79627800	1.33590000
H	2.98435900	0.16656900	1.38389200
H	2.60768600	1.66668800	1.50879400
O	4.27430500	-1.05019100	1.51617700
H	5.10719700	-1.04387900	1.03630200
H	4.43512300	-1.51968000	2.33978400
C	0.18620100	-1.07323100	-2.90225600
H	1.07800100	-1.66064800	-2.67602000
H	-0.68117200	-1.54275300	-2.43671100
H	0.04298400	-1.07880600	-3.98743100
C	-0.91537800	0.21953800	-0.01775900
C	-0.78062500	0.09652400	1.37547300
C	-2.17085800	0.13870700	-0.63888300
C	-1.92199800	-0.11259700	2.13796500
H	0.20237800	0.17866700	1.82685000
C	-3.29762400	-0.06686400	0.14210000
H	-2.27833800	0.23480500	-1.71407100
C	-3.17204900	-0.19525900	1.52659500
H	-1.83307000	-0.21079100	3.21394000
H	-4.27300300	-0.12960700	-0.32646600
H	-4.05783600	-0.35973600	2.13095200

*

ts2-6beckm

C	-0.18674600	0.59355900	0.13757200
N	-0.49871700	1.74280500	0.28073900
C	-1.73569700	0.13404500	0.51675100
H	-1.43915500	-0.58688700	1.28839300
H	-2.32297500	0.92543200	0.98784200
O	1.58473800	2.66022300	-0.18592000
H	1.53294700	3.26524600	-0.93443700
H	1.87307100	3.19537600	0.56218900
C	0.88207100	-0.39633900	-0.21227300
H	0.97363900	-0.38371600	-1.31052700
H	1.81376000	-0.00099200	0.21733900
O	0.50008300	-1.62625200	0.29562500
C	1.46589300	-2.65408400	0.06441700
H	2.41576800	-2.40852300	0.55156800
H	1.05265600	-3.55995800	0.50330100
H	1.62250900	-2.80234000	-1.00991200
C	-2.36834800	-0.42811400	-0.73374600

H	-2.56240100	0.35140500	-1.47324300
H	-1.76791900	-1.22642900	-1.17189700
H	-3.33283000	-0.85212500	-0.43461900

*

ts2-6bbeckm

C	-0.25264300	0.40414100	-0.49937800
N	0.48474400	0.63207000	-1.40795100
C	-1.39606400	0.08746600	-1.71858500
H	-2.16468300	0.68631100	-1.21368000
H	-1.18132100	0.54805800	-2.68401200
O	2.24406100	1.06880600	0.25997200
H	2.93329700	0.37465400	0.25218200
H	2.70327900	1.90524000	0.14227000
O	4.05551400	-1.00350300	0.28378600
H	4.58529000	-1.27469700	-0.47155900
H	4.56714100	-1.22220200	1.06822500
C	-0.56809200	0.27037300	0.95350400
H	-0.05959600	-0.64171300	1.30258300
H	-0.09213100	1.13479000	1.43790700
O	-1.94755000	0.22580900	1.08956100
C	-2.37265600	0.18651200	2.45257100
H	-2.04988900	1.08901300	2.98313000
H	-3.45981100	0.14328900	2.42932200
H	-1.97867300	-0.70445000	2.95412700
C	-1.61342700	-1.40139200	-1.77200200
H	-0.76541900	-1.91153300	-2.23263300
H	-1.81869100	-1.82342400	-0.78709100
H	-2.49275500	-1.57037700	-2.40284900

*

ts2-5beckm

C	-0.18435200	0.93505300	-0.02835800
N	-0.18364900	2.06925100	0.36572900
C	-1.76362000	0.84202500	0.51777500
H	-1.62524600	0.02307500	1.23072100
H	-2.07750700	1.71935100	1.09153000
O	2.01691600	2.58892700	-0.17793700
H	2.06707800	3.36607700	-0.74607500
H	2.50656900	2.81585200	0.62116400
C	0.58308800	-0.12958500	-0.71858000
H	0.14281500	-0.30657500	-1.70396500
H	1.59963900	0.25599300	-0.83391400
C	0.59922900	-1.43819200	0.07221200
F	1.43802100	-2.29319500	-0.49424500
F	-0.62600400	-1.99201700	0.10534800
F	0.97880200	-1.21733100	1.33849500
C	-2.64513600	0.59289700	-0.68579300
H	-2.65574500	1.44926000	-1.36346900
H	-2.36788500	-0.31227600	-1.22737500
H	-3.66221500	0.46017200	-0.30265400

*

ts2-5bbeckm

C	0.18243400	0.27753900	-0.60313900
N	1.00758600	0.84926900	-1.24673400
C	-0.69767200	0.38585500	-2.08434700
H	-1.57025800	0.87078900	-1.63633700
H	-0.28626300	1.08062700	-2.82016100
O	2.49151100	0.88722600	0.72385500
H	3.28872600	0.32670900	0.62749800
H	2.80662200	1.76886500	0.94333100
O	4.62778000	-0.81410800	0.46874700
H	5.31364900	-0.75216700	-0.20243400
H	5.03623200	-1.21850400	1.24000700
C	-0.25313000	-0.38013300	0.64504100
H	-0.20823500	-1.46483400	0.51275300
H	0.48838800	-0.07219900	1.39017300
C	-1.65960200	0.02661600	1.07788300
F	-1.91573900	-0.46218300	2.28297000
F	-1.77976100	1.36099400	1.10562700
F	-2.58215000	-0.44521500	0.21772100
C	-0.85154100	-1.01846400	-2.60957500
H	0.09373100	-1.40943800	-2.99098800
H	-1.27145600	-1.70381900	-1.87222900
H	-1.55300800	-0.95987900	-3.44884400

*

ts2-4beckm

C	-0.05661800	0.58931000	0.08005400
N	0.11469600	1.75324200	0.32764900
C	-1.65286300	0.82552500	0.53039200
H	-1.69074300	0.12043300	1.36618500
H	-1.85608400	1.81892500	0.93892400
O	2.36035600	1.88540800	-0.17366500
H	2.52317600	2.51740400	-0.88313300
H	2.85033000	2.21479900	0.58842400
C	0.50456300	-0.68757200	-0.41487100
H	0.06479800	-0.87999400	-1.39947100
H	1.57714500	-0.51580100	-0.53697000
C	0.23005300	-1.87598300	0.51630500
H	-0.84213900	-2.05457200	0.64719000
H	0.70669500	-1.73921700	1.49109700
F	0.77710100	-2.97978500	-0.08935500
C	-2.50487500	0.51853800	-0.68289000
H	-2.34207400	1.24707200	-1.48005500
H	-2.34542900	-0.49015700	-1.06764100
H	-3.54931100	0.60253400	-0.36605200

*

ts2-4bbeckm

C	0.10887700	0.24368100	-0.91657300
N	1.17033600	0.74195200	-1.16068500
C	-0.09534800	0.23991900	-2.59939100
H	-1.03998400	0.79307400	-2.60298900
H	0.63225000	0.83550600	-3.15519200
O	1.81412400	0.96291200	1.12467000
H	1.82468100	0.16802500	1.68780400
H	2.66381300	1.40312300	1.21753700
O	1.05226700	-1.33736300	2.47819200
H	1.56990900	-2.12501400	2.67421600
H	0.51918300	-1.16851700	3.26299800
C	-0.87099800	-0.26165400	0.06424400
H	-1.70686400	-0.74467100	-0.44483200
H	-0.33340200	-0.99734100	0.67684100
C	-1.36063300	0.87477600	0.98366100
H	-2.01161000	1.57406600	0.45286200
H	-0.51461400	1.39749800	1.43337900
F	-2.09693800	0.26558400	1.97871700
C	-0.12456400	-1.20527600	-3.03746700
H	0.86281900	-1.66359300	-2.95468800
H	-0.85225900	-1.80208300	-2.48440700
H	-0.41026900	-1.21300100	-4.09446700

*

ts2-3beckm

C	-0.19164700	0.49179000	0.05936600
N	-0.61935900	1.55828000	0.42000700
C	-1.54541700	-0.25690600	0.62812400
H	-1.04023600	-0.96474900	1.29643600
H	-2.17954900	0.38880200	1.23992200
O	1.09778000	2.90995900	-0.19046600
H	0.77057500	3.57351700	-0.80877000
H	1.38060300	3.39627500	0.59264400
C	0.94006200	-0.25026800	-0.58042900
H	0.56891000	-0.63871600	-1.53431100
H	1.69935300	0.51147000	-0.78016600
C	1.44129600	-1.35713000	0.31022300
C	1.31331100	-2.64785300	0.00956400
H	1.95088000	-1.04916600	1.22013200
H	1.71367600	-3.41442100	0.66413700
H	0.83259200	-2.98404400	-0.90590500
C	-2.28434400	-0.85094900	-0.55140000
H	-2.67340400	-0.07519100	-1.21437200
H	-1.66575300	-1.54562900	-1.12189300
H	-3.13801700	-1.40570900	-0.15012500

*

ts2-3bbeckm

C	-0.25054000	0.31450000	-0.22560100
N	0.55852100	0.95589300	-0.82690000
C	-1.22621400	0.61220500	-1.59074700
H	-2.06707800	0.99845300	-1.00199000
H	-0.88734300	1.41032700	-2.25274800
O	2.17851700	0.74733300	0.96104100
H	2.95281300	0.23203400	0.65707600
H	2.52214400	1.59422800	1.25955900
O	4.28963100	-0.79621300	0.10274900
H	4.88181700	-0.57925900	-0.62297600
H	4.79201200	-1.34568500	0.71139800
C	-0.71788200	-0.45813800	0.95477300
H	-0.85391400	-1.49627900	0.63581300
H	0.12875200	-0.40753100	1.64802000
C	-1.98572000	0.12110400	1.52943400
C	-3.15716400	-0.50981500	1.48733200
H	-1.89297200	1.08943700	2.01499200
H	-4.04224500	-0.07308600	1.93698000
H	-3.26958100	-1.48945300	1.02912300
C	-1.42674700	-0.70551900	-2.29717700
H	-0.50680400	-1.04453700	-2.77742400
H	-1.80333600	-1.48464900	-1.63224200
H	-2.17390100	-0.54004900	-3.08020500

*

ts2-2beckm

C	0.12711700	0.14522900	0.06692300
N	0.75927000	1.12616800	0.36997600
C	-1.24778300	0.89146900	0.59336300
H	-1.53517000	0.17571400	1.37015800
H	-1.06619900	1.85064600	1.08489100
O	2.84435200	0.45878000	-0.19214400
H	3.20369400	1.06636600	-0.84899700
H	3.40287700	0.55356700	0.58819700
C	0.16094700	-1.22397100	-0.51623900
H	-0.27494300	-1.14000700	-1.51783700
H	1.22275600	-1.45655100	-0.62797000
C	-0.58376600	-2.25917100	0.32861800
H	-1.65421400	-2.04768000	0.39247700
H	-0.46954100	-3.23531800	-0.14564600
H	-0.16983100	-2.32718000	1.33712400
C	-2.17977900	1.02622000	-0.59390300
H	-1.78044100	1.71683400	-1.33984200
H	-2.40032900	0.06636800	-1.06406600
H	-3.11758300	1.44764000	-0.21896400

*

ts2-2bbeckm

C	-0.56807500	0.28354500	0.03096300
N	0.07382900	0.96516000	-0.71216300
C	-1.78503000	0.53339400	-1.14439400
H	-2.52084500	0.94296700	-0.44537700
H	-1.58048000	1.30403700	-1.88962600
O	2.02099900	0.83159000	0.72281700
H	2.72919600	0.31345700	0.29041000
H	2.40092800	1.69180800	0.92284900
O	3.92955600	-0.74681000	-0.48366500
H	4.38171600	-0.54516900	-1.30795800
H	4.53740200	-1.27810000	0.03890500
C	-0.74385800	-0.53116500	1.25352200
H	-0.67842700	-1.57954700	0.94294000
H	0.15078600	-0.30022000	1.84026500
C	-2.04413400	-0.24335700	2.00492100
H	-2.92578200	-0.53853800	1.42968100
H	-2.04516700	-0.82498200	2.92809800
H	-2.12735500	0.81175100	2.27508700
C	-2.07605800	-0.81516800	-1.75833700
H	-1.24257000	-1.15753000	-2.37493000
H	-2.31785500	-1.57480200	-1.01290000
H	-2.94597100	-0.68998700	-2.41103500

*

ts2-1beckm

C	0.04050000	-0.19452800	0.22231800
N	0.53805700	0.89742500	0.12316500
C	-1.41909300	0.55974100	0.49242000
H	-1.63160200	0.13126500	1.47695500
H	-1.33654900	1.64234000	0.61893900
O	2.69727100	0.29270200	-0.25913200
H	2.99363500	0.64902200	-1.10465700
H	3.26009900	0.69963800	0.40974400
C	0.22855400	-1.65714500	0.18687300
H	1.29934300	-1.84900800	0.10842700
H	-0.18703200	-2.10263300	1.09326100
H	-0.29222100	-2.06389500	-0.68332200
C	-2.34622900	0.18430000	-0.64421400
H	-2.00073100	0.59864400	-1.59385700
H	-2.48100700	-0.89416200	-0.73941400
H	-3.32036700	0.63152500	-0.42310000

*

ts2-1bbeckm

C	-0.81342500	0.13299400	0.50762200
N	-0.31327900	0.98643500	-0.16172000
C	-2.21324000	0.58721300	-0.38948700
H	-2.83100400	0.78518600	0.49162000
H	-2.13674900	1.52118000	-0.94892300
O	1.84090900	0.62380500	0.87602500
H	2.48427500	0.25899800	0.23516400

H	2.23827300	1.42507600	1.22898200
O	3.57502500	-0.52371600	-0.92587000
H	3.89313900	-0.11807300	-1.73755700
H	4.26066300	-1.13359100	-0.63799800
C	-0.78421000	-0.92358200	1.52589500
H	-1.56236500	-0.74771800	2.27143900
H	-0.94454600	-1.89322900	1.04928500
H	0.21086500	-0.88049300	1.97419900
C	-2.58057700	-0.59122300	-1.25769500
H	-1.84911100	-0.73747600	-2.05507900
H	-2.69917200	-1.51677300	-0.69228400
H	-3.54206400	-0.35579000	-1.72553400

*

ts2-10beckm

C	0.09497900	2.01240600	0.19944600
N	0.95805400	2.91447000	0.19309000
C	-1.16180100	2.96755900	0.22878600
H	-1.68238400	2.67560200	1.14635700
H	-0.85376500	4.00767500	0.37191500
O	2.74427700	2.14618700	0.18621300
H	3.19966300	2.86005000	-0.28245500
H	3.02311800	2.20436600	1.11156500
C	-1.97372800	2.81575000	-1.05126600
H	-1.38791800	3.11893600	-1.92209700
H	-2.32258100	1.79292900	-1.20147400
H	-2.84310900	3.47490200	-0.98502000
C	-0.01765400	0.57125100	0.16835800
C	-1.20366100	-0.04728600	0.62048200
C	1.02136900	-0.23191400	-0.33268500
C	-1.33159000	-1.41543300	0.59551400
H	-2.02798200	0.54137000	1.00788600
C	0.89030600	-1.60882300	-0.37877100
H	1.92882400	0.22240900	-0.70707000
C	-0.28587000	-2.21500000	0.09368700
H	-2.23070300	-1.90265500	0.95400900
H	1.70067800	-2.20129900	-0.78405600
O	-0.50945300	-3.52529100	0.10374000
C	0.48318000	-4.41948600	-0.39636500
H	1.40902400	-4.33461600	0.18064900
H	0.06361000	-5.41531300	-0.27126300
H	0.67399300	-4.23125900	-1.45729700

*

ts2-10bbeckm

C	1.38380900	0.45990600	-0.83270600
N	2.53214000	0.67436900	-0.50556500
C	2.07267500	0.31738600	-2.35943200
H	1.39051800	1.01329400	-2.85907500
H	3.06951700	0.75111400	-2.46239800
O	2.42145600	0.91125800	1.82909400
H	3.10077100	0.28436600	2.15083700
H	2.74365400	1.79074700	2.04822600
O	4.26948300	-0.90817000	2.75740500
H	5.20052100	-0.95320400	2.52283000
H	4.18408400	-1.31027200	3.62654200
C	2.03278600	-1.13643600	-2.77512400
H	2.76241200	-1.71755900	-2.20811500
H	1.04683700	-1.58632100	-2.65197800
H	2.30738800	-1.18588700	-3.83328200
C	-0.00737700	0.28004300	-0.55429800
C	-0.41428800	0.23321300	0.79124800
C	-0.96286800	0.16307900	-1.58538600
C	-1.75187900	0.06980100	1.10356100
H	0.33013500	0.33582500	1.57370700
C	-2.29129500	0.00401800	-1.27428300
H	-0.67131600	0.19807700	-2.63002100
C	-2.70158300	-0.04684800	0.07447100
H	-2.04782400	0.03656400	2.14431400
H	-3.04487600	-0.08411400	-2.04786400
O	-4.00703400	-0.20764100	0.26648700
C	-4.52635500	-0.27432000	1.59372300
H	-4.33461600	0.65937800	2.13094800
H	-5.59843900	-0.41660900	1.47693700
H	-4.09842600	-1.12412800	2.13393800

*

ts2-0beckm

C	0.15263200	-0.08138000	-0.50359100
N	0.45379300	0.57970200	0.43552500
C	-1.42778900	0.50838400	-0.19192100
H	-1.57047600	0.92758600	-1.19338100
H	-1.47071800	1.33663100	0.52038300
H	0.37659800	-0.77301100	-1.30574100
O	2.79370600	-0.23684100	0.02608900
H	3.46290700	0.40170400	-0.24292800
H	3.21918100	-0.78804100	0.69166000
C	-2.25088000	-0.68486600	0.22202600
H	-2.19973600	-1.50651100	-0.49415300
H	-1.97295500	-1.03883500	1.21670400
H	-3.28887300	-0.33601900	0.27220200

*

ts2-0bbeckm

C	-0.75673600	-0.31287900	0.08439100
N	-0.49576200	0.76201400	-0.34447100
C	-2.23400500	-0.07292600	-0.76180500
H	-2.16129600	-1.01278000	-1.31908900
H	-2.22296000	0.73966100	-1.49212400
H	-0.54062900	-1.19919100	0.66684600
O	1.60860700	0.19372100	0.96665400
H	2.40559100	0.04580500	0.41690900
H	1.90504100	0.70124600	1.72795300
O	3.77473700	-0.27434800	-0.65512700
H	4.21370000	0.40102100	-1.18031100
H	4.42884100	-0.95848200	-0.48541400
C	-3.31071700	0.06703500	0.28338500
H	-3.29864300	-0.73974300	1.01814800
H	-3.24768600	1.03093300	0.79176800
H	-4.26750800	0.03140900	-0.24956500

*

ts1-9beckm

C	-0.37712400	1.25725700	-0.11660100
N	-0.00913100	2.37268500	0.17299100
C	-1.59758300	2.08568800	-0.85625100
H	-1.46502800	1.78946000	-1.89751600
H	-2.47175000	1.69390500	-0.33442100
H	-1.60534900	3.17682500	-0.79242900
O	1.98417700	2.02677600	1.40909000
H	2.70397000	2.50183000	0.97889000
H	1.89102200	2.42584500	2.28141900
C	-0.24751400	-0.18579100	-0.10619600
C	-1.19742300	-0.99632800	-0.73350800

C	0.84997900	-0.75693500	0.54658000
C	-0.99979800	-2.37299900	-0.67519700
H	-2.06249500	-0.59449000	-1.24904900
C	0.93654300	-2.14746500	0.53274800
H	1.59389000	-0.14122000	1.03843200
N	0.03999900	-2.93749300	-0.05912300
H	-1.70874400	-3.04827500	-1.14395100
H	1.76845300	-2.64421400	1.02241300

*

ts1-9bbeckm

C	0.27310000	0.11417500	-1.17780000
N	1.40445200	0.26156400	-1.54223900
C	0.25998800	-0.23996800	-2.87421000
H	-0.11133200	-1.26337900	-2.81997000
H	-0.44264900	0.52634200	-3.20068400
H	1.15156700	-0.20934800	-3.50148200
O	2.29778700	0.89812700	0.76351100
H	2.99065900	0.26062900	1.02685900
H	2.70302300	1.76779100	0.82373600
O	4.18917600	-0.96277800	1.53087800
H	5.03584900	-1.11887600	1.10314600
H	4.28988800	-1.23847600	2.44654800
C	-0.88950200	0.05679900	-0.32778600
C	-0.71408900	0.31787500	1.03554500
C	-2.15452400	-0.24935500	-0.83921500
C	-1.85488100	0.25415200	1.83406600
H	0.26701500	0.55822100	1.43258100
C	-3.21239800	-0.27929400	0.06367500
H	-2.32954400	-0.45612400	-1.88879400
N	-3.06991200	-0.03523900	1.36785600
H	-1.78020300	0.44565000	2.89997900
H	-4.21675700	-0.50919200	-0.27803700

*

ts1-8beckm

C	-0.39618400	1.25481800	-0.01136000
N	0.09171000	2.36911900	-0.09806600
C	-1.81121500	2.04053800	0.15008500
H	-2.38388800	1.67816100	-0.70455000
H	-2.15288300	1.71997900	1.13514700
H	-1.78373300	3.13282100	0.12177100
O	2.33390900	2.09578600	-0.37727900
H	2.55561100	2.57397600	-1.18477500
H	2.75141000	2.58321600	0.34239100
C	-0.25389600	-0.17992400	0.00045400
C	1.02840200	-0.73289100	-0.16674300
C	-1.38212500	-0.99741800	0.17495500
C	1.16503500	-2.11301700	-0.15651700
H	1.88457500	-0.08220500	-0.30001400
C	-1.22324000	-2.37436700	0.18283900
H	-2.37138300	-0.57137900	0.30419100
C	0.04669300	-2.92927100	0.01727200
H	2.14667300	-2.55469000	-0.28503800
H	-2.08650200	-3.01588400	0.31722900
H	0.16539200	-4.00764600	0.02418000

*

ts1-8bbeckm

C	0.27591500	0.12896100	-1.15329600
N	1.42438800	0.29427900	-1.47068300
C	0.28928100	-0.16630400	-2.83925400
H	-0.07316300	-1.19423700	-2.83970800
H	-0.41171700	0.60387500	-3.16038300
H	1.19723000	-0.10232800	-3.43983600
O	2.31593600	0.87940400	0.82183200
H	3.02283900	0.24640200	1.05472700
H	2.71923400	1.75108800	0.86105200
O	4.27163200	-0.95181100	1.51605800
H	5.08740300	-1.12553300	1.03834200
H	4.42507900	-1.21596600	2.42762100
C	-0.89086300	0.04858500	-0.32483000
C	-2.14935000	-0.24881100	-0.87237300
C	-0.71941500	0.28223200	1.05169400
C	-3.24744800	-0.30979000	-0.02920700
H	-2.27560100	-0.42819400	-1.93467600
C	-1.83469700	0.21279700	1.87447000
H	0.26984200	0.51159200	1.43570600
C	-3.08885100	-0.07988900	1.33893000
H	-4.22634300	-0.53499700	-0.43632200
H	-1.72324300	0.38892400	2.93825800
H	-3.95314600	-0.12918900	1.99289700

*

ts1-6beckm

C	-0.46423200	0.65859800	-0.06046300
N	-0.64649900	1.83190200	0.08468600
C	-2.10338500	0.57510300	0.16966700
H	-2.44567700	0.20531000	-0.79654100
H	-2.10954100	-0.14217600	0.99338400
H	-2.64619100	1.48003600	0.45246900
O	1.79129900	2.22296800	-0.20227300
H	2.04465600	2.75547700	-0.96369100
H	2.19917000	2.65170800	0.55768500
C	0.39212700	-0.53941800	-0.32061600
H	0.61035000	-0.53477600	-1.40138500
H	1.33151200	-0.35981500	0.22236300
O	-0.31498700	-1.65162800	0.10071800
C	0.40506100	-2.87544000	-0.07609400
H	1.33434600	-2.86320900	0.50345100
H	-0.24862600	-3.66284700	0.29322600
H	0.62339600	-3.04324500	-1.13641000

*

ts1-6bbeckm

C	-0.27594800	0.18683500	-0.93240300
N	0.53940200	0.38094300	-1.78016700
C	-1.17309000	-0.19495900	-2.31244400
H	-1.42328400	-1.22842900	-2.07338500
H	-1.97329800	0.53704100	-2.20533800
H	-0.72531500	-0.12860800	-3.30575600
O	1.91907100	1.00475000	0.33179900
H	2.60804600	0.35701500	0.58011900
H	2.34838900	1.86462600	0.33019700
O	3.70307900	-0.97294600	1.03285500
H	4.37742100	-1.31689300	0.43962600
H	4.04723700	-1.07701800	1.92489500
C	-0.83572800	0.11053100	0.44891600
H	-0.15707000	-0.54691400	1.01291300
H	-0.74723900	1.12432100	0.86785000
O	-2.13553900	-0.35975200	0.35225500
C	-2.77850200	-0.47146800	1.62379700
H	-2.86589200	0.51144300	2.09971700
H	-3.77022900	-0.87373000	1.42737000

H	-2.22550000	-1.15636900	2.27606600

*

ts1-5beckm

C	-0.54866800	0.98688200	-0.21191600
N	-0.77550000	2.10299500	0.16027900
C	-2.17836100	0.80767600	0.10213100
H	-2.54260900	0.50812300	-0.88160300
H	-2.16049100	0.04638300	0.88226700
H	-2.74638500	1.67507500	0.45085000
O	1.56030600	2.77298400	-0.06936400
H	1.73430500	3.47978700	-0.70058200
H	1.93790200	3.07285100	0.76497900
C	0.39012900	-0.02377000	-0.74464500
H	0.20650500	-0.14184100	-1.81741900
H	1.39079400	0.38989500	-0.58751500
C	0.24384200	-1.38064400	-0.05310100
F	1.20540300	-2.19479500	-0.45802100
F	-0.94650600	-1.93230000	-0.34221400
F	0.31339100	-1.23538600	1.27807300

*

ts1-5bbeckm

C	0.21432800	-0.00834800	-0.91473000
N	1.01441000	0.47060600	-1.65885100
C	-0.55570500	-0.20348200	-2.42546300
H	-0.66815600	-1.28787600	-2.42982900
H	-1.45347400	0.39054000	-2.25658700
H	-0.06782300	0.11890000	-3.34802600
O	2.36166900	0.96687700	0.44319000
H	3.15820600	0.41455700	0.58083100
H	2.65054600	1.88037000	0.52340400
O	4.47750900	-0.73615300	0.83178600
H	5.21233000	-0.84330000	0.22061000
H	4.82137200	-0.93382000	1.70814200
C	-0.27561800	-0.43860000	0.40681700
H	-0.09077100	-1.51158000	0.51874200
H	0.34566700	0.11511200	1.11998400
C	-1.76105500	-0.14797200	0.61590900
F	-2.09341300	-0.39863700	1.87290300
F	-2.03435400	1.13357700	0.33055200
F	-2.51286700	-0.91403200	-0.19384800

*

ts1-4beckm

C	-0.50286000	0.61155800	-0.11960400
N	-0.57509500	1.77431900	0.17091300
C	-2.16179300	0.70351600	0.02577000
H	-2.46756200	0.38617500	-0.97244700
H	-2.35614900	0.00904700	0.84317700
H	-2.62295600	1.66788800	0.25265900
O	1.78298300	2.14851600	0.06250800
H	2.04590500	2.77655300	-0.61932600
H	2.15552000	2.48639300	0.88433000
C	0.26956800	-0.58784600	-0.49757600
H	0.04359600	-0.80825700	-1.54720700
H	1.32000100	-0.29448700	-0.41282600
C	-0.04072800	-1.81723900	0.37155700
H	-1.08500100	-2.13026100	0.27525900
H	0.20208400	-1.63607400	1.42245300
F	0.75712300	-2.82924300	-0.09702400

*

ts1-4bbeckm

C	-0.34249600	-0.13942200	-1.23774900
N	0.14519700	0.65758800	-1.98718200
C	-1.20160900	-0.52069600	-2.63122100
H	-0.91730100	-1.56511600	-2.76029800
H	-2.22081800	-0.32727700	-2.29610100
H	-1.01248800	0.02585100	-3.55770600
O	1.50890300	1.57139400	-0.16414500
H	2.09359300	1.01246500	0.37719700
H	1.99946900	2.36215400	-0.40483300
O	2.44596000	-0.49662800	1.44195000
H	3.29042600	-0.95595200	1.38861200
H	2.24602700	-0.41674800	2.38123100
C	-0.48581200	-0.77478100	0.08308800
H	-1.09102300	-1.67948300	0.00600100
H	0.53570700	-1.03181900	0.39815300
C	-1.07526900	0.21298900	1.11216700
H	-2.13542800	0.40627300	0.93039200
H	-0.50363400	1.14278000	1.12045700
F	-0.95105200	-0.40540100	2.33788100

*

ts1-3beckm

C	-0.37625400	0.49879100	-0.20505800
N	-0.65793400	1.62008400	0.11933800
C	-2.00935400	0.25871300	-0.20645300
H	-2.21169200	0.04138200	-1.25526100
H	-2.06292800	-0.58799400	0.48291300
H	-2.66238000	1.06022600	0.14470600
O	1.59775500	2.38460900	0.25096000
H	1.77987700	3.09956900	-0.36862500
H	1.81457200	2.73391100	1.12224900
C	0.61954400	-0.56913200	-0.50146600
H	0.45245200	-0.88904500	-1.53527200
H	1.58949400	-0.06689100	-0.42899600
C	0.48159700	-1.71889800	0.46654500
C	0.07033500	-2.93018900	0.09781900
H	0.76915400	-1.51722900	1.49530300
H	0.01556600	-3.74003600	0.81717100
H	-0.19666400	-3.16319300	-0.93004900

*

ts1-3bbeckm

C	-0.37871800	-0.15282800	-0.59255100
N	0.40522500	0.20433400	-1.42112900
C	-1.18377600	-0.65496000	-1.99059900
H	-1.17646200	-1.73747300	-1.87152000
H	-2.13349400	-0.15083000	-1.79441800
H	-0.82016900	-0.37644100	-2.98039400
O	1.84431800	1.05815000	0.41323200
H	2.67791800	0.54908400	0.46701300
H	2.10030700	1.98307100	0.35883700
O	4.11529600	-0.48872600	0.58868700
H	4.77991500	-0.58350100	-0.09957600
H	4.56352600	-0.64286900	1.42541200
C	-0.92998600	-0.30444600	0.77482800
H	-1.06849300	-1.37439100	0.96033800
H	-0.12989100	0.08029000	1.41725500
C	-2.22217300	0.46337300	0.92228600
C	-3.39865500	-0.12843800	1.11413800
H	-2.13861800	1.54678600	0.89374800

H	-4.30120500	0.45793100	1.24813300
H	-3.50116300	-1.20928500	1.17262400

*

ts1-2beckm

C	-0.34941200	0.11188700	-0.14615500
N	-0.51097200	1.27603600	0.10702500
C	-1.94846200	-0.01370000	0.25576700
H	-2.37431100	-0.40014100	-0.67112100
H	-1.90507500	-0.70765800	1.09598600
H	-2.48674500	0.88803900	0.55645200
O	1.71376900	1.94026100	-0.33738700
H	1.76838500	2.59413400	-1.04293600
H	2.11916000	2.35357600	0.43283700
C	0.51490900	-1.01482000	-0.57310800
H	0.13068100	-1.35659900	-1.54045300
H	1.49710000	-0.55918000	-0.72683300
C	0.55057900	-2.15832800	0.44676800
H	-0.42315000	-2.64113800	0.55786200
H	1.25288800	-2.91165400	0.08642600
H	0.89885200	-1.81365700	1.42267400

*

ts1-2bbeckm

C	-0.82246200	0.00927800	-0.15668200
N	-0.38538400	0.61222100	-1.09249400
C	-2.13683300	-0.06477400	-1.22652300
H	-2.23667500	-1.14550400	-1.32853200
H	-2.89425700	0.44133100	-0.62812000
H	-2.08595800	0.39352800	-2.21528400
O	1.71639400	0.95230400	0.16655600
H	2.46319800	0.43524400	-0.19630000
H	2.01500900	1.86530200	0.20156900
O	3.74349900	-0.63277000	-0.81547700
H	4.10751500	-0.58762400	-1.70432900
H	4.44786200	-0.95934900	-0.24795700
C	-0.79527000	-0.58550800	1.19383200
H	-0.70952500	-1.66915800	1.05608100
H	0.14687000	-0.21494900	1.61063000
C	-2.01198300	-0.21879200	2.04868300
H	-2.93842000	-0.63088800	1.64128500
H	-1.87065000	-0.64695400	3.04214300
H	-2.11340000	0.86272400	2.16044400

*

ts1-1beckm

C	-0.37756800	-0.26717600	-0.02173700
N	-0.12033500	0.90468900	0.03833000
C	-1.94217100	0.28716100	-0.10470700
H	-2.25780200	-0.11226100	-1.06919300
H	-2.37815600	-0.17487900	0.78170400
H	-2.12293900	1.36438900	-0.07947000
O	2.22858600	0.61613500	0.19471500
H	2.70399500	1.04552500	-0.52503200
H	2.59498400	0.98526600	1.00587800
C	0.02237700	-1.68237400	-0.04639200
H	1.11084400	-1.71135600	0.02698400
H	-0.43930600	-2.20217500	0.79675400
H	-0.31912400	-2.13823000	-0.97898800

*

ts1-1bbeckm

C	-1.25825100	-0.03095200	0.11938700
N	-0.76441800	0.44396600	-0.85984200
C	-2.49893300	-0.26367800	-1.02550600
H	-2.58534400	-1.34945700	-0.98486600
H	-3.29828500	0.30849300	-0.55491700
H	-2.37896700	0.05560100	-2.06194600
O	1.25415300	0.93590000	0.47864800
H	2.02555400	0.42632100	0.15775000
H	1.53480900	1.85505700	0.50353400
O	3.36048500	-0.59905400	-0.40011200
H	3.75301400	-0.55438100	-1.27675700
H	4.05078500	-0.90827200	0.19376200
C	-1.32578700	-0.43352500	1.52632900
H	-2.17247600	0.06002500	2.00921000
H	-1.44907800	-1.51718100	1.59230300
H	-0.37939500	-0.11863300	1.97225000

*

ts1-10beckm

C	-0.38827500	2.18476100	-0.00138800
N	0.23056400	3.24991700	-0.00258300
C	-1.74472500	3.04072000	-0.00099900
H	-2.24351500	2.74184700	-0.92380800
H	-2.24213900	2.74330900	0.92302200
H	-1.62202800	4.12692700	-0.00195500
O	2.37503900	2.81542300	-0.00376600
H	2.68898800	3.30368900	-0.77469800
H	2.69006100	3.30324300	0.76701200
C	-0.34303000	0.75477100	-0.00047600
C	0.89987000	0.09296000	-0.00144700
C	-1.53788000	0.00109800	0.00130600
C	0.95240500	-1.28727000	-0.00062800
H	1.81665900	0.67001300	-0.00285500
C	-1.48705500	-1.37085600	0.00217500
H	-2.50666700	0.48927600	0.00205800
C	-0.24126200	-2.03296100	0.00124000
H	1.91713300	-1.77804100	-0.00147900
H	-2.39106200	-1.96837100	0.00356000
O	-0.29982100	-3.35801700	0.00225000
C	0.90450600	-4.12565900	0.00144900
H	1.49294600	-3.91967200	0.90038200
H	0.58452800	-5.16527300	0.00297600
H	1.49050600	-3.92154100	-0.89950100

*

ts1-10bbeckm

C	1.43998000	0.17353200	-1.28928500
N	2.63132700	0.37191400	-1.24622200
C	1.93944400	-0.11850300	-2.88365000
H	1.63522400	-1.15949900	-2.99058700
H	1.33677000	0.62531300	-3.40482000
H	2.98052400	-0.01421300	-3.19115600
O	2.80407700	0.95516000	1.19623600
H	3.39534900	0.29951300	1.61389400
H	3.23587200	1.80715000	1.30358100
O	4.42705900	-0.94879400	2.38831100
H	5.30320600	-1.21158000	2.09312700
H	4.37549200	-1.16508300	3.32353400
C	0.09706400	0.06926200	-0.83679500
C	-0.95183800	-0.30630600	-1.70439500
C	-0.16450700	0.35558700	0.51735000
C	-2.23403300	-0.39269200	-1.22207300

H	-0.76448000	-0.52757200	-2.74981900
C	-1.45723000	0.26894100	0.99763600
H	0.65758200	0.64221200	1.16633400
C	-2.50219800	-0.10579000	0.13373100
H	-3.05926700	-0.67841900	-1.86351300
H	-1.64728800	0.49137300	2.03980400
O	-3.77384400	-0.21791200	0.49731800
C	-4.15584800	0.06384500	1.84375900
H	-3.93522600	1.10532500	2.09614600
H	-5.23015400	-0.10257400	1.88086900
H	-3.65397500	-0.61597900	2.53854700

*

ts1-0beckm

C	-0.21612900	-0.35230200	-0.03845100
N	-0.05389400	0.82077200	0.07658600
C	-1.82057900	0.12967600	0.03431900
H	-2.17829100	-0.22082600	-0.93476200
H	-2.14783200	-0.40596400	0.92639000
H	-2.05459200	1.19339500	0.14487000
H	0.13394700	-1.37259300	-0.14621500
O	2.40153200	-0.02747200	-0.02806300
H	2.96343700	0.19147800	-0.77932700
H	2.97240200	0.04383400	0.74465400

*

ts1-0bbeckm

C	-1.25109600	-0.16831700	0.28287800
N	-1.08846200	0.94177900	-0.11302000
C	-2.73136200	0.05927300	-0.47103000
H	-2.69875700	-0.73542400	-1.21697800
H	-3.40820100	-0.06464400	0.37500500
H	-2.94108200	1.01224100	-0.96585500
H	-0.92827800	-1.06399500	0.80158900
O	1.10955900	0.23240700	1.10883100
H	1.88342900	0.06837800	0.53008200
H	1.45619600	0.65639800	1.89963600
O	3.17237700	-0.29823700	-0.61314500
H	3.62145900	0.37122500	-1.13764400
H	3.80421700	-1.01108400	-0.48035000

*

ts0-9beckm

C	-1.05651200	1.19363300	0.00062600
N	-1.12381800	2.40366000	0.00079400
O	0.93802400	3.03957100	-0.00062900
H	1.07107100	3.60523000	-0.77236000
H	1.07224100	3.60480800	0.77120900
C	-0.46151400	-0.11741900	0.00011100
C	-1.26400300	-1.26236400	0.00090200
C	0.93566300	-0.21507200	-0.00118700
C	-0.60786800	-2.48996000	0.00034900
H	-2.34742600	-1.21219700	0.00193300
C	1.46757700	-1.50219500	-0.00163700
H	1.56752600	0.66517600	-0.00181300
N	0.72101300	-2.60791600	-0.00090000
H	-1.17509400	-3.41536100	0.00092500
H	2.54354400	-1.64642000	-0.00267600
H	-2.21994200	1.45840400	0.00157900

*

ts0-9bbeckm

C	0.28230200	0.08909400	-1.36797800
N	1.40716800	0.27447900	-1.76273100
O	2.38458600	0.94404800	0.08344300
H	3.09735100	0.29732900	0.29685000
H	2.81982500	1.78958900	-0.07180500
O	4.25746000	-0.89718000	0.69475300
H	4.98336800	-1.18576100	0.13378600
H	4.50936800	-1.09876300	1.60091000
C	-0.86518500	0.01110900	-0.50669800
C	-0.70184100	0.38865100	0.83171500
C	-2.10138900	-0.42792600	-0.98951700
C	-1.83282200	0.30129000	1.64051800
H	0.25570700	0.73045800	1.20780800
C	-3.15086100	-0.47034200	-0.07662800
H	-2.25071900	-0.72321800	-2.02217700
N	-3.02191000	-0.11609000	1.20347100
H	-1.77394300	0.57932500	2.68825300
H	-4.13554500	-0.80337500	-0.38918900
H	0.38052000	-0.16470600	-2.55937400

*

ts0-8beckm

C	-1.05504800	1.20048600	0.00295300
N	-1.11480700	2.41622700	0.00374900
O	0.93308800	3.05783500	0.00167700
H	1.04258500	3.62826300	-0.76999600
H	1.04425900	3.62735300	0.77378400
C	-0.46457900	-0.10106800	0.00144000
C	0.94043200	-0.19380700	-0.00035000
C	-1.28606900	-1.24148500	0.00169600
C	1.51338400	-1.45554300	-0.00192500
H	1.54653000	0.70501600	-0.00048300
C	-0.68780400	-2.49094900	0.00010200
H	-2.36756500	-1.14872000	0.00313200
C	0.70490300	-2.59479600	-0.00171500
H	2.59289900	-1.55438700	-0.00336700
H	-1.30214300	-3.38388800	0.00025700
H	1.16695500	-3.57629700	-0.00301300
H	-2.21473200	1.44643900	0.00457200

*

ts0-8bbeckm

C	0.28714400	0.09026200	-1.34760900
N	1.42127200	0.27315700	-1.73086300
O	2.40506300	0.92691800	0.10853200
H	3.13006200	0.28735300	0.29568700
H	2.82817800	1.77521900	-0.06341100
O	4.34377000	-0.87579600	0.66019600
H	5.02044600	-1.19824100	0.05799100
H	4.65848100	-1.04891600	1.55229900
C	-0.85861300	0.01275700	-0.50031700
C	-2.09960200	-0.39099200	-1.01997600
C	-0.68803200	0.35475000	0.85429000
C	-3.18727700	-0.45108600	-0.16388400
H	-2.20892000	-0.64810200	-2.06868900
C	-1.79367100	0.28562100	1.68821600
H	0.28672600	0.66307000	1.21629400
C	-3.03212500	-0.11452000	1.18271600
H	-4.15481300	-0.75942300	-0.54266200
H	-1.68969200	0.54280600	2.73609500
H	-3.88856600	-0.16353700	1.84693000
H	0.37301100	-0.15794600	-2.53189700

*

ts0-6beckm

C	-0.61235600	0.58034800	0.07255100
N	-1.02228400	1.70504600	0.14580800
O	0.86204600	2.80566700	-0.10638500
H	0.82722100	3.39290000	-0.87191900
H	1.04198000	3.37247000	0.65421600
C	0.34716800	-0.55789800	-0.07891900
H	0.81988100	-0.42779400	-1.06681800
H	1.11384300	-0.41373500	0.70070600
O	-0.39401300	-1.71596400	0.05339800
C	0.39705600	-2.90536200	-0.05333100
H	1.15406500	-2.93551000	0.73761000
H	-0.29795100	-3.73305000	0.06991800
H	0.87250000	-2.96471000	-1.03826300
H	-1.79617600	0.48891500	0.24024500

*

ts0-6bbeckm

C	-0.33134000	-0.29095800	-0.72118500
N	0.61339100	-0.39727600	-1.44606200
O	1.94132200	0.95292900	-0.29278100
H	2.70951800	0.45526700	0.07068000
H	2.30591400	1.67457300	-0.81637200
O	3.96264800	-0.49307300	0.76867100
H	4.59980400	-1.01774900	0.27484100
H	4.38043600	-0.26424900	1.60442100
C	-1.22144600	0.11735000	0.40350400
H	-0.84890600	-0.40380000	1.30070900
H	-1.05458200	1.19965700	0.53017400
O	-2.50849900	-0.23047500	0.03558300
C	-3.48712600	0.13576200	1.01367700
H	-3.49736700	1.22129100	1.16041200
H	-4.44385900	-0.18956200	0.61081100
H	-3.29286000	-0.37408100	1.96368100
H	-0.57917700	-1.05165000	-1.64923100

*

ts0-5beckm

C	-0.70836200	0.98148000	-0.30658400
N	-1.08836200	2.05208600	0.08221000
O	0.79038400	3.14181500	0.12881700
H	0.75028900	3.93188400	-0.42538200
H	0.98058000	3.45258800	1.02359700
C	0.24280100	-0.04451400	-0.77209100
H	0.05200400	-0.25086700	-1.82996100
H	1.24315300	0.38278500	-0.64665000
C	0.08974300	-1.33880300	0.04446900
F	0.94697400	-2.24267400	-0.39833300
F	-1.16053000	-1.80587900	-0.08185900
F	0.31670100	-1.09866500	1.33851700
H	-1.90025200	0.89510400	-0.13896000

*

ts0-5bbeckm

C	0.21645700	-0.39531600	-0.70923800
N	1.13760300	-0.20692300	-1.44961400
O	2.37620600	0.94667700	-0.04766400
H	3.21624700	0.46290600	0.13572800
H	2.62539600	1.80993300	-0.39599000
O	4.58708700	-0.48332200	0.50406900
H	5.26269700	-0.74762600	-0.12775300
H	5.01194000	-0.45281700	1.36682700
C	-0.62399800	-0.36603500	0.49573700
H	-0.60105400	-1.35212100	0.96973700
H	-0.16879100	0.37818500	1.15877500
C	-2.07257000	0.01239900	0.14986600
F	-2.80556900	0.00777200	1.25134500
F	-2.11664900	1.22183600	-0.41354600
F	-2.57349000	-0.87440500	-0.72351800
H	0.03483500	-0.91665600	-1.81478900

*

ts0-4beckm

C	-0.82803100	0.60777300	-0.27937100
N	-0.99255800	1.75611700	0.04700800
O	1.02756700	2.45227000	0.23635300
H	1.18506600	3.17927300	-0.38012400
H	1.16462500	2.81686000	1.12040900
C	-0.07369400	-0.61198100	-0.61685200
H	-0.42974900	-0.98299600	-1.58318600
H	0.96936700	-0.29093900	-0.71260900
C	-0.21651800	-1.71363300	0.45680600
H	-1.25535400	-2.03771000	0.56558800
H	0.18142100	-1.38225900	1.41932900
F	0.53079100	-2.76871200	0.00656600
H	-2.01327900	0.76613800	-0.23543800

*

ts0-4bbeckm

C	-0.70217800	-0.60387400	-1.20486900
N	-0.04325200	-0.21036700	-2.12942300
O	1.30908300	1.12102900	-1.04561800
H	1.89872100	0.56565300	-0.49724800
H	1.84985900	1.66104600	-1.63203500
O	2.17597300	-0.71806800	0.77503200
H	2.83225800	-1.40861000	0.63098000
H	2.34454900	-0.38116500	1.66245000
C	-1.12432200	-0.74689600	0.19532900
H	-2.00639200	-1.38606400	0.26486100
H	-0.27529100	-1.23282600	0.69566300
C	-1.37583000	0.63851200	0.83677500
H	-2.28495700	1.09905600	0.44351800
H	-0.51792500	1.29855400	0.69314500
F	-1.54472100	0.39552100	2.17783600
H	-1.11316000	-1.10716700	-2.22945800

*

ts0-3beckm

C	-0.72690600	0.41632500	-0.21779200
N	-1.08840900	1.48444300	0.20757800
O	0.74121100	2.61799900	0.16630300
H	0.62472900	3.39213100	-0.39930400
H	0.92248800	2.95864300	1.05161500
C	0.23982700	-0.60419400	-0.70123300
H	-0.14977900	-1.01546400	-1.63712800
H	1.15108900	-0.03086800	-0.91155000
C	0.45530100	-1.67691200	0.34472200
C	0.11759900	-2.94763400	0.14545300
H	0.94136300	-1.35951400	1.26300500
H	0.32207500	-3.69765100	0.90158200
H	-0.35240000	-3.28899900	-0.77317000
H	-1.88694200	0.27062900	0.00074300

*

ts0-3bbeckm

C	-0.44430800	-0.28889200	-0.66494500
N	0.47266000	0.03648700	-1.36938700
O	1.73628800	0.97779800	0.13437100
H	2.57387000	0.46426100	0.21233500
H	1.98374000	1.86809600	-0.13801300
O	3.96137500	-0.52203900	0.40258700
H	4.61305300	-0.71317200	-0.27844800
H	4.40933300	-0.61299600	1.24901200
C	-1.28459500	-0.41507200	0.54542400
H	-1.46014500	-1.48072300	0.72159900
H	-0.63760100	-0.02409200	1.34181600
C	-2.57579500	0.36200500	0.40706000
C	-3.76893100	-0.22387000	0.43272000
H	-2.47751200	1.44048400	0.32034200
H	-4.67616100	0.36661200	0.36691700
H	-3.88759100	-1.29911900	0.53768700
H	-0.67158100	-0.62672900	-1.81074500

*

ts0-2beckm

C	-0.69396800	-0.07703700	-0.21473600
N	-1.09955000	1.00051700	0.14389300
O	0.69077500	2.17493500	0.15773800
H	0.58507600	2.92299900	-0.44411100
H	0.80700800	2.55401000	1.03851000
C	0.30823900	-1.08513200	-0.62667000
H	0.00204400	-1.46348200	-1.60690200
H	1.23496000	-0.51315700	-0.74367600
C	0.44467600	-2.21829500	0.40262800
H	-0.48542400	-2.77976300	0.51007500
H	1.21451700	-2.90397200	0.04559200
H	0.75194300	-1.83580500	1.37729800
H	-1.86214500	-0.23597900	-0.05368100

*

ts0-2bbeckm

C	-0.95538700	-0.50332700	-0.51549500
N	-0.09850500	-0.21816700	-1.30831300
O	1.19322000	0.95507100	-0.02013100
H	2.06704300	0.50653400	0.06461300
H	1.36402500	1.81628000	-0.41679200
O	3.53189300	-0.34671400	0.29176600
H	4.14949200	-0.60474800	-0.39891000
H	4.03737100	-0.28501700	1.10787700
C	-1.70470000	-0.54939400	0.75257800
H	-1.88194600	-1.60156400	0.99395400
H	-0.99778800	-0.13573300	1.48127200
C	-3.01280500	0.25480300	0.68909400
H	-3.70616200	-0.15742000	-0.04657800
H	-3.48715600	0.19902000	1.66989600
H	-2.82193500	1.30422100	0.45942400
H	-1.22153900	-0.98223600	-1.60258300

*

ts0-1beckm

C	-0.78891200	0.36374100	0.00156500
N	0.16577200	1.09889200	0.00246200
O	1.79626100	-0.29184700	0.00047100
H	2.35524700	-0.13385800	-0.77140100
H	2.35526500	-0.13601000	0.77277000
C	-1.51105200	-0.91532700	0.00002000
H	-0.75342900	-1.70311800	-0.00216600
H	-2.13649400	-0.98019600	0.89372200
H	-2.13852100	-0.97706900	-0.89247300
H	-1.23820800	1.47108200	0.00315600

*

ts0-1bbeckm

C	-1.60941600	-0.23537800	-0.22235400
N	-0.83436700	0.00533700	-1.10709600
O	0.70551100	1.00368900	0.05396200
H	1.53825600	0.47684200	0.01410300
H	0.90909400	1.87009200	-0.31522600
O	2.92259700	-0.52350600	0.01952700
H	3.42058400	-0.79731200	-0.75646300
H	3.53207900	-0.54811400	0.76341700
C	-2.19114500	-0.29365100	1.11905600
H	-3.15252000	0.22492600	1.12727600
H	-2.32657500	-1.33597000	1.41650600
H	-1.47576700	0.20778300	1.77759800
H	-2.03294700	-0.64835900	-1.29338700

*

ts0-10beckm

C	-0.81457100	2.32845300	0.00017000
N	-0.51548800	3.51467500	0.00019200
O	1.62565000	3.53679200	0.00003100
H	1.87615200	4.06034800	-0.77169100
H	1.87623700	4.06048600	0.77163300
C	-0.62440500	0.92627300	0.00011000
C	0.69064500	0.41627800	0.00008600
C	-1.73635100	0.05202100	0.00007100
C	0.89289800	-0.94791200	0.00001800
H	1.53371600	1.09827600	0.00012900
C	-1.53070700	-1.30330100	0.00000000
H	-2.74835700	0.44482600	0.00009200
C	-0.21540700	-1.82023600	-0.00002800
H	1.90439200	-1.33343300	0.00000200
H	-2.35915400	-2.00165800	-0.00003300
O	-0.12500900	-3.14006200	-0.00009700
C	1.15644900	-3.77431500	-0.00012200
H	1.71725400	-3.50774400	0.90033100
H	0.94908700	-4.84189900	-0.00018300
H	1.71727000	-3.50764600	-0.90053700
H	-1.85560500	2.88426600	0.00025400

*

ts0-10bbeckm

C	1.48424500	0.08833500	-1.47923100
N	2.68594800	0.26965000	-1.52581300
O	3.11535400	0.90294800	0.52052100
H	3.76369500	0.25706100	0.88153700
H	3.57776100	1.74593200	0.46098000
O	4.85424000	-0.91129200	1.53924800
H	5.64463800	-1.25555600	1.11363300
H	4.94487100	-1.08560800	2.48046300
C	0.16066300	0.01221000	-0.98768500
C	-0.90528800	-0.39321800	-1.82224300
C	-0.06693600	0.35501800	0.36025400
C	-2.17612100	-0.45297500	-1.30970100
H	-0.72796400	-0.65425000	-2.86072000

C	-1.34846200	0.29401200	0.86956600
H	0.76656700	0.66399100	0.98214700
C	-2.41323900	-0.11039800	0.04011800
H	-3.01719200	-0.75917000	-1.92037200
H	-1.51810600	0.55839700	1.90533800
O	-3.67492200	-0.20067700	0.43453700
C	-4.03058200	0.13100500	1.77789200
H	-3.80468200	1.18072000	1.98705100
H	-5.10392300	-0.03268400	1.84153700
H	-3.51555000	-0.52446100	2.48608500
H	1.88098500	-0.14957700	-2.59372800

*

ts0-0beckm

C	-1.21626800	-0.37160400	0.00488600
N	-0.68272900	0.69282500	-0.00929500
H	-1.39664500	-1.44493700	0.01951800
O	1.43399100	0.01463400	-0.00051700
H	1.95009800	0.27246600	-0.77470800
H	1.95019600	0.29168800	0.76694200
H	-2.03864300	0.54492800	-0.00680300

*

ts0-0bbeckm

C	-1.98103000	-0.69708800	-0.08028200
N	-1.55192200	0.38291400	-0.32729700
H	-2.14720500	-1.69944800	0.30646000
O	0.24895800	0.13267700	0.97062500
H	1.09772500	0.11001500	0.46926700
H	0.37717200	0.75169100	1.69770600
O	2.51150900	-0.02526900	-0.48467300
H	2.92550600	0.69602800	-0.96799900
H	3.20538500	-0.66276100	-0.29049200
H	-2.67208800	-0.01236300	-0.88232900

*

sm7-0beckm

C	1.15432600	-0.47989100	-0.02094200
N	2.06995100	0.10814400	-0.66838400
O	3.37172900	0.14150900	0.31256400
H	4.07405700	-0.30994600	-0.19186500
H	3.61400500	1.08470100	0.36864000
C	-0.15717900	-0.63308300	-0.72604200
H	-0.32661100	-1.71483200	-0.80883800
H	-0.08206400	-0.22245600	-1.73726200
H	1.29373500	-0.86005800	0.99103500
C	-1.33747000	0.02880100	0.04583000
C	-1.51103400	-0.61126700	1.42984800
H	-0.67396500	-0.39614100	2.10458700
H	-2.40708800	-0.21082100	1.91150000
H	-1.63332000	-1.69726500	1.35926000
C	-2.59579100	-0.22193900	-0.79866100
H	-3.46666600	0.22429400	-0.31065200
H	-2.50501200	0.22579400	-1.79337300
H	-2.79127500	-1.29227800	-0.91896100
C	-1.08941100	1.53648800	0.17889800
H	-0.93491400	2.00546300	-0.79849900
H	-1.95154800	2.01905200	0.64701400
H	-0.21730400	1.75633800	0.80710400

*

sm7-0bbeckm

C	0.49083700	-0.32807700	-0.04532100
N	1.42341200	0.53927900	-0.02594700
O	2.52937900	0.00092800	0.88242700
H	3.39660200	-0.04149000	0.27757100
H	2.65809300	0.69490800	1.55190500
C	-0.71609600	-0.04381200	-0.87114900
H	-0.78035900	-0.84567800	-1.61859100
H	-0.57885800	0.90340800	-1.40104300
H	0.56496800	-1.25318400	0.52716800
O	4.55325200	-0.06935300	-0.52685100
H	4.63552900	0.43939500	-1.34122700
H	5.19065200	-0.79178000	-0.54169300
C	-2.03538400	-0.01255900	-0.04418100
C	-1.96200000	1.10609700	1.00310400
H	-2.91423200	1.18866600	1.53450400
H	-1.18642200	0.91552500	1.75467700
H	-1.75495200	2.07525600	0.53721100
C	-3.17014400	0.27827400	-1.03644700
H	-3.24390400	-0.50344600	-1.79955600
H	-4.12808200	0.32302200	-0.51046900
H	-3.02204500	1.23817800	-1.54163400
C	-2.28504000	-1.36563600	0.63678900
H	-3.26105900	-1.35965600	1.12989000
H	-2.28756700	-2.18553200	-0.08946700
H	-1.54338700	-1.59018100	1.41188700

*

sm6-9beckm

C	0.85986700	0.77463100	0.28622800
N	2.12528000	0.92413500	0.27538400
O	2.82187500	-0.44350000	0.04758200
H	3.64741400	-0.40366700	0.56278000
H	2.26270100	-1.21625500	0.29157100
C	0.11877600	2.07677500	0.52442500
H	-0.33848000	2.01183000	1.52746300
H	0.82754700	2.91675700	0.52456400
C	0.10970000	-0.48966600	0.10684000
C	-0.74150200	-0.94473100	1.11541700
C	0.23443200	-1.23237000	-1.07172200
C	-1.41567400	-2.14589400	0.90290900
H	-0.88085400	-0.39576700	2.04053800
C	-0.50329500	-2.41204200	-1.17734800
H	0.84341300	-0.88693900	-1.90112600
H	-2.08245200	-2.53920100	1.66443800
H	-0.44822800	-3.01016700	-2.08215700
N	-1.30269300	-2.86743100	-0.21347700
O	-0.83020800	2.15976900	-0.48659700
C	-1.58060900	3.37015300	-0.44722600
H	-0.92787600	4.23739100	-0.60135100
H	-2.30387600	3.30870300	-1.25839900
H	-2.10902400	3.46921600	0.50864300

*

sm6-9bbeckm

C	0.41658000	0.95327200	0.76331600
N	1.50699200	0.99453100	1.42596200
O	2.05921700	-0.36305000	1.65130200
H	2.26211700	-0.96297200	0.72758700
H	2.86954400	-0.18220200	2.15325500
C	-0.19369900	2.32079900	0.53654700
H	-1.19587400	2.31722800	0.99568900
H	0.41905300	3.08255500	1.03708800

O	2.47959100	-1.64554400	-0.36842700
H	2.94176600	-2.49261900	-0.33665300
H	1.70050400	-1.73048000	-0.94101000
C	-0.25057100	-0.26503800	0.23606800
C	-0.72338300	-1.24630000	1.10765500
C	-0.40164100	-0.45113400	-1.14219000
C	-1.31000300	-2.38609000	0.55818000
H	-0.64351500	-1.13447500	2.18349700
C	-0.99084400	-1.63876200	-1.57814000
H	-0.08407400	0.31081900	-1.84461200
H	-1.69425300	-3.16836000	1.20647900
H	-1.12344400	-1.82409500	-2.64047700
N	-1.42815200	-2.59489800	-0.75379500
O	-0.25745800	2.51452200	-0.84530100
C	-0.90219500	3.73094900	-1.20327900
H	-0.37608100	4.59201200	-0.77416500
H	-0.86970700	3.79248400	-2.29007900
H	-1.94597100	3.73193400	-0.86604300

*

sm6-8beckm

C	0.84104000	0.77454100	0.27739800
N	2.10722700	0.94513400	0.27240800
O	2.82092800	-0.41551500	0.08242600
H	3.59835500	-0.38545800	0.66742900
H	2.22492600	-1.17903600	0.27833500
C	0.09492700	2.07441000	0.50944000
H	-0.35947000	2.01724700	1.51415200
H	0.80324700	2.91496000	0.50491600
C	0.11364100	-0.49894600	0.10496400
C	-0.81261100	-0.90164000	1.07611400
C	0.33441200	-1.28806400	-1.03601200
C	-1.48476500	-2.10780800	0.92105400
H	-1.00138700	-0.28385700	1.94817900
C	-0.35899700	-2.48559500	-1.18637300
H	0.99860600	-0.94209200	-1.82322300
C	-1.25869300	-2.89802200	-0.20610300
H	-2.19501400	-2.42676600	1.67567400
H	-0.20524800	-3.08569800	-2.07626500
H	-1.79465100	-3.83338000	-0.32536000
O	-0.85944000	2.16099900	-0.49708700
C	-1.58919500	3.38215900	-0.46292200
H	-0.92419600	4.23819300	-0.62910900
H	-2.31987600	3.32664400	-1.26802500
H	-2.10897600	3.49957500	0.49598400

*

sm6-8bbeckm

C	0.28777900	0.81193500	0.61498000
N	1.48658200	1.06234400	0.99641300
O	2.26272400	-0.24614300	1.14379800
H	2.96423700	-0.25290800	0.37130700
H	2.72584400	-0.13112800	1.99008700
C	-0.54631400	2.07808500	0.57234600
H	-1.30986500	1.99116100	1.36528400
H	0.08123100	2.94932000	0.80725400
O	3.84149500	-0.22177000	-0.77803000
H	4.15855400	0.60320700	-1.16254800
H	4.43642800	-0.93305900	-1.03921900
C	-0.32301300	-0.47331100	0.23797400
C	-1.55497300	-0.83144700	0.79631500
C	0.28500700	-1.30520100	-0.70948500
C	-2.15071900	-2.03571900	0.44097500
H	-2.04321500	-0.18422900	1.51761700
C	-0.33105700	-2.49380400	-1.08063100
H	1.21033900	-1.00434300	-1.19070600
C	-1.54173900	-2.86518700	-0.49813900
H	-3.09648600	-2.31982500	0.88874000
H	0.12897900	-3.12841900	-1.83024700
H	-2.01684200	-3.79701200	-0.78546600
O	-1.11771300	2.15141700	-0.69473300
C	-2.00702400	3.24894100	-0.83529100
H	-1.48175600	4.20322200	-0.70346000
H	-2.41172500	3.19277500	-1.84474800
H	-2.82776400	3.18150000	-0.10944500

*

sm6-6beckm

C	0.80604500	-0.25851700	0.22894000
N	2.04210800	-0.43908200	0.37752900
O	2.39740700	-2.03281200	-0.05016400
H	3.10285900	-1.92937100	-0.71337500
H	2.81015000	-2.39630800	0.75362500
C	0.44867700	1.17864700	0.62171100
H	-0.29750700	1.08010600	1.42534200
H	1.33043300	1.71220700	1.00275200
C	-0.30062400	-1.13987100	-0.29570900
H	-0.50273700	-0.77009200	-1.31532700
H	0.02550100	-2.18503200	-0.35326700
O	-1.38441800	-0.96348400	0.55606400
C	-2.59409100	-1.51164900	0.03997400
H	-2.49499900	-2.59124600	-0.12320000
H	-3.35856800	-1.32751900	0.79269200
H	-2.87117500	-1.01637400	-0.89798000
O	-0.06913100	1.74078000	-0.53742800
C	-0.47665300	3.09626000	-0.36055100
H	0.37577400	3.72349100	-0.07450000
H	-0.86602000	3.42603700	-1.32218100
H	-1.26304600	3.16665200	0.39949400

*

sm6-6bbeckm

C	-0.40716700	0.29888400	0.07581600
N	0.45268100	1.22807400	-0.08239400
O	1.74258700	0.71420900	-0.49045800
H	2.45736600	1.38504200	-0.15605400
H	1.81084300	-0.29075300	-0.19651300
C	-1.79508300	0.74297800	0.45911200
H	-1.92899900	0.57192600	1.54112800
H	-1.91570300	1.81564000	0.26091400
O	3.47352900	2.32872500	0.30094600
H	3.26758700	2.96304700	0.99646000
H	4.05553600	2.76439300	-0.33161600
C	-0.19218700	-1.18502900	-0.08956400
H	-0.85346600	-1.73304400	0.58792300
H	-0.44966300	-1.46628600	-1.11980700
O	1.17054900	-1.49051900	0.20575300
C	1.55154000	-2.84416500	-0.08570400
H	1.42323800	-3.05221000	-1.15193600
H	2.59770000	-2.94168100	0.19802700
H	0.94065900	-3.52377600	0.51315300
O	-2.67285800	-0.04693900	-0.29238700
C	-4.03989300	0.22964100	-0.02010800
H	-4.27988600	1.27271500	-0.25895100

H -4.62637600 -0.43211100 -0.65565200
H -4.27492700 0.03153100 1.03334600
*

sm6-5beckm
C 0.74256100 0.54302400 0.05347100
N 1.91524200 0.27293800 0.46737800
O 2.36897300 -1.08557800 -0.10566900
H 3.26484200 -1.20934000 0.25654200
H 1.75485500 -1.80465100 0.19500500
C 0.20725900 1.84680200 0.61623700
H -0.50676500 1.58092400 1.41481600
H 1.02543300 2.42769000 1.06280600
C -0.12754000 -0.25949000 -0.87944500
H -0.89748300 0.40595300 -1.27672500
H 0.45524000 -0.65568800 -1.71523600
C -0.81322600 -1.41530800 -0.16718900
F -1.57291700 -2.11408600 -0.98664000
F -1.52793200 -1.01469900 0.87881200
F 0.14238700 -2.29546500 0.33995400
O -0.41039900 2.49928200 -0.44626900
C -0.96463300 3.76298100 -0.08434400
H -0.18102100 4.44397400 0.26609700
H -1.42014000 4.16623600 -0.98690100
H -1.72862100 3.64238400 0.69268000
*

sm6-5bbeckm
C 0.50272000 0.56399700 -0.07059400
N 1.57335600 -0.07162400 0.20912600
O 1.55867200 -1.43329800 -0.36737400
H 2.51155700 -1.85173600 -0.10717900
H 0.80666900 -1.94413900 0.00794000
C 0.42629500 1.94581800 0.53757700
H -0.23610600 1.88267500 1.41819100
H 1.42271500 2.25709500 0.87876200
O 3.72557000 -2.37757100 0.25704900
H 4.27945500 -1.90074700 0.88661100
H 4.29566000 -2.82325200 -0.38058400
C -0.65474200 0.07644900 -0.90510200
H -1.21089000 0.94705700 -1.25907700
H -0.30122900 -0.49118500 -1.76941400
C -1.60231700 -0.80186200 -0.10633900
F -2.60978700 -1.23635300 -0.84517700
F -2.08113000 -0.18294200 0.97197900
F -0.93583100 -1.92320000 0.36072500
O -0.09783200 2.79002200 -0.44122900
C -0.28555200 4.12530000 0.01297600
H 0.66931800 4.57432500 0.31029400
H -0.70211500 4.68005500 -0.82611800
H -0.98618900 4.15350100 0.85646500
*

sm6-4beckm
C 0.78606500 -0.09562400 0.14062800
N 2.02965500 -0.25508000 0.33536700
O 2.43881700 -1.77475900 -0.11759500
H 3.07561600 -1.64762200 -0.84498500
H 2.93694100 -2.11531200 0.64804200
C 0.38156600 1.30310800 0.61162100
H -0.16854600 1.16510400 1.55912500
H 1.27145200 1.91159300 0.82426900
C -0.24340900 -0.98738800 -0.46952000
H -0.48901500 -0.53287300 -1.43736900
H 0.15172400 -1.98859200 -0.63851300
C -1.53365300 -1.06933300 0.35822400
H -2.02700300 -0.09689000 0.42518700
H -1.34945600 -1.47290300 1.35884600
F -2.37271200 -1.93846300 -0.30344100
O -0.41166900 1.82991300 -0.39829500
C -0.77739300 3.19083600 -0.17173500
H 0.11207400 3.83054800 -0.14418800
H -1.41022500 3.47924200 -1.00904700
H -1.33727400 3.29131200 0.76531700
*

sm6-4bbeckm
C 0.16680100 0.19599100 0.49822400
N 1.04478900 -0.32771900 1.26335800
O 1.12759500 -1.82251700 0.98656200
H 1.87647800 -1.97075100 0.25162700
H 1.43067300 -2.18476800 1.83566500
C -0.01501000 1.67692500 0.76541900
H -1.00152000 1.80367700 1.24586600
H 0.75129300 2.03208200 1.46770900
O 2.80941700 -2.09862400 -0.79150100
H 3.58804900 -1.53962700 -0.89250900
H 2.89391100 -2.86610400 -1.36820700
C -0.67698800 -0.46273700 -0.54794500
H -0.87711600 0.27794300 -1.32521500
H -0.16193800 -1.31183700 -0.99946700
C -2.00843700 -0.95231200 0.04073900
H -2.60523300 -0.12429100 0.43413900
H -1.85297400 -1.69936300 0.82494600
F -2.71844900 -1.54619100 -0.98094600
O 0.04547100 2.32094300 -0.46946700
C -0.15366100 3.72604500 -0.37163000
H 0.62412600 4.18854500 0.24784100
H -0.09255900 4.11959600 -1.38501300
H -1.14126600 3.94978200 0.05025400
*

sm6-3beckm
C 0.43735700 0.09015600 0.15112200
N 1.60057800 -0.39961600 0.33553600
O 1.63739300 -1.83922000 -0.16307900
H 2.43957500 -2.21826900 0.23549500
H 0.79669400 -2.35166900 0.08757300
C 0.32167900 1.52656100 0.61523000
H -0.21418100 1.51946700 1.58091000
H 1.31939400 1.95519500 0.78009200
C -0.76720700 -0.61256600 -0.41956700
H -1.61196500 0.07920500 -0.37802300
H -0.57906300 -0.81943400 -1.47963500
C -1.07388900 -1.88390100 0.34545900
C -1.10683800 -3.10488800 -0.20384400
H -1.29518900 -1.76663100 1.40522100
H -1.36213000 -3.97699800 0.38950700
H -0.93541100 -3.26079100 -1.26765200
O -0.39743200 2.19309500 -0.37435900
C -0.57209800 3.57938200 -0.10022200
H 0.39668500 4.08930500 -0.04567000
H -1.15138200 3.98602100 -0.92737500
H -1.11882000 3.72433000 0.83954700

*

sm6-3bbeckm

C	0.10971400	0.40382600	0.04015700
N	1.05273600	-0.45710400	0.08977600
O	0.60472200	-1.76639000	-0.41499700
H	1.36646700	-2.44172200	-0.14495600
H	-0.29799400	-2.00643700	-0.06202400
C	0.49932700	1.77148900	0.54610400
H	0.03955300	1.90610000	1.54158100
H	1.58959600	1.83656300	0.66139800
O	2.40723700	-3.32031500	0.24239000
H	3.09933200	-2.98568200	0.82459700
H	2.81471900	-3.91885500	-0.39416100
C	-1.30441800	0.15768800	-0.42094400
H	-1.85474900	1.09777500	-0.33643200
H	-1.28656300	-0.10875200	-1.48400100
C	-1.96828400	-0.92797100	0.39947700
C	-2.46011700	-2.06311100	-0.10879400
H	-2.05127700	-0.74127100	1.46903400
H	-2.95009500	-2.79401900	0.52600600
H	-2.43414400	-2.27007600	-1.17724200
O	0.00019700	2.69338700	-0.37539800
C	0.24947900	4.04187800	-0.00397800
H	1.32658300	4.23443600	0.07020900
H	-0.17705300	4.66473700	-0.78880000
H	-0.23182700	4.27738900	0.95380600

*

sm6-2beckm

C	0.53508800	0.25711800	0.11912600
N	1.19741000	1.30090200	0.38631500
O	2.77184200	1.06945000	-0.11518500
H	2.90933600	1.77048600	-0.77713500
H	3.29136100	1.31155300	0.67196400
C	-0.89776100	0.45367800	0.62801100
H	-1.00502500	-0.21284800	1.50025500
H	-1.05384100	1.48772300	0.96532700
C	0.89244900	-1.01803100	-0.57686600
H	0.29919800	-1.01110700	-1.49892100
H	1.94917500	-1.01150500	-0.84254400
C	0.53213100	-2.25855300	0.25284000
H	-0.54576900	-2.33750600	0.40610600
H	0.85604100	-3.14663600	-0.29241400
H	1.03590600	-2.25872000	1.22277400
O	-1.72408600	0.09733700	-0.42852800
C	-3.10510200	0.33539100	-0.16366500
H	-3.28703600	1.40102800	0.01713800
H	-3.64456900	0.01852600	-1.05445800
H	-3.44155800	-0.25126600	0.69914600

*

sm6-2bbeckm

C	0.07957300	-0.21387900	0.36824000
N	0.99686800	0.39456500	1.01851100
O	2.36954200	-0.14382900	0.60389200
H	2.84778700	0.66127900	0.12251000
H	2.81815900	-0.30153200	1.45157500
C	-1.29876300	0.25411900	0.79679700
H	-1.72101700	-0.53375700	1.44402900
H	-1.22121200	1.17390500	1.39224600
O	3.48816500	1.76765600	-0.49853700
H	3.17187600	2.67255900	-0.39981800
H	4.03140700	1.71179900	-1.29216900
C	0.18197200	-1.26651700	-0.68958700
H	-0.26957900	-0.82634900	-1.58565900
H	1.22564100	-1.49490200	-0.90247900
C	-0.59166800	-2.53994000	-0.31263600
H	-1.65992200	-2.34028000	-0.20725400
H	-0.46773500	-3.27835200	-1.10670600
H	-0.21704900	-2.97881400	0.61592600
O	-2.05008200	0.43042000	-0.36483200
C	-3.39120000	0.81921500	-0.10174100
H	-3.42465200	1.77523800	0.43498800
H	-3.87781100	0.92985500	-1.06962800
H	-3.91200600	0.05172100	0.48475000

*

sm6-1beckm

C	0.57752000	-0.09243200	0.22246900
N	1.33373500	0.92314800	0.17334700
O	2.85835400	0.41582900	-0.19141100
H	3.06755600	0.87937300	-1.02285800
H	3.40658200	0.81483600	0.50838900
C	-0.83729200	0.35694700	0.59959300
H	-1.02160200	-0.03387900	1.61474800
H	-0.90248400	1.45344800	0.63371400
C	0.82254300	-1.53642900	-0.02733000
H	0.50681900	-2.09869800	0.85648500
H	0.16436600	-1.84044800	-0.84684100
H	1.86085700	-1.75686000	-0.26223800
O	-1.67449300	-0.20715900	-0.35336300
C	-3.03490000	0.19946900	-0.21729900
H	-3.13007400	1.28472600	-0.33813300
H	-3.58287000	-0.30542000	-1.01089600
H	-3.43551000	-0.10388900	0.75681300

*

sm6-1bbeckm

C	-0.08556500	-0.57449000	0.22027900
N	0.85630100	-0.13582800	0.96486300
O	2.19351800	-0.68309100	0.47395200
H	2.72517800	0.15833600	0.11908400
H	2.62631200	-0.99985200	1.28487200
C	-1.44448700	-0.09000500	0.68692500
H	-1.95560300	-0.95702800	1.14042900
H	-1.33290600	0.68333600	1.45909600
O	3.42843700	1.29808000	-0.32795500
H	3.18389400	2.19443800	-0.07203000
H	3.96241000	1.33498800	-1.12901400
C	-0.02766200	-1.44645100	-0.98417100
H	-0.77833800	-2.23484700	-0.88503100
H	-0.32879300	-0.84285200	-1.84614000
H	0.95426300	-1.88269300	-1.15151900
O	-2.11491800	0.37382300	-0.44489500
C	-3.43983100	0.80599800	-0.16441700
H	-3.43801800	1.62694300	0.56288500
H	-3.85846800	1.15641500	-1.10662200
H	-4.04680200	-0.02298800	0.22026500

*

sm6-10beckm

C	0.78079700	1.73318500	0.16912700
N	1.95817800	2.22629700	0.05187600

O	2.99837900	1.07516800	-0.01235600
H	3.77131100	1.41290100	0.47310000
H	2.63569200	0.24785000	0.38832700
C	-0.25223200	2.83145300	0.34726800
H	-0.57927100	2.80641300	1.40196100
H	0.21618100	3.80999700	0.16868900
C	0.38873300	0.32087100	0.17709900
C	-0.52838200	-0.14509200	1.14114800
C	0.84299900	-0.56599900	-0.81357900
C	-0.92670900	-1.46209000	1.14445800
H	-0.92051000	0.52911900	1.89519600
C	0.42878100	-1.89149700	-0.82791800
H	1.47466700	-0.20936200	-1.62280300
C	-0.45404900	-2.35219100	0.16021100
H	-1.62095500	-1.83613600	1.88809100
H	0.77540000	-2.54490400	-1.61849600
O	-0.91110600	-3.59986500	0.24610200
C	-0.51131300	-4.56591600	-0.72306300
H	0.57239200	-4.71787500	-0.69576100
H	-1.01572700	-5.48736700	-0.44030200
H	-0.83098400	-4.26578500	-1.72587700
O	-1.29117100	2.55875000	-0.53412400
C	-2.29834100	3.56228100	-0.52718400
H	-1.88681200	4.53114100	-0.83542400
H	-3.05412800	3.24408700	-1.24334100
H	-2.75267000	3.65564400	0.46737600

*

sm6-10bbeckm

C	1.60460200	0.08538500	-0.01320900
N	2.37222300	-0.90793100	-0.28059400
O	1.59937600	-2.15453600	-0.45928200
H	2.21869800	-2.92652200	-0.09258900
H	0.71827500	-2.08755800	-0.02478200
C	2.36654300	1.36067100	0.26784600
H	2.27957900	1.57519800	1.34784800
H	3.43142200	1.20890500	0.04210000
O	3.08813200	-3.91750800	0.41261900
H	3.93790200	-3.63371600	0.76973000
H	3.22463000	-4.74461500	-0.06368300
C	0.13465500	0.06580700	0.07178200
C	-0.50896200	0.63304600	1.18864100
C	-0.64858500	-0.43868800	-0.97617100
C	-1.88386300	0.64936800	1.27060400
H	0.07350200	1.05537200	2.00102500
C	-2.03710400	-0.40841700	-0.91010100
H	-0.17591700	-0.81405500	-1.87950800
C	-2.66419400	0.13112700	0.22065100
H	-2.38956900	1.07221100	2.13105200
H	-2.61332300	-0.78451500	-1.74603700
O	-3.98665600	0.20677800	0.39294500
C	-4.85382200	-0.27363600	-0.62834400
H	-4.70186400	-1.34493900	-0.79651000
H	-5.86274500	-0.10280600	-0.25849500
H	-4.70550300	0.28106400	-1.56041000
O	1.79117900	2.36718000	-0.50454300
C	2.39810000	3.63276500	-0.29974000
H	3.46282400	3.60765600	-0.56469000
H	1.87930600	4.33505600	-0.95067800
H	2.28958900	3.95468700	0.74444800

*

sm6-0beckm

C	0.65482800	-0.32997500	0.02873600
N	1.59380200	0.32605000	-0.50566100
O	2.91098000	0.00157000	0.35204900
H	3.57254900	-0.29770100	-0.30105100
H	3.20857300	0.86474500	0.69953300
C	-0.70689200	-0.21121000	-0.61310600
H	-0.88479500	-1.16246600	-1.14496600
H	-0.72686900	0.60501300	-1.34920000
H	0.75865400	-0.95225000	0.91797200
C	-2.93924500	0.12150800	0.04979300
H	-3.28539200	-0.78178800	-0.46580500
H	-3.51054400	0.26241300	0.96536300
H	-3.06336200	0.99484800	-0.60060400
O	-1.57714400	-0.01830900	0.45204200

*

sm6-0bbeckm

C	-0.14873500	-0.23738900	0.20430900
N	0.84809500	0.52196300	-0.00336500
O	1.98176600	0.03872700	0.87542500
H	2.82981500	-0.05000300	0.22827000
H	2.14653700	0.76496800	1.50284000
C	-1.40685900	0.00822800	-0.57662300
H	-1.44853100	-0.75088100	-1.37781700
H	-1.38512500	1.00105300	-1.04808600
H	-0.13929400	-1.05207200	0.92911900
O	3.93360200	-0.13302400	-0.59941200
H	3.98588600	0.33532400	-1.44050700
H	4.53344000	-0.88736500	-0.61889800
C	-3.73283800	0.00715200	-0.25109500
H	-3.89424300	-0.75295000	-1.02573400
H	-4.45788600	-0.13008700	0.54939800
H	-3.85228500	1.00711500	-0.68545700
O	-2.44690900	-0.13949900	0.33878400

*

sm5-9beckm

C	1.01689000	0.32121800	0.46514100
N	2.29049700	0.37185900	0.36291100
O	2.84099200	-0.98074200	-0.07956900
H	3.70534200	-1.07707300	0.36076200
H	2.23040500	-1.73588500	0.09746200
C	0.42576500	1.64144400	0.88958600
H	-0.12357400	1.50843300	1.82636400
H	1.21538300	2.37990100	1.04321000
C	-0.54278200	2.17973800	-0.16555500
C	0.17354000	-0.87655500	0.22879400
C	-0.59472100	-1.40304300	1.26939400
C	0.12905300	-1.48096500	-1.03275800
C	-1.36151400	-2.53545900	1.00010000
H	-0.60306300	-0.95918600	2.25920900
C	-0.69017800	-2.60068800	-1.18653800
H	0.66656300	-1.06989400	-1.88181600
H	-1.96751700	-2.98274600	1.78227500
H	-0.76939400	-3.08990700	-2.15278300
N	-1.41067600	-3.12443200	-0.19581500
F	-0.89354900	3.42666000	0.13575300
F	0.03582900	2.17456600	-1.37871800
F	-1.65341600	1.43197400	-0.24124500

*

sm5-9bbeckm

C	0.57731000	0.14460700	0.90479700
N	1.72020200	-0.32164400	1.24694700
O	1.70841300	-1.82096900	1.13387100
H	2.21794900	-2.07088400	0.23206800
H	2.23860600	-2.10955700	1.89620900
C	0.51560500	1.64704800	1.02388400
H	-0.34368400	1.92847600	1.63966300
H	1.42750800	2.02943500	1.48601600
O	2.82828600	-2.31634200	-0.99864400
H	3.58284200	-1.80320600	-1.31169500
H	2.84224800	-3.18339500	-1.42061000
C	0.35794800	2.31345400	-0.34136200
C	-0.60168300	-0.61811300	0.43064200
C	-1.77824400	-0.60726400	1.17694300
C	-0.56967600	-1.30642800	-0.78141500
C	-2.86892100	-1.32158500	0.68387700
H	-1.85610700	-0.07801300	2.12092800
C	-1.72713200	-1.96591000	-1.18681400
H	0.31038600	-1.29893700	-1.41609700
H	-3.79978700	-1.35151400	1.24236300
H	-1.75055600	-2.49816500	-2.13345000
N	-2.85216700	-1.98636200	-0.47144300
F	0.52475100	3.63084800	-0.22858800
F	-0.84770500	2.08594800	-0.87753500
F	1.28120400	1.84270000	-1.20542800

*

sm5-8beckm

C	1.01132600	0.32913700	0.47068600
N	2.28695400	0.39581500	0.37075700
O	2.84778900	-0.96063600	-0.03908300
H	3.67795400	-1.07133900	0.45877900
H	2.19974500	-1.69514200	0.11081800
C	0.41216900	1.65015600	0.88257900
H	-0.13886300	1.52418400	1.81908200
H	1.19790000	2.39332000	1.03350700
C	-0.55466400	2.18003100	-0.17655700
C	0.18274900	-0.87461100	0.23888800
C	-0.65233700	-1.34572600	1.26146700
C	0.21099700	-1.51800000	-1.01042000
C	-1.43086600	-2.47486300	1.04084900
H	-0.69035900	-0.84151300	2.22193400
C	-0.58820400	-2.63805500	-1.22143600
H	0.80502200	-1.11215100	-1.82500400
C	-1.40061200	-3.11819600	-0.19674200
H	-2.07299900	-2.84603200	1.83177900
H	-0.58499100	-3.12356800	-2.19085900
H	-2.02222800	-3.99076800	-0.36564300
F	-0.90766000	3.43014100	0.11764900
F	0.02421300	2.17163300	-1.38958100
F	-1.66650000	1.43408100	-0.25133400

*

sm5-8bbeckm

C	0.57613200	0.06783400	0.90375600
N	1.68247100	-0.50149700	1.22281700
O	1.53091700	-2.00250400	1.13238600
H	2.06042800	-2.29460500	0.26970900
H	2.00159000	-2.32193600	1.92067700
C	0.69027800	1.56992900	1.02158900
H	-0.13070200	1.95601900	1.63141400
H	1.63766400	1.84490100	1.48865000
O	2.72080200	-2.56417700	-0.96118200
H	3.43150300	-2.00201700	-1.29133700
H	2.80321400	-3.43733600	-1.36069500
C	0.62279300	2.24802400	-0.34448500
C	-0.68075800	-0.55227200	0.44543600
C	-1.87761500	-0.23225300	1.09604100
C	-0.68941600	-1.39843800	-0.66951400
C	-3.07131500	-0.79031800	0.65370400
H	-1.88215800	0.43260700	1.95422000
C	-1.89146000	-1.92703300	-1.12296700
H	0.22941100	-1.60204000	-1.21059300
C	-3.07957300	-1.63182700	-0.45673000
H	-3.99687600	-0.55700900	1.16804800
H	-1.90333800	-2.56304300	-2.00135900
H	-4.01545900	-2.05007600	-0.81120800
F	0.90368200	3.54702600	-0.22245000
F	1.52052900	1.70164900	-1.19008400
F	-0.58483200	2.13238600	-0.91078700

*

sm5-6beckm

C	0.60670200	-0.76380000	0.39563500
N	1.82133200	-1.13569600	0.41162800
O	1.90137000	-2.62528200	-0.22812100
H	2.51611700	-2.52309200	-0.97919500
H	2.36117100	-3.15119000	0.45325600
C	0.47482600	0.63632600	0.95671100
H	-0.41647700	0.65371000	1.58865500
H	1.35665500	0.91389600	1.53707400
C	-0.62113900	-1.48987400	-0.10728200
H	-0.69384000	-1.26550800	-1.18656500
H	-0.49246000	-2.57412000	0.00695200
O	-1.70691500	-1.00860300	0.60663800
C	-2.96552600	-1.34920200	0.02884200
H	-3.08350000	-2.43657300	-0.04088900
H	-3.72435200	-0.94437900	0.69574400
H	-3.06627600	-0.89570200	-0.96399100
C	0.31062600	1.66301300	-0.16648000
F	0.16650500	2.87753400	0.35382000
F	-0.75792000	1.39744200	-0.93575700
F	1.38906300	1.66221100	-0.96764000

*

sm5-6bbeckm

C	0.04341800	0.15533400	0.35164600
N	0.89048400	1.08769400	0.13601600
O	2.10812300	0.59797000	-0.45090900
H	2.86140300	1.28071400	-0.21887600
H	2.22200400	-0.41513300	-0.18679300
C	-1.27319700	0.63242800	0.90536700
H	-1.48151300	0.14228400	1.86095700
H	-1.23100200	1.71144600	1.06257000
O	3.91332400	2.21861200	0.07761200
H	3.83866000	2.81819900	0.82845400
H	4.35889500	2.69045100	-0.63515400
C	0.25766600	-1.32070200	0.09995000
H	-0.33127100	-1.91277400	0.80631700
H	-0.07890100	-1.56440000	-0.91790300
O	1.64254800	-1.61714800	0.26549000
C	2.01247300	-2.95546300	-0.11210700
H	1.81476600	-3.11732700	-1.17554000

H 3.07578500 -3.05312400 0.09752300
H 1.44790000 -3.66552000 0.49619900
C -2.43505100 0.33720800 -0.03517900
F -3.57063800 0.83188900 0.45164400
F -2.59814600 -0.99502800 -0.20270200
F -2.22219700 0.86484300 -1.24986300
*

sm5-5beckm
C 0.59004600 0.06348100 0.19353900
N 1.82055100 -0.12067000 0.47858600
O 2.35935400 -1.32786500 -0.29044900
H 3.30613000 -1.35758500 -0.05841300
H 1.88844300 -2.15205100 0.00230500
C 0.01949300 1.25146200 0.92893100
H -0.90715100 0.95129500 1.42751300
H 0.72811100 1.60869400 1.67866100
C -0.26680200 -0.77172800 -0.72823100
H -1.16021500 -0.21130200 -1.00723400
H 0.27523000 -1.02284100 -1.64433700
C -0.72695600 -2.06137200 -0.05791900
F -1.48143300 -2.78337400 -0.85939000
F -1.36747600 -1.83097500 1.08306700
F 0.37501100 -2.84574700 0.27313400
C -0.28864000 2.41510400 -0.01642700
F -0.70588400 3.46518700 0.67988000
F 0.80159600 2.75862000 -0.71499400
F -1.24968000 2.08458800 -0.90216800
*

sm5-5bbeckm
C 0.21950100 0.08374400 0.16686500
N 1.32645100 -0.51471200 0.39202500
O 1.42724800 -1.77316700 -0.36040700
H 2.44125900 -2.13129400 -0.17655600
H 0.73617500 -2.39890100 -0.04778500
C 0.08314500 1.38435500 0.91550900
H -0.89276300 1.42399200 1.40792900
H 0.86554200 1.46459500 1.67188500
O 3.68292700 -2.56612000 0.06783100
H 4.19457700 -2.15774600 0.77705800
H 4.27935800 -2.79282300 -0.65623100
C -0.89330800 -0.40693400 -0.72910500
H -1.53785600 0.42681900 -1.01073400
H -0.48888400 -0.85060100 -1.64256300
C -1.76384000 -1.44400400 -0.03323200
F -2.73463500 -1.87256900 -0.82088900
F -2.28578100 -0.97895300 1.10140700
F -1.00793500 -2.54776900 0.31384100
C 0.20185700 2.59746600 -0.00478900
F 0.13117300 3.72020000 0.70560000
F -0.78512700 2.61995600 -0.92376600
F 1.36645700 2.58248000 -0.67000100
*

sm5-4beckm
C 0.67642500 -0.60191900 0.31184100
N 1.90838500 -0.91178100 0.43578400
O 2.16224600 -2.32969700 -0.26174300
H 2.72929000 -2.13741100 -1.03346300
H 2.70562300 -2.81536300 0.38770000
C 0.46090000 0.75204700 0.96649700
H -0.33125800 0.67240200 1.71646500
H 1.37450300 1.08554500 1.46346100
C -0.43701600 -1.34654500 -0.35287300
H -0.67012400 -0.81884400 -1.28601900
H -0.12785200 -2.36032800 -0.60590200
C -1.71438900 -1.41981000 0.50011900
H -2.18827400 -0.44244900 0.61605600
H -1.51812700 -1.86269100 1.48202100
F -2.58553400 -2.24263500 -0.17480400
C 0.07181800 1.82170200 -0.05595500
F 0.05192400 3.01509400 0.52657400
F 0.95382400 1.84520300 -1.06791100
F -1.14211000 1.58653100 -0.58228200
*

sm5-4bbeckm
C -0.10232400 -0.27933200 0.61196000
N 0.87332700 -0.72886500 1.30764800
O 1.18250000 -2.13974700 0.88873000
H 1.92759300 -2.05978700 0.11301500
H 1.57501200 -2.53045200 1.68777800
C -0.41991600 1.15529000 0.95976500
H -1.50313100 1.29519900 1.01288600
H 0.02363800 1.42364300 1.92041200
O 2.77617700 -1.82066700 -0.93267400
H 3.36169700 -1.05406800 -0.95551400
H 3.11066600 -2.49690400 -1.53326900
C -0.91454200 -1.01437300 -0.41241000
H -1.33897100 -0.29495800 -1.11491800
H -0.30152300 -1.71825000 -0.97748200
C -2.06055100 -1.78690400 0.26264000
H -2.74045300 -1.11659000 0.79727700
H -1.68140200 -2.54757600 0.95139700
F -2.77124900 -2.41997700 -0.73157000
C 0.13116400 2.12080500 -0.08804500
F -0.08006100 3.37936600 0.28667500
F 1.45633900 1.94261300 -0.25272400
F -0.44561200 1.93524900 -1.28870300
*

sm5-3beckm
C 0.33418400 -0.36485200 0.36093100
N 1.53268300 -0.81093500 0.35872700
O 1.58645400 -2.14828300 -0.33616100
H 2.45238300 -2.52246200 -0.09609100
H 0.80534400 -2.75090000 -0.07449700
C 0.24801300 1.00683600 0.98149400
H -0.52109800 1.01036300 1.75893300
H 1.20574800 1.28565400 1.42524900
C -0.88917200 -1.08816300 -0.14958200
H -1.77680800 -0.50230400 0.09824200
H -0.83548200 -1.13522400 -1.24424400
C -1.00799300 -2.47345900 0.45647800
C -1.05038300 -3.60653400 -0.25670200
H -1.09104800 -2.51969800 1.54135300
H -1.17242700 -4.56761400 0.23237500
H -1.01691000 -3.60304500 -1.34498900
C -0.11426000 2.07037000 -0.05517900
F -0.12674000 3.27358700 0.51110100
F -1.33139700 1.84158900 -0.58699800
F 0.76967000 2.07476800 -1.06230800
*

sm5-3bbeckm

C	-0.10325000	-0.04857600	0.33195400
N	0.87429400	-0.87469900	0.31097800
O	0.51474500	-2.11560900	-0.37240100
H	1.32144100	-2.78569700	-0.19332100
H	-0.38168800	-2.45070000	-0.08255100
C	0.25324000	1.26678300	0.97427200
H	-0.49554800	1.53168200	1.72594000
H	1.23126800	1.19952700	1.45392900
O	2.38901900	-3.64401400	0.04810900
H	3.05113700	-3.39159600	0.70266800
H	2.84043200	-4.07986200	-0.68442900
C	-1.49527300	-0.32103100	-0.18803400
H	-2.12462800	0.54991600	0.00580600
H	-1.44616100	-0.44513100	-1.27644100
C	-2.10048200	-1.54437200	0.47084700
C	-2.52700200	-2.62404200	-0.19283500
H	-2.20311900	-1.50509300	1.55414800
H	-2.98187700	-3.45846400	0.33069500
H	-2.47901400	-2.68619100	-1.27859200
C	0.31598200	2.40037800	-0.04584200
F	0.67896600	3.53599700	0.55012800
F	1.20026900	2.12673900	-1.01646000
F	-0.87900200	2.60207700	-0.63596900

*

sm5-2beckm

C	0.89947900	0.10747900	0.27359900
N	1.59730300	1.15080000	0.49465600
O	3.03589200	0.98272500	-0.22698300
H	3.04968500	1.67863700	-0.91085100
H	3.67061900	1.24282800	0.46636900
C	-0.44417200	0.29794600	0.95906400
H	-0.61443800	-0.53659600	1.64423600
H	-0.45405800	1.22716100	1.53298300
C	1.22426000	-1.13791000	-0.49537300
H	0.59605000	-1.11725600	-1.39418000
H	2.26408200	-1.10758100	-0.81888700
C	0.93861400	-2.41912900	0.30306700
H	-0.12739400	-2.55324500	0.49318600
H	1.27663400	-3.27386400	-0.28509700
H	1.47885000	-2.43218700	1.25310800
C	-1.59369800	0.36224400	-0.04624800
F	-2.71679600	0.70832300	0.57548000
F	-1.33270400	1.26750900	-1.00410600
F	-1.79409000	-0.82034100	-0.65152100

*

sm5-2bbeckm

C	0.37449300	-0.60991500	0.44395700
N	1.23710600	0.03895500	1.13203200
O	2.62133100	-0.21577500	0.57995200
H	2.85947500	0.67976000	0.04436800
H	3.17909500	-0.26267200	1.37504100
C	-1.03012100	-0.33253600	0.93043200
H	-1.61933600	-1.25269200	0.90700700
H	-1.01491700	0.05590500	1.95044900
O	3.07127900	1.87797700	-0.61151000
H	2.47095700	2.62564000	-0.50737900
H	3.58739700	1.99185200	-1.41729200
C	0.60504700	-1.58230400	-0.67321700
H	-0.20699600	-1.47047900	-1.39491200
H	1.54437000	-1.36976100	-1.18398300
C	0.62010800	-3.02212500	-0.12226800
H	-0.31857700	-3.27594100	0.37663600
H	0.75392800	-3.71892100	-0.95135500
H	1.43913400	-3.16975200	0.58559100
C	-1.73582800	0.70218600	0.05788000
F	-2.93854000	0.98102200	0.55598100
F	-1.02794700	1.84676700	-0.00385900
F	-1.89484500	0.26732400	-1.20532800

*

sm5-1beckm

C	0.98775600	-0.11817900	0.40219200
N	1.77720900	0.87764100	0.28958600
O	3.18000100	0.35413900	-0.28577600
H	3.25460500	0.75651900	-1.17226900
H	3.84204000	0.78455600	0.28771200
C	-0.34385800	0.37532700	0.93850400
H	-0.57350700	-0.16733300	1.86011300
H	-0.30147600	1.44427600	1.15791200
C	1.20586600	-1.55717700	0.08173100
H	0.68873500	-2.17568200	0.81699800
H	0.74544900	-1.76544900	-0.89066100
H	2.26107700	-1.81918400	0.04204300
C	-1.47336900	0.14378600	-0.06625400
F	-2.58561900	0.73355400	0.35803100
F	-1.14483400	0.65025100	-1.26713100
F	-1.72376900	-1.16521200	-0.23349500

*

sm5-1bbeckm

C	0.38525000	-0.90289200	0.38508200
N	1.28363500	-0.52299900	1.21435100
O	2.64689700	-0.78445000	0.63269400
H	2.90938000	0.09206000	0.07911300
H	3.21549500	-0.87268100	1.41591500
C	-1.00034900	-0.59945100	0.90267700
H	-1.62070300	-1.49831400	0.85089800
H	-0.95298700	-0.25746500	1.93829600
O	3.07870400	1.22938700	-0.69461300
H	2.74076600	2.09074200	-0.42127200
H	3.79905700	1.35759300	-1.32250600
C	0.58049700	-1.53790000	-0.95226200
H	-0.26239100	-2.19096100	-1.18067000
H	0.60019300	-0.75677700	-1.72008200
H	1.51083200	-2.10317000	-0.99905000
C	-1.67422200	0.49441700	0.08049200
F	-2.82935700	0.85096900	0.63541100
F	-0.88530600	1.58658500	0.00329600
F	-1.91759100	0.09323000	-1.17944500

*

sm5-10beckm

C	1.00223400	1.34862600	0.39928000
N	2.17786300	1.80549500	0.15669500
O	3.11902800	0.64602200	-0.15213100
H	3.97101600	0.88197400	0.25701200
H	2.74745600	-0.20997100	0.18612500
C	0.04123900	2.46007800	0.74216600
H	-0.41508800	2.26560300	1.71669600
H	0.56545200	3.41700900	0.78528400

C	-1.07588100	2.57426200	-0.29436100
C	0.59040300	-0.06354100	0.37032500
C	-0.07890500	-0.62818300	1.47575700
C	0.77558200	-0.83924500	-0.78702800
C	-0.50239700	-1.93679900	1.43551900
H	-0.25258200	-0.04389200	2.37386600
C	0.32964900	-2.15320900	-0.84059500
H	1.21506000	-0.39773400	-1.67779700
C	-0.30850000	-2.71456200	0.27599200
H	-1.00338100	-2.39010100	2.28311300
H	0.46104800	-2.71724100	-1.75533400
F	-1.79404100	3.67385800	-0.06535600
O	-0.77235500	-3.95922200	0.33668700
C	-0.64790300	-4.81713700	-0.79658300
H	0.40596700	-4.98773200	-1.03773400
H	-1.11214700	-5.75566400	-0.50203800
H	-1.17706700	-4.39992000	-1.65860000
F	-1.90183900	1.51799800	-0.25611800
F	-0.56617600	2.64746500	-1.53543000

*

sm5-10bbeckm

C	1.35621500	-0.28152600	0.28413900
N	2.08438900	-1.28256800	-0.05833400
O	1.26725400	-2.43718800	-0.43275400
H	1.81419600	-3.30417600	-0.13927900
H	0.35488600	-2.35966000	-0.06704600
C	2.16858700	0.91622600	0.70317500
H	1.89187800	1.22136700	1.71634600
H	3.23236900	0.67187000	0.68794900
O	2.54871500	-4.41606600	0.24395200
H	3.35193400	-4.28266700	0.76136300
H	2.71187500	-5.12680100	-0.38745100
C	1.94535000	2.10959800	-0.22089900
C	-0.11952500	-0.25228700	0.29616100
C	-0.80993000	0.10942400	1.46918100
C	-0.85021700	-0.50055800	-0.87472500
C	-2.18572100	0.17975600	1.47460000
H	-0.26627100	0.32571200	2.38372300
C	-2.23707000	-0.40953900	-0.88229400
H	-0.33216100	-0.71694000	-1.80507300
C	-2.91512300	-0.07407600	0.29767700
H	-2.73040200	0.44467000	2.37355700
H	-2.77130100	-0.58074500	-1.80826900
F	2.82088700	3.07732900	0.06613700
O	-4.24025300	0.03606500	0.40643500
C	-5.05659500	-0.15367200	-0.74530900
H	-4.94398900	-1.16803200	-1.14179200
H	-6.07945500	-0.00687800	-0.40487300
H	-4.81685600	0.58517500	-1.51648100
F	2.11273500	1.75812600	-1.50799800
F	0.70983600	2.61859900	-0.09794800

*

sm5-0beckm

C	1.09827300	-0.42649500	-0.14865400
N	2.05980400	0.27354500	-0.59071300
O	3.28305000	0.00431500	0.36987600
H	4.01789900	-0.27006600	-0.21405600
H	3.51489400	0.87508100	0.75065200
C	-0.18644500	-0.33379300	-0.91352800
H	-0.39645400	-1.31660600	-1.34957100
H	-0.12825100	0.40965300	-1.71082400
H	1.17414400	-1.06401700	0.73330900
C	-1.33164700	0.02394500	0.03597200
F	-2.47719400	0.09455100	-0.62881900
F	-1.09555800	1.20507700	0.62796700
F	-1.44519100	-0.90159700	1.00314800

*

sm5-0bbeckm

C	0.42011500	-0.34502900	-0.07440700
N	1.39907200	0.46708000	-0.07082500
O	2.46765300	-0.11005900	0.81324600
H	3.38624600	-0.07403600	0.23839900
H	2.54490600	0.50851400	1.56210800
C	-0.77216000	0.01830900	-0.89995800
H	-0.90654400	-0.73816900	-1.68029300
H	-0.64852000	0.99791000	-1.36521200
H	0.42910400	-1.27195300	0.50039700
O	4.54650300	-0.01070000	-0.45782200
H	4.68857700	0.61130700	-1.18098500
H	5.15982800	-0.74983400	-0.54382900
C	-2.03247100	0.02626900	-0.03816500
F	-3.09887300	0.32922300	-0.77270200
F	-1.92285300	0.92567600	0.95296100
F	-2.22915500	-1.17810700	0.52735100

*

sm4-9beckm

C	0.81004700	0.76130800	0.27134600
N	2.06907000	0.96038600	0.19687900
O	2.81499200	-0.38836300	-0.07713800
H	3.71883400	-0.22869200	0.24974300
H	2.40805500	-1.16369500	0.36498000
C	0.04650900	2.03078700	0.54477100
H	-0.47348400	1.90238100	1.50134900
H	0.73447600	2.87396900	0.64154400
C	-0.98543200	2.31338500	-0.55433700
H	-1.71179100	1.50053500	-0.64806200
H	-0.50021200	2.49072000	-1.51893800
C	0.10369000	-0.53525700	0.12558900
C	-0.71942000	-0.99934800	1.15411300
C	0.21688000	-1.28422000	-1.05086800
C	-1.37592800	-2.21486500	0.96330500
H	-0.84737000	-0.45018100	2.08094900
C	-0.50431500	-2.47529400	-1.13547200
H	0.81420000	-0.94364300	-1.89066100
H	-2.01705200	-2.61658200	1.74226000
H	-0.45763900	-3.07820700	-2.03762900
N	-1.27637100	-2.93827200	-0.15262700
F	-1.66120900	3.45604300	-0.19081900

*

sm4-9bbeckm

C	0.57432900	0.62305700	0.62535300
N	1.77169200	0.30518400	0.95487300
O	1.88627100	-1.20892800	1.04380100
H	2.48286600	-1.52200200	0.23491400
H	2.37396500	-1.34819600	1.87320100
C	0.34434000	2.10857200	0.58664200
H	-0.42373100	2.34012900	1.33458600
H	1.25803300	2.63949900	0.86251200
O	3.21659100	-1.90094100	-0.92585900

H	4.00220000	-1.42462500	-1.21899700
H	3.27628000	-2.81164100	-1.23696600
C	-0.14973900	2.58105600	-0.78377200
H	-1.09180900	2.10065800	-1.06298900
H	0.60039000	2.40197400	-1.56073600
C	-0.53764100	-0.29726700	0.28503800
C	-1.71889100	-0.26876900	1.02422900
C	-0.44652100	-1.15182700	-0.81430000
C	-2.74854800	-1.12867100	0.64431300
H	-1.84348800	0.38584200	1.88035500
C	-1.54715700	-1.95007400	-1.11642900
H	0.43927900	-1.17941100	-1.44130900
H	-3.67846600	-1.14806800	1.20492800
H	-1.52119800	-2.61873300	-1.97224600
N	-2.67338900	-1.95114600	-0.40238200
F	-0.36571000	3.94038400	-0.70057200

*

sm4-8beckm

C	0.79837900	0.77571400	0.28153800
N	2.05923500	0.98715900	0.21690000
O	2.81050200	-0.34927500	-0.02905100
H	3.64378100	-0.26372100	0.46742200
H	2.28543900	-1.13469000	0.25236500
C	0.02348400	2.03992400	0.54513200
H	-0.50041000	1.91632600	1.49996700
H	0.70519400	2.88836900	0.64061200
C	-1.00391300	2.30866500	-0.55955800
H	-1.72300600	1.48944800	-0.65208100
H	-0.51531400	2.48330400	-1.52299200
C	0.11379400	-0.52843400	0.12946400
C	-0.76020900	-0.97069700	1.13276500
C	0.30102500	-1.29373500	-1.03447400
C	-1.41500900	-2.18757300	0.98275700
H	-0.91852800	-0.37848500	2.02854500
C	-0.37584300	-2.50137500	-1.17897200
H	0.93311000	-0.92787500	-1.83914500
C	-1.22540300	-2.94994300	-0.16992900
H	-2.08328200	-2.53601700	1.76235000
H	-0.24689600	-3.08242200	-2.08533500
H	-1.74890100	-3.89279600	-0.28527800
F	-1.69300400	3.44950000	-0.20973000

*

sm4-8bbeckm

C	0.56495400	0.58253500	0.61087000
N	1.77107900	0.25772500	0.91263400
O	1.88368900	-1.26354600	1.01363200
H	2.55103100	-1.54829300	0.26198400
H	2.30528400	-1.39899800	1.87867800
C	0.38016400	2.07783800	0.58010400
H	-0.36199100	2.33972300	1.34291900
H	1.31604900	2.57849800	0.83770900
O	3.40311100	-1.86794100	-0.85843200
H	4.12043100	-1.28752000	-1.13731100
H	3.59741300	-2.76234400	-1.15982400
C	-0.11941600	2.56453800	-0.78215200
H	-1.08400500	2.12023600	-1.04195000
H	0.60863800	2.35176400	-1.57137600
C	-0.57020700	-0.30465600	0.28566500
C	-1.79926400	-0.11059700	0.92715000
C	-0.44685800	-1.27965600	-0.71214900
C	-2.88494000	-0.91427000	0.59682300
H	-1.90786700	0.64930600	1.69456100
C	-1.54471900	-2.05947400	-1.05548600
H	0.49097100	-1.40388100	-1.24496500
C	-2.75997900	-1.88424900	-0.39570600
H	-3.83160200	-0.77435600	1.10673200
H	-1.45440100	-2.80192700	-1.84085200
H	-3.61347900	-2.49833600	-0.66227700
F	-0.27990500	3.93348200	-0.70394800

*

sm4-6beckm

C	0.62115400	-0.45066300	0.20938500
N	1.75891500	-1.01228200	0.19574300
O	1.53414200	-2.57729900	-0.30561200
H	2.14954600	-2.66598800	-1.05601900
H	1.89896700	-3.10816500	0.42600700
C	0.74789600	0.98773900	0.65456700
H	-0.01743200	1.13956100	1.42229200
H	1.73599300	1.16854100	1.08442200
C	-0.71992900	-1.04035600	-0.16143900
H	-0.70433800	-1.25289600	-1.24500400
H	-0.84167600	-2.00231400	0.36053700
O	-1.70206200	-0.12748200	0.18412200
C	-3.02267300	-0.58957100	-0.08709700
H	-3.23370400	-1.51026500	0.46970600
H	-3.69651100	0.19812600	0.24442600
H	-3.16138600	-0.76426300	-1.16054200
C	0.50763500	1.96171800	-0.50906600
H	-0.49431500	1.84625000	-0.92848300
H	1.26752700	1.84080300	-1.28754100
F	0.62129500	3.23245400	0.00851800

*

sm4-6bbeckm

C	-0.42672300	0.17645800	0.14979200
N	0.44021500	1.09164100	-0.06456600
O	1.69992400	0.55717200	-0.54653100
H	2.43411200	1.24588500	-0.27909000
H	1.79699700	-0.41911400	-0.20505600
C	-1.77983700	0.66147700	0.58454300
H	-2.03321000	0.19120800	1.54101600
H	-1.74620200	1.74290500	0.73040300
O	3.44686700	2.20483400	0.09807700
H	3.25211400	2.86764800	0.77024500
H	4.01230300	2.61445600	-0.56631600
C	-0.20676200	-1.31124200	-0.01221500
H	-0.86928600	-1.85969500	0.66577700
H	-0.44908900	-1.60111200	-1.04629900
O	1.14890300	-1.61757300	0.29174800
C	1.51436300	-2.98823300	0.06257700
H	1.38342500	-3.24359600	-0.99318900
H	2.55994900	-3.08213200	0.34923600
H	0.89882900	-3.63464700	0.69286600
C	-2.86732300	0.32139500	-0.43896400
H	-2.95227700	-0.75739800	-0.61337400
H	-2.68880300	0.83148700	-1.39049800
F	-4.07354300	0.75766000	0.06634600

*

sm4-5beckm

C	0.58082900	0.49932400	0.00912000

N	1.81039100	0.33489900	0.30991500
O	2.33735700	-0.97682100	-0.32061700
H	3.28296600	-0.98482900	-0.08446100
H	1.86636400	-1.75000800	0.08153100
C	0.01448200	1.77247400	0.57677100
H	-0.94630800	1.52963900	1.04430100
H	0.68059300	2.17414400	1.34317200
C	-0.28921800	-0.42528400	-0.80930100
H	-1.17956100	0.11000800	-1.14473900
H	0.24139800	-0.79532000	-1.69097400
C	-0.77135100	-1.62224400	0.00603600
F	-1.54134900	-2.41500100	-0.71297000
F	-1.40914700	-1.25248600	1.10999300
F	0.31335600	-2.38266900	0.42202800
C	-0.20739000	2.83377900	-0.51302800
H	-0.83413500	2.46779400	-1.33347600
H	0.74593900	3.19211500	-0.91203500
F	-0.85813300	3.88942900	0.07933500

*

sm4-5bbeckm

C	0.41862500	0.42146800	-0.02465600
N	1.34622300	-0.43021700	0.20062400
O	1.05399500	-1.73948600	-0.42509700
H	1.92093900	-2.34095000	-0.20930600
H	0.23845900	-2.11695800	-0.02611200
C	0.66941300	1.77839400	0.56836300
H	-0.25091600	2.11785300	1.05552900
H	1.45699600	1.71664800	1.32162900
O	3.00931200	-3.10092800	0.10590300
H	3.63895800	-2.78878900	0.76677100
H	3.48347500	-3.61717200	-0.55655300
C	-0.84808200	0.17020500	-0.81062400
H	-1.28119700	1.12103800	-1.12723000
H	-0.64867600	-0.42967300	-1.70269500
C	-1.90826400	-0.54321400	0.01899300
F	-3.01434700	-0.74434800	-0.67786400
F	-2.20245200	0.11876600	1.13530900
F	-1.44930100	-1.78510300	0.41375900
C	1.07251800	2.80064200	-0.50248400
H	0.32787700	2.88448000	-1.30160000
H	2.04559900	2.55268600	-0.93631200
F	1.17175400	4.02905300	0.11213400

*

sm4-4beckm

C	0.68410200	-0.20197600	0.13093800
N	1.91450100	-0.49881600	0.27270900
O	2.14791000	-2.03802800	-0.21710800
H	2.81031100	-1.96621100	-0.92930500
H	2.58802200	-2.45918000	0.54463800
C	0.46783300	1.22510800	0.59400200
H	-0.25107000	1.19717300	1.42082600
H	1.39896300	1.64879800	0.97850100
C	-0.44771800	-1.01844700	-0.41004300
H	-0.68888200	-0.61719300	-1.40305400
H	-0.15637300	-2.06024400	-0.53983900
C	-1.71014900	-0.95738400	0.46291900
H	-2.14043600	0.04719600	0.50464100
H	-1.51410900	-1.31564000	1.47828300
F	-2.63260400	-1.79407200	-0.12110700
C	-0.07061800	2.12021900	-0.53062300
H	-1.03465400	1.77058700	-0.91391100
H	0.64773800	2.19675600	-1.35264500
F	-0.25339500	3.37172800	0.00719200

*

sm4-4bbeckm

C	0.02918100	0.20215100	0.41451600
N	0.94383500	-0.40547000	1.07307000
O	0.88094500	-1.89735900	0.76465900
H	1.66368900	-2.10915500	0.08015500
H	1.08630200	-2.30965200	1.62057000
C	0.02287000	1.68676300	0.66571200
H	-1.01744200	2.00312800	0.79984400
H	0.57791000	1.92327700	1.57590400
O	2.65143100	-2.32537100	-0.89406400
H	3.54890100	-1.98001300	-0.82368500
H	2.65347800	-3.07655000	-1.49850600
C	-0.99341100	-0.40471200	-0.50069800
H	-1.31932000	0.34950900	-1.22069500
H	-0.58526500	-1.25258700	-1.05302200
C	-2.22126100	-0.87843900	0.29406100
H	-2.70941700	-0.04887700	0.81395000
H	-1.95590400	-1.65772500	1.01435400
F	-3.11620500	-1.41087700	-0.60704500
C	0.62567600	2.46240200	-0.51191600
H	0.11431700	2.24822300	-1.45584300
H	1.69376000	2.24922400	-0.61605200
F	0.47825600	3.80590500	-0.24538000

*

sm4-3beckm

C	0.30390600	0.10084700	0.15173000
N	1.50614800	-0.33043300	0.21302900
O	1.57820500	-1.75032400	-0.34754000
H	2.44476300	-2.08236900	-0.05488600
H	0.81294000	-2.32418500	-0.01141100
C	0.16953300	1.51929900	0.63084800
H	-0.61413900	1.54419300	1.39645800
H	1.10407900	1.86163300	1.08037500
C	-0.90094400	-0.68263200	-0.31408900
H	-1.79547600	-0.07109800	-0.16881900
H	-0.80742900	-0.87374200	-1.39017600
C	-1.04922900	-1.98062400	0.45670700
C	-1.07401200	-3.19304700	-0.11027700
H	-1.16556100	-1.88692900	1.53515600
H	-1.21497300	-4.08599600	0.49005800
H	-1.00529700	-3.32639000	-1.18867000
C	-0.22517700	2.46108800	-0.51489900
H	-1.14142400	2.13777700	-1.02043800
H	0.58355800	2.55242000	-1.24570800
F	-0.45889700	3.70148000	0.03423400

*

sm4-3bbeckm

C	-0.02096900	0.42830600	0.10335100
N	0.92965400	-0.42987300	0.09165500
O	0.48408700	-1.72169300	-0.46491800
H	1.27081100	-2.39232500	-0.24346600
H	-0.39363900	-1.99660500	-0.07967600
C	0.38293200	1.78032000	0.61725600
H	-0.32685800	2.08028500	1.39647100
H	1.38082900	1.73360200	1.05776000

O	2.33347500	-3.25113100	0.09335200
H	3.02588700	-2.92056200	0.67777100
H	2.74244600	-3.82768200	-0.56232300
C	-1.44728700	0.16116900	-0.31961600
H	-2.03165700	1.07487100	-0.18107400
H	-1.46788700	-0.08217900	-1.38870600
C	-2.06226900	-0.95837400	0.49586700
C	-2.57080500	-2.07858900	-0.02739800
H	-2.09276000	-0.80550200	1.57335700
H	-3.02192200	-2.83517000	0.60607500
H	-2.59585300	-2.25111600	-1.10192600
C	0.36327300	2.83427100	-0.49448400
H	-0.61223600	2.90009900	-0.98800700
H	1.13707500	2.63348700	-1.24124500
F	0.62669400	4.05896600	0.08307500

*

sm4-2beckm

C	0.52004100	0.18511100	0.10456400
N	1.21589000	1.22793100	0.31161100
O	2.76524100	0.94752000	-0.20054100
H	2.92529900	1.65660100	-0.84924100
H	3.30226100	1.15294700	0.58612700
C	-0.89025600	0.45023900	0.59927500
H	-1.08699000	-0.27338100	1.39781300
H	-0.96911100	1.45223800	1.02837500
C	0.88758400	-1.13325500	-0.51245100
H	0.40028200	-1.15501100	-1.49590700
H	1.96232300	-1.17356500	-0.68675400
C	0.42595500	-2.33400100	0.32496100
H	-0.66152700	-2.38849000	0.41044100
H	0.76497800	-3.24955800	-0.16241900
H	0.85609800	-2.30955500	1.32912900
C	-1.92952900	0.29220000	-0.51642800
H	-1.93961700	-0.72020800	-0.93180500
H	-1.76590800	1.01967000	-1.31774500
F	-3.16178000	0.53484600	0.04519700

*

sm4-2bbeckm

C	-0.17355900	-0.33936700	0.17577800
N	0.79268800	0.29779600	0.72421100
O	2.12383300	-0.26324500	0.21286900
H	2.68468300	0.59463400	-0.03867600
H	2.53681800	-0.66884100	0.99429700
C	-1.51913700	0.16439600	0.62996700
H	-2.14755600	-0.70667900	0.84546500
H	-1.42202200	0.75543300	1.54286900
O	3.43712000	1.75864800	-0.32147200
H	3.20124900	2.62478200	0.02922000
H	4.01514300	1.87501800	-1.08337700
C	-0.09760500	-1.49881700	-0.77408000
H	-1.00675900	-1.49966200	-1.38041200
H	0.75635600	-1.38954900	-1.44467100
C	-0.00153300	-2.82725000	0.00016200
H	-0.83465100	-2.94951300	0.69675300
H	-0.03010400	-3.65682000	-0.70821200
H	0.93202000	-2.89873200	0.56423800
C	-2.20085100	1.00876900	-0.45235800
H	-2.30099200	0.46981300	-1.39977100
H	-1.66036600	1.94524100	-0.61915100
F	-3.46807000	1.31803800	-0.00522100

*

sm4-1beckm

C	0.56240100	-0.10130400	0.23115000
N	1.32299300	0.90476300	0.05888200
O	2.82772700	0.36179000	-0.28049300
H	3.02875800	0.74324800	-1.15495700
H	3.38966300	0.82230600	0.36991700
C	-0.83648100	0.37512300	0.56200200
H	-1.07559500	-0.02219500	1.55515000
H	-0.87649700	1.46592900	0.61222100
C	0.86140000	-1.56032500	0.13353700
H	0.23926700	-2.10851900	0.84304700
H	0.59544300	-1.90137700	-0.87391800
H	1.91237300	-1.78324200	0.30773400
C	-1.86687800	-0.12984100	-0.45620200
H	-1.90449600	-1.22261200	-0.49843800
H	-1.67020500	0.27452500	-1.45402600
F	-3.09696000	0.32369600	-0.04024300

*

sm4-1bbeckm

C	-0.12105700	-0.65463300	0.19852900
N	0.81278900	-0.16554600	0.92624100
O	2.16783600	-0.65487000	0.41436500
H	2.66487800	0.20808800	0.06486500
H	2.61720100	-0.96791300	1.21771600
C	-1.48515000	-0.20734000	0.65458000
H	-2.05441600	-1.10476100	0.92296600
H	-1.40151400	0.42262100	1.54279400
O	3.26449600	1.38770500	-0.43768100
H	3.19063500	2.24704700	-0.00770700
H	3.98808300	1.41572100	-1.07326100
C	0.00441700	-1.55258400	-0.98946400
H	-0.84867100	-2.23270400	-1.02626100
H	-0.02300200	-0.94144400	-1.89922200
H	0.93142500	-2.12322300	-0.98818400
C	-2.23893900	0.55047800	-0.44230500
H	-2.40122000	-0.06449100	-1.33280800
H	-1.71478800	1.47035000	-0.72108100
F	-3.47326800	0.89727500	0.06366600

*

sm4-10beckm

C	0.72088400	1.74963600	0.20735200
N	1.87004600	2.28104100	-0.00037800
O	2.93595700	1.16404400	-0.19537700
H	3.74749100	1.53177500	0.19687500
H	2.66023500	0.32436300	0.24249400
C	-0.33431700	2.79773900	0.45547500
H	-0.74073300	2.63658900	1.46035000
H	0.11064100	3.79523700	0.42975600
C	-1.47019600	2.70000800	-0.56690400
H	-1.95777100	1.72140400	-0.53625300
H	-1.11071900	2.90297500	-1.58026500
C	0.39284000	0.31439800	0.21644300
C	-0.36160300	-0.22814200	1.27741000
C	0.73665600	-0.51199200	-0.86674700
C	-0.71677700	-1.55847700	1.27004800
H	-0.65474400	0.39247800	2.11826400
C	0.36067600	-1.84892500	-0.89063900
H	1.25468600	-0.10024800	-1.72887600

C	-0.36477700	-2.38491000	0.18427900
H	-1.28101900	-1.99250400	2.08751900
H	0.61851900	-2.45431400	-1.75052800
F	-2.40541800	3.65863800	-0.23878900
O	-0.77280700	-3.64835200	0.26761800
C	-0.50235400	-4.55117500	-0.80311700
H	0.57577600	-4.67952800	-0.94091800
H	-0.94743700	-5.49749900	-0.50389500
H	-0.96881600	-4.20285800	-1.72979600

*

sm4-10bbeckm

C	1.58781200	-0.08943200	-0.05963800
N	2.24023600	-1.17437300	-0.27997800
O	1.34225600	-2.34752200	-0.34500000
H	1.90991400	-3.15336100	0.04331100
H	0.49800200	-2.17418500	0.12858700
C	2.46653500	1.12534700	0.05892300
H	2.32054000	1.56505300	1.05206700
H	3.51591000	0.83636900	-0.03155400
O	2.70708500	-4.17862400	0.57311200
H	3.58627000	-3.94691700	0.89550000
H	2.76269100	-5.03816400	0.13966700
C	2.12152300	2.17730600	-0.99633900
H	1.08347300	2.51163300	-0.90965700
H	2.30410800	1.79994400	-2.00719100
C	0.12089400	0.02946900	0.07663000
C	-0.43118500	0.69406500	1.18956400
C	-0.74029400	-0.43437700	-0.92894300
C	-1.79545700	0.85183200	1.30477600
H	0.21328500	1.07537700	1.97547800
C	-2.11619200	-0.26036500	-0.83015200
H	-0.33443700	-0.89946700	-1.82328800
C	-2.65446600	0.37958700	0.29470000
H	-2.23226000	1.34734000	2.16426400
H	-2.75144400	-0.61029700	-1.63404800
F	2.94282400	3.26875400	-0.78922700
O	-3.95585600	0.59424800	0.49698200
C	-4.89782800	0.17204600	-0.48437800
H	-4.85792000	-0.91346500	-0.62164800
H	-5.87270400	0.45373600	-0.09206500
H	-4.72395400	0.68253300	-1.43703700

*

sm4-0beckm

C	0.67832500	-0.37694200	-0.01945100
N	1.58267200	0.42345200	-0.40956800
O	2.93625100	-0.04481800	0.30207300
H	3.55821800	-0.23224500	-0.42788200
H	3.26548400	0.74600500	0.77189600
C	-0.68957400	-0.15258200	-0.57862800
H	-0.94203400	-1.05190700	-1.15513000
H	-0.70057800	0.70490400	-1.25554700
H	0.87316200	-1.19211700	0.67926000
C	-1.73409900	0.02336800	0.53494500
H	-1.72748100	-0.82014000	1.23319200
H	-1.58142000	0.95896900	1.08034600
F	-2.96466900	0.07173400	-0.07407400

*

sm4-0bbeckm

C	-0.10480200	-0.34759700	0.17239500
N	0.81926100	0.52875300	0.15600200
O	1.99198300	-0.02483300	0.93599700
H	2.81494500	-0.05012500	0.24813100
H	2.17989800	0.64626700	1.61577000
C	-1.36527400	-0.04519100	-0.56396200
H	-1.48666200	-0.81584700	-1.33533100
H	-1.29846000	0.92823300	-1.05479500
H	0.01593500	-1.29372100	0.70317900
O	3.87311000	-0.06148200	-0.63242700
H	3.92022800	0.50430300	-1.41153300
H	4.49969700	-0.78791800	-0.72584200
C	-2.58952500	-0.09417900	0.35883100
H	-2.67441200	-1.05917700	0.86923300
H	-2.56635500	0.71275600	1.09675700
F	-3.70703300	0.07350800	-0.42876200

*

sm3-0beckm

C	0.48716900	-0.27994800	-0.23760100
N	1.51597600	0.45935800	-0.25913800
O	2.69448000	-0.38494500	0.45339000
H	3.41359300	-0.41719900	-0.20577500
H	2.99799300	0.17708400	1.19144400
C	-0.77484400	0.30627000	-0.81125000
H	-1.12719500	-0.39561600	-1.57531300
H	-0.56344700	1.26663500	-1.28887700
H	0.47419200	-1.27924500	0.19731000
C	-1.78807400	0.44185300	0.30570800
H	-1.60647800	1.24094800	1.02048500
C	-2.85700000	-0.34266600	0.42045600
H	-3.07015800	-1.13687500	-0.29058800
H	-3.57189800	-0.19666700	1.22300400

*

sm3-0bbeckm

C	-0.29803500	-0.23703100	-0.10020200
N	0.71792800	0.49071200	0.14405200
O	1.74886000	-0.38195500	0.84381700
H	2.64290600	-0.27302200	0.27663700
H	1.88494700	0.03446200	1.71289100
C	-1.48303400	0.39395900	-0.76280800
H	-1.72284200	-0.20409100	-1.64904000
H	-1.23466300	1.40948200	-1.08306300
H	-0.34000200	-1.28762700	0.19004700
O	3.82090200	-0.10077500	-0.45107800
H	3.95163900	0.63706800	-1.05750400
H	4.41230300	-0.82046700	-0.69851100
C	-2.64682800	0.38767300	0.20635800
H	-2.58667500	1.10315500	1.02344400
C	-3.69813700	-0.42039300	0.09477300
H	-3.79205900	-1.13651100	-0.71784600
H	-4.51180600	-0.37876900	0.81075100

*

sm2-9beckm

C	0.10137900	1.28444600	0.23607600
N	1.16477600	1.98583500	0.25776300
O	2.43637000	1.04611700	0.12210300
H	3.16191200	1.59558400	0.46792800
H	2.36024400	0.21178700	0.62902400
C	-1.14873200	2.12748800	0.33963200
H	-1.68323200	1.77932400	1.23114600

H	-0.87356900	3.17215500	0.50322900
C	-2.02159900	1.96848200	-0.91118200
H	-2.31847100	0.92852800	-1.06708500
H	-2.92746800	2.56463000	-0.78766500
H	-1.50107500	2.32531700	-1.80320500
C	0.00760600	-0.19153200	0.11864100
C	-0.61086100	-0.92982700	1.12920000
C	0.49699300	-0.85307000	-1.01106900
C	-0.69081600	-2.31270100	0.96813700
H	-1.01559500	-0.45879800	2.01878700
C	0.34150300	-2.23782400	-1.07141200
H	0.95481900	-0.31492000	-1.83451100
H	-1.15343600	-2.92580300	1.73585700
H	0.69257600	-2.78862800	-1.93903600
N	-0.22978400	-2.95662800	-0.10477500

*

sm2-9bbeckm

C	0.68380100	0.98802400	0.45266700
N	1.82364200	0.57915900	0.86938800
O	1.74564900	-0.91927800	1.16762300
H	2.25550900	-1.41591700	0.40104000
H	2.24934500	-1.00556000	1.99403500
C	0.63687900	2.46840200	0.17744200
H	-0.13441400	2.88101900	0.83925100
H	1.59130100	2.91930700	0.45881500
O	2.89001300	-2.05702500	-0.72465300
H	3.72434100	-1.74655100	-1.09472800
H	2.80620200	-2.99944900	-0.90926900
C	0.28637900	2.76581200	-1.28547600
H	-0.68376000	2.34456500	-1.55968800
H	0.24064800	3.84647200	-1.43164400
H	1.04575100	2.36586500	-1.96278300
C	-0.52576900	0.16485400	0.20771500
C	-1.69131500	0.40402000	0.93258600
C	-0.53634800	-0.80954500	-0.79017600
C	-2.81270400	-0.37177000	0.64186000
H	-1.73609900	1.15885300	1.71038000
C	-1.72004500	-1.51003600	-1.00838400
H	0.33763300	-1.00260500	-1.40410100
H	-3.73637700	-0.22490100	1.19391300
H	-1.77249200	-2.26730300	-1.78552500
N	-2.83614200	-1.30808800	-0.30708500

*

sm2-8beckm

C	0.08716600	1.32847100	0.13339000
N	1.10450200	2.07375000	-0.07195000
O	2.35368000	1.18940100	-0.40654000
H	3.10474700	1.65988400	-0.00470900
H	2.26397800	0.27190400	-0.06022300
C	-1.15872300	2.12143800	0.44558700
H	-1.52630100	1.76443300	1.41438700
H	-0.90316500	3.17814500	0.55443500
C	-2.22181800	1.91442600	-0.64058000
H	-2.49303000	0.86079600	-0.74270000
H	-3.12033100	2.47307000	-0.37201300
H	-1.87032100	2.28348400	-1.60742600
C	0.05273900	-0.15192400	0.07847900
C	-0.37102700	-0.86945700	1.20516700
C	0.39722700	-0.82723500	-1.10329700
C	-0.42317800	-2.25771800	1.15377800
H	-0.64725000	-0.35037100	2.11764300
C	0.32175800	-2.21687200	-1.14659400
H	0.67747500	-0.27041900	-1.99324300
C	-0.08129900	-2.92987700	-0.01944100
H	-0.74074000	-2.81464700	2.02835100
H	0.56771800	-2.73838800	-2.06496800
H	-0.13932500	-4.01229900	-0.05774200

*

sm2-8bbeckm

C	0.68758600	0.92548100	0.44204300
N	1.83477900	0.47831500	0.80272300
O	1.73014300	-1.02892400	1.08717200
H	2.28513700	-1.50148700	0.34658400
H	2.19225800	-1.12336800	1.93624500
C	0.70145900	2.41521800	0.20137900
H	-0.03455200	2.85126900	0.88707300
H	1.68064500	2.81774300	0.47125600
O	3.00535800	-2.09672500	-0.77341800
H	3.79964200	-1.69808200	-1.14619900
H	3.01133600	-3.03813200	-0.97793800
C	0.33788900	2.75731900	-1.24804900
H	-0.65654700	2.38762100	-1.50886700
H	0.34443200	3.84143400	-1.37619300
H	1.06308000	2.33106500	-1.94663100
C	-0.55795900	0.16092000	0.21846400
C	-1.72636200	0.54403900	0.88442100
C	-0.58892500	-0.88754500	-0.70758800
C	-2.91143000	-0.14517900	0.64909900
H	-1.71225200	1.36140500	1.59852800
C	-1.78372900	-1.55313400	-0.95730900
H	0.30735800	-1.15708600	-1.25872400
C	-2.94222700	-1.18877300	-0.27329600
H	-3.81345300	0.14060700	1.17889000
H	-1.81244900	-2.35159600	-1.69074000
H	-3.87247300	-1.71221400	-0.46603300

*

sm2-6beckm

C	0.63214800	0.26491700	0.19680300
N	1.89443300	0.26074500	0.16074200
O	2.39145100	-1.32024600	-0.20971800
H	2.95451600	-1.19290700	-0.99329500
H	2.98299800	-1.54561900	0.52979500
C	0.12434800	1.66883000	0.48069900
H	-0.59280900	1.55412700	1.29923200
H	0.94930100	2.30032500	0.81858400
C	-0.33452900	-0.87039300	-0.04504800
H	-0.35333800	-1.04934700	-1.13496200
H	0.04956800	-1.78201400	0.43479000
O	-1.57339300	-0.49641800	0.44963500
C	-2.57935500	-1.48331800	0.24475500
H	-2.31543500	-2.42070300	0.74882800
H	-3.49324900	-1.08232700	0.67912500
H	-2.73143400	-1.66762900	-0.82559200
C	-0.54585400	2.25332600	-0.76875900
H	-1.39215600	1.64356300	-1.09055500
H	-0.92104000	3.24917900	-0.52422900
H	0.16802800	2.35509600	-1.58988900

*

sm2-6bbeckm

C	-0.72802200	0.53510500	0.13337200
N	0.38006600	1.12775600	-0.10233500
O	1.41204000	0.20844300	-0.56799300
H	2.31786900	0.62770500	-0.29084800
H	1.19394200	-0.73932400	-0.20873800
C	-1.86985200	1.42508600	0.53608000
H	-2.17448300	1.14239000	1.55067000
H	-1.50766600	2.45458700	0.57317200
O	3.58885500	1.22604800	0.11052400
H	3.59333900	1.93534600	0.76287900
H	4.27012600	1.42187600	-0.54219300
C	-0.99085900	-0.94883500	0.01710900
H	-1.77486500	-1.23814700	0.72512200
H	-1.34323700	-1.17171400	-1.00091000
O	0.20316000	-1.66633000	0.30918400
C	0.11126300	-3.08260800	0.09428800
H	-0.09803700	-3.29422800	-0.95868500
H	1.07288200	-3.50348000	0.38169400
H	-0.67593400	-3.49369000	0.73118400
C	-3.06107000	1.30527100	-0.42514300
H	-3.49166200	0.29923000	-0.43000200
H	-3.84809900	1.99370300	-0.11227300
H	-2.77270100	1.56619000	-1.44687400

*

sm2-5beckm

C	0.28936500	0.97309600	0.01151300
N	1.53430800	1.10325300	0.25203400
O	2.32782200	-0.06750200	-0.42145600
H	3.25844900	0.16208900	-0.24858500
H	2.09129100	-0.91778700	0.02227000
C	-0.53204100	2.09391200	0.59820900
H	-1.33007000	1.62527400	1.18535000
H	0.08933900	2.67821900	1.28031900
C	-0.38066700	-0.13663000	-0.76119200
H	-1.38314400	0.17891600	-1.05540100
H	0.17935200	-0.38514200	-1.66675300
C	-0.53704200	-1.40074100	0.07727900
F	-1.13355300	-2.35994300	-0.60595200
F	-1.20154900	-1.17442900	1.20598400
F	0.70538500	-1.88577600	0.45152600
C	-1.11546000	2.99393500	-0.50163500
H	-1.77289900	2.45051800	-1.18551200
H	-1.71002900	3.77864000	-0.03053200
H	-0.32178000	3.47186600	-1.08039600

*

sm2-5bbeckm

C	-0.00421000	0.91724000	0.01807300
N	1.24387400	0.72896400	0.22410000
O	1.71935000	-0.52788300	-0.42174800
H	2.77385600	-0.52155600	-0.26138000
H	1.29029200	-1.29617500	0.01411900
C	-0.54631500	2.19123900	0.60402200
H	-1.45088900	1.93580500	1.16709300
H	0.18363600	2.59699100	1.30767200
O	4.13634000	-0.50707500	-0.01008500
H	4.49847000	0.12959800	0.61728300
H	4.78908800	-0.66415200	-0.70206900
C	-0.92588800	-0.00114300	-0.75010100
H	-1.81894200	0.54717600	-1.05514500
H	-0.44036200	-0.39364700	-1.64715000
C	-1.39344500	-1.17942900	0.09329400
F	-2.20667200	-1.97069700	-0.58938800
F	-1.99831000	-0.78872200	1.21380400
F	-0.31718100	-1.95037500	0.48323900
C	-0.86735100	3.22276700	-0.48828900
H	-1.61234800	2.86027000	-1.20233900
H	-1.27400900	4.12150100	-0.02125700
H	0.03421400	3.50336500	-1.03813800

*

sm2-4beckm

C	0.65702100	0.18379600	0.10696700
N	1.87490600	-0.15922100	0.21374700
O	2.02480400	-1.74864800	-0.22866600
H	2.65858800	-1.72772000	-0.96835100
H	2.48307600	-2.15213600	0.53059800
C	0.52536300	1.64689900	0.50871600
H	-0.13694600	1.66734600	1.38212300
H	1.49732200	2.02562600	0.83466000
C	-0.53110600	-0.59896200	-0.35591500
H	-0.79333900	-0.21820500	-1.35084900
H	-0.28967600	-1.65650600	-0.45780000
C	-1.74859500	-0.44689200	0.56557300
H	-2.13165500	0.57712900	0.58108200
H	-1.52461200	-0.77135300	1.58654000
F	-2.73397100	-1.26399700	0.05882000
C	-0.03364700	2.49859100	-0.63645000
H	-1.04099000	2.19281400	-0.92942600
H	-0.08553600	3.53733000	-0.30486400
H	0.61657200	2.45969700	-1.51396800

*

sm2-4bbeckm

C	-0.19167500	0.62206000	0.34803700
N	0.90220200	0.50208400	1.00078600
O	1.46910500	-0.90893200	0.78108500
H	2.26411900	-0.81179100	0.09768700
H	1.83111700	-1.13816300	1.65304300
C	-0.81282100	1.98754400	0.49610400
H	-1.87159100	1.83311800	0.73422200
H	-0.35073300	2.51038900	1.33636900
O	3.27585100	-0.64644800	-0.88846700
H	3.90563900	0.08301600	-0.88976400
H	3.61863200	-1.35479700	-1.44480500
C	-0.88157600	-0.42396600	-0.47778200
H	-1.54018700	0.07152400	-1.19422200
H	-0.16705900	-1.03174800	-1.03585400
C	-1.73403900	-1.34362300	0.40825800
H	-2.48639500	-0.77992000	0.96798700
H	-1.11569300	-1.91972400	1.10256500
F	-2.38738300	-2.23093400	-0.41974400
C	-0.67444300	2.81245600	-0.79189400
H	-1.14400700	2.32515800	-1.65028900
H	-1.16274300	3.77856200	-0.65163600
H	0.37730900	2.99617900	-1.02488300

*

sm2-3beckm

C	0.20705000	0.59367500	0.13000400
N	1.44054300	0.26132700	0.12813000
O	1.59486100	-1.15980100	-0.45330600
H	2.50452800	-1.41262400	-0.22069400

H	0.91415000	-1.79298000	-0.05913000
C	-0.01967400	2.00297100	0.60855700
H	-0.73957000	1.95151700	1.43353200
H	0.91422400	2.41124200	1.00162700
C	-0.95305800	-0.28483400	-0.27227800
H	-1.88326500	0.26233500	-0.09782000
H	-0.89239900	-0.48899500	-1.34779000
C	-0.96422800	-1.57270700	0.52791100
C	-0.92639000	-2.79615900	-0.01341800
H	-1.02701000	-1.46439500	1.60951000
H	-0.96542000	-3.68281800	0.61111200
H	-0.90783900	-2.94841700	-1.09131000
C	-0.56147800	2.88716600	-0.52455400
H	-1.50563500	2.51211800	-0.92933100
H	-0.74292600	3.89033000	-0.13473700
H	0.16287900	2.96523700	-1.33870200

*

sm2-3bbeckm

C	0.28519900	0.82754700	0.11264800
N	0.97075200	-0.25342500	0.08211400
O	0.18212000	-1.37498600	-0.48839700
H	0.76233000	-2.22636500	-0.28789000
H	-0.72541700	-1.41524500	-0.08013800
C	1.03967000	2.02385300	0.62020500
H	0.46553300	2.44798300	1.45247200
H	2.00648400	1.70001200	1.01209700
O	1.58051500	-3.34925500	0.03294200
H	2.35644500	-3.20394200	0.58668400
H	1.80283200	-4.01713100	-0.62556000
C	-1.16588100	0.95329000	-0.28936700
H	-1.47966600	1.99060400	-0.14520100
H	-1.27000000	0.72617700	-1.35665100
C	-2.04193000	0.03985400	0.54305200
C	-2.84577000	-0.90089500	0.03712900
H	-2.00213500	0.19000900	1.62067800
H	-3.46866500	-1.51061100	0.68317300
H	-2.94274800	-1.05664800	-1.03584800
C	1.23045900	3.07676400	-0.48098600
H	0.27839200	3.43457700	-0.88333100
H	1.75741400	3.93888500	-0.06792100
H	1.82680200	2.67675200	-1.30465600

*

sm2-2beckm

C	0.15981700	0.21396900	0.09501300
N	1.18828200	0.92975400	0.26819600
O	2.55341100	0.05023100	-0.21375700
H	2.95207700	0.61493400	-0.89870600
H	3.12974700	0.10350700	0.56848200
C	-1.05299100	1.02853500	0.54086200
H	-1.48127100	0.48183900	1.38811900
H	-0.72614100	2.00160700	0.91607700
C	-0.00140000	-1.17109400	-0.45920600
H	-0.47096200	-1.04380600	-1.44256400
H	0.97834800	-1.61913300	-0.62124500
C	-0.87565200	-2.06929700	0.42486400
H	-1.89502000	-1.68898200	0.51918600
H	-0.93281300	-3.05895800	-0.03149500
H	-0.44893200	-2.18413700	1.42428400
C	-2.06757200	1.19405400	-0.59388600
H	-2.48068000	0.23704000	-0.92109000
H	-2.89474900	1.80928500	-0.23462200
H	-1.62383600	1.70029000	-1.45491700

*

sm2-2bbeckm

C	-0.61316800	0.06617700	0.19565000
N	0.52480800	0.29036600	0.73659900
O	1.53962300	-0.76102800	0.23802200
H	2.36427100	-0.18824600	-0.06272000
H	1.79504500	-1.24899400	1.03877800
C	-1.65418700	1.07336300	0.61762900
H	-2.54533600	0.50829400	0.91377300
H	-1.29993600	1.62342100	1.49224500
O	3.49428700	0.62047700	-0.41303600
H	3.57293300	1.53633300	-0.12407500
H	4.05073400	0.49011900	-1.18838800
C	-0.99540500	-1.05657200	-0.72362300
H	-1.84424400	-0.72471800	-1.32566600
H	-0.17439200	-1.29441400	-1.40206900
C	-1.39597800	-2.30399600	0.08557400
H	-2.20244000	-2.08446000	0.78973900
H	-1.74764600	-3.07831300	-0.59843200
H	-0.55025900	-2.70950700	0.64680700
C	-1.99309700	2.04260800	-0.52374400
H	-2.38433500	1.52686900	-1.40435000
H	-2.75798100	2.74334300	-0.18373400
H	-1.11402300	2.62062600	-0.81992100

*

sm2-1beckm

C	0.04587200	-0.04353200	0.21890700
N	0.91777300	0.86161000	0.04235400
O	2.37953700	0.13045100	-0.25429000
H	2.63942300	0.47811100	-1.12622200
H	2.97113900	0.53773600	0.40367100
C	-1.30141500	0.60772100	0.49496800
H	-1.56983200	0.30405200	1.51369400
H	-1.19551900	1.69528700	0.49249300
C	0.16641800	-1.52876100	0.16215000
H	-0.50895400	-1.97407800	0.89497700
H	-0.16070800	-1.85840800	-0.83019300
H	1.18484900	-1.87357000	0.32903800
C	-2.36121000	0.15417900	-0.51433900
H	-2.54110600	-0.92219100	-0.46481700
H	-3.30106300	0.65942600	-0.28413800
H	-2.08025800	0.42131000	-1.53629600

*

sm2-1bbeckm

C	-0.70190600	-0.36251600	0.31623600
N	0.34034500	0.02761000	0.94883300
O	1.55206900	-0.81346700	0.50551100
H	2.18673000	-0.14169200	0.01322800
H	1.96806300	-1.07117800	1.34507000
C	-1.92817500	0.43064400	0.69367100
H	-2.63563900	-0.28063400	1.13632900
H	-1.66671900	1.16028200	1.46329800
O	2.99591800	0.81522800	-0.68652300
H	3.06761200	1.74140700	-0.43138300
H	3.68279000	0.61343100	-1.33077100
C	-0.80905500	-1.43885500	-0.71412000
H	-1.79883200	-1.89589200	-0.66424700

H	-0.70708600	-0.98376400	-1.70548600
H	-0.04147200	-2.20297400	-0.60383400
C	-2.56276800	1.11968500	-0.52116600
H	-2.89233900	0.40002700	-1.27469100
H	-3.43889200	1.68378000	-0.19627300
H	-1.86562600	1.82144700	-0.98708400

*

sm2-10beckm

C	0.12382400	2.12053400	0.15385000
N	1.19071100	2.82933800	0.13366300
O	2.44231800	1.89191800	0.17832300
H	3.08383200	2.36822500	0.73351300
H	2.20557600	1.01125300	0.55625500
C	-1.12307700	2.97311500	0.14039700
H	-1.67916900	2.73533400	1.05458200
H	-0.84202200	4.02784000	0.19448400
C	-1.97716300	2.68578900	-1.09921600
H	-2.28532100	1.63825200	-1.14031700
H	-2.87577400	3.30491800	-1.06745000
H	-1.43287700	2.92740300	-2.01575200
C	0.04381300	0.64836100	0.17958400
C	-0.74428400	0.00481800	1.15445800
C	0.67200200	-0.12577000	-0.80849500
C	-0.85882500	-1.36814100	1.16161500
H	-1.25456700	0.58276500	1.91843900
C	0.54215500	-1.50968600	-0.81939000
H	1.22676600	0.35442600	-1.61029900
C	-0.21876300	-2.14208700	0.17432100
H	-1.44831700	-1.87774200	1.91521200
H	1.01885400	-2.07758000	-1.60843600
O	-0.40247700	-3.45853500	0.26634200
C	0.18396300	-4.32053000	-0.70588500
H	1.27494200	-4.23094500	-0.69749400
H	-0.10095600	-5.32840200	-0.41147900
H	-0.21059400	-4.10514600	-1.70384400

*

sm2-10bbeckm

C	1.75086200	0.47816200	-0.34251600
N	2.60862000	-0.47473500	-0.41461000
O	1.97075100	-1.79947900	-0.19509000
H	2.68682100	-2.36757100	0.32433800
H	1.11339100	-1.70089900	0.27730900
C	2.34436100	1.84913000	-0.53330000
H	2.16692100	2.41447800	0.38918300
H	3.42578300	1.75172100	-0.65548700
O	3.68677700	-3.08525500	1.03810500
H	4.50177800	-2.62178000	1.26386700
H	3.91520000	-3.99021300	0.79700100
C	1.71473500	2.57845700	-1.72528700
H	0.63947400	2.71636000	-1.58730400
H	2.17317000	3.56345800	-1.83296100
H	1.87897300	2.02698400	-2.65489000
C	0.30064300	0.31490600	-0.09963000
C	-0.31852400	1.01453400	0.95369000
C	-0.49238000	-0.46759200	-0.94982100
C	-1.67623000	0.90166000	1.16559300
H	0.27142900	1.63999200	1.61636900
C	-1.86597100	-0.57110600	-0.75475200
H	-0.04376800	-0.97186400	-1.80144500
C	-2.46645700	0.11026900	0.31193600
H	-2.16183200	1.42376000	1.98220200
H	-2.45285100	-1.16861300	-1.44096400
O	-3.77130400	0.07889300	0.59757000
C	-4.65298300	-0.66502600	-0.23637100
H	-4.39488500	-1.72920800	-0.22883800
H	-5.64353300	-0.52886800	0.19283400
H	-4.64063300	-0.27734500	-1.26018700

*

sm2-0beckm

C	0.07759700	-0.36149300	-0.03812200
N	1.00483000	0.40788900	-0.43099500
O	2.34944600	-0.08713400	0.31297900
H	2.97788500	-0.28512700	-0.40732500
H	2.68258000	0.70055200	0.78350900
C	-1.29062200	-0.09934100	-0.59122400
H	-1.56559700	-1.00256400	-1.14975300
H	-1.25813800	0.73502600	-1.29578100
H	0.23927300	-1.17083900	0.67499800
C	-2.29265100	0.15356800	0.54513000
H	-3.28494500	0.28957800	0.11172600
H	-2.33995600	-0.68949100	1.23935300
H	-2.04160200	1.05854000	1.10312600

*

sm2-0bbeckm

C	-0.71357800	-0.34138700	0.04183400
N	0.23254900	0.50988600	0.08284000
O	1.34997600	-0.08571200	0.92778700
H	2.21287000	-0.07890800	0.30441000
H	1.48782200	0.55447900	1.64758700
C	-1.94407000	0.00218400	-0.72947400
H	-2.04976000	-0.75860200	-1.51227700
H	-1.81674600	0.97214100	-1.21590400
H	-0.64673100	-1.29516200	0.56814800
O	3.34662200	-0.04030100	-0.50670900
H	3.42299700	0.54442600	-1.26917100
H	3.97351500	-0.76617600	-0.60066500
C	-3.18108900	-0.01105900	0.18172200
H	-4.06993700	0.19934700	-0.41562400
H	-3.32066500	-0.98467700	0.65964700
H	-3.10792100	0.75379800	0.95858900

*

sm1-9beckm

C	-0.43317700	1.45937000	0.21786200
N	0.44130500	2.37542000	0.04554600
O	1.82403600	1.74550700	-0.27902800
H	1.76769500	0.87127500	-0.72493200
H	2.27445200	2.40840000	-0.83258700
C	-1.79427000	1.99797900	0.54530600
H	-2.50580400	1.64860100	-0.20828000
H	-2.10203400	1.59368000	1.51371800
H	-1.80007200	3.08779600	0.57819600
C	-0.20449100	-0.00374400	0.10491900
C	-0.92578200	-0.74968900	-0.82976100
C	0.71221700	-0.65535400	0.93772100
C	-0.67840700	-2.12006100	-0.90066100
H	-1.65195600	-0.29161600	-1.49283400
C	0.86289800	-2.03448400	0.78698000
H	1.26487800	-0.12355200	1.70605500
H	-1.21249700	-2.73450500	-1.61924800

H	1.55172600	-2.58135600	1.42407600
N	0.19324700	-2.75274900	-0.11410800

*

sm1-9bbeckm

C	-1.50845900	0.74532400	-0.09969400
N	-1.48496000	1.84086100	0.56161100
O	-0.22006000	1.95552300	1.35481200
H	0.67514400	1.98762100	0.75282300
H	-0.35036700	2.78167900	1.84692300
C	-2.74675400	0.54251400	-0.91787600
H	-2.47532300	0.41812800	-1.97015000
H	-3.23665200	-0.37968900	-0.59222700
H	-3.43360000	1.38273300	-0.81481800
O	1.79391800	1.96495000	-0.04752100
H	2.57641900	2.50481700	0.11598700
H	2.07118700	1.10464600	-0.39125400
C	-0.44093800	-0.29041800	-0.11748200
C	-0.07705200	-0.97602700	1.04126400
C	0.20719700	-0.59927800	-1.31644200
C	0.93483000	-1.93146800	0.94884700
H	-0.56626000	-0.78581900	1.99012400
C	1.21684500	-1.56098600	-1.28873400
H	-0.05148800	-0.10627500	-2.24806500
H	1.23914700	-2.49083900	1.82875000
H	1.74864700	-1.82225700	-2.19946000
N	1.58388600	-2.21190100	-0.18220400

*

sm1-8beckm

C	-0.50187500	1.46656000	0.04658100
N	0.33458000	2.38571900	0.35560400
O	1.70574300	1.75809500	0.70791500
H	1.81350300	0.85268200	0.33074900
H	2.36669600	2.38500400	0.36471900
C	-1.85294500	2.00950000	-0.31537800
H	-2.10599100	1.69079300	-1.33036400
H	-2.59258200	1.58363900	0.36794800
H	-1.87824400	3.09815700	-0.25847400
C	-0.22937900	0.00989700	0.01482000
C	-0.45777900	-0.70541900	-1.16873200
C	0.22391300	-0.65463900	1.16705000
C	-0.20723400	-2.07231200	-1.20315000
H	-0.81651200	-0.19957200	-2.05947000
C	0.45285500	-2.02745600	1.12281500
H	0.35016100	-0.11472800	2.10162800
C	0.24444900	-2.73229700	-0.06061600
H	-0.37342400	-2.62374200	-2.12186500
H	0.78339000	-2.54400200	2.01711300
H	0.42518300	-3.80131300	-0.09144900

*

sm1-8bbeckm

C	-1.26606000	0.65406500	0.03280300
N	-0.94173100	1.84902800	0.37632700
O	0.48735600	1.85542300	0.91944500
H	1.04888500	2.41507500	0.24305200
H	0.40607900	2.35201000	1.75049700
C	-2.68622600	0.56398500	-0.44600200
H	-2.69363600	0.16429400	-1.46321500
H	-3.23258400	-0.13900200	0.18938500
H	-3.18322600	1.53388100	-0.41755900
O	1.77748000	3.12437800	-0.79026900
H	1.38560300	3.84648900	-1.29408500
H	2.73638500	3.21005900	-0.82507900
C	-0.41934300	-0.55797900	0.04065100
C	-0.87872100	-1.71273000	0.68296300
C	0.80129100	-0.57564100	-0.64489500
C	-0.10127800	-2.86618100	0.66923100
H	-1.82745700	-1.71147300	1.21011100
C	1.55839400	-1.74071100	-0.67726800
H	1.14120600	0.30391900	-1.18340200
C	1.11295800	-2.88243900	-0.01305000
H	-0.45094800	-3.75515600	1.18233300
H	2.49478300	-1.76006100	-1.22423900
H	1.70864500	-3.78871100	-0.03496400

*

sm1-6beckm

C	0.73333900	0.46305400	0.00007000
N	1.95757900	0.12615100	-0.05055900
O	2.00544400	-1.50160900	-0.31950100
H	2.54842300	-1.58515700	-1.12437700
H	2.53867700	-1.84124800	0.42210700
C	0.57749800	1.93661300	0.24425700
H	0.00114500	2.35371400	-0.58547100
H	-0.01155000	2.06273400	1.15633900
H	1.54064500	2.43856800	0.33585300
C	-0.48324200	-0.42050000	-0.14944900
H	-0.42409000	-0.92776200	-1.12686600
H	-0.44120100	-1.19969500	0.63002500
O	-1.60855300	0.37721200	-0.03638100
C	-2.82863700	-0.34753200	-0.15599000
H	-2.91125000	-1.10432600	0.63330800
H	-3.62823500	0.38264200	-0.04605100
H	-2.90305600	-0.82690400	-1.13944400

*

sm1-6bbeckm

C	-0.19425100	1.20226100	-0.46246800
N	1.02438500	0.81770800	-0.41947700
O	1.15052300	-0.62988100	-0.46236200
H	2.03588500	-0.85974000	0.02805500
H	0.27326400	-1.05142700	-0.10290900
C	-0.41305400	2.68209900	-0.50057500
H	-1.05573800	2.93955700	-1.34873000
H	-0.91939400	3.00633800	0.41348300
H	0.53502900	3.21016200	-0.59825400
O	3.27768100	-1.16767800	0.72119200
H	3.69446500	-0.49366500	1.26969400
H	3.97712900	-1.66354300	0.28124800
C	-1.41662500	0.31388200	-0.49772500
H	-2.25457500	0.82461000	-0.01132100
H	-1.68662100	0.12768100	-1.54825200
O	-1.14097700	-0.90603400	0.17993800
C	-2.18186400	-1.89051700	0.08316100
H	-2.35096500	-2.16241700	-0.96308200
H	-1.84375000	-2.75562800	0.65026700
H	-3.09715600	-1.49197700	0.52759500

*

sm1-5beckm

C	1.04707600	0.74051100	-0.31784900
N	2.13286000	0.35027600	0.22874100

O	2.20556900	-1.19746500	0.19989300
H	3.08664600	-1.39483700	0.56638900
H	1.49260600	-1.57207100	0.77471800
C	0.90194200	2.23111500	-0.34206500
H	0.81642000	2.55738100	-1.38332000
H	-0.02222100	2.50110300	0.17836600
H	1.74967700	2.72857900	0.12876800
C	-0.04386600	-0.12902400	-0.89752100
H	-0.67126800	0.47209900	-1.55849000
H	0.37514000	-0.95510200	-1.47842700
C	-0.95094000	-0.70680000	0.18383700
F	-1.90140900	-1.46027000	-0.33456300
F	-1.49128500	0.23911200	0.94429400
F	-0.21546300	-1.51592800	1.03823200

*

sm1-5bbeckm

C	-0.22095400	1.03415400	-0.28719900
N	1.00386200	0.76148800	-0.03694500
O	1.27753500	-0.69096900	-0.18234900
H	2.33681900	-0.77321300	-0.03877400
H	0.77736600	-1.18494500	0.50341200
C	-0.57724800	2.48155300	-0.17022600
H	-0.96001400	2.83785200	-1.13174500
H	-1.37487000	2.59600500	0.57040400
H	0.28356100	3.08129700	0.12444600
O	3.69035200	-0.85204300	0.18744600
H	4.16069700	-0.07782800	0.51902400
H	4.27895000	-1.34697000	-0.39436300
C	-1.29211500	0.03911400	-0.67127500
H	-2.11816900	0.56177600	-1.15751800
H	-0.90319100	-0.71081700	-1.36504300
C	-1.86930400	-0.68396500	0.53794200
F	-2.80097100	-1.55470200	0.18307500
F	-2.37453200	0.16036000	1.43569200
F	-0.88022800	-1.39386500	1.18761800

*

sm1-4beckm

C	0.69524700	0.51440300	-0.08904500
N	1.89483300	0.15348100	0.14595400
O	1.98382400	-1.46685000	0.05451500
H	2.63513000	-1.63355400	-0.65195300
H	2.39969000	-1.72417600	0.89825600
C	0.59047000	2.01784700	-0.00672500
H	0.13521400	2.38540100	-0.93058400
H	-0.06004900	2.27954900	0.83309000
H	1.56288000	2.48968700	0.13628400
C	-0.50552900	-0.30815700	-0.43982900
H	-0.69051900	-0.14969400	-1.51021100
H	-0.32000300	-1.37079300	-0.28639600
C	-1.76493500	0.10004800	0.33727200
H	-2.08568200	1.11778600	0.09990900
H	-1.62071200	-0.00087700	1.41752700
F	-2.76407900	-0.76529400	-0.04678900

*

sm1-4bbeckm

C	-0.12484200	1.03057000	0.07933500
N	0.89723100	0.89101800	0.83835400
O	1.28664200	-0.58532700	0.88866200
H	2.12513500	-0.69899300	0.25450600
H	1.56782200	-0.70773100	1.81073600
C	-0.58543100	2.45218700	-0.03350600
H	-0.47838700	2.77569700	-1.07338500
H	-1.64845000	2.50525800	0.22098000
H	-0.01803800	3.11817300	0.61641500
O	3.18460900	-0.80657400	-0.67229700
H	3.88004400	-0.14503500	-0.76072200
H	3.48941100	-1.63439600	-1.06068400
C	-0.87905800	-0.04851700	-0.64301900
H	-1.38824600	0.39601100	-1.50123700
H	-0.20734300	-0.82692600	-1.00932700
C	-1.92961000	-0.69360700	0.27299100
H	-2.66499200	0.03770300	0.62099500
H	-1.46526200	-1.18380000	1.13358300
F	-2.59036600	-1.65284800	-0.46306200

*

sm1-3beckm

C	0.58454300	0.78858700	-0.25350900
N	1.45636900	0.08559200	0.36423900
O	0.98795500	-1.37048500	0.43229900
H	1.57907800	-1.78252300	1.08568100
H	0.01476500	-1.43491400	0.70215700
C	0.98581200	2.22127400	-0.41991300
H	0.98888700	2.45912600	-1.48843700
H	0.24301700	2.86118700	0.06591600
H	1.97201400	2.41808600	0.00044600
C	-0.75029500	0.29912000	-0.76395900
H	-1.29122900	1.14723800	-1.19186400
H	-0.57850600	-0.42025300	-1.57377700
C	-1.57045800	-0.32351200	0.34910500
C	-2.03432300	-1.57913200	0.32972700
H	-1.79712300	0.32025000	1.19748000
H	-2.64082900	-1.96091500	1.14459100
H	-1.86809000	-2.24158100	-0.51825100

*

sm1-3bbeckm

C	-0.36650100	1.22115300	-0.37290100
N	0.82303400	0.78647000	-0.18123800
O	0.90031900	-0.67487900	-0.40093700
H	1.85465100	-0.94288000	-0.04620600
H	0.15855800	-1.14716000	0.06698800
C	-0.52386600	2.69956800	-0.21277500
H	-0.92739700	3.11690200	-1.14084700
H	-1.24732800	2.90503800	0.58246100
H	0.42445400	3.18310200	0.02094700
O	3.14091400	-1.25362900	0.46314500
H	3.63502800	-0.55674600	0.91056000
H	3.76487800	-1.78718100	-0.04215500
C	-1.56798400	0.36501000	-0.70145500
H	-2.44133900	1.01447500	-0.80521500
H	-1.40633800	-0.12449500	-1.66904800
C	-1.82184300	-0.66206200	0.38315400
C	-1.85916000	-1.98228600	0.17672100
H	-1.99385400	-0.26900600	1.38379700
H	-2.07016200	-2.66981400	0.98912400
H	-1.72953300	-2.41212500	-0.81500900

*

sm1-2beckm

C	0.00595900	0.45185700	-0.09008700

N	1.20800800	0.77836900	0.16702300
O	2.16055400	-0.56062100	0.04136100
H	2.81398000	-0.31782700	-0.63932000
H	2.62940800	-0.58196600	0.89507700
C	-0.87560000	1.67329800	0.02850800
H	-1.44778500	1.77333100	-0.89783000
H	-1.57045000	1.51702800	0.85769600
H	-0.30209000	2.58323600	0.20654800
C	-0.58591300	-0.86870000	-0.49045800
H	-0.83928000	-0.76158800	-1.55331500
H	0.16358600	-1.65551000	-0.41114400
C	-1.84652400	-1.22841500	0.30667200
H	-2.65359500	-0.51124900	0.14423600
H	-2.20210300	-2.20574700	-0.02416900
H	-1.63999000	-1.29175500	1.37795400

*

sm1-2bbeckm

C	-0.75978900	0.54435800	-0.19713100
N	0.33456400	0.70177600	0.45025900
O	1.08786300	-0.63466700	0.50088300
H	2.06849200	-0.38353700	0.21183100
H	1.10672900	-0.86999000	1.44385100
C	-1.56207800	1.80791700	-0.29200500
H	-1.67943900	2.07211800	-1.34703900
H	-2.55995600	1.62482000	0.11780400
H	-1.09447600	2.63418500	0.24335600
O	3.39611000	-0.00659600	-0.12863500
H	3.67623200	0.91554900	-0.12790400
H	3.97603100	-0.50781900	-0.71219600
C	-1.30993200	-0.71885600	-0.79361200
H	-1.93769000	-0.43822200	-1.64308800
H	-0.50029300	-1.34938100	-1.16440800
C	-2.15334900	-1.49071100	0.23783600
H	-2.97317700	-0.88096100	0.62541100
H	-2.58731400	-2.37130700	-0.23889200
H	-1.54775400	-1.82958700	1.08252200

*

sm1-1beckm

C	-0.43065100	-0.04903800	-0.00052300
N	0.40552600	0.91186500	-0.00212600
O	1.91349900	0.29170000	-0.00222500
H	2.33017600	0.69074900	-0.78808300
H	2.33118300	0.69294400	0.78197700
C	-1.83934700	0.49332100	-0.00023500
H	-2.35242100	0.11289800	-0.88768200
H	-2.35127800	0.11505500	0.88879400
H	-1.86545500	1.58325800	-0.00155800
C	-0.21451800	-1.52442400	0.00100500
H	-0.71094300	-1.94403800	0.88101300
H	-0.71154000	-1.94594000	-0.87776100
H	0.83501600	-1.80834200	0.00094500

*

sm1-1bbeckm

C	-1.11807400	-0.03492600	-0.01431400
N	-0.07885400	0.52966300	0.47949700
O	1.02693200	-0.50588200	0.68163700
H	1.86431500	-0.09062900	0.19122700
H	1.20181600	-0.49069700	1.63816900
C	-2.24092200	0.93433700	-0.24649500
H	-2.52803000	0.90207100	-1.30146400
H	-3.10703700	0.62316800	0.34476400
H	-1.96282800	1.95249600	0.02575700
O	2.98378700	0.50770300	-0.43354900
H	3.01189700	1.44810300	-0.64247100
H	3.61434700	0.04308300	-0.99457200
C	-1.32769600	-1.47044500	-0.36740700
H	-2.23793000	-1.82399100	0.12510600
H	-1.50532100	-1.54003100	-1.44532000
H	-0.49304500	-2.11300900	-0.09792000

*

sm1-10beckm

C	-0.25753900	2.33520000	0.20887300
N	0.67999900	3.15436300	-0.10298800
O	1.88215800	2.37140900	-0.69686400
H	1.59574400	1.50141600	-1.06532400
H	2.24278800	2.96033600	-1.38259500
C	-1.46673800	3.04415600	0.74960100
H	-2.32972800	2.81318600	0.11912200
H	-1.66995100	2.66827000	1.75540500
H	-1.32025800	4.12439000	0.77749500
C	-0.22962700	0.86763000	0.07454600
C	-1.29901900	0.19952200	-0.55571200
C	0.80838600	0.10816300	0.63934600
C	-1.30446300	-1.17468000	-0.64920400
H	-2.12033500	0.76050100	-0.98971800
C	0.79740800	-1.27949100	0.56684500
H	1.60849600	0.59545000	1.19042300
C	-0.25991400	-1.93191600	-0.08424800
H	-2.11396400	-1.69902100	-1.14405100
H	1.59914300	-1.83786600	1.03349800
O	-0.37161200	-3.25260800	-0.21240100
C	0.62145100	-4.10284500	0.35625800
H	0.68041500	-3.96129600	1.43993000
H	0.29538800	-5.11754900	0.13842900
H	1.59718800	-3.92402200	-0.10661400

*

sm1-10bbeckm

C	-1.09252800	0.67000500	-0.58120300
N	-0.65866400	1.87549600	-0.67947200
O	0.80670600	1.94352600	-0.48136300
H	0.97651200	2.86054600	0.01109100
H	1.13288400	1.15190800	0.00414700
C	-2.57743000	0.54116200	-0.73865500
H	-2.79172300	-0.12496500	-1.57856300
H	-3.00377400	0.08610000	0.15985100
H	-3.04421400	1.51205900	-0.90675500
O	1.13947100	4.09585600	0.67987500
H	0.34502000	4.59357800	0.90638700
H	1.81722300	4.72363600	0.40410400
C	-0.26204200	-0.52719700	-0.32241200
C	-0.58600400	-1.38802600	0.74394800
C	0.80722400	-0.86645100	-1.16377800
C	0.15639400	-2.52634600	0.97589900
H	-1.41490800	-1.15487200	1.40486800
C	1.54806500	-2.02388400	-0.94865200
H	1.04256600	-0.24629600	-2.02463300
C	1.22979300	-2.85984700	0.12977200
H	-0.07649600	-3.18976400	1.80100400
H	2.35134800	-2.26940400	-1.63186400

O	1.87684000	-3.98865400	0.43221300
C	2.96057500	-4.41314400	-0.38770000
H	3.32003500	-5.33843900	0.05764400
H	2.62293200	-4.60548400	-1.41123600
H	3.76467000	-3.67000400	-0.38649600

*

sm1-0beckm

C	-0.52887600	-0.36471600	0.06530000
N	0.39160600	0.44773500	-0.26008000
O	1.76833000	-0.33884700	-0.07995900
H	2.20160800	-0.30265100	-0.95474400
H	2.29640900	0.20820800	0.53323200
C	-1.92797700	0.14132000	-0.01988200
H	-2.48707900	-0.50418400	-0.70442100
H	-2.38684900	0.05257300	0.96979100
H	-1.97293000	1.17557600	-0.36146500
H	-0.32321400	-1.38426300	0.39252500

*

sm1-0bbeckm

C	-1.30743500	-0.42036100	0.04107400
N	-0.38124700	0.42299600	0.27478000
O	0.77308500	-0.32998500	0.90430500
H	1.61295600	-0.17074500	0.26390500
H	0.93763400	0.12595200	1.74836000
C	-2.56315700	0.06938300	-0.58585700
H	-2.72381600	-0.46427300	-1.52764700
H	-3.40360300	-0.17148400	0.07235200
H	-2.53246700	1.14363800	-0.76878800
H	-1.19122600	-1.47486100	0.29423000
O	2.71021500	0.04868800	-0.55484000
H	2.76864800	0.78495700	-1.17419100
H	3.34299400	-0.63232300	-0.80938300

*

sm0-9beckm

C	-0.92337000	1.37563500	0.04722400
N	-0.47844100	2.54736800	-0.17730400
O	1.09790600	2.47272800	-0.49644600
H	1.17458600	2.63287000	-1.45642800
H	1.44770500	3.26008100	-0.03843500
C	-0.42786800	0.00256200	0.08659800
C	-1.40291600	-1.00151800	0.03405400
C	0.91953300	-0.37222700	0.19046700
C	-0.98823800	-2.33151600	0.05660900
H	-2.46014600	-0.76537100	-0.03149000
C	1.21399000	-1.73138500	0.22283600
H	1.72095500	0.34806200	0.27187400
H	-1.71729500	-3.13419400	0.00578700
H	2.24396300	-2.06283200	0.31410100
N	0.29046500	-2.69289500	0.14914000
H	-1.99581300	1.52210300	0.23266700

*

sm0-9bbeckm

C	-1.04846100	1.19259300	-0.89749700
N	-0.80530100	2.35399300	-0.43117200
O	0.01995300	2.26644400	0.80933500
H	1.00522800	1.84854800	0.62923900
H	0.06098400	3.18660300	1.11578600
O	2.17864900	1.21693600	0.36638800
H	3.04272500	1.64462200	0.33758800
H	2.28394500	0.29771900	0.64280100
C	-0.65652900	-0.15570100	-0.45007400
C	-0.63183300	-0.54658200	0.89413400
C	-0.31800400	-1.09276600	-1.42824100
C	-0.23949700	-1.85026900	1.18788100
H	-0.94134700	0.11971600	1.69047000
C	0.08361700	-2.36328300	-1.01678100
H	-0.35242600	-0.84708600	-2.48476300
H	-0.22454300	-2.19741700	2.21723100
H	0.37261300	-3.11067700	-1.74951700
N	0.13225200	-2.73808800	0.26184900
H	-1.62514000	1.28471000	-1.81908000

*

sm0-8beckm

C	0.08119200	1.35535500	-0.92572400
N	0.19861300	2.55705900	-0.50903100
O	0.32674300	2.56433000	1.11156700
H	1.18185300	3.00489100	1.27010900
H	-0.37443800	3.18375300	1.38538600
C	0.02601500	-0.00193100	-0.42575600
C	-0.07795600	-1.00062000	-1.41402400
C	0.06534700	-0.37008000	0.93458400
C	-0.13651700	-2.33891000	-1.05483000
H	-0.11191400	-0.72649700	-2.46439200
C	0.00311700	-1.71006900	1.28057300
H	0.13838900	0.37370600	1.71496800
C	-0.09651400	-2.69238800	0.29210500
H	-0.21430900	-3.10205700	-1.82055900
H	0.03153900	-1.99442300	2.32647200
H	-0.14501900	-3.73824900	0.57645500
H	0.00573400	1.47841400	-2.01543700

*

sm0-8bbeckm

C	-0.89562200	0.48811800	-1.11219500
N	-0.59720100	1.72702600	-1.01341600
O	0.57581400	1.94593300	-0.14297100
H	0.48258600	2.96213500	0.19034000
H	0.62847400	1.28543200	0.57956100
O	0.30867100	4.26372000	0.58485800
H	-0.51122100	4.70435600	0.32979700
H	1.03076500	4.89906900	0.51099500
C	-0.29204400	-0.70017500	-0.49712300
C	-1.16237200	-1.69767400	-0.03226600
C	1.09870500	-0.87800200	-0.39060700
C	-0.64951800	-2.83620400	0.57589200
H	-2.23584300	-1.57629800	-0.14178400
C	1.60098300	-2.03162300	0.20241700
H	1.78553600	-0.15671700	-0.82331400
C	0.72991200	-3.00152500	0.69603400
H	-1.32503600	-3.60023100	0.94416000
H	2.67332800	-2.17910500	0.26745800
H	1.12827500	-3.89767500	1.15946400
H	-1.78599500	0.36897500	-1.73008200

*

sm0-6beckm

C	0.65348500	0.81534500	0.05060400
N	1.91434500	0.75428100	0.06501000
O	2.30688500	-0.81841300	-0.08012600

H 2.89192200 -0.85006500 -0.86087000
H 2.85978200 -1.00168400 0.70322600
C -0.42122000 -0.22512300 -0.07160900
H -0.29866000 -0.74168600 -1.03977100
H -0.28360800 -0.97650100 0.72494800
O -1.62471000 0.45343200 0.02939400
C -2.76381300 -0.39862800 -0.05213400
H -2.76029800 -1.13127800 0.76368600
H -3.63419000 0.24781700 0.04196700
H -2.79431700 -0.91612900 -1.01835000
H 0.34237600 1.86330500 0.14802800
*

sm0-6bbeckm
C -0.36968100 1.64140700 0.07530000
N 0.84358500 1.30959100 -0.11829500
O 0.97494800 -0.07236400 -0.51687200
H 1.91027500 -0.40574700 -0.18738100
H 0.12066700 -0.59264800 -0.23462300
O 3.19171100 -0.87020300 0.24980300
H 3.64374500 -0.43035100 0.97877400
H 3.85709200 -1.13543600 -0.39543900
C -1.61283100 0.80074600 0.00527800
H -2.30915500 1.11875100 0.78945200
H -2.09694400 0.95681200 -0.97046800
O -1.26262900 -0.56441300 0.19074300
C -2.33474700 -1.49823700 -0.01538700
H -2.72209600 -1.41060300 -1.03463100
H -1.91554600 -2.48924900 0.14670100
H -3.12505800 -1.30627800 0.71431800
H -0.47868300 2.69411800 0.32621600
*

sm0-5beckm
C 1.14826700 1.15866700 -0.09716600
N 2.15399300 0.49147800 0.29411500
O 2.01855300 -0.96310200 -0.16927800
H 2.82756500 -1.39965400 0.15682900
H 1.20115700 -1.37150600 0.22104400
C -0.06076300 0.72816000 -0.88097800
H -0.56982100 1.60823500 -1.27745000
H 0.22956800 0.08977400 -1.72114600
C -1.05915000 -0.03275200 -0.01209900
F -2.10329700 -0.43021700 -0.70856500
F -1.45279700 0.67338700 1.03753500
F -0.45370600 -1.17987500 0.49715200
H 1.25209400 2.19395600 0.23274300
*

sm0-5bbeckm
C 0.15492800 1.52205600 -0.06921700
N 1.34229300 1.13299800 0.16829400
O 1.60369900 -0.19498000 -0.40974600
H 2.64603400 -0.40569100 -0.16292300
H 0.97825100 -0.85294800 -0.03033700
O 3.91755800 -0.64784000 0.17159700
H 4.34716900 -0.08482100 0.82747400
H 4.56779000 -0.90630600 -0.49291800
C -0.93945100 0.81823700 -0.82392000
H -1.69809000 1.54151700 -1.12705900
H -0.54198900 0.33554900 -1.72154600
C -1.62798500 -0.24331000 0.02479300
F -2.57638000 -0.86810600 -0.65161500
F -2.13757700 0.26023800 1.14444600
F -0.70870800 -1.20229100 0.41104900
H -0.02869900 2.50468500 0.36172900
*

sm0-4beckm
C 0.69184000 0.86435900 -0.14224900
N 1.91947600 0.68555900 0.11234400
O 2.20448800 -0.90248000 0.10906600
H 2.86015800 -1.03751400 -0.60188900
H 2.65859300 -1.06402800 0.95823400
C -0.44926400 -0.04233400 -0.43989300
H -0.66553700 0.07532300 -1.51064000
H -0.19372700 -1.08615400 -0.25531100
C -1.71216100 0.35159900 0.34332000
H -1.99821400 1.38988600 0.14805400
H -1.58272400 0.19926500 1.41856100
F -2.72286700 -0.47048300 -0.09320900
H 0.52988900 1.95092500 -0.13633100
*

sm0-4bbeckm
C -0.12075600 1.12556800 -0.06674700
N 1.02750800 1.36093500 0.42929100
O 1.60366500 0.07447900 0.98637000
H 2.25781800 -0.33302500 0.24561100
H 2.12158600 0.37169100 1.75362400
O 3.01956900 -0.86394700 -0.78401300
H 3.65351400 -0.34501500 -1.29283900
H 3.25351100 -1.79728000 -0.84823600
C -0.90885600 -0.13378400 -0.17716300
H -0.74305200 -0.53178200 -1.18782300
H -0.56381700 -0.88259800 0.53774600
C -2.41101700 0.11054600 -0.00766900
H -2.78133200 0.85305200 -0.72173200
H -2.65648300 0.42444700 1.01086700
F -3.05116400 -1.08484300 -0.25625700
H -0.52861400 2.05236400 -0.47872300
*

sm0-3beckm
C 1.16517500 0.89551400 0.01696300
N 1.74685500 -0.21852800 0.20942400
O 0.80916600 -1.35470400 -0.15354800
H 1.19379300 -2.14501000 0.26504900
H -0.14937000 -1.17519100 0.13869300
C -0.24951900 1.17054300 -0.41017200
H -0.43962100 2.24198500 -0.30976900
H -0.34715700 0.92283800 -1.47474400
C -1.24524400 0.39003700 0.42778400
C -2.10514300 -0.50890300 -0.06832600
H -1.25602900 0.61444900 1.49323000
H -2.81622200 -1.01325200 0.57813300
H -2.15913400 -0.72865600 -1.13323300
H 1.85750400 1.70904900 0.23874000
*

sm0-3bbeckm
C -0.60632500 1.57104700 0.07397900
N 0.63054900 1.26652900 0.08013700
O 0.84638500 -0.09474200 -0.42615900

H	1.85293200	-0.33181500	-0.17424700
H	0.16630100	-0.73351600	-0.06999500
O	3.16986300	-0.60224700	0.18015300
H	3.62742800	0.01734900	0.76108500
H	3.80138400	-0.92746900	-0.47213400
C	-1.77591900	0.70628100	-0.30911900
H	-2.69807900	1.26481800	-0.13107600
H	-1.72250900	0.49834700	-1.38494100
C	-1.79406100	-0.58408400	0.48730600
C	-1.72910900	-1.80357800	-0.05710400
H	-1.88439900	-0.47911400	1.56696500
H	-1.77302900	-2.69394100	0.56160200
H	-1.67790100	-1.95011400	-1.13450000
H	-0.75557100	2.59210900	0.42151400

*

sm0-2beckm

C	0.02203500	0.77646800	-0.15918700
N	1.24587100	0.82323400	0.15745900
O	1.83135100	-0.69597500	0.12034000
H	2.54876100	-0.66302300	-0.53995200
H	2.24788200	-0.80649200	0.99550500
C	-0.93171700	-0.30869800	-0.52640000
H	-1.19339600	-0.13200400	-1.57748300
H	-0.44560500	-1.28238400	-0.45871600
C	-2.20085800	-0.24311000	0.33930200
H	-2.70508900	0.72120800	0.24010600
H	-2.89261200	-1.01933600	0.00870900
H	-1.97650000	-0.41548800	1.39417500
H	-0.34291400	1.81246200	-0.12425400

*

sm0-2bbeckm

C	-0.77332500	0.74827800	0.20656600
N	0.37242900	0.72616400	0.76113400
O	1.03722200	-0.62378700	0.49137500
H	1.96787800	-0.37471900	0.04415400
H	1.21667400	-0.98453700	1.37689900
O	3.20547000	-0.02030800	-0.50611900
H	3.57734100	0.86415600	-0.41280400
H	3.61738500	-0.45798000	-1.25930900
C	-1.54296500	-0.25422800	-0.58288600
H	-1.67518300	0.17903600	-1.58217900
H	-0.98326600	-1.18344000	-0.69119600
C	-2.92329500	-0.49957600	0.05031800
H	-3.49195800	0.42909800	0.14465900
H	-3.49423700	-1.18004800	-0.58345000
H	-2.83196200	-0.95334400	1.03993600
H	-1.23686400	1.72017600	0.39804100

*

sm0-1beckm

C	-0.65306300	0.62629300	0.00005700
N	0.58877000	0.87503400	0.00004300
O	1.38078000	-0.53367900	-0.00003100
H	1.95710000	-0.48698300	-0.78645500
H	1.95731200	-0.48655500	0.78620800
C	-1.46284300	-0.61597900	0.00002200
H	-2.11612600	-0.59986700	0.87836400
H	-2.11573700	-0.60004200	-0.87862000
H	-0.85688900	-1.51970300	0.00025400
H	-1.16242300	1.59869500	0.00002400

*

sm0-1bbeckm

C	-1.44225900	0.53874200	-0.30327400
N	-0.30739600	0.95301500	0.09962600
O	0.44034300	-0.19309700	0.76721300
H	1.38913700	-0.20886100	0.27864900
H	0.57752200	0.10301900	1.68409200
O	2.62978800	-0.18341600	-0.34464900
H	2.94564100	0.59010400	-0.82584200
H	3.09775800	-0.96489800	-0.65965400
C	-2.11268200	-0.78286300	-0.21885900
H	-3.07265800	-0.65300400	0.29064100
H	-2.34161800	-1.12081800	-1.23462200
H	-1.51820300	-1.53490800	0.29548700
H	-1.96683300	1.36453200	-0.78980200

*

sm0-10beckm

C	-0.73834000	2.39033800	-0.12784700
N	-0.09124900	3.49467700	-0.20952600
O	1.51327800	3.18120400	-0.23993400
H	1.80664700	3.62985700	-1.05315800
H	1.85103500	3.69784200	0.51353100
C	-0.52349200	0.97536400	-0.05471300
C	-1.69041600	0.17555500	0.03852800
C	0.73196600	0.33140600	-0.07032000
C	-1.60939100	-1.19265100	0.11578700
H	-2.66876500	0.64683900	0.05093000
C	0.82205300	-1.04393000	0.00516700
H	1.64857100	0.90005200	-0.14292100
C	-0.34945100	-1.82192800	0.10049900
H	-2.49775300	-1.80900000	0.18769100
H	1.79850100	-1.51084400	-0.01091800
O	-0.36605200	-3.14538700	0.18066100
C	0.85846900	-3.87947400	0.16673000
H	1.48639500	-3.60070100	1.01821500
H	0.57030300	-4.92480700	0.25248100
H	1.39157900	-3.72043300	-0.77522400
H	-1.78492000	2.72737000	-0.11333400

*

sm0-10bbeckm

C	-1.19254100	1.65806700	0.64494700
N	-0.64823500	2.81498100	0.79549000
O	0.87310200	2.71489400	0.63656800
H	1.09588300	3.57055200	0.06625900
H	1.22336100	2.85002300	1.53371200
O	1.32234600	4.77842400	-0.65248100
H	0.59537400	5.40423700	-0.75009700
H	1.89160900	4.84629800	-1.42669800
C	-0.78419900	0.30028700	0.38186600
C	-1.81944800	-0.66491000	0.40208200
C	0.52647100	-0.13824700	0.10715800
C	-1.56248500	-1.99562600	0.17236500
H	-2.84071200	-0.35585000	0.60524500
C	0.79477500	-1.47402000	-0.13029300
H	1.34693000	0.56405100	0.06500500
C	-0.24788400	-2.41754600	-0.09605100
H	-2.35279200	-2.73674800	0.19066900
H	1.81220700	-1.77837600	-0.34126200
O	-0.09122800	-3.72206800	-0.30655600

C 1.20410200 -4.24550200 -0.58745800
H 1.06169100 -5.31589400 -0.71941300
H 1.60714500 -3.81297700 -1.50855500
H 1.88633900 -4.06568100 0.24925100
H -2.26698300 1.81400600 0.77610700
*

sm0-0beckm
C -1.22091000 -0.14504600 0.06758200
N -0.21980600 0.46460200 -0.39708400
O 1.04810800 -0.42488100 -0.04482600
H 1.47997100 -0.61527800 -0.90186900
H 1.64435300 0.16100100 0.46345200
H -1.20669700 -1.09364100 0.60157700
H -2.15539500 0.38724700 -0.12191400
*

sm0-0bbeckm
C -1.89306400 -0.30608000 -0.53599900
N -0.96431900 0.49397400 -0.22039500
O -0.05636200 -0.21673100 0.75080700
H 0.93813900 -0.17371000 0.31130700
H -0.06508300 0.33224800 1.55563200
H -1.99885300 -1.31623800 -0.14477200
H -2.60823300 0.10118400 -1.24778000
O 2.16757400 -0.11028900 -0.23821700
H 2.41489000 0.56178100 -0.88437900
H 2.76168700 -0.86607000 -0.31395500
*

prod7-0beckm
C 0.00573200 -2.73341600 0.18815400
N 0.00362700 -1.72689100 -0.34753300
C 0.00113700 -0.43460200 -0.98739200
H -0.89173600 -0.42428900 -1.62014000
H 0.89385800 -0.42105400 -1.62029300
H 0.00783400 -3.68890600 0.68734900
C -0.00086400 0.72737300 0.03827000
C -0.00275300 2.00681100 -0.81382500
H -0.89178800 2.06990500 -1.44922900
H -0.00429900 2.87850800 -0.15478100
H 0.88634500 2.07278300 -1.44883900
C -1.26423100 0.66437900 0.90431100
H -2.17455800 0.69152700 0.29673700
H -1.29152700 -0.23202000 1.53424300
H -1.29033100 1.52467900 1.57763900
C 1.26248100 0.66832800 0.90463000
H 2.17287100 0.69799900 0.29726600
H 1.28592500 1.52887400 1.57774200
H 1.29224000 -0.22782600 1.53479000
*

prod6-9beckm
C -0.40494500 -0.04936100 0.25148700
N -0.62006100 -0.06818500 1.38016700
C -0.76197300 -0.08497100 2.81706500
H -1.25143600 -1.03761200 3.06623600
H -1.43004700 0.75018300 3.07299400
C -0.15604200 -0.02347600 -1.14714600
C 0.00897000 1.20710300 -1.79572200
C -0.07473300 -1.22708500 -1.85918700
C 0.25330700 1.16566500 -3.16558200
H -0.04761400 2.14998300 -1.26444900
C 0.17203500 -1.13094800 -3.22572300
H -0.19693700 -2.19008600 -1.37740000
H 0.39141600 2.08675100 -3.72289500
H 0.24422700 -2.02850500 -3.83185300
N 0.33164300 0.03069300 -3.86226400
O 0.50796300 0.04132100 3.32961500
C 0.52307600 0.08506400 4.76028300
H 0.11617700 -0.84051100 5.18229100
H 1.56936900 0.18652900 5.03990200
H -0.04191900 0.94902100 5.12772600
*

prod6-8beckm
C -0.40353000 0.04388000 0.25635500
N -0.61656400 0.06616300 1.38844000
C -0.71045900 0.08235500 2.82392300
H -1.36290000 -0.75690500 3.10524100
H -1.19818400 1.03013300 3.09382300
C -0.16757100 0.01655700 -1.13369700
C -0.00585800 1.23267300 -1.82478800
C -0.09255300 -1.22713000 -1.78959500
C 0.23062400 1.19055000 -3.18914400
H -0.06509500 2.17926200 -1.29901300
C 0.14577700 -1.24086900 -3.15421500
H -0.21732100 -2.15212500 -1.23747700
C 0.30580300 -0.03893600 -3.84732700
H 0.35850000 2.11481500 -3.74050800
H 0.20895200 -2.18690500 -3.67919800
H 0.49194000 -0.06117100 -4.91592500
O 0.57565300 -0.03398400 3.30395000
C 0.62799800 -0.06366200 4.73205300
H 0.07704700 -0.92627800 5.12395000
H 1.68139500 -0.15738800 4.98716300
H 0.22711400 0.86361100 5.15693700
*

prod6-6beckm
C 0.19346900 0.45608300 -0.53646700
N -0.11344000 0.50710500 0.56387900
C -0.44680500 0.50553900 1.97399900
H -1.48637700 0.15218900 2.03767800
H -0.38712200 1.55208900 2.30595700
C 0.57059500 0.38208200 -1.96077600
H 0.72277100 1.41697700 -2.31025500
H 1.54189900 -0.13968900 -2.00041600
O -0.45753700 -0.28612900 -2.60398500
C -0.20996600 -0.45702600 -4.00438900
H 0.69214000 -1.05731600 -4.16579700
H -1.07804200 -0.98322300 -4.39536000
H -0.11222200 0.51530800 -4.49975000
O 0.46692400 -0.32628500 2.57346700
C 0.24387300 -0.46383100 3.98211100
H -0.74360400 -0.89675400 4.17575600
H 1.01678300 -1.14176900 4.33701300
H 0.34131900 0.50417600 4.48546700
*

prod6-5beckm
C 0.43394800 -0.18306700 0.05628100
N -0.06772400 -0.20377200 1.08330500
C -0.63576900 -0.20664000 2.42104200

H	-1.42809600	0.55575800	2.41324800
H	-1.07604200	-1.20418200	2.56275100
C	1.05249400	-0.15704100	-1.25926300
H	1.78968700	0.65246000	-1.28220500
H	1.55800500	-1.11314500	-1.42987700
C	-0.00962600	0.07320000	-2.35525600
F	0.58629300	0.08068000	-3.53730600
F	-0.92321700	-0.90105400	-2.32139900
F	-0.62822500	1.24040500	-2.15874900
O	0.41031500	0.07492100	3.26333400
C	0.01265500	0.12833800	4.64042100
H	-0.38359300	-0.83937600	4.96621300
H	0.91791700	0.36118900	5.19639500
H	-0.73142900	0.91677600	4.79676900

*

prod6-4beckm

C	0.14917400	-0.12488100	-0.51362000
N	0.17628000	0.29426400	0.55242200
C	0.19230700	0.70592100	1.94411600
H	-0.57161800	1.49071500	2.04146300
H	1.18965500	1.13074200	2.12741800
C	0.11651600	-0.63550400	-1.86916300
H	1.07059400	-1.14106300	-2.05734900
H	-0.68415300	-1.38272300	-1.91912900
C	-0.11915500	0.47194400	-2.92481300
H	0.68567000	1.21099700	-2.91068000
H	-1.08744800	0.95742100	-2.78009500
F	-0.11736400	-0.16364200	-4.13704500
O	-0.07646600	-0.43021100	2.66726400
C	-0.11544300	-0.18808800	4.07924900
H	-0.91043200	0.52331900	4.32787900
H	-0.32818500	-1.15147200	4.53692800
H	0.85144400	0.18374800	4.43566100

*

prod6-3beckm

C	-0.30793000	-0.34300700	-0.66833100
N	-0.37914200	0.12358700	0.37605500
C	-0.42432000	0.63307600	1.72980000
H	0.07877800	1.61042800	1.70882700
H	-1.48767500	0.76758400	1.97431900
C	-0.19033500	-0.89660300	-2.01100700
H	0.30240000	-1.87161500	-1.90176300
H	-1.20751000	-1.07442700	-2.37812000
C	0.58795100	0.04155800	-2.91760600
C	0.02471200	0.65583500	-3.95271900
H	1.64443100	0.15745600	-2.69394300
H	0.61529200	1.29178900	-4.60293700
H	-1.02741200	0.53764900	-4.19774200
O	0.21378200	-0.30591700	2.50508200
C	0.26408600	0.06259900	3.88736600
H	-0.74584200	0.15688800	4.30169200
H	0.79083800	-0.74636800	4.38871600
H	0.81521200	1.00030400	4.01909900

*

prod6-2beckm

C	0.42554600	0.16232800	-1.15743500
N	0.53245600	-0.18792400	-0.07113100
C	0.59586900	-0.53965000	1.33186400
H	0.35693200	-1.61088800	1.39560700
H	1.63578600	-0.37144400	1.64637200
C	0.26865000	0.58762200	-2.53776400
H	-0.19505900	1.58035000	-2.49536700
H	1.27624400	0.71234400	-2.95019000
C	-0.57502900	-0.40061300	-3.36624500
H	-1.57258000	-0.52296900	-2.94173500
H	-0.67660700	0.01016300	-4.37150400
H	-0.08822600	-1.37417600	-3.43898500
O	-0.31628800	0.27403500	1.96131000
C	-0.39892000	0.02418100	3.36878800
H	0.56788500	0.20251800	3.85217700
H	-1.13442400	0.72923000	3.74973400
H	-0.73539600	-1.00067600	3.56043500

*

prod6-1beckm

C	-0.08573400	-0.00139900	-1.75831200
N	-0.42604200	-0.00177000	-0.66499600
C	-0.75877000	-0.00216600	0.74652900
H	-1.36161500	-0.90490600	0.92061800
H	-1.36999000	0.89518900	0.91925500
C	0.33946000	-0.00046600	-3.14055000
H	1.10989900	0.76458900	-3.27280700
H	-0.51932200	0.22453900	-3.77934700
H	0.74431700	-0.98475700	-3.39109000
O	0.44350100	0.00380400	1.41148900
C	0.28252500	0.00221400	2.83502300
H	-0.24633100	-0.89912900	3.16374200
H	1.28945000	0.00608900	3.24619100
H	-0.25369500	0.89876800	3.16489000

*

prod6-10beckm

C	-0.53946900	-0.18732600	1.23829300
N	-0.76765900	-0.18681300	2.37261200
C	-0.74474300	-0.11981600	3.80504200
H	-1.08083100	-1.09995200	4.17299800
H	-1.47742900	0.64566700	4.09802200
C	-0.31461900	-0.19705200	-0.13921200
C	-0.41941600	1.00556400	-0.86917900
C	0.02417600	-1.40873500	-0.79242100
C	-0.19504700	1.00532400	-2.22938200
H	-0.67364800	1.93116000	-0.36398300
C	0.24478500	-1.40368900	-2.14521100
H	0.10853800	-2.33138000	-0.22883700
C	0.13715100	-0.20088100	-2.87995900
H	-0.27700100	1.93331600	-2.78027100
H	0.50436700	-2.31164400	-2.67659000
O	0.36693400	-0.30841900	-4.17796000
C	0.28992300	0.84378100	-5.02159300
H	1.03569900	1.58653500	-4.72479600
H	0.50934400	0.48103200	-6.02303700
H	-0.71571200	1.27290800	-4.99605000
O	0.54808600	0.19089000	4.17616300
C	0.69575100	0.30003300	5.59217000
H	0.45872900	-0.65058900	6.08388900
H	1.74085200	0.54788600	5.76566700
H	0.05759600	1.09728700	5.99085200

*

prod6-0beckm

C	-0.00003500	-2.39364700	0.33581000

N	-0.00004600	-1.36093300	-0.14417900
C	-0.00001100	-0.00645800	-0.68980000
H	-0.90187000	0.05868000	-1.31642500
H	0.90188200	0.05865700	-1.31637700
H	0.00013700	-3.37443300	0.78756300
C	0.00001800	2.21170800	0.04876600
H	-0.90016100	2.46396500	-0.52131000
H	-0.00000100	2.74841900	0.99448200
H	0.90024900	2.46391900	-0.52124900
O	-0.00002900	0.81892700	0.40174500

*

prod5-9beckm

C	-0.60218400	-0.04224100	-0.33446600
N	-0.88073900	-0.06255700	0.78122100
C	-1.12984500	-0.08808900	2.18433000
H	-1.58942400	-1.04647400	2.43615800
H	-1.80916600	0.72977000	2.43489000
C	0.19503700	0.07603600	2.94722600
C	-0.26734600	-0.02157100	-1.71285300
C	-0.04791300	1.20542000	-2.35364600
C	-0.15405400	-1.23072400	-2.41286200
C	0.28323400	1.15389400	-3.70482600
H	-0.12798000	2.15163700	-1.83133200
C	0.18118500	-1.14314500	-3.76087500
H	-0.31594500	-2.19039100	-1.93606100
H	0.46723300	2.07043600	-4.25634100
H	0.28326500	-2.04422400	-4.35730900
N	0.39395200	0.01425500	-4.38954700
F	-0.06017800	0.03484600	4.24844500
F	0.76720900	1.24400200	2.64082600
F	1.04300000	-0.90519000	2.62665000

*

prod5-8beckm

C	-0.61242500	-0.03257500	-0.33330200
N	-0.89810600	-0.04660200	0.78373600
C	-1.12897800	-0.06610800	2.18770200
H	-1.60579500	-1.01354700	2.44850300
H	-1.78867400	0.76433900	2.44891700
C	0.20339500	0.07061800	2.93941900
C	-0.27921000	-0.01819000	-1.70101400
C	-0.05943300	1.21875300	-2.33895800
C	-0.17032700	-1.24264300	-2.39000200
C	0.27125500	1.21636400	-3.68385900
H	-0.14578800	2.14923600	-1.78864800
C	0.16057700	-1.21491100	-3.73447400
H	-0.34090100	-2.18338300	-1.87817500
C	0.37925300	0.00716300	-4.37495000
H	0.44572400	2.15570700	-4.19546500
H	0.25020400	-2.14445200	-4.28448200
H	0.63730300	0.01741100	-5.42871400
F	-0.04014000	0.03488900	4.24466900
F	0.79886600	1.22702900	2.63069200
F	1.03099000	-0.92733200	2.61475600

*

prod5-6beckm

C	0.05210500	0.48693500	-1.03150900
N	-0.33014900	0.61758400	0.03887800
C	-0.78103700	0.75819800	1.38634800
H	-1.71918500	0.20811600	1.49076700
H	-0.93797000	1.81965000	1.58981600
C	0.51597500	0.30897200	-2.42208700
H	0.88797200	1.28892300	-2.76607800
H	1.36940900	-0.38938000	-2.38335700
O	-0.57531000	-0.16572100	-3.12887100
C	-0.27762600	-0.39907900	-4.51231700
H	0.51408800	-1.14922900	-4.61364200
H	-1.19925900	-0.77520100	-4.95091800
H	0.01462000	0.53445000	-5.00496900
C	0.27726000	0.18632300	2.34536700
F	-0.15800900	0.33909900	3.58789800
F	1.43717700	0.83209300	2.19345400
F	0.48069500	-1.10935000	2.09623500

*

prod5-5beckm

C	0.27268000	-0.19883200	-0.49239800
N	-0.30631800	-0.20767100	0.49430300
C	-0.98599600	-0.20982200	1.75181800
H	-1.73593900	0.58430300	1.73739900
H	-1.46195100	-1.18421700	1.88345600
C	0.98511500	-0.18731400	-1.75875100
H	1.72882600	0.61665300	-1.73523600
H	1.49364300	-1.14932700	-1.88436600
C	0.00108300	0.04168500	-2.92861600
F	0.67690800	0.03774000	-4.06517300
F	-0.91756100	-0.92758800	-2.94822500
F	-0.62136100	1.21335800	-2.77908400
C	0.03372500	0.04044500	2.87825400
F	-0.61057700	0.02663300	4.03502600
F	0.63039000	1.22279300	2.70778100
F	0.97138700	-0.91059700	2.87253600

*

prod5-4beckm

C	0.44698500	0.06759100	-1.07713700
N	0.46160800	0.51317500	-0.02068700
C	0.46891300	1.00175800	1.32184800
H	-0.10962200	1.92756900	1.35751200
H	1.50427900	1.18978400	1.61482100
C	0.41935400	-0.48076000	-2.41630800
H	1.45704600	-0.59591800	-2.75148200
H	-0.03160100	-1.47855400	-2.34979600
C	-0.37400900	0.39875300	-3.41576500
H	0.08309000	1.38550300	-3.52402200
H	-1.42166200	0.49040800	-3.11883300
F	-0.30587300	-0.25629200	-4.61452800
C	-0.16112200	-0.04909100	2.25283200
F	-0.13860200	0.42202800	3.49112700
F	-1.42264000	-0.29432800	1.88795600
F	0.52492500	-1.19323600	2.19388800

*

prod5-3beckm

C	-0.18848500	-0.17682400	-1.18124100
N	-0.18591300	0.39439500	-0.18654700
C	-0.18141300	1.04667800	1.08303200
H	0.61654900	1.79215700	1.08867200
H	-1.15059300	1.52843200	1.22926600
C	-0.16815700	-0.86019000	-2.46697400
H	0.34796100	-1.81606600	-2.30799600
H	-1.21030700	-1.07969900	-2.72728900

C	0.51750000	-0.00174800	-3.51794500
C	-0.13956600	0.51262800	-4.55213200
H	1.58743000	0.13819200	-3.39533100
H	0.38649700	1.08910800	-5.30520400
H	-1.20655500	0.36669600	-4.69752900
C	0.06067900	0.00848300	2.19086700
F	0.05315700	0.63307800	3.36090400
F	1.23918200	-0.59577300	2.01392000
F	-0.89661800	-0.92314600	2.17479200

*

prod5-2beckm

C	0.27007900	0.07147800	-1.60881100
N	0.40215000	-0.40486300	-0.57360500
C	0.54557100	-0.93508000	0.74432200
H	0.00122700	-1.88017200	0.80096400
H	1.60768200	-1.09773600	0.93960000
C	0.09536800	0.64610600	-2.93060600
H	-0.65386400	1.43978700	-2.82393700
H	1.04718600	1.12161400	-3.19412500
C	-0.33163400	-0.40716600	-3.97346200
H	-1.28664300	-0.86366100	-3.70969400
H	-0.44714500	0.10323400	-4.93032600
H	0.42628400	-1.18362700	-4.08693200
C	-0.02879800	0.06299700	1.76333200
F	0.11717200	-0.44720500	2.97861000
F	0.61579400	1.23049800	1.68701900
F	-1.32470700	0.28109100	1.52194000

*

prod5-1beckm

C	-0.21058200	0.00447500	-2.13584700
N	-0.64166000	0.00607700	-1.07413700
C	-1.11424700	0.00802500	0.27440300
H	-1.72798100	-0.88208800	0.42832900
H	-1.70634100	0.91188700	0.43293100
C	0.33509500	0.00116600	-3.47388000
H	1.07895200	0.79946500	-3.55452100
H	-0.47213700	0.16949800	-4.19274200
H	0.80692500	-0.96781000	-3.66182800
C	0.08842400	-0.00880200	1.23405500
F	-0.36870900	-0.00252200	2.47810300
F	0.85849000	1.06393500	1.03302600
F	0.82800900	-1.10266400	1.03284700

*

prod5-10beckm

C	-0.68199900	-0.34988300	0.59729400
N	-0.92426200	-0.38664800	1.72849200
C	-1.01105600	-0.36611400	3.14682200
H	-1.17048600	-1.38599100	3.50383600
H	-1.85545300	0.26049500	3.44257000
C	0.28411300	0.19577600	3.74830200
C	-0.42136900	-0.30752100	-0.77103700
C	-0.50235800	0.92605300	-1.45312900
C	-0.06740900	-1.49403500	-1.46363400
C	-0.23615200	0.98099900	-2.80379400
H	-0.76902800	1.83129800	-0.91815800
C	0.19295900	-1.43297200	-2.80721800
H	-0.00256400	-2.43973800	-0.93693200
C	0.11294800	-0.19913500	-3.49398100
H	-0.29834600	1.93118100	-3.31806000

H	0.46527300	-2.31910400	-3.36830700
F	0.16892300	0.21508100	5.07258300
O	0.38441400	-0.25333200	-4.78544900
C	0.34265500	0.93298000	-5.58462100
H	1.08283800	1.65737100	-5.23349500
H	0.59371700	0.60908000	-6.59190800
H	-0.66083000	1.36734900	-5.57559200
F	0.50529300	1.43977500	3.30721900
F	1.33262300	-0.56025400	3.40701700

*

prod5-0beckm

C	0.00013000	-2.64404700	0.25025000
N	0.00042700	-1.65708400	-0.31898600
C	0.00091900	-0.39855900	-1.00073500
H	-0.89740400	-0.35113100	-1.62132900
H	0.90082800	-0.35057500	-1.61897500
H	-0.00016000	-3.58269900	0.78669900
C	-0.00078400	0.74361700	0.03751900
F	0.00003000	1.89293600	-0.61817400
F	1.08161000	0.66081500	0.81207500
F	-1.08545000	0.66051400	0.80884800

*

prod4-9beckm

C	-0.33960500	0.06563700	0.24047400
N	-0.50161100	0.10290700	1.37832800
C	-0.68187600	0.14845600	2.79878100
H	-1.41228800	-0.62095000	3.06097300
H	-1.09342300	1.13071700	3.04405000
C	0.65237700	-0.08617300	3.52442400
H	1.05445900	-1.07930800	3.30658300
H	1.38396600	0.68471200	3.26758700
C	-0.14324600	0.01636500	-1.16721300
C	0.19575500	1.18480000	-1.86171400
C	-0.29076200	-1.20242900	-1.84181000
C	0.37519300	1.06622100	-3.23720300
H	0.31491600	2.13895100	-1.36175800
C	-0.08374600	-1.18593100	-3.21840200
H	-0.55265100	-2.11893400	-1.32600900
H	0.63943300	1.93634500	-3.83008600
H	-0.18313700	-2.09967000	-3.79576700
N	0.24036700	-0.08474600	-3.89803500
F	0.36587800	-0.00383200	4.86326200

*

prod4-8beckm

C	-0.34449200	-0.04775100	0.24190600
N	-0.51216100	-0.08177700	1.38156600
C	-0.69428700	-0.12615600	2.80030300
H	-1.13360500	-1.09652300	3.04453600
H	-1.40341100	0.66118900	3.06743000
C	0.64403500	0.06772800	3.52693300
H	1.35481200	-0.72068400	3.26468600
H	1.07351700	1.05049000	3.31438700
C	-0.14269300	-0.00693600	-1.15525900
C	-0.22013300	1.23081300	-1.82146500
C	0.12980000	-1.20522700	-1.84238300
C	-0.01995300	1.25676000	-3.19220400
H	-0.43207400	2.14265300	-1.27390500
C	0.32595800	-1.15088500	-3.21291100
H	0.18421500	-2.14871200	-1.31036100

C	0.25146800	0.07287000	-3.88148100
H	-0.07540200	2.19885500	-3.72514000
H	0.53660600	-2.06171300	-3.76132100
H	0.40712700	0.10488400	-4.95465200
F	0.36025100	-0.01400300	4.86791800

*

prod4-6beckm

C	0.09761000	0.71067900	-0.56058000
N	-0.07673900	0.70953500	0.57062600
C	-0.29331600	0.66939800	1.98798700
H	-1.36733600	0.77841200	2.15636700
H	0.23132300	1.52408800	2.42213800
C	0.30513800	0.68439700	-2.02296600
H	-0.21116600	1.56636600	-2.43756600
H	1.38708800	0.80656700	-2.19796300
O	-0.20593800	-0.52711400	-2.45822100
C	-0.07349200	-0.70640400	-3.87419400
H	0.98291700	-0.70777900	-4.16420000
H	-0.51675400	-1.67628900	-4.08898900
H	-0.61632400	0.07715800	-4.41405800
C	0.22919600	-0.65434900	2.57184100
H	1.30626500	-0.75907100	2.41588600
H	-0.30204100	-1.51102400	2.14881400
F	-0.02510100	-0.58604900	3.91710700

*

prod4-5beckm

C	0.47105700	-0.15943000	0.04297700
N	-0.00383200	-0.14708200	1.08433300
C	-0.59408700	-0.13258100	2.39285400
H	-1.34354500	0.66273000	2.39877600
H	-1.08437200	-1.09875100	2.53593000
C	1.04895000	-0.17803400	-1.29196100
H	1.79714300	0.61813600	-1.36575300
H	1.53462700	-1.14646900	-1.45175100
C	-0.04501800	0.03612700	-2.36107700
F	0.51502500	0.00577500	-3.55978700
F	-0.96725700	-0.92558400	-2.27271600
F	-0.64290900	1.21575800	-2.17533600
C	0.47950800	0.10764400	3.46930200
H	1.22821700	-0.68920000	3.46760600
H	0.95751800	1.08279600	3.34185000
F	-0.19229000	0.08961800	4.66245000

*

prod4-4beckm

C	0.28105600	-0.14492000	-0.53755400
N	0.26367900	0.21509600	0.55127300
C	0.25574000	0.64036300	1.92243100
H	-0.39660600	1.51448700	1.98893500
H	1.27820800	0.92926400	2.17796600
C	0.30758600	-0.57972700	-1.92060300
H	1.35298200	-0.78092000	-2.18290900
H	-0.24810900	-1.52233100	-1.98673900
C	-0.29360200	0.46325700	-2.89451700
H	0.26929500	1.39968500	-2.87023200
H	-1.34941300	0.64520100	-2.67910100
F	-0.18362100	-0.09017500	-4.14132400
C	-0.24235100	-0.48923700	2.83969800
H	0.41235900	-1.36334600	2.78524800
H	-1.27322400	-0.76750700	2.60416100
F	-0.19713200	0.02590200	4.10891600

*

prod4-3beckm

C	-0.22427700	-0.39491300	-0.68128100
N	-0.27063200	0.05616800	0.37255900
C	-0.33230500	0.60795700	1.69519300
H	0.22977700	1.54492400	1.68524200
H	-1.38244100	0.81817000	1.91188600
C	-0.14554200	-0.91939700	-2.04028300
H	0.36753000	-1.88758000	-1.98137600
H	-1.17419600	-1.10426100	-2.37079600
C	0.57678000	0.05951500	-2.95097200
C	-0.04155800	0.69955900	-3.93795200
H	1.64105600	0.18280900	-2.77291500
H	0.51130400	1.36356800	-4.59327800
H	-1.10223200	0.57531600	-4.13910300
C	0.25336600	-0.37629100	2.71936600
H	-0.31194500	-1.31209800	2.73942600
H	1.31036200	-0.57381400	2.52093000
F	0.13589200	0.24345700	3.93718000

*

prod4-2beckm

C	0.24084300	0.20017000	-1.15317500
N	0.26692000	-0.16538500	-0.06586700
C	0.31343200	-0.60950100	1.29766100
H	-0.25521200	-1.54047800	1.35848700
H	1.36046000	-0.80848800	1.53871300
C	0.20915100	0.63030900	-2.54229300
H	-0.34704600	1.57420000	-2.56886200
H	1.24611600	0.84976900	-2.82177600
C	-0.41900500	-0.42943300	-3.46924800
H	-1.46027800	-0.62119600	-3.20624700
H	-0.38807200	-0.03844500	-4.48700900
H	0.14180100	-1.36486300	-3.44269300
C	-0.27213400	0.45518100	2.23844200
H	0.29835300	1.38676400	2.18880100
H	-1.32707200	0.64234000	2.02021200
F	-0.16365200	-0.06954600	3.50077000

*

prod4-1beckm

C	-0.06277200	-0.00008100	-1.77263900
N	-0.35478500	0.00012400	-0.66426500
C	-0.70785600	0.00058300	0.72734100
H	-1.31112200	-0.89176100	0.91069800
H	-1.31191600	0.89253200	0.90996100
C	0.29609100	-0.00086500	-3.17491200
H	1.05727400	0.76442000	-3.35110600
H	-0.59507100	0.21933100	-3.76977200
H	0.68893100	-0.98524700	-3.44429000
C	0.55449800	0.00126700	1.60570300
H	1.15498900	-0.89673200	1.43703600
H	1.15373000	0.90020400	1.43761500
F	0.10109400	0.00058000	2.89911700

*

prod4-10beckm

C	-0.36228400	-0.16865000	1.24756000
N	-0.48997300	-0.18481700	2.39589200
C	-0.62274400	-0.20677500	3.81888200

H	-0.81666300	-1.24158500	4.11247100
H	-1.48926100	0.40308700	4.08649600
C	0.65002200	0.31846300	4.49484500
H	1.51808100	-0.29356100	4.23451500
H	0.83433400	1.36504300	4.23733300
C	-0.21056500	-0.16306700	-0.14376500
C	-0.31146400	1.04975300	-0.85508500
C	0.04263100	-1.37905200	-0.82587600
C	-0.16228300	1.05615000	-2.22632200
H	-0.50554500	1.97928300	-0.33066700
C	0.18925600	-1.36702800	-2.18909700
H	0.11930200	-2.31187200	-0.27800400
C	0.08912800	-0.15328100	-2.90581800
H	-0.24141100	1.99259300	-2.76317700
H	0.38271900	-2.27910100	-2.74119100
F	0.42892100	0.22751300	5.84836500
O	0.24623100	-0.25450400	-4.21522400
C	0.16488700	0.90879000	-5.04319400
H	0.94672100	1.62539400	-4.77617300
H	0.32472600	0.55040500	-6.05741600
H	-0.82473000	1.36775500	-4.96448000

*

prod4-0beckm

C	0.00009800	-2.44849400	0.24953400
N	0.00021800	-1.38258300	-0.15543300
C	0.00038100	-0.03381400	-0.64773100
H	-0.89312100	0.07820200	-1.26767200
H	0.89452300	0.07832600	-1.26672200
H	0.00005400	-3.45989900	0.62850300
C	-0.00030600	0.97346800	0.51996800
H	-0.89996700	0.86993300	1.13238700
H	0.89873200	0.87009000	1.13333000
F	-0.00009900	2.20477200	-0.07638700

*

prod3-0beckm

C	0.42286700	-2.20027300	0.32990600
N	0.05078800	-1.23811200	-0.15552900
C	-0.41413400	0.01910400	-0.72530500
H	-1.40864500	-0.19933200	-1.12619700
H	0.26840600	0.23579300	-1.55129900
H	0.77683300	-3.11135800	0.78642000
C	-0.42129000	1.09698200	0.32520900
H	-1.16720900	1.00284300	1.10932100
C	0.40236800	2.13969300	0.26575500
H	1.13999300	2.25477800	-0.52427400
H	0.35060300	2.92766300	1.00909100

*

prod2-9beckm

C	-0.25697700	0.10266500	0.80232400
N	-0.37844900	0.16291400	1.94338300
C	-0.49391800	0.23264800	3.38019100
H	-1.39084900	-0.33540300	3.63956500
H	-0.67005800	1.28614100	3.61149200
C	0.75682200	-0.30958200	4.06335400
H	0.61536800	-0.21628400	5.14189700
H	0.91339400	-1.36384200	3.82804800
H	1.64274400	0.26305600	3.78253300
C	-0.11131100	0.03613000	-0.61308300
C	0.08617100	1.21513600	-1.34208200
C	-0.16676300	-1.20434100	-1.25937500
C	0.22190600	1.08516300	-2.72143700
H	0.13315300	2.18640800	-0.86369600
C	-0.01625900	-1.19845200	-2.64361300
H	-0.31884600	-2.13058400	-0.71766200
H	0.37716400	1.96384900	-3.33953600
H	-0.04941000	-2.13005500	-3.19975200
N	0.17282400	-0.08693800	-3.35648900

*

prod2-8beckm

C	-0.28060900	-0.05580100	0.80906500
N	-0.41197100	-0.07166500	1.95283700
C	-0.52782700	-0.08991300	3.38918600
H	-0.96594700	-1.05883000	3.64120200
H	-1.24753400	0.69075200	3.64750400
C	0.82463200	0.12407600	4.05962900
H	0.67606500	0.08913000	5.14077100
H	1.53182200	-0.66041400	3.78397000
H	1.24494300	1.09809800	3.80225000
C	-0.12460500	-0.03376300	-0.59731500
C	0.04244700	1.20342200	-1.24559200
C	-0.13898600	-1.24760000	-1.30820800
C	0.19686700	1.21364300	-2.62293900
H	0.04985300	2.12828700	-0.67920900
C	0.01733100	-1.20998000	-2.68495800
H	-0.26998200	-2.19112300	-0.78986300
C	0.18469700	0.01355600	-3.33635900
H	0.32753600	2.15640700	-3.14136700
H	0.00946900	-2.13409900	-3.25134200
H	0.30803900	0.03217000	-4.41401800

*

prod2-6beckm

C	0.16426200	0.42651200	0.06484700
N	-0.16822900	0.36704800	1.15800000
C	-0.58574500	0.25268300	2.53646200
H	-1.57890200	-0.20230200	2.50038300
H	-0.68053400	1.27535300	2.90930300
C	0.55927000	0.48302300	-1.35728400
H	0.53918100	1.54372700	-1.65788700
H	1.60122200	0.12744600	-1.41906100
O	-0.34751300	-0.31336400	-2.03823600
C	-0.07784600	-0.38118700	-3.44273500
H	0.91277100	-0.81268700	-3.62435900
H	-0.84375700	-1.03291100	-3.85780700
H	-0.15004800	0.61218200	-3.89906700
C	0.40536000	-0.57691200	3.34501800
H	1.38630700	-0.09989600	3.38248700
H	0.02182100	-0.65337600	4.36426300
H	0.50221500	-1.58533000	2.93864800

*

prod2-5beckm

C	0.43352100	-0.14349100	0.57710300
N	-0.05785700	-0.11733200	1.61001900
C	-0.65802600	-0.08149100	2.92555700
H	-1.40061500	0.71935800	2.88897500
H	-1.17282400	-1.03830700	3.04150200
C	1.03631800	-0.17845400	-0.74731100
H	1.79741000	0.60581500	-0.81135500
H	1.51089900	-1.15424000	-0.89333400

C	-0.03142100	0.04543500	-1.83776700
F	0.55095900	0.00143000	-3.02658100
F	-0.97002500	-0.90236800	-1.76555000
F	-0.61725000	1.23461700	-1.67168100
C	0.39905100	0.14821400	4.00089500
H	-0.10928800	0.16046900	4.96687300
H	1.13681800	-0.65592800	4.01235000
H	0.90100000	1.10794200	3.86629300

*

prod2-4beckm

C	0.22808400	-0.13060300	0.06482000
N	0.16018900	0.22542000	1.15243800
C	0.07338500	0.62709900	2.53934500
H	-0.65524000	1.44069700	2.56925500
H	1.05776000	1.02500900	2.79714000
C	0.31115300	-0.56320500	-1.31810500
H	1.36353100	-0.77589900	-1.53808700
H	-0.25238900	-1.49868100	-1.41020900
C	-0.23875900	0.48887800	-2.30944200
H	0.33926400	1.41543500	-2.26609900
H	-1.29736600	0.69026500	-2.12748300
F	-0.09983000	-0.06341600	-3.55496000
C	-0.33109800	-0.54821700	3.42300700
H	0.40919300	-1.34912300	3.38206200
H	-0.38852900	-0.18639900	4.45149700
H	-1.31120900	-0.93821500	3.14239900

*

prod2-3beckm

C	-0.26518000	-0.34263500	-0.07152900
N	-0.24786200	0.13007100	0.97305100
C	-0.22359800	0.70114500	2.29972600
H	0.33612400	1.63559600	2.21399600
H	-1.26284400	0.93586900	2.54224700
C	-0.26149600	-0.90208700	-1.41991500
H	0.14795600	-1.91708100	-1.34328600
H	-1.30744100	-0.98471500	-1.73624100
C	0.54969400	-0.02851700	-2.36109600
C	-0.00578300	0.64654400	-3.36191600
H	1.62241600	-0.01035200	-2.19129200
H	0.60678300	1.23556500	-4.03560000
H	-1.07519800	0.62779600	-3.55448600
C	0.40178200	-0.26245400	3.30195000
H	0.38305300	0.21635200	4.28282100
H	-0.16381900	-1.19396300	3.36443900
H	1.44068800	-0.48120400	3.04819700

*

prod2-2beckm

C	0.22342200	0.18031100	-0.54697500
N	0.20727300	-0.18642200	0.53956900
C	0.19327000	-0.61624500	1.91934600
H	-0.39763100	-1.53502600	1.94214800
H	1.22977000	-0.85865900	2.16597100
C	0.24467500	0.61684100	-1.93550900
H	-0.28385100	1.57574900	-1.97399600
H	1.29460700	0.80841300	-2.18425600
C	-0.38462900	-0.41962300	-2.88615300
H	-1.43802900	-0.58387200	-2.65438800
H	-0.31305700	-0.02688500	-3.90121200
H	0.14816100	-1.37090800	-2.84692500
C	-0.38049400	0.46528300	2.82782100
H	-0.35377300	0.08897600	3.85233900
H	0.21433200	1.37952100	2.78312000
H	-1.41779200	0.69000200	2.57285200

*

prod2-1beckm

C	-0.07888600	-0.00388400	-1.16786200
N	-0.33925400	-0.00590900	-0.05209800
C	-0.63629000	-0.00986800	1.36324000
H	-1.23498100	-0.90653400	1.54010200
H	-1.25815400	0.87069700	1.54094200
C	0.24291600	-0.00183800	-2.58020500
H	0.99489800	0.76737600	-2.77579100
H	-0.66373000	0.21213600	-3.15269700
H	0.63555600	-0.98314000	-2.85992800
C	0.64458600	0.00594100	2.19056100
H	0.36178900	-0.00124100	3.24503200
H	1.25490500	-0.87732000	1.99373400
H	1.22940100	0.90743800	1.99910700

*

prod2-10beckm

C	-0.28724000	-0.09069200	1.79747300
N	-0.38463600	-0.08116400	2.94752200
C	-0.44317500	-0.06311000	4.38537500
H	-0.64915200	-1.09275400	4.68888400
H	-1.30674600	0.55089100	4.65314800
C	0.85126000	0.46500400	4.99341800
H	0.75106400	0.44338800	6.08056700
H	1.70265300	-0.15840600	4.71326900
H	1.04038900	1.49561500	4.68701600
C	-0.17519500	-0.11946700	0.39899900
C	-0.23599500	1.08045400	-0.33510600
C	0.00023500	-1.35868200	-0.26209600
C	-0.12312400	1.05135800	-1.71068200
H	-0.37142500	2.02877000	0.17379900
C	0.11218900	-1.38261400	-1.62935800
H	0.04550900	-2.28261300	0.30407300
C	0.05308500	-0.18136100	-2.36943600
H	-0.17138100	1.97958400	-2.26526600
H	0.24734700	-2.31455800	-2.16535000
O	0.17380500	-0.31649400	-3.68163000
C	0.12214200	0.83310700	-4.52921500
H	0.24804100	0.45047300	-5.53944700
H	-0.84722400	1.33208100	-4.44052300
H	0.93602900	1.52418200	-4.29150400

*

prod2-0beckm

C	0.00027900	-1.86686700	0.24396800
N	0.00032000	-0.80088500	-0.16037300
C	0.00031000	0.56363300	-0.64351500
H	-0.88969000	0.64848300	-1.27255100
H	0.89081500	0.64876400	-1.27179700
H	0.00034500	-2.87674800	0.62404200
C	-0.00033100	1.55221200	0.51877100
H	-0.00025100	2.55627800	0.09021800
H	-0.89414500	1.44241600	1.13497400
H	0.89294000	1.44262600	1.13580000

*

prod1-9beckm

C	-0.00099800	-0.00069300	1.34701500
N	-0.00027500	-0.00068100	2.49582800
C	0.00232000	-0.00211600	3.92518400
H	0.23257500	-1.01060300	4.27139200
H	-0.98721400	0.30384400	4.26770400
H	0.75802300	0.70608300	4.26757200
C	-0.00086000	-0.00033900	-0.07637100
C	1.21718900	-0.00018400	-0.76751500
C	-1.21830600	-0.00013900	-0.76852700
C	1.14892000	0.00038800	-2.15809200
H	2.17137500	-0.00053200	-0.25365500
C	-1.14883200	0.00044000	-2.15903700
H	-2.17294700	-0.00045300	-0.25553500
H	2.05965500	0.00056600	-2.74882300
H	-2.05906000	0.00063400	-2.75054900
N	0.00032900	0.00072800	-2.83641800

*

prod1-8beckm

C	-0.00077000	-0.00090200	1.34073000
N	-0.00023500	-0.00061800	2.49189600
C	0.00281200	-0.00261000	3.91997300
H	0.23093100	-1.01002800	4.27038300
H	-0.98563900	0.30524700	4.26389200
H	0.75753700	0.70534300	4.26487900
C	-0.00078600	-0.00070400	-0.07320900
C	1.22946100	-0.00034300	-0.75650800
C	-1.23055500	-0.00067400	-0.75732900
C	1.21577900	0.00037800	-2.14225500
H	2.16629600	-0.00060700	-0.21014000
C	-1.21590000	0.00003200	-2.14306000
H	-2.16778500	-0.00120400	-0.21165700
C	0.00017800	0.00061200	-2.82925400
H	2.15204100	0.00072000	-2.68831100
H	-2.15178500	0.00009300	-2.68976200
H	0.00055300	0.00121300	-3.91416900

*

prod1-6beckm

C	0.37512000	-0.04019400	0.66223400
N	0.07355000	-0.01370900	1.76511100
C	-0.31833600	0.01959000	3.14060400
H	-1.37229600	-0.25657200	3.19640700
H	-0.16407300	1.03121700	3.51748000
H	0.29613400	-0.69572700	3.68861700
C	0.73688400	-0.07348600	-0.76989300
H	1.46662600	0.73625300	-0.93755900
H	1.24394000	-1.03609100	-0.95031100
O	-0.44853700	0.07930500	-1.46950800
C	-0.25407400	0.06763200	-2.88874600
H	0.17854600	-0.88592800	-3.21108200
H	-1.24384900	0.18823900	-3.32375000
H	0.38932500	0.89940300	-3.19611400

*

prod1-5beckm

C	0.58937000	0.07012500	1.08963200
N	0.18578500	0.04063600	2.15933700
C	-0.32927900	0.00009700	3.49470200
H	-1.22235700	0.62561200	3.53086400
H	0.43926300	0.38302300	4.16764100
H	-0.57089700	-1.03673000	3.73214800
C	1.09039200	0.10672600	-0.27582200
H	1.56705800	1.07722600	-0.44936200
H	1.83432600	-0.68731000	-0.39993900
C	-0.05826500	-0.09508000	-1.28682500
F	0.43644800	-0.05110900	-2.51384300
F	-0.64723000	-1.27661100	-1.08381700
F	-0.97453400	0.86568900	-1.13907800

*

prod1-4beckm

C	0.34452100	-0.01441300	0.66715900
N	0.05857100	-0.00557000	1.77687400
C	-0.27754200	0.00155400	3.16875200
H	-1.36085900	-0.08644000	3.26008700
H	0.07077700	0.94092200	3.59971800
H	0.21721100	-0.84732000	3.64245400
C	0.69011800	-0.02922100	-0.74157200
H	1.34584700	0.82749000	-0.93513500
H	1.25946800	-0.94605000	-0.93395100
C	-0.55135100	0.02803500	-1.66453700
H	-1.10480600	0.95986200	-1.52502800
H	-1.20436200	-0.83368300	-1.50557800
F	-0.06475500	-0.01241800	-2.94332000

*

prod1-3beckm

C	0.45589100	0.18283200	0.52618700
N	0.08058900	-0.01456900	1.59119000
C	-0.39108000	-0.26239300	2.91910200
H	-1.47460200	-0.38048000	2.87914700
H	-0.11891900	0.58847800	3.54466800
H	0.08049400	-1.17463100	3.28621100
C	0.88287300	0.43411400	-0.84629900
H	1.42668100	1.38715200	-0.84242900
H	1.59344700	-0.35687300	-1.11175500
C	-0.31336700	0.46107000	-1.78294500
C	-0.50002500	-0.45982400	-2.72268500
H	-0.99419400	1.29932700	-1.66626600
H	-1.34364800	-0.39228000	-3.40085800
H	0.18058700	-1.29533700	-2.86299000

*

prod1-2beckm

C	0.33955100	0.01385200	0.04192400
N	0.07654000	0.00638400	1.15781200
C	-0.23971500	0.00075600	2.55363000
H	-1.32305800	0.06673600	2.66035900
H	0.24356100	0.86154400	3.01720300
H	0.13340400	-0.92815700	2.98623200
C	0.64342600	0.02707700	-1.38094400
H	1.21978200	0.94008900	-1.56870500
H	1.30395100	-0.82635600	-1.57100800
C	-0.62979400	-0.03081100	-2.24813500
H	-1.27972900	0.82448000	-2.05753500
H	-0.32241900	-0.00257500	-3.29430000
H	-1.18378200	-0.95498200	-2.07730500

*

prod1-1beckm

C	0.00027100	0.00184600	-0.59597100
N	-0.00078800	0.00124900	0.54933200

C	-0.00051000	-0.00013700	1.98120800
H	0.13134500	-1.02783200	2.32153600
H	-0.95561000	0.39968900	2.32406700
H	0.82374800	0.62723900	2.32255900
C	0.00059300	0.00058400	-2.04329800
H	-0.13047100	1.02476000	-2.40329900
H	-0.82187500	-0.62584200	-2.40039100
H	0.95331000	-0.39956600	-2.40197900

*

prod1-10beckm

C	-0.04869500	-0.00348900	2.32016700
N	-0.11636300	-0.00111900	3.47223100
C	-0.19812900	0.00303900	4.89665200
H	0.06128700	-0.98764900	5.27227500
H	-1.22100900	0.25739600	5.17807500
H	0.49296000	0.75322900	5.28344800
C	0.03918100	-0.00501900	0.92120800
C	1.31025100	-0.01086000	0.29716000
C	-1.13832700	0.00089400	0.14762800
C	1.38707500	-0.01022900	-1.07233900
H	2.21608700	-0.01553800	0.89363300
C	-1.05547900	0.00164000	-1.22976000
H	-2.11031600	0.00508300	0.62905300
C	0.20892200	-0.00367900	-1.85197500
H	2.34334400	-0.01448100	-1.58182700
H	-1.96646600	0.00622200	-1.81417200
O	0.39667200	-0.00299200	-3.16237200
C	-0.72381600	0.00625600	-4.05045800
H	-1.32188900	0.91028400	-3.90461200
H	-0.29622600	0.00605400	-5.05036300
H	-1.33363100	-0.89062900	-3.90926300

*

prod1-0beckm

C	-0.00017600	-1.33741600	-0.00066000
N	-0.05211800	-0.19918300	-0.00077200
C	-0.11820300	1.23159700	0.00037000
H	-0.66394100	1.54223600	-0.89195800
H	-0.64082800	1.54169800	0.90661900
H	0.90358800	1.61431600	-0.01279700
H	0.05031800	-2.41599300	-0.00080200

*

prod0-9beckm

C	-0.00010600	0.00009200	1.92719100
N	-0.00029800	-0.00019900	3.07664400
C	0.00006300	0.00002600	0.51204100
C	1.22186100	0.00000000	-0.17735500
C	-1.22155100	-0.00000400	-0.17767500
C	1.15111300	-0.00004600	-1.56740200
H	2.17573100	0.00002100	0.33714000
C	-1.15043900	-0.00005300	-1.56770100
H	-2.17555400	0.00001400	0.33657200
H	2.06054600	-0.00006200	-2.16000700
H	-2.05971500	-0.00006900	-2.16054800
N	0.00042500	-0.00007200	-2.24269000
H	-0.00047700	-0.00051800	4.08423500

*

prod0-8beckm

C	-0.00008700	-0.00021700	1.93004000
N	-0.00021600	-0.00027100	3.08257000
C	0.00008100	-0.00011300	0.52585500
C	1.23428500	-0.00005700	-0.15678500
C	-1.23394200	-0.00006300	-0.15711000
C	1.21823000	0.00005400	-1.54104100
H	2.17075500	-0.00009800	0.39014200
C	-1.21752000	0.00004800	-1.54136200
H	-2.17055400	-0.00010800	0.38957300
C	0.00044500	0.00010700	-2.22613700
H	2.15341700	0.00010100	-2.08877500
H	-2.15256400	0.00009000	-2.08934000
H	0.00058900	0.00019800	-3.31114100
H	-0.00032900	-0.00043500	4.08816800

*

prod0-6beckm

C	0.14336500	-0.00040100	1.35118300
N	-0.26688800	-0.00036600	2.41797900
C	0.65432400	-0.00077700	-0.03485600
H	1.29291400	0.89424900	-0.13467000
H	1.29442100	-0.89479700	-0.13387900
O	-0.46523100	-0.00205200	-0.84525300
C	-0.14028200	-0.00033500	-2.24423500
H	0.42789500	-0.89893600	-2.50683800
H	-1.09626700	-0.00259700	-2.76290500
H	0.42311200	0.90160500	-2.50562600
H	-0.64047100	-0.00035700	3.35648900

*

prod0-5beckm

C	0.33289700	-0.00048800	1.64948700
N	-0.19693100	-0.00138700	2.66230300
C	0.99959900	0.00064400	0.36173900
H	1.62919400	0.89591700	0.29875500
H	1.63088800	-0.89338800	0.29806300
C	-0.03526300	0.00013600	-0.79204600
F	0.61468500	0.00143400	-1.94250400
F	-0.80593900	-1.08484600	-0.70839100
F	-0.80821900	1.08345100	-0.70724600
H	-0.67030500	-0.00207400	3.55577000

*

prod0-4beckm

C	0.16440300	-0.00044100	1.36445200
N	-0.20956800	-0.00078000	2.44747300
C	0.61674300	0.00015600	-0.00599200
H	1.24842300	0.88696200	-0.14292500
H	1.24076900	-0.89152100	-0.14626400
C	-0.54879100	0.00695300	-1.03447500
H	-1.15722100	0.90890500	-0.93486000
H	-1.16512400	-0.88997100	-0.93792200
F	0.05391100	0.00636600	-2.26023100
H	-0.52154100	-0.00127500	3.40764300

*

prod0-3beckm

C	0.12758500	-0.06129400	1.22993100
N	-0.48812200	-0.07675100	2.19677600
C	0.86495000	-0.02387000	-0.02117500
H	1.74450700	0.61190700	0.14557200
H	1.21791900	-1.04527800	-0.20987700
C	-0.02703700	0.49540100	-1.14081400

C	-0.45768100	-0.28941900	-2.12281700
H	-0.26065300	1.55521900	-1.10852400
H	-1.06066900	0.12009200	-2.92599900
H	-0.21845100	-1.34809500	-2.17637400
H	-1.02927600	-0.09466400	3.04714900

*

prod0-2beckm

C	0.16506200	0.00042600	0.77038100
N	-0.24475300	-0.00259200	1.84100600
C	0.65586000	0.00430400	-0.59209100
H	1.29139700	0.89317600	-0.68853200
H	1.30017800	-0.87801500	-0.69056000
C	-0.49221100	-0.00014800	-1.62561900
H	-1.11725000	0.88837800	-1.52876400
H	-0.03630900	0.00411800	-2.61625200
H	-1.10740000	-0.89582800	-1.53179100
H	-0.59467300	-0.00507100	2.78680800

*

prod0-1beckm

C	-0.00016100	-0.00001000	0.16508200
N	0.00013400	-0.00033900	1.30990400
C	-0.00053000	0.00000400	-1.27637400
H	0.48734700	0.91322500	-1.63170900
H	-1.03567400	-0.03436200	-1.63101000
H	0.54607400	-0.87975000	-1.63080900
H	0.00048000	-0.00090700	2.31935500

*

prod0-10beckm

C	-0.17368100	0.03871800	2.84139100
N	-0.29812400	0.01311000	3.99159800
C	-0.02140000	0.02836500	1.45994300
C	1.28119800	0.01986100	0.89357100
C	-1.16757400	0.02582500	0.63254000
C	1.41751100	0.00786100	-0.46853000
H	2.15797500	0.02176400	1.53189400
C	-1.02189500	0.01358300	-0.73636400
H	-2.15943500	0.03250200	1.07151200
C	0.27256000	0.00411600	-1.30080000
H	2.39456200	0.00020900	-0.93677900
H	-1.90434300	0.01095300	-1.36299700
O	0.51864400	-0.00873400	-2.59649600
C	-0.55724900	-0.01501300	-3.54177200
H	-1.16297200	0.88920700	-3.43722500
H	-0.07877400	-0.02708800	-4.51802800
H	-1.16909400	-0.91267000	-3.41823400
H	-0.40355100	0.14248900	4.98175100

*

prod0-0beckm

C	-0.02399800	-0.61049900	0.00000000
N	-0.05833300	0.52587000	0.00000000
H	0.00945900	-1.69383900	0.00000000
H	-0.08853200	1.54062000	0.00000000

*

h2o

O	0.00000000	-0.12682900	0.00000000
H	0.76645600	0.45125600	0.00000000
H	-0.76645600	0.45125600	0.00000000

*

ts5-0bbeckm

C	0.46918200	-0.34611900	-0.06423400
N	0.64926700	0.77822900	-0.40659100
C	-0.93701200	-0.06121300	-0.98552000
H	-0.87816000	-0.97712300	-1.58346000
H	-0.97871600	0.78751300	-1.67379900
H	0.69401600	-1.27729600	0.44500100
O	2.69877700	0.07795000	0.96815400
H	3.54404800	-0.00739600	0.47701900
H	2.92543400	0.46104600	1.82130000
O	4.97345900	-0.19266300	-0.51554200
H	5.44313800	0.54841900	-0.90959600
H	5.62278500	-0.88972200	-0.38301400
C	-2.10436900	-0.00930800	0.01134900
F	-3.22252900	-0.17557600	-0.68366000
F	-2.13021800	1.15488800	0.64619000
F	-1.99302700	-0.99125300	0.91442300

C0

C	-1.37869202	-2.74017809	1.96504976
H	-0.29906905	-2.73796720	1.75102452
H	-1.65105889	-3.78356354	2.18972774
C	-2.12254871	-2.36487034	0.75457332
C	-2.92002704	-2.11794085	-0.15222233
H	-3.76480853	-2.07871596	-0.83027561
C	-2.31457315	0.54871900	2.93206833
H	-2.08967203	1.61311347	2.82065697
H	-3.36815764	0.27345835	3.03763857
C	-1.35346311	-0.38608317	2.96794470
C	-1.71606411	-1.84052252	3.16632006
H	-1.19179758	-2.25470327	4.04134279
H	-2.79146904	-1.91813531	3.38066567
Au	-1.16070563	-0.93021972	-0.76011378
P	0.32617892	0.37135112	-1.97132979
C	0.12791482	0.11079964	-3.75730548
C	0.13461496	2.15636444	-1.71216073
C	2.05970179	-0.01559979	-1.60219649
H	2.25656240	-1.07398104	-1.82523707
H	2.72027661	0.61692461	-2.21389296
H	2.26039191	0.16753275	-0.53690022
H	0.30340902	-0.94749092	-3.99821596
H	0.84730754	0.73487453	-4.30843078
H	-0.89407862	0.38053579	-4.05993248
H	-0.89013212	2.45877897	-1.97102251
H	0.84849971	2.70427528	-2.34521770
H	0.32199749	2.40058399	-0.65737825
C	0.08924036	-0.03318587	2.79873597
C	1.10288953	-0.80311751	3.39038549
C	0.47086106	1.08838930	2.04332865
C	2.44590317	-0.45465740	3.24806209
H	0.84739221	-1.68217695	3.98693626
C	1.81132407	1.44197784	1.90663443
H	-0.29558199	1.68900644	1.54663216
C	2.80674419	0.67071860	2.50873641
H	3.21537968	-1.06748833	3.72487703
H	2.08165829	2.32275741	1.31773203
H	3.85935350	0.94351423	2.39747819

*

C1

C	-2.78599579	-1.18849870	0.36624134
H	-1.89597040	-1.75516950	0.67857463
H	-3.54627828	-1.93339619	0.08350633
C	-2.47128364	-0.41460305	-0.84352989
C	-2.40505072	0.27936389	-1.85992927
H	-2.59766298	0.86337684	-2.75229313
C	-2.83868077	2.10169085	1.80851898
H	-2.21891080	2.95034782	2.11187585
H	-3.84635725	2.32692177	1.44663161
C	-2.40499901	0.83593406	1.89551623
C	-3.31712645	-0.30737540	1.51012603
H	-3.49222682	-0.96964581	2.37293475
H	-4.29705247	0.10016051	1.22347466
Au	-0.27162560	-0.00936679	-1.34663416
P	2.04378637	-0.06938090	-1.37209303
C	2.69233658	-0.57805029	-2.98919670
C	2.82105716	1.52861441	-1.00950594
C	2.71928610	-1.25108074	-0.17272834
H	2.34846184	-2.26047175	-0.40100406
H	3.81846841	-1.24398565	-0.22178427
H	2.39578991	-0.97274333	0.84021612
H	2.30860001	-1.57591848	-3.24533587
H	3.79183560	-0.60637644	-2.95728912
H	2.36855807	0.13660348	-3.75932665
H	2.49555221	2.27300890	-1.75006093
H	3.91609325	1.42835944	-1.04765166
H	2.51936541	1.86663300	-0.00842278
C	-1.02208054	0.51423793	2.35778884
C	-0.73575727	-0.67783241	3.04084736
C	0.04260265	1.39953929	2.12622089
C	0.55231209	-0.95942769	3.49118129
H	-1.53121882	-1.40038203	3.24128875
C	1.32682747	1.11896594	2.58635262
H	-0.13286154	2.32172308	1.56560976
C	1.60862534	-0.06473077	3.27987533
H	0.73882482	-1.89739886	4.02272722
H	2.13248963	1.83406422	2.39382985
C	2.99519027	-0.36475487	3.78374571
H	3.08934762	-0.10488469	4.85098626
H	3.23481399	-1.43430126	3.68862362
H	3.75589755	0.21055923	3.23645858

*

C10

C	-2.70114044	-1.03121920	1.27606277
H	-1.86820169	-1.75048605	1.27689365
H	-3.62278644	-1.61782150	1.41722944
C	-2.80362330	-0.40700359	-0.04956567
C	-3.06734337	0.17215761	-1.10504355
H	-3.54293682	0.64397790	-1.95685486
C	-1.47974673	2.18891701	2.08369594
H	-0.63384946	2.87964985	2.04082323
H	-2.47420346	2.62171177	1.94084001
C	-1.33362058	0.87712780	2.32597738
C	-2.56301634	-0.00131265	2.41112459
H	-2.57414927	-0.55961645	3.35968336
H	-3.45713076	0.63799455	2.41606153
Au	-0.86753529	-0.03343912	-1.22189358
P	1.34696949	-0.08699634	-1.89587171
C	1.51608956	-0.48973628	-3.65775529
C	2.21931081	1.48549658	-1.66578982
C	2.29662943	-1.35036107	-1.00536699
H	1.85938176	-2.33985822	-1.20179275
H	3.34385704	-1.33987217	-1.34240139
H	2.25326601	-1.15442592	0.07534840
H	1.06320888	-1.47127985	-3.85798304
H	2.58111480	-0.51545594	-3.93318023
H	1.00126292	0.27032839	-4.26259210
H	1.72890499	2.26475935	-2.26676201
H	3.26789059	1.38442137	-1.98306299
H	2.18105573	1.77995058	-0.60822613
C	0.01108089	0.24767372	2.51569212
C	0.14620258	-1.00245438	3.13588575
C	1.18308919	0.90111199	2.09290521
C	1.40791814	-1.56529531	3.33362556
H	-0.73274586	-1.54605252	3.48874345
C	2.45382581	0.35938754	2.29726333
H	1.10764988	1.86784602	1.58795865
C	2.55372576	-0.89190974	2.92371942
H	1.49270504	-2.53916108	3.82306904
H	3.53901416	-1.33885813	3.08663876
C	3.69115031	1.07957551	1.82887461
H	4.09107059	0.62104650	0.90917800
H	3.48655554	2.13800552	1.61415116
H	4.48889735	1.03080763	2.58496588

*

C11

C	-2.64697392	-1.04216447	1.18546620
H	-1.77000997	-1.70595458	1.22616363
H	-3.53485584	-1.69356046	1.16605769
C	-2.63848440	-0.29633537	-0.08136267
C	-2.82698680	0.38929185	-1.08797695
H	-3.23213297	0.97082133	-1.90786054
C	-1.77504702	2.17834479	2.46164525
H	-0.96733768	2.90930642	2.55853727
H	-2.79010777	2.57171495	2.35239438
C	-1.55042150	0.85749139	2.50452722
C	-2.70337690	-0.11617995	2.41210644
H	-2.73803322	-0.75954203	3.30518053
H	-3.64703039	0.44756917	2.39508060
Au	-0.64834409	0.01382208	-1.18267097
P	1.55050515	-0.13276056	-1.90159304
C	1.63925763	-0.50257571	-3.67701369
C	2.51965870	1.37993039	-1.66121563
C	2.47120907	-1.45785334	-1.07085611
H	1.97174019	-2.42173569	-1.24331216
H	3.49761520	-1.50032393	-1.46500052
H	2.50327563	-1.26352279	0.01060385
H	1.12424788	-1.45115115	-3.88563184
H	2.69136934	-0.58049757	-3.98955385
H	1.14941928	0.30122089	-4.24521134
H	2.00330945	2.23216115	-2.12520174
H	3.51052095	1.25825974	-2.12474902
H	2.64786002	1.56853916	-0.58531973
C	-0.16771993	0.30272583	2.62639708
C	0.06985003	-0.91454366	3.28553900
C	0.92078642	0.99563621	2.08015484
C	1.36859407	-1.40506008	3.40611513
H	-0.75677039	-1.47643607	3.72515940
C	2.22142372	0.50383964	2.21376221
H	0.77302051	1.93243569	1.53762076
C	2.45065940	-0.70683152	2.87526857
H	1.54376688	-2.35029267	3.92645068

H 3.46943871 -1.09516085 2.96817523
O 3.22145682 1.22979516 1.66404199
H 4.06857352 0.78076997 1.79610106
*

C12
C -0.87538629 -1.23108842 2.80248020
H -0.18117747 -1.70854848 2.09475466
H -1.31929725 -2.04396767 3.39835468
C -1.97204692 -0.59770128 2.05602918
C -2.96787905 -0.02613479 1.60797917
H -3.93915844 0.42721381 1.44714753
C 0.00692983 2.20285075 3.41587045
H 0.41456286 3.11560096 2.97232338
H -0.73784604 2.32744357 4.20761091
C 0.41140230 0.98174071 3.03654811
C -0.14272801 -0.24495804 3.72800187
H 0.66662207 -0.80528460 4.22282163
H -0.83010089 0.07752200 4.52294505
Au -1.64837436 -0.13510454 -0.16596550
P -0.74569772 -0.07964088 -2.29820834
C -1.88302173 -0.74517474 -3.54605504
C -0.30918929 1.58727963 -2.86298761
C 0.76900255 -1.07105676 -2.41543100
H 0.54206222 -2.12121724 -2.18269880
H 1.18826097 -1.00062420 -3.43027198
H 1.50235223 -0.69603140 -1.68838265
H -2.13553931 -1.78599522 -3.29799826
H -1.40848514 -0.71072595 -4.53824851
H -2.80642897 -0.14868453 -3.56218495
H -1.20893763 2.21881166 -2.87761274
H 0.12107250 1.53781493 -3.87448885
H 0.42520691 2.02790336 -2.17462187
C 1.40185405 0.79240043 1.93529944
C 2.25786150 -0.31967585 1.90067806
C 1.51624524 1.72620473 0.89331339
C 3.19512498 -0.48335633 0.88345235
H 2.20798974 -1.07375398 2.69022553
C 2.46160616 1.56783001 -0.11583657
H 0.84811967 2.59117399 0.86286390
C 3.31918234 0.45981846 -0.14569636
H 3.83857079 -1.36793620 0.90202928
H 2.53079743 2.31808722 -0.90906104
C 4.28732178 0.30962433 -1.27121139
C 5.62363168 -0.36624310 -1.09071515
C 4.55862988 -1.04091879 -1.89361099
H 4.27421372 1.15465386 -1.96591802
H 5.85645794 -0.76858811 -0.10013885
H 6.47950750 0.06602673 -1.61606360
H 4.66951971 -1.08443318 -2.98055950
H 4.05328385 -1.90581198 -1.45227979
*

C13
C -2.98091994 -0.97396309 -0.68107098
H -2.29952824 -1.69020106 -0.19740784
H -3.75821164 -1.57162297 -1.18267812
C -2.26009259 -0.24813736 -1.73622323
C -1.84241198 0.43007418 -2.67673185
H -1.71530330 1.00515989 -3.58655670
C -2.74404692 2.20221424 0.91825644
H -2.09069266 2.89568154 1.45464379
H -3.49318560 2.64291527 0.25409699
C -2.66646009 0.87280214 1.07592738
C -3.63357725 -0.03090126 0.34457166
H -4.19016629 -0.66002626 1.05670746
H -4.37964746 0.59121342 -0.16962474
Au 0.00981993 0.02067917 -1.53943173
P 2.21244750 -0.15594316 -0.84449705
C 3.32516838 -0.61254600 -2.20356838
C 2.87305786 1.38945122 -0.16261891
C 2.43980651 -1.42978511 0.42653986
H 2.13946576 -2.40725296 0.02280512
H 3.49593321 -1.46613626 0.73257929
H 1.81432990 -1.20153670 1.30012476
H 3.02093283 -1.58459616 -2.61725216
H 4.35871087 -0.67985964 -1.83198468
H 3.27083892 0.14545592 -2.99795876
H 2.93061428 2.14219181 -0.96220017
H 3.87716313 1.22167911 0.25472189
H 2.20585301 1.76556873 0.62430410
C -1.63550095 0.24814192 1.95992667
C -1.83326587 -1.01695357 2.53363920
C -0.43398786 0.91374975 2.25171323
C -0.87914834 -1.59347886 3.37667342
H -2.75560891 -1.56828656 2.33649022
C 0.51868971 0.34426096 3.09658943
H -0.22488227 1.89083401 1.80583009
C 0.30007460 -0.91855144 3.66223140
H -1.06836477 -2.57803718 3.81126362
H 1.06196380 -1.35140202 4.31510162
C 1.78498881 1.07296170 3.36432087
O 2.71971030 0.60770699 3.97224967
H 1.82512858 2.11162273 2.95660039
*

C14
C -1.44230489 -1.27395956 2.58948058
H -0.62866001 -1.78613640 2.05385202
H -1.99159045 -2.05428700 3.13937151
C -2.38402299 -0.69840706 1.61879318
C -3.30336448 -0.18882249 0.97542903
H -4.24922209 0.19261316 0.60860523
C -0.60342723 2.17369982 3.03894675
H -0.07661608 3.02421609 2.59767188
H -1.50810274 2.39415555 3.61300258
C -0.15746142 0.91546183 2.91883903
C -0.89456172 -0.22907963 3.57674251
H -0.23565520 -0.76032007 4.28130195
H -1.72593344 0.17732214 4.16972978
Au -1.65011979 -0.15156348 -0.49041445
P -0.44775604 0.07893324 -2.45958864
C -1.45280702 -0.38113426 -3.89955755
C 0.11561057 1.77416675 -2.77332892
C 1.03009546 -0.97122389 -2.53357626
H 0.74662612 -2.02258889 -2.38383782
H 1.51386670 -0.85744208 -3.51530922
H 1.73720812 -0.67590850 -1.74642239
H -1.76743166 -1.43082725 -3.81068693
H -0.86453668 -0.25276355 -4.82045041
H -2.34767036 0.25587357 -3.94559615
H -0.75226565 2.44738859 -2.81836222
H 0.66231048 1.81522402 -3.72726082
H 0.77791213 2.10011297 -1.95946064

C 1.06410754 0.59993238 2.12084221
C 1.88058881 -0.49321723 2.45322839
C 1.41764948 1.38477812 1.00907972
C 3.01860045 -0.79369643 1.71298696
H 1.63534035 -1.12249398 3.31103746
C 2.55149939 1.09940256 0.25999031
H 0.78609288 2.22610162 0.71504038
C 3.33424290 0.00734064 0.62203465
H 3.65357093 -1.64099668 1.97305299
H 2.82660613 1.70539862 -0.60383965
N 4.51482925 -0.32020599 -0.18815917
O 5.24677503 -1.20128206 0.20500274
O 4.69119548 0.30673771 -1.21119458
*

C15
C -2.92469611 -0.97747214 -0.11599803
H -2.12148626 -1.56365390 0.35517618
H -3.67943030 -1.70250110 -0.45843730
C -2.39943012 -0.30383801 -1.31287789
C -2.16305478 0.32714377 -2.34457059
H -2.20013075 0.85943526 -3.28785343
C -2.96066523 2.40064057 1.15012815
H -2.31901764 3.21624086 1.49587936
H -3.89912697 2.67779229 0.66104198
C -2.63580053 1.11436571 1.33422332
C -3.55900346 0.00517265 0.88401922
H -3.90009489 -0.58691200 1.74849072
H -4.45796751 0.45112519 0.43547029
Au -0.12621663 -0.05006245 -1.56613069
P 2.18487340 -0.18466714 -1.43186015
C 2.88275718 -0.88740451 -2.95341716
C 2.98743051 1.42655623 -1.21201051
C 2.79532468 -1.23788200 -0.08596950
H 2.32393282 -2.22909997 -0.14134102
H 3.88679720 -1.34554732 -0.17440506
H 2.56071509 -0.78235829 0.88478693
H 2.48665299 -1.90147447 -3.10708251
H 3.97935067 -0.93153726 -2.87424206
H 2.60438140 -0.26217702 -3.81378503
H 2.70822947 2.09563442 -2.03834044
H 4.08029572 1.29947201 -1.19658630
H 2.65942866 1.87045107 -0.26231914
C -1.33989578 0.72518495 1.96446880
C -1.25407000 -0.41442917 2.77679736
C -0.18262853 1.48255487 1.73054328
C -0.04106698 -0.77397823 3.34183491
H -2.12303935 -1.03764379 2.99608505
C 1.01516468 1.10585813 2.31412282
H -0.19361936 2.36232413 1.08399659
C 1.10837253 -0.02401316 3.11912920
F 2.12955501 1.80300760 2.08431368
F 2.27630571 -0.39488477 3.63219222
F 0.05041517 -1.85595444 4.11139254
*

C16
C -2.90453414 -0.97020550 -0.37232344
H -2.16059696 -1.67047979 0.03409531
H -3.58239137 -1.57119415 -0.99827470
C -2.22443281 -0.01343248 -1.25749463
C -1.82829395 0.86894640 -2.02036340
H -1.71674427 1.66285369 -2.74949697
C -3.51802694 2.01973800 1.64105980
H -3.02464507 2.81969249 2.20079927
H -4.50449316 2.24057989 1.22243922
C -2.95814893 0.81152678 1.48789682
C -3.70054345 -0.28579319 0.75489968
H -4.00563212 -1.07770310 1.45838168
H -4.62671570 0.13646556 0.33961968
Au 0.07961027 0.05989316 -1.22211967
P 2.33011543 -0.41261818 -0.93648673
C 3.08705763 -0.90319726 -2.51296615
C 3.30028779 0.99943168 -0.34195649
C 2.67708977 -1.77393036 0.21261951
H 2.04294926 -2.64015781 -0.02204693
H 3.73572320 -2.06013183 0.12005356
H 2.48792430 -1.44835578 1.24483073
H 2.60709849 -1.82003121 -2.88471227
H 4.16216156 -1.08773517 -2.36768499
H 2.95151639 -0.10501933 -3.25676938
H 3.21601475 1.83049594 -1.05703572
H 4.35697610 0.70899897 -0.24209491
H 2.91546230 1.32995456 0.63355142
C -1.59271417 0.51050489 2.00736524
C -1.25034443 -0.76541129 2.47140429
C -0.60283768 1.50985159 2.01652596
C 0.03589883 -1.03937981 2.93874322
H -1.99500871 -1.56480387 2.48487693
C 0.67449677 1.24205405 2.48782934
H -0.82305856 2.50892108 1.62890807
C 1.00483320 -0.04282845 2.95288018
H 0.30201634 -2.03501047 3.30158135
O 1.68916721 2.14466996 2.51465265
H 1.39107999 3.00960088 2.19924842
O 2.25981550 -0.31483971 3.37154064
H 2.78269494 0.50067044 3.34261447
*

C17
C -3.08286142 -0.52705716 -0.33066827
H -2.40069100 -1.22295174 0.17809050
H -3.86329333 -1.14885085 -0.79601761
C -2.34974206 0.14128107 -1.41522612
C -1.88833983 0.79328137 -2.35301155
H -1.72049487 1.40907083 -3.22860681
C -3.35655413 2.87191868 1.02607140
H -2.79130159 3.73906456 1.37893792
H -4.35780904 3.06029001 0.62723402
C -2.86549186 1.62594127 1.09508764
C -3.72713715 0.45582520 0.66640625
H -4.02973782 -0.13573892 1.54635568
H -4.65351820 0.85431950 0.22930556
Au -0.06517328 -0.10277450 -1.45330478
P 2.10556788 -0.81703512 -1.07763837
C 2.73149781 -1.79468732 -2.47405913
C 3.27192237 0.55630101 -0.87366820
C 2.30597788 -1.87578441 0.38233608
H 1.58460025 -2.70426015 0.34483148
H 3.32782145 -2.28446164 0.39125396
H 2.14674347 -1.29766501 1.30259765
H 2.11390827 -2.69542662 -2.60025103
H 3.77380787 -2.09088241 -2.28227410
H 2.68632210 -1.19934669 -3.39716747

H	3.29636570	1.15720138	-1.79405666
H	4.27937691	0.16879518	-0.66056025
H	2.93917637	1.19540849	-0.04430338
C	-1.48316760	1.35812648	1.58853763
C	-1.16967090	0.13938855	2.22411443
C	-0.46224425	2.29382381	1.41910136
C	0.11556377	-0.12765258	2.68224558
H	-1.95122090	-0.60535531	2.37926474
C	0.83056325	2.04786276	1.89468598
H	-0.65978153	3.23121524	0.89423662
C	1.13379801	0.84753839	2.52906884
H	1.60492504	2.80272975	1.74885246
O	2.34880964	0.50193411	2.99944815
C	3.37644395	1.46805098	3.00498774
H	3.65869281	1.77846290	1.98519009
H	3.08861397	2.35903439	3.58780003
H	4.24467799	0.99587591	3.48133969
O	0.49591919	-1.27672848	3.27486416
C	-0.42993926	-2.33512260	3.35729020
H	-0.78728182	-2.64250212	2.35927482
H	0.09793131	-3.17793821	3.82074126
H	-1.29733963	-2.07279917	3.98636542

*

C18

C	-1.67218965	-1.16175741	2.52683623
H	-0.85597867	-1.72270083	2.04874641
H	-2.36995052	-1.91126860	2.93189694
C	-2.40019068	-0.40297779	1.49964174
C	-3.12722574	0.29788307	0.79343799
H	-3.92757519	0.88685632	0.36114527
C	-0.69685258	2.16321715	3.57797130
H	-0.11317292	3.04625538	3.30179842
H	-1.58733343	2.32832889	4.19183855
C	-0.33401585	0.92615217	3.20587424
C	-1.14460114	-0.26789221	3.66355436
H	-0.53904544	-0.91174497	4.32213180
H	-1.99244696	0.08740464	4.26657042
Au	-1.40532883	-0.07883938	-0.54899640
P	-0.06038759	-0.17516119	-2.43433800
C	-0.96987875	-0.83295932	-3.86202031
C	0.56162418	1.44756805	-2.95422888
C	1.39199452	-1.24693768	-2.25398599
H	1.07414784	-2.25548873	-1.95386795
H	1.92812483	-1.30202645	-3.21337879
H	2.06497991	-0.84118600	-1.48671212
H	-1.30624978	-1.85673583	-3.64391235
H	-0.31696897	-0.84462126	-4.74778341
H	-1.84903313	-0.20563606	-4.06704520
H	-0.28684994	2.09850896	-3.21016844
H	1.21428198	1.33615638	-3.83291541
H	1.12477218	1.90879210	-2.13115009
C	0.83847255	0.67686785	2.32225536
C	1.58004652	-0.51231655	2.39074067
C	1.24879299	1.61763153	1.36378134
C	2.66628881	-0.75740845	1.55921073
H	1.31654602	-1.27890003	3.12490612
C	2.33543446	1.39756895	0.52764215
H	0.68978884	2.55112588	1.25208413
C	3.07465108	0.19211725	0.59371449
H	3.19995759	-1.70220808	1.66491068
H	2.60000114	2.16826541	-0.19705865
N	4.11525778	-0.05854379	-0.26577694
C	4.51384566	0.93924253	-1.23351740
C	4.82141111	-1.31913964	-0.19927293
H	5.36984449	0.56364731	-1.80642510
H	3.70610765	1.17100709	-1.95129300
H	4.82088153	1.88483671	-0.75364872
H	5.59440805	-1.34172913	-0.97635504
H	5.31920040	-1.46759033	0.77525943
H	4.14878826	-2.17848577	-0.36997491

*

C19

C	-1.14665387	-0.92929444	3.01598452
H	-0.43360516	-1.49936439	2.40058795
H	-1.45721636	-1.59941827	3.83310794
C	-2.35529906	-0.64240532	2.23047771
C	-3.46149135	-0.36343591	1.76453899
H	-4.51326291	-0.16694293	1.59056898
C	-0.56056498	2.52180160	2.42500124
H	-0.19744871	3.26050172	1.70552955
H	-1.40338714	2.81875743	3.05541258
C	0.00417385	1.31503055	2.56950179
C	-0.50573823	0.33922869	3.60479487
H	0.30870304	0.01804750	4.27202320
H	-1.24157532	0.85137397	4.23996329
Au	-2.18574159	-0.31915497	-0.03883787
P	-1.41026201	-0.21825095	-2.22038908
C	-2.60404750	-0.90940178	-3.40026833
C	-1.08480694	1.47073937	-2.79517890
C	0.11550802	-1.17144662	-2.45675797
H	-0.09467114	-2.23546265	-2.27574488
H	0.48548112	-1.04026783	-3.48447546
H	0.88660717	-0.84379944	-1.74716473
H	-2.81044987	-1.95958963	-3.14952689
H	-2.19185806	-0.85065480	-4.41885429
H	-3.54341342	-0.34013248	-3.35456705
H	-2.02930167	2.03375462	-2.80914250
H	-0.65723951	1.44737389	-3.80853460
H	-0.38595449	1.97631255	-2.11591120
C	1.14462274	0.89376548	1.70368384
C	2.08054659	-0.04580974	2.14831847
C	1.30529704	1.42300872	0.41663207
C	3.15694920	-0.44146189	1.34682256
H	1.99646815	-0.49091027	3.14043979
C	2.36752866	1.03583686	-0.40362949
H	0.58753761	2.14533956	0.02461637
C	3.29201295	0.09718776	0.06556856
N	4.38870107	-0.34315654	-0.80366162
O	5.45895675	-0.55221620	-0.28464144
O	4.14156304	-0.46896251	-1.98069445
C	4.06624669	-1.42651276	1.87745000
N	4.74472768	-2.22077790	2.37085789
C	2.45848557	1.62812233	-1.71526587
N	2.45094245	2.15414264	-2.74417110

*

C2

C	-0.57972324	-1.22784143	2.80317468
H	0.02916228	-1.74833522	2.04886635
H	-1.01986404	-2.01042908	3.44095251
C	-1.69565799	-0.53833297	2.14003753
C	-2.69595883	0.08509869	1.78026757

H	-3.65452673	0.58401110	1.69784582
C	0.57401597	2.15046094	3.27774144
H	1.00933552	3.01922733	2.77584953
H	-0.09288178	2.34751627	4.12225457
C	0.85865985	0.89509758	2.90409922
C	0.27653529	-0.27906068	3.65968277
H	1.07996394	-0.88467781	4.10867420
H	-0.33085118	0.10086923	4.49345645
Au	-1.54517843	-0.06525481	-0.10294194
P	-0.92878513	0.02078844	-2.33508136
C	-2.28885105	-0.50005767	-3.41868580
C	-0.45987793	1.68144228	-2.89306585
C	0.46627786	-1.06524702	-2.74043496
H	0.21593588	-2.10299298	-2.47830012
H	0.68305700	-1.00115215	-3.81723079
H	1.35607356	-0.75901990	-2.17348614
H	-2.57674391	-1.53350202	-3.17830197
H	-1.96955160	-0.44503957	-4.47025043
H	-3.15713835	0.15676283	-3.26608788
H	-1.29880933	2.37445788	-2.73671759
H	-0.19920255	1.65659121	-3.96171437
H	0.40525761	2.03438993	-2.31484326
C	1.73837860	0.61292577	1.73099344
C	2.54711344	-0.53258969	1.68632189
C	1.77441691	1.48601063	0.63134197
C	3.36985996	-0.79927207	0.59441079
H	2.55336280	-1.23555287	2.52255018
C	2.59825647	1.23973809	-0.46275666
H	1.13710566	2.37393239	0.62084419
C	3.38868106	0.09220917	-0.47432127
H	3.99232912	-1.69614902	0.57623921
H	2.61821324	1.92799119	-1.31038330
Cl	4.38565953	-0.24768350	-1.86254383

*

C20

C	-1.10157646	-0.29048505	3.49294023
H	-0.55817858	-1.06997759	2.93730175
H	-1.25968396	-0.68881551	4.50775661
C	-2.43409419	-0.11974822	2.90013384
C	-3.60237984	0.09166022	2.57099931
H	-4.66959366	0.27723428	2.53345098
C	-0.73182591	2.83911990	1.96565583
H	-0.60844619	3.38981894	1.03047688
H	-1.43809547	3.26034200	2.68628438
C	-0.06209257	1.71368729	2.25523145
C	-0.30237104	1.02395084	3.58028366
H	0.64952413	0.79965490	4.08532565
H	-0.84571284	1.71612168	4.23791806
Au	-2.62207142	-0.22863180	0.61118244
P	-2.14918503	-0.56496944	-1.63086619
C	-3.48410069	-1.44668240	-2.48782262
C	-1.90888722	0.97744552	-2.55366145
C	-0.65969241	-1.57225382	-1.87500988
H	-0.81353528	-2.56108469	-1.41944236
H	-0.45587164	-1.69183424	-2.94907850
H	0.20053607	-1.09307772	-1.39110826
H	-3.64796389	-2.42356184	-2.01096279
H	-3.21120049	-1.59698232	-3.54321854
H	-4.41308254	-0.86151642	-2.43202691
H	-2.83710569	1.56576623	-2.51794806
H	-1.65896471	0.75443231	-3.60126944
H	-1.10075144	1.56614030	-2.10010852
C	0.90129504	1.09911231	1.29208421
C	1.70009068	0.01338415	1.66572601
C	1.03484829	1.57901956	-0.01855556
C	2.58898012	-0.58657109	0.77659563
H	1.64235723	-0.39488066	2.67400844
C	1.91509748	1.00259618	-0.92474209
H	0.44144683	2.42851650	-0.35559394
C	2.68945987	-0.08709598	-0.51897020
N	3.59229222	-0.73865369	-1.48528338
O	4.77239411	-0.72540846	-1.23521128
O	3.07912745	-1.24229743	-2.45622061
C	3.40887713	-1.77139749	1.25364387
F	2.86053069	-2.33013178	2.33402526
F	4.65216255	-1.41892353	1.57840340
F	3.48717460	-2.72095514	0.31421946
C	1.99432057	1.56696957	-2.33089368
F	1.51619731	2.81072538	-2.37878695
F	1.28657193	0.84211176	-3.20258975
F	3.25568868	1.60538189	-2.77136847

*

C21

C	-1.21809123	-1.14347353	2.79665426
H	-0.52944349	-1.66681453	2.11607139
H	-1.73365793	-1.92501328	3.37627690
C	-2.24355859	-0.44030847	2.01109830
C	-3.18901567	0.19554993	1.54167562
H	-4.11541601	0.72798948	1.35954052
C	-0.11933445	2.23067754	3.37643472
H	0.36585474	3.08996396	2.90511802
H	-0.86666164	2.44050860	4.14707888
C	0.19913308	0.96958643	3.05708896
C	-0.45771823	-0.20288262	3.74758132
H	0.29140912	-0.81057660	4.27959415
H	-1.15083786	0.17973234	4.50996295
Au	-1.88561395	-0.06182380	-0.22758864
P	-1.06747339	-0.09338557	-2.39625473
C	-2.29022239	-0.76459331	-3.55771117
C	-0.62718728	1.54836527	-3.02796806
C	0.41632180	-1.11789999	-2.59232378
H	0.23827980	-2.12393326	-2.18722695
H	0.67133046	-1.19203694	-3.66019545
H	1.25955688	-0.65567788	-2.06199954
H	-2.53949134	-1.79779106	-3.27620444
H	-1.87698557	-0.75377321	-4.57749317
H	-3.20575660	-0.15649946	-3.52905094
H	-1.50864281	2.20471727	-3.00107957
H	-0.26549682	1.46317573	-4.06376851
H	0.16485778	1.98254301	-2.40270278
C	1.20257738	0.68438314	1.99076847
C	2.06197694	-0.41790720	2.09378358
C	1.28634794	1.51561823	0.86394780
C	2.99937516	-0.66016413	1.10460041
H	2.03229918	-1.09907549	2.94583953
C	2.22049497	1.25166636	-0.12023402
H	0.62163950	2.37125890	0.73266669
C	3.09355505	0.16585952	-0.01775749
N	4.06067354	-0.11673067	-1.06970104
O	5.17941998	-0.42356258	-0.73131714
O	3.67940008	-0.02767513	-2.21687382
F	2.29406316	2.04980315	-1.17715976

F 3.79701176 -1.71107343 1.20949681
*

C22
C -2.74575064 -1.46511996 1.76543099
H -1.81181307 -1.97189571 1.48164183
H -3.51335226 -2.25100653 1.84444036
C -3.15452783 -0.56551774 0.67716955
C -3.65188056 0.23418576 -0.11744490
H -4.29717803 0.89335446 -0.68590474
C -2.22626285 1.67257505 3.50429295
H -1.61897030 2.58203090 3.53208809
H -3.25907778 1.75429396 3.85605577
C -1.73218127 0.49934021 3.08025217
C -2.60079221 -0.74039505 3.11569407
H -2.19542881 -1.47423155 3.83134239
H -3.59860919 -0.46276981 3.48460260
Au -1.61375911 -0.02687461 -0.94667005
P 0.23067275 0.05754120 -2.34735079
C -0.20282687 -0.42585304 -4.04318914
C 0.94540385 1.71994525 -2.47858825
C 1.59107634 -1.03928209 -1.85996268
H 1.22713363 -2.07355393 -1.78282115
H 2.38837829 -0.98629717 -2.61647525
H 1.98968063 -0.72571974 -0.88576031
H -0.59044441 -1.45467061 -4.04666903
H 0.68826816 -0.36956416 -4.68633383
H -0.97831978 0.24817940 -4.43433233
H 0.19341267 2.41302478 -2.88233578
H 1.82141331 1.70157095 -3.14429576
H 1.24820985 2.06868063 -1.48139272
C -0.34973994 0.37153051 2.54157377
C 0.38645261 -0.81794419 2.65975722
C 0.27707696 1.43774750 1.87522278
C 1.67195550 -0.94579790 2.14759607
H -0.04753834 -1.67986828 3.17455900
C 1.56418265 1.33613155 1.36436078
H -0.26384816 2.37857536 1.73867914
C 2.29723243 0.13161982 1.47892051
H 2.19413769 -1.89719751 2.26039307
H 2.00543562 2.20009222 0.86462619
N 3.54201275 0.00284028 0.93196018
C 4.18522304 1.03604858 0.13159657
C 4.35956526 -1.19596926 1.06241179
H 3.49063358 1.44694879 -0.61993452
H 4.52323457 1.88010126 0.76268112
C 5.36267766 0.30558365 -0.51226021
H 4.41336151 -1.53110593 2.11115259
H 3.93651437 -2.03179042 0.47130977
C 5.72148311 -0.75935119 0.52395384
H 5.03497989 -0.17327323 -1.44942359
H 6.19494350 0.98156553 -0.75337122
H 6.31696805 -0.31066902 1.33525912
H 6.29672819 -1.59857206 0.10859713
*

C23
C -1.86374127 -1.40210695 2.29564263
H -0.99703175 -1.87449173 1.80905908
H -2.44971052 -2.21750734 2.74823660
C -2.71730730 -0.78115898 1.27308313
C -3.56649544 -0.24291922 0.56026129
H -4.47034285 0.16572931 0.12374617
C -1.23527823 2.02585123 3.01126048
H -0.72760736 2.93662675 2.68246194
H -2.21169560 2.14813732 3.48908368
C -0.69110496 0.80809668 2.86741441
C -1.42532808 -0.40836251 3.38588472
H -0.79768697 -0.96589982 4.09859829
H -2.31277062 -0.07722346 3.94350481
Au -1.75319601 -0.11961054 -0.70232415
P -0.24890475 0.17872084 -2.43847519
C -0.93867775 -0.32533364 -4.04009000
C 0.28995726 1.89697022 -2.65351784
C 1.25951973 -0.80437328 -2.21813435
H 1.00365065 -1.87269701 -2.18065183
H 1.94926299 -0.61982343 -3.05517450
H 1.74313348 -0.51963746 -1.27369527
H -1.22951021 -1.38479027 -4.00110397
H -0.18566966 -0.18157819 -4.82958665
H -1.82714458 0.27988857 -4.27033338
H -0.58034406 2.53049000 -2.87679156
H 1.01064083 1.96131120 -3.48254783
H 0.76337928 2.25574477 -1.72915325
C 0.62091134 0.60593815 2.18771431
C 1.41804167 -0.51692889 2.46175883
C 1.12372397 1.52971488 1.26167050
C 2.64683752 -0.67852844 1.85041040
H 1.12077598 -1.27473160 3.18978697
C 2.35760487 1.34825473 0.64305394
H 0.53172410 2.40826298 0.99380046
C 3.16514625 0.22600800 0.90263447
H 2.69288212 2.08998730 -0.08293345
N 4.40769404 0.02498424 0.30609685
C 5.00486049 1.14091858 -0.39272085
C 4.67618324 -1.25341290 -0.33418419
H 6.05774794 0.90354433 -0.60421136
H 4.51175609 1.36661021 -1.36035824
H 4.98368025 2.04825107 0.22799634
H 4.32397380 -1.26546242 -1.38443338
H 5.75976311 -1.44788076 -0.33978946
H 4.18940849 -2.07726634 0.19723545
F 3.38431962 -1.74720257 2.20489567
*

C24
C -3.22812818 0.09084004 -0.97549211
H -2.75794320 -0.72114838 -0.39999738
H -4.24418474 -0.25227814 -1.22651453
C -2.51348324 0.26100471 -2.24791202
C -2.06524277 0.51127936 -3.36819013
H -1.91398741 0.77695800 -4.40799736
C -1.60524133 3.16918743 -0.35226397
H -0.66577129 3.67108201 -0.10734870
H -2.26003518 3.67766338 -1.06549217
C -1.97412064 2.00379802 0.19925104
C -3.31076489 1.39452901 -0.16150819
H -3.89981834 1.17880752 0.74308877
H -3.88687948 2.13193726 -0.73741921
Au -0.27336705 -0.22373671 -2.29511995
P 1.82426550 -0.99249782 -1.68903047
C 2.53553977 -2.10968125 -2.92990765
C 3.04277535 0.33138594 -1.46525903
C 1.77104709 -1.94769483 -0.14751423

H 1.12587923 -2.82604333 -0.29277397
H 2.78378474 -2.27853068 0.12579227
H 1.35443792 -1.33413267 0.66208637
H 1.87026917 -2.97271145 -3.07477162
H 3.52157555 -2.46105746 -2.59057895
H 2.64583643 -1.58048152 -3.88717712
H 3.19208791 0.84388203 -2.42662209
H 4.00001619 -0.08793535 -1.12229974
H 2.67790096 1.06216647 -0.73190661
C -1.09421932 1.28280389 1.16969213
C -1.61279423 0.30543044 2.02880899
C 0.27591182 1.57389778 1.26133558
C -0.79598039 -0.34231541 2.95397040
H -2.67116867 0.04517361 1.99472775
C 1.08074438 0.93790064 2.20034726
H 0.72455444 2.30893552 0.59187651
C 0.55498832 -0.02821798 3.05702726
H 1.19276524 -0.53306113 3.78682981
C 2.55287608 1.25040834 2.29067930
F 2.89927305 2.30236719 1.54145680
F 3.30247969 0.21349818 1.88034471
F 2.92447812 1.51611698 3.54757971
C -1.35541509 -1.40671463 3.86336793
F -1.16842045 -1.10139210 5.15363686
F -2.66617527 -1.59333562 3.68509685
F -0.75510077 -2.58761810 3.65598616
*

C3
C -2.91849269 -0.86782595 -1.69050044
H -2.37490973 -1.67038488 -1.16913307
H -3.63805558 -1.36318377 -2.36139847
C -1.98752937 -0.12313531 -2.54914657
C -1.36921733 0.57923417 -3.35114754
H -1.04446776 1.18923733 -4.18613659
C -2.68123657 2.14320827 0.12946373
H -2.07640614 2.75532553 0.80334927
H -3.22889286 2.67382163 -0.65464023
C -2.78311783 0.81102421 0.24895429
C -3.67166388 0.04358961 -0.70509538
H -4.38311117 -0.58845487 -0.15219482
H -4.27411200 0.76038249 -1.28046846
Au 0.18976057 0.07393210 -1.86326462
P 2.16787493 -0.23888822 -0.70249582
C 3.56048135 -0.61858511 -1.80281771
C 2.68331566 1.20823560 0.26031165
C 2.05693178 -1.63793745 0.44732464
H 1.83930336 -2.55369614 -0.12089682
H 3.00931580 -1.75783408 0.98435015
H 1.24704757 -1.46691287 1.16932135
H 3.34099840 -1.53050778 -2.37629173
H 4.47281977 -0.77181370 -1.20707200
H 3.71904587 0.21301718 -2.50423276
H 2.88568821 2.04208294 -0.42728559
H 3.59414791 0.97692158 0.83197619
H 1.88327897 1.50721722 0.94967368
C -2.03843921 0.05325023 1.30243542
C -2.42370128 -1.24015660 1.68151437
C -0.93737608 0.62713133 1.96090771
C -1.75145208 -1.93053448 2.69152492
H -3.27520606 -1.72400212 1.19881543
C -0.27957479 -0.05610721 2.97770178
H -0.59126224 1.62271651 1.67954011
C -0.67969425 -1.34293602 3.35145273
H -2.07762336 -2.93536205 2.96953975
H -0.15704833 -1.87682751 4.14871085
C 0.90089113 0.56442172 3.67753036
F 1.05597434 1.85939499 3.37642658
F 2.05239030 -0.05006476 3.35155698
F 0.79024014 0.47846501 5.00873380
*

C4
C -2.73980136 -1.26512224 -1.34598000
H -2.08883084 -1.75601150 -0.60650092
H -3.33614745 -2.06372729 -1.81453989
C -1.91501380 -0.67215725 -2.40865763
C -1.40378526 -0.14249759 -3.39717950
H -1.17928862 0.26981294 -4.37413952
C -3.15858371 2.17658629 -0.48105744
H -2.68328241 3.04516447 -0.01694576
H -3.83847800 2.37067563 -1.31581013
C -2.95433842 0.92767872 -0.03881236
C -3.67162068 -0.23781176 -0.68147333
H -4.27346928 -0.77988579 0.06466114
H -4.37651959 0.14690190 -1.43199385
Au 0.26484424 -0.12212192 -1.94610503
P 2.33449186 0.06607946 -0.92157084
C 3.68305196 -0.46809722 -2.01226737
C 2.74991908 1.75712988 -0.41426778
C 2.45794815 -0.96065284 0.56825628
H 2.29523631 -2.01528319 0.30419619
H 3.45262470 -0.84488929 1.02401881
H 1.69052899 -0.65347162 1.29069034
H 3.52772631 -1.51651813 -2.30443383
H 4.64575952 -0.37423179 -1.48774250
H 3.69792353 0.15549218 -2.91761905
H 2.75449019 2.41499437 -1.29500378
H 3.74326668 1.77075479 0.05871356
H 2.00094890 2.12369767 0.30143256
C -2.01335237 0.64821862 1.08714581
C -2.21554461 -0.44000861 1.94924028
C -0.90023304 1.47476768 1.31595109
C -1.35177125 -0.68566331 3.01328988
H -3.06960840 -1.10540018 1.80575767
C -0.03908492 1.24102290 2.38148107
H -0.69889053 2.31207247 0.64367306
C -0.26401960 0.15761596 3.23303287
H -1.53419663 -1.53391034 3.67646170
H 0.81598247 1.90074042 2.54658880
C 0.71306924 -0.13548798 4.33815503
F 1.74492362 -0.88222042 3.90174696
F 0.15219582 -0.81105288 5.34675859
F 1.24297781 0.98213675 4.85035311
*

C5
C -2.72434516 -1.08567425 0.40694799
H -1.85016217 -1.69608465 0.67588589
H -3.45731856 -1.77739053 -0.03650478
C -2.33338868 -0.12924868 -0.63896945
C -2.18868402 0.73319569 -1.50670329
H -2.31210235 1.49896124 -2.26354691
C -3.17050984 1.94575185 2.41294056

H	-2.64360166	2.80588272	2.83635537
H	-4.23886016	2.06760699	2.21034275
C	-2.55024281	0.78189928	2.17306467
C	-3.33108219	-0.40447633	1.64781116
H	-3.42046460	-1.17952437	2.42693762
H	-4.35471462	-0.07638356	1.41712623
Au	-0.09824343	0.07899765	-1.14845936
P	2.18501363	-0.25788473	-1.35595830
C	2.61419265	-0.76617783	-3.04533371
C	3.17780494	1.22170058	-1.01536357
C	2.84627539	-1.54966559	-0.26602807
H	2.24645129	-2.46622652	-0.35377186
H	3.88746530	-1.76551645	-0.54972576
H	2.82756578	-1.19942049	0.77558935
H	2.10924814	-1.71332429	-3.28390773
H	3.70286958	-0.90094108	-3.13050925
H	2.28491776	0.00192914	-3.75969364
H	2.86963483	2.04063140	-1.68070797
H	4.24338019	0.99986946	-1.17673070
H	3.02252609	1.53123521	0.02743325
C	-1.08377817	0.61804785	2.38796802
C	-0.53509528	-0.60232079	2.81484598
C	-0.19715843	1.67693561	2.14084608
C	0.83510511	-0.76164737	2.98990917
H	-1.19045167	-1.45137035	3.02729485
C	1.17596355	1.53459062	2.31974431
H	-0.58540063	2.63225909	1.77759755
C	1.70013320	0.30863232	2.74066967
H	1.25028662	-1.71456682	3.32656941
H	1.84791909	2.37424913	2.11825130
O	3.02612867	0.09684807	2.89137132
H	3.51454522	0.90994318	2.69902413

*

C6

C	-2.78998147	-1.07479656	-0.95689302
H	-2.12532834	-1.69949813	-0.34229580
H	-3.33266858	-1.76234969	-1.62426765
C	-1.97465218	-0.19525129	-1.80720917
C	-1.46773605	0.60961402	-2.59047056
H	-1.24816371	1.31228591	-3.38563472
C	-3.68766524	2.07450878	0.62011243
H	-3.30250230	2.90527946	1.21845488
H	-4.51991130	2.29207983	-0.05600964
C	-3.18587555	0.83523021	0.71943337
C	-3.79047530	-0.29699432	-0.08291432
H	-4.26711004	-1.02965651	0.58879601
H	-4.58893130	0.10849855	-0.72065009
Au	0.26319062	0.03664836	-1.33023532
P	2.41691846	-0.21201128	-0.51112769
C	3.56586704	-0.73251835	-1.81739454
C	3.10650352	1.32352658	0.16507256
C	2.57423713	-1.46678506	0.78999831
H	2.20877178	-2.43283009	0.41345103
H	3.63209345	-1.56344656	1.07719184
H	1.98492178	-1.18528217	1.67292375
H	3.24262885	-1.70000271	-2.22751164
H	4.58074014	-0.83129081	-1.40370427
H	3.57270720	0.01203367	-2.62631939
H	3.16158525	2.08027653	-0.63076411
H	4.11534838	1.13907449	0.56360014
H	2.45602689	1.69991847	0.96627777
C	-2.01702843	0.52954682	1.59363887
C	-1.87734212	-0.72002671	2.22461307
C	-1.00409938	1.47376491	1.80273729
C	-0.78275522	-1.00648424	3.02717338
H	-2.64566006	-1.48727624	2.09629134
C	0.09833767	1.20645175	2.61529215
H	-1.06228986	2.44581336	1.30550351
C	0.21851971	-0.04528225	3.22828441
H	-0.68054572	-1.97924588	3.51388613
H	0.86060588	1.97526284	2.75018332
O	1.26399999	-0.42186748	3.99262781
C	2.29316729	0.50789849	4.25261163
H	2.80560250	0.81992708	3.32677093
H	1.91057949	1.40084999	4.77420751
H	3.01634437	-0.00079768	4.90198672

*

C7

C	-2.75231316	-1.14221951	-0.86259818
H	-2.05060715	-1.70773389	-0.23131853
H	-3.31362768	-1.88701574	-1.44831602
C	-1.99385399	-0.32173437	-1.81881898
C	-1.53858120	0.42036723	-2.69126653
H	-1.36720033	1.04505742	-3.56012263
C	-3.48954019	2.09793915	0.52374980
H	-3.06333981	2.94680116	1.06593789
H	-4.29130332	2.31438457	-0.18857779
C	-3.07454257	0.84020486	0.73133062
C	-3.72312570	-0.30920531	-0.00746079
H	-4.20415819	-1.00233649	0.70113485
H	-4.52164429	0.08709588	-0.65079219
Au	0.23882161	0.03406606	-1.42815497
P	2.37392532	-0.10456608	-0.54007121
C	3.60594927	-0.62473881	-1.76731234
C	2.98276543	1.46440724	0.13659353
C	2.47447552	-1.31785591	0.80419349
H	2.21888993	-2.31480180	0.41752637
H	3.49366305	-1.33465980	1.21816002
H	1.76031986	-1.05175880	1.59525600
H	3.32686103	-1.60586112	-2.17717965
H	4.59634068	-0.69479611	-1.29288456
H	3.64601433	0.10576504	-2.58800339
H	3.05333295	2.20870890	-0.66963849
H	3.97603933	1.31873833	0.58697767
H	2.28439958	1.83443313	0.89957512
C	-1.95707373	0.53577895	1.67262547
C	-1.92904929	-0.65056953	2.41996204
C	-0.89369294	1.43940332	1.84148772
C	-0.88848378	-0.91567031	3.30757325
H	-2.73819596	-1.37923221	2.32425789
C	0.14233042	1.17601147	2.72939858
H	-0.87498408	2.36558321	1.26081810
C	0.17210296	-0.01448351	3.47270906
H	-0.89587381	-1.84718294	3.88103872
H	0.93972790	1.91390689	2.85146630
C	1.28717065	-0.35561095	4.38189575
C	2.51193683	0.18083042	4.35635018
H	1.07036064	-1.14341591	5.11306293
H	3.27701341	-0.14145461	5.06816040
H	2.80382841	0.94645199	3.62965094

*

C8

C	-0.58254567	-1.23065764	2.77491995
H	0.02616233	-1.75443735	2.02257870
H	-1.04505172	-2.01155601	3.39876751
C	-1.67686936	-0.51345283	2.10469383
C	-2.65899754	0.13215350	1.73390297
H	-3.60529049	0.65222098	1.64110572
C	0.61413661	2.11990684	3.28910560
H	1.06868431	2.98723712	2.80222644
H	-0.06812931	2.31874685	4.12082146
C	0.89365591	0.86354303	2.91394689
C	0.28118857	-0.30695670	3.65085769
H	1.06669688	-0.93224487	4.10427839
H	-0.32961291	0.07678328	4.48035784
Au	-1.48213019	-0.04240444	-0.13223716
P	-0.77920116	0.00190741	-2.33910709
C	-2.09463510	-0.51756937	-3.47669230
C	-0.25244998	1.64240094	-2.90405962
C	0.61546629	-1.11590213	-2.64903872
H	0.32610989	-2.14635731	-2.39831523
H	0.90678015	-1.06407043	-3.70873513
H	1.46908514	-0.82351085	-2.02153528
H	-2.41284525	-1.54043110	-3.22952655
H	-1.72396728	-0.49041244	-4.51235686
H	-2.95726286	0.15725667	-3.38050138
H	-1.08133207	2.35594244	-2.79321205
H	0.05104316	1.59400661	-3.96052740
H	0.59627667	1.98542333	-2.29626627
C	1.80034637	0.58223827	1.76151379
C	2.59281961	-0.57561133	1.72228651
C	1.88512131	1.47408425	0.67859074
C	3.44831733	-0.82889656	0.65488897
H	2.55797351	-1.29352316	2.54493063
C	2.74465288	1.23452309	-0.38638731
H	1.25923226	2.36994728	0.66429077
C	3.53698537	0.07579103	-0.41284145
H	4.05686526	-1.73608747	0.64767348
H	2.80034383	1.94438304	-1.21507802
C	4.40291340	-0.19103421	-1.53217750
C	5.12491937	-0.41704284	-2.47859903
H	5.77099962	-0.61866306	-3.31822226

*

C9

C	-0.47699906	-1.25208637	2.78879760
H	0.10407879	-1.77615902	2.01492381
H	-0.88424985	-2.03063815	3.45287222
C	-1.62604450	-0.57851180	2.16669389
C	-2.64960388	0.02617551	1.84167477
H	-3.62122224	0.50355375	1.78997424
C	0.63847260	2.13256822	3.13036115
H	1.04909048	2.99615410	2.60012932
H	-0.03390067	2.33974125	3.96796191
C	0.95965737	0.87253406	2.80438337
C	0.40430000	-0.28735283	3.60044852
H	1.22029258	-0.88224073	4.04008999
H	-0.18016830	0.10982133	4.44232758
Au	-1.53026749	-0.06170421	-0.06513264
P	-0.90186382	0.04045782	-2.29280936
C	-2.25608551	-0.44769432	-3.39796903
C	-0.38970765	1.69295496	-2.83606500
C	0.47882283	-1.07252312	-2.67407558
H	0.19246482	-2.10842465	-2.44313194
H	0.73943595	-0.99265105	-3.73997634
H	1.35126446	-0.80064083	-2.06356651
H	-2.57265864	-1.47392585	-3.16312379
H	-1.91844221	-0.39962249	-4.44412280
H	-3.11035566	0.23021330	-3.25882075
H	-1.21375399	2.40452197	-2.68401914
H	-0.11884447	1.66796750	-3.90217357
H	0.47766122	2.02232813	-2.24736413
C	1.85970295	0.57848868	1.64962651
C	2.66822679	-0.56921695	1.63384490
C	1.91909315	1.44767757	0.54597930
C	3.52005158	-0.83630988	0.56789386
H	2.64951361	-1.26715287	2.47324131
C	2.77326420	1.19955679	-0.51990199
H	1.27919167	2.33256035	0.51627166
C	3.57790291	0.05140380	-0.51302599
H	4.14310881	-1.73301498	0.57379214
H	2.81249082	1.88747446	-1.36717927
C	4.44099395	-0.22577241	-1.63148928
N	5.12742042	-0.44873816	-2.53614772

*

D0

Au	-0.36827562	-1.87031849	-0.02489586
C	-1.03247315	0.06374230	-0.20754888
C	-0.30725264	1.16169640	-0.46878164
C	0.25812723	3.47257827	-0.21345672
C	-0.85311383	2.57482413	-0.59185483
H	-1.03258309	2.81562101	-1.66453421
C	1.19002031	1.29161359	-0.66072135
H	1.44076315	1.26392985	-1.73560760
H	1.75813793	0.49309771	-0.16514645
C	1.50734616	2.68148286	-0.09860546
P	0.36340472	-4.08773601	0.18160428
C	-0.98324962	-5.31053458	0.28817273
C	1.39536054	-4.66225315	-1.20591749
H	-2.11242138	0.26066403	-0.09771032
C	1.37818703	-4.40534225	1.66158275
H	0.79758425	-4.16408695	2.56411940
H	1.68802810	-5.46068155	1.70119125
H	2.27310770	-3.76601475	1.64148951
H	-1.61943916	-5.08241077	1.15592826
H	-0.57661266	-6.32779881	0.39389832
H	-1.60138872	-5.26038806	-0.62011821
H	0.82901463	-4.58431745	-2.14554246
H	1.70331753	-5.70799050	-1.05441064
H	2.29134039	-4.02907358	-1.28431747
H	-1.80270659	2.77881308	-0.07615094
H	1.68091246	2.62592889	0.99697691
H	2.38867072	3.18636979	-0.51876610
C	0.14793792	4.87015050	0.01453335
C	-1.10785350	5.52093680	-0.10487246
C	1.29062175	5.63597046	0.36458626
C	-1.21113030	6.88303518	0.11398749
H	-1.99801501	4.94922489	-0.37294562
C	1.17596755	6.99621230	0.58947303
H	2.26561489	5.15481435	0.45991595
C	-0.07177088	7.61682745	0.46183689
H	-2.17730694	7.38225051	0.01881973
H	2.05491809	7.58372290	0.86144689
H	-0.15798219	8.69263451	0.63599482

*

D1
Au -0.37370806 -2.20944166 -0.08575691
C -1.03424345 -0.27539272 -0.28086356
C -0.30598568 0.81966016 -0.54712857
C 0.27096402 3.12983522 -0.29739909
C -0.84687455 2.23423667 -0.67664154
H -1.03728514 2.46904846 -1.74747614
C 1.19206014 0.94191460 -0.73759588
H 1.44246112 0.91349785 -1.81259623
H 1.75505870 0.13885508 -0.24330130
C 1.51754536 2.32945946 -0.17358966
P 0.36011868 -4.42460587 0.13781404
C -0.92218130 -5.58524380 0.71114535
C 0.98429901 -5.16004333 -1.40800588
H -2.11372077 -0.07519284 -0.17207316
C 1.72529665 -4.63836464 1.32588506
H 1.40058097 -4.30484393 2.32246691
H 2.03362198 -5.69354010 1.37928622
H 2.58442870 -4.02525639 1.01633716
H -1.30463301 -5.25607843 1.68846240
H -0.51238400 -6.60233807 0.80538342
H -1.75794866 -5.59591594 -0.00386511
H 0.19337145 -5.13806671 -2.17195911
H 1.30181791 -6.20100010 -1.24381674
H 1.83971306 -4.57370017 -1.77458173
H -1.79039389 2.44047749 -0.15051405
H 1.69427114 2.27056466 0.92019064
H 2.40078081 2.82693569 -0.59846081
C 0.16793034 4.52073657 -0.07759151
C -1.08309127 5.18744705 -0.20048846
C 1.31052134 5.28963027 0.27172988
C -1.17641869 6.54532482 0.01165532
H -1.97867897 4.62345081 -0.46766610
C 1.20258474 6.64825209 0.48904001
H 2.28511533 4.80828657 0.37131743
C -0.03796341 7.30049002 0.36195644
H -2.14198590 7.04685430 -0.08868105
H 2.08818048 7.22829731 0.75808880
C -0.16229698 8.77128456 0.60167995
H -0.78203353 8.95371903 1.49444094
H 0.81564270 9.24596901 0.75336528
H -0.67086647 9.25900192 -0.24378983
*

D10
Au -0.35978711 -2.15381719 -0.04409710
C -0.92681831 -0.18744349 -0.20702271
C -0.15336706 0.87661436 -0.47000046
C 0.52152956 3.15733158 -0.20694631
C -0.63361617 2.31423842 -0.58149642
H -0.81146058 2.56800902 -1.65128450
C 1.34656648 0.93757618 -0.67394445
H 1.58662602 0.90513336 -1.75113232
H 1.88064808 0.10991069 -0.18838759
C 1.73375326 2.30757389 -0.10571334
P 0.25378390 -4.40871050 0.14449202
C -1.10791841 -5.50826558 0.65033384
C 0.88155167 -5.13985922 -1.40187782
H -1.99510454 0.06010762 -0.08587418
C 1.57097246 -4.72271543 1.36372699
H 1.23764492 -4.39211630 2.35842704
H 1.81993824 -5.79408247 1.40207902
H 2.47006046 -4.15115359 1.09018640
H -1.49676085 -5.18565349 1.62729701
H -0.76194937 -6.55051294 0.72293990
H -1.92262302 -5.44666288 -0.08608639
H 0.11342208 -5.05911073 -2.18496472
H 1.14134120 -6.19923288 -1.25501954
H 1.77477450 -4.58947684 -1.73225162
H -1.56765617 2.55926097 -0.05549467
H 1.91319423 2.23584074 0.98769801
H 2.63429451 2.77294041 -0.53059010
C 0.47753490 4.55601379 0.02891220
C -0.74810643 5.26349605 -0.08925930
C 1.65338835 5.26262354 0.38471129
C -0.81664664 6.63158278 0.13129944
H -1.65772332 4.72476811 -0.36306527
C 1.58988859 6.62539895 0.61631195
H 2.60608491 4.73900053 0.47958619
C 0.37129934 7.29603473 0.48781631
H 2.48875232 7.17894471 0.89474686
H 0.33751944 8.37464690 0.66944367
C -2.10693280 7.39243766 -0.00006526
H -2.93780331 6.72706841 -0.27066081
H -2.36219522 7.89621250 0.94462371
H -2.02257821 8.17112176 -0.77344321
*

D11
Au -0.37083604 -2.13748522 0.01866041
C -0.95930798 -0.17513924 -0.11795957
C -0.21235913 0.88550918 -0.45873721
C 0.47329805 3.16846799 -0.27602852
C -0.70258949 2.32098675 -0.55115250
H -0.94999581 2.56349665 -1.61070775
C 1.26604917 0.94696437 -0.78395427
H 1.41861087 0.90005619 -1.87638474
H 1.84040014 0.12768009 -0.33144460
C 1.69254384 2.32561165 -0.26729536
P 0.26955258 -4.38806103 0.16252501
C -1.07001180 -5.56518648 -0.21062366
C 1.61546014 -4.84997779 -0.97538784
H -2.01482914 0.07226309 0.08610009
C 0.87294877 -4.89575969 1.80513068
H 0.08511232 -4.73131133 2.55486768
H 1.15641714 -5.95924365 1.80402593
H 1.74747728 -4.28805847 2.08041339
H -1.89741235 -5.41565114 0.49850505
H -0.70939014 -6.60213242 -0.13350663
H -1.44647530 -5.38696806 -1.22853164
H 1.30791913 -4.64980178 -2.01224307
H 1.86519944 -5.91666127 -0.87036053
H 2.50734879 -4.24554810 -0.75377059
H -1.59994102 2.57462871 0.03146191
H 1.96169742 2.27057195 0.80899178
H 2.55288522 2.79094798 -0.76860027
C 0.44361958 4.57367331 -0.04717377
C -0.78989789 5.26614132 -0.06857210
C 1.64440794 5.28214844 0.20202903
C -0.82584628 6.63872052 0.14797963
H -1.72989097 4.74462412 -0.25547339
C 1.59513492 6.64816545 0.42588748

H	2.60240080	4.76132748	0.22151491
C	0.37448343	7.32331814	0.39733288
H	2.51221214	7.20665085	0.62282835
H	0.34526940	8.40362375	0.57033765
O	-2.01789924	7.25368196	0.11232151
H	-1.91506484	8.20138139	0.28199969

*

D12

Au	-0.38706054	-2.81992400	0.15449692
C	-1.14041157	-0.91086985	0.08608935
C	-0.56425569	0.18195344	-0.43758861
C	-0.00105587	2.50764758	-0.44310994
C	-1.15957748	1.58074682	-0.44911808
H	-1.66264146	1.76729363	-1.42348355
C	0.79921023	0.32050812	-1.08274039
H	0.70705889	0.25782358	-2.18128893
H	1.50810265	-0.45540094	-0.76385875
C	1.24643337	1.73212715	-0.68561406
P	0.46406947	-5.00239609	0.24820511
C	-0.67946366	-6.28319885	-0.36125922
C	1.98002289	-5.24137275	-0.73423632
H	-2.13986955	-0.72453977	0.51459150
C	0.91727304	-5.55529745	1.92425742
H	0.02741889	-5.53752266	2.57056391
H	1.32793668	-6.57616526	1.90029850
H	1.66857203	-4.87183956	2.34646293
H	-1.60279466	-6.26799757	0.23616903
H	-0.21931604	-7.28061443	-0.29248565
H	-0.93915634	-6.07503289	-1.40958537
H	1.77257526	-5.01529925	-1.79052299
H	2.34204306	-6.27723385	-0.64961399
H	2.76151377	-4.55413503	-0.37816629
H	-1.91083002	1.78815208	0.32656055
H	1.76049347	1.71319454	0.29667650
H	1.93744950	2.22918628	-1.38117822
C	-0.06596617	3.89760322	-0.23204689
C	-1.31070535	4.54445840	0.00823258
C	1.11242691	4.69744612	-0.25056884
C	-1.36674526	5.90462615	0.21310427
H	-2.23432477	3.96299145	0.02837307
C	1.04796090	6.05501365	-0.03748526
H	2.08328639	4.23194525	-0.43048949
C	-0.19310142	6.68969192	0.19847326
H	-2.32925725	6.38878039	0.39227552
H	1.97071078	6.63916101	-0.05276664
C	-0.30150020	8.14148939	0.43134526
C	0.79322942	8.89338831	1.16696589
C	0.61442447	9.11485849	-0.28908586
H	-1.32308262	8.48635533	0.60799160
H	1.65740161	8.31452804	1.50520437
H	0.46046737	9.66769997	1.86263507
H	0.15469636	10.04646598	-0.62833034
H	1.35235465	8.69227452	-0.97701727

*

D13

Au	-0.38182035	-2.37225919	0.00778150
C	-0.90682707	-0.39307595	-0.14729447
C	-0.12556872	0.64214897	-0.48866897
C	0.62658017	2.90142241	-0.29863659
C	-0.57161573	2.09194194	-0.58845475
H	-0.79559041	2.34015584	-1.65216977
C	1.35619133	0.65834869	-0.80363201
H	1.51557275	0.60487372	-1.89471950
H	1.90215603	-0.17677318	-0.34512265
C	1.82039050	2.02421903	-0.28578089
P	0.18398377	-4.64068885	0.17898016
C	-1.18019611	-5.77754484	-0.22793310
C	1.54661197	-5.14962452	-0.91839507
H	-1.95547263	-0.11229988	0.04888003
C	0.72582372	-5.15565250	1.84062266
H	-0.07788926	-4.96533824	2.56709107
H	0.97976320	-6.22654250	1.85266703
H	1.60884780	-4.57052146	2.13714220
H	-2.02473305	-5.59672912	0.45319757
H	-0.85552433	-6.82488575	-0.13325631
H	-1.51772153	-5.59431598	-1.25857834
H	1.27258035	-4.94956327	-1.96468416
H	1.76310689	-6.22195758	-0.79838365
H	2.44893971	-4.56848144	-0.67752870
H	-1.46884763	2.37281162	-0.01830664
H	2.08512484	1.96309951	0.79148025
H	2.69502879	2.46551375	-0.78467504
C	0.63679582	4.30485723	-0.06022867
C	-0.56854835	5.04792288	-0.09255433
C	1.85280659	4.98194137	0.21103432
C	-0.54964338	6.41536134	0.13112593
H	-1.52148836	4.55335349	-0.29731248
C	1.86322680	6.34845518	0.44411672
H	2.79175558	4.42613263	0.24005152
C	0.66700298	7.06265111	0.40150909
H	2.80299001	6.86174283	0.65490362
H	0.65573325	8.14179363	0.57787783
C	-1.81864839	7.19677488	0.08914004
O	-1.87193513	8.38599789	0.27901476
H	-2.73272474	6.59898313	-0.13421540

*

D14

Au	-0.38353585	-2.80449302	0.08905539
C	-1.06243994	-0.87461816	-0.08730445
C	-0.38656963	0.19346771	-0.53506034
C	0.22096402	2.50256518	-0.51056185
C	-0.93571579	1.60666931	-0.63737006
H	-1.25929955	1.80101504	-1.68939630
C	1.05394933	0.29374535	-0.99474244
H	1.11374056	0.20149145	-2.09319858
H	1.70238421	-0.47769079	-0.55905112
C	1.46374247	1.71065944	-0.58132407
P	0.37669871	-5.01195191	0.29583147
C	-0.88475518	-6.18798889	0.88301560
C	0.98728508	-5.73380108	-1.26151887
H	-2.10515496	-0.65327236	0.19611950
C	1.76044004	-5.21301448	1.46426850
H	1.44534972	-4.88707164	2.46640385
H	2.08133516	-6.26482437	1.50933138
H	2.60811931	-4.58900564	1.14522133
H	-1.25642625	-5.86647383	1.86699646
H	-0.46217332	-7.20067720	0.96757941
H	-1.73052740	-6.20518301	0.18003331
H	0.18366637	-5.72264484	-2.01234413
H	1.32364632	-6.76997886	-1.10493691
H	1.82746966	-5.13341939	-1.64033297

H	-1.80343163	1.85822995	-0.01098097
H	1.80939414	1.72185667	0.47697951
H	2.26949509	2.18526910	-1.15897117
C	0.15768882	3.92655796	-0.33401915
C	-1.09192935	4.57036022	-0.19048324
C	1.34430834	4.69357057	-0.30110882
C	-1.15733673	5.94382409	-0.01970587
H	-2.01862992	3.99564235	-0.20787357
C	1.28677632	6.06685986	-0.12712266
H	2.31828866	4.21557114	-0.41395766
C	0.03537795	6.65815106	0.00902421
H	-2.11152749	6.45786603	0.09256723
H	2.19061786	6.67485066	-0.09926925
N	-0.03049930	8.12858646	0.19734209
O	-1.12539574	8.62759165	0.30616751
O	1.01501213	8.73321705	0.23055043

*

D15

Au	-0.37753195	-2.71060315	0.00909490
C	-1.05227026	-0.77929510	-0.16762721
C	-0.34630987	0.30439436	-0.52217790
C	0.24684878	2.61328231	-0.37421191
C	-0.89346093	1.71792919	-0.63557874
H	-1.14256697	1.93635965	-1.70080015
C	1.12874463	0.42050628	-0.84855393
H	1.28423545	0.36138815	-1.93986165
H	1.73696724	-0.36538579	-0.38162309
C	1.49813871	1.82387950	-0.35630879
P	0.38178388	-4.91936527	0.20592248
C	-0.87039376	-6.09572916	0.81198650
C	0.96848273	-5.64106267	-1.36087190
H	-2.11713099	-0.57079338	0.03054494
C	1.78346687	-5.12149869	1.35276954
H	1.48336263	-4.79762136	2.36021393
H	2.10611248	-6.17302799	1.39127769
H	2.62573171	-4.49624398	1.02219507
H	-1.22498857	-5.77613178	1.80276283
H	-0.44735100	-7.10899034	0.88731240
H	-1.72817518	-6.11096078	0.12382540
H	0.15396237	-5.62836898	-2.09988774
H	1.30577990	-6.67786773	-1.21005167
H	1.80389550	-5.04173809	-1.75190367
H	-1.80384701	1.94498597	-0.06229863
H	1.76764475	1.79979841	0.72181660
H	2.34006881	2.31649639	-0.86351491
C	0.16102936	4.01982006	-0.16018631
C	-1.09994320	4.66313062	-0.17473740
C	1.33571177	4.77544977	0.07034775
C	-1.16875563	6.02151414	0.03391398
H	-2.02936735	4.11932016	-0.34862337
C	1.23730971	6.13123010	0.28393095
H	2.32642234	4.31931906	0.08889040
C	-0.00868756	6.76670017	0.26707401
F	-2.32710302	6.66152567	0.02509728
F	-0.08891483	8.05795476	0.46896649
F	2.31057856	6.87278892	0.50676489

*

D16

Au	-0.36594802	-2.44904237	-0.01201451
C	-0.96890568	-0.49588397	-0.20312299
C	-0.22224883	0.56830039	-0.53671122
C	0.45571920	2.85626047	-0.33265394
C	-0.71840557	2.00032211	-0.65221529
H	-0.97113901	2.23101630	-1.70930888
C	1.26409045	0.63219360	-0.82040246
H	1.44315253	0.59672881	-1.90946849
H	1.82379735	-0.19565836	-0.36439216
C	1.68112251	2.00402824	-0.27563673
P	0.29669811	-4.68654250	0.21348513
C	-1.08025369	-5.84932306	0.48087091
C	1.17558240	-5.35539882	-1.23615503
H	-2.03137993	-0.25545849	-0.02749537
C	1.42805660	-4.99354584	1.60888723
H	0.94182356	-4.69531657	2.54936317
H	1.70019905	-6.05866372	1.66273757
H	2.34076472	-4.39266549	1.48312855
H	-1.63070728	-5.56879563	1.39096653
H	-0.70516101	-6.87854057	0.58642489
H	-1.77170582	-5.80173594	-0.37322550
H	0.52619522	-5.29494139	-2.12198002
H	1.46034540	-6.40504403	-1.06699724
H	2.08092035	-4.76058507	-1.42700918
H	-1.61658114	2.23736747	-0.06334544
H	1.93593442	1.93018548	0.79972291
H	2.54980412	2.46311192	-0.76825335
C	0.41348442	4.23648082	-0.10943217
C	-0.82663970	4.95115075	-0.17418566
C	1.60433067	4.95810373	0.19549093
C	-0.85959390	6.29980137	0.05283355
H	-1.75711184	4.42840466	-0.40654047
C	1.57146791	6.31243548	0.43143331
H	2.55958065	4.43350543	0.24795651
C	0.34714648	6.99602072	0.36288753
H	2.47351022	6.87823723	0.67017554
O	0.30574271	8.29074610	0.58554354
H	-0.61043935	8.61191039	0.50044314
O	-1.94751448	7.09297836	0.02280091
H	-2.75058077	6.59304474	-0.18635582

*

D17

Au	-0.42649072	-3.05004858	-0.00114721
C	-1.21187861	-1.16305734	-0.19473842
C	-0.58964772	-0.05474469	-0.62632103
C	-0.08965990	2.28401568	-0.52345349
C	-1.21243474	1.32789158	-0.73880407
H	-1.55557505	1.50120139	-1.78077169
C	0.85592412	0.11925082	-1.04314828
H	0.94030795	0.06404075	-2.14282255
H	1.52165766	-0.64601132	-0.62144884
C	1.20276248	1.53652642	-0.57223348
P	0.45758006	-5.20964214	0.22840494
C	-0.77075642	-6.54455284	0.05858858
C	1.74476218	-5.61406028	-0.99647245
H	-2.27194470	-1.00350852	0.06662246
C	1.24909552	-5.52150582	1.84001732
H	0.50905541	-5.39014463	2.64317116
H	1.65663610	-6.54287128	1.88498705
H	2.06323745	-4.79869269	1.99643835
H	-1.54988176	-6.42545819	0.82576002
H	-0.29483843	-7.53024762	0.17386001
H	-1.24536010	-6.48459043	-0.93186352

H	1.32646570	-5.53193354	-2.01040628
H	2.12509268	-6.63528678	-0.84248065
H	2.57643435	-4.90018488	-0.90363366
H	-2.08407251	1.50214171	-0.09216275
H	1.57469414	1.52165232	0.47084403
H	1.97185205	2.05439989	-1.16366110
C	-0.21122559	3.65616440	-0.29187027
C	-1.49262257	4.26878236	-0.23539977
C	0.95039380	4.47549320	-0.09584736
C	-1.62364475	5.61844235	0.00297091
H	-2.39335785	3.67078171	-0.38196611
C	0.83436858	5.81949954	0.14843588
H	1.93836917	4.01812101	-0.13896886
C	-0.47737726	6.41097381	0.20293775
H	-2.61446135	6.07032830	0.04189577
O	-0.49905094	7.69676483	0.44536585
C	-1.73630205	8.40014039	0.52668269
H	-2.27618831	8.34860373	-0.43022668
H	-2.35964591	7.99829796	1.33907084
H	-1.47550049	9.44122153	0.74531894
O	1.84335536	6.67384197	0.34848451
C	3.16459641	6.17510296	0.30246075
H	3.39715896	5.74542367	-0.68626732
H	3.82601554	7.02982426	0.48691894
H	3.33456519	5.41400817	1.08225631

*

D18

Au	-0.33789821	-2.90654008	0.18918072
C	-1.01996834	-0.97172342	0.13377989
C	-0.39701863	0.11661406	-0.34709808
C	0.22086157	2.43854689	-0.33839377
C	-0.96688267	1.52578363	-0.37689550
H	-1.48568311	1.70692734	-1.33948575
C	1.00056255	0.22648828	-0.92072809
H	0.95763742	0.18329539	-2.02375302
H	1.66861643	-0.57855183	-0.58466655
C	1.47074411	1.61894091	-0.48011678
P	0.39575548	-5.13322478	0.24437664
C	-0.92465241	-6.33930536	0.59417334
C	1.13089808	-5.71022611	-1.32023471
H	-2.03577363	-0.76793456	0.51437654
C	1.67264598	-5.47643529	1.49824308
H	1.28542400	-5.22769985	2.49721458
H	1.96693468	-6.53677098	1.47707316
H	2.55631529	-4.85143791	1.30241139
H	-1.36912104	-6.12553038	1.57731796
H	-0.52738126	-7.36567864	0.59140617
H	-1.71136377	-6.25248761	-0.16963388
H	0.39219046	-5.61517645	-2.12967752
H	1.44838899	-6.76100166	-1.24000154
H	2.00193861	-5.08668339	-1.57003890
H	-1.70271878	1.73423916	0.41421296
H	1.92701130	1.56798166	0.52646073
H	2.21741292	2.08220717	-1.14046826
C	0.17029506	3.80974347	-0.18730874
C	-1.08230150	4.50492129	-0.04409668
C	1.36266038	4.61614229	-0.16324059
C	-1.14626999	5.85533050	0.10454957
H	-2.01407351	3.93553088	-0.05451439
C	1.31780390	5.96688725	-0.00841450
H	2.33660404	4.13424172	-0.27135605
C	0.05614563	6.65029206	0.13225467
H	-2.11890349	6.33332139	0.21008192
H	2.24809074	6.53274214	0.00051425
N	0.00260241	7.97058521	0.28138097
C	-1.27947103	8.66099455	0.38201346
C	1.22321336	8.76881327	0.33872454
H	-1.09872563	9.73923176	0.42944225
H	-1.90737886	8.45434257	-0.49668383
H	-1.82319720	8.35859499	1.28973975
H	0.95736117	9.80963215	0.54772660
H	1.88744851	8.41662233	1.14096650
H	1.76617390	8.73271699	-0.61795785

*

D19

Au	-0.38781471	-3.46321717	-0.04157481
C	-1.06781056	-1.53742822	-0.26371199
C	-0.34575535	-0.45806722	-0.59534684
C	0.24324236	1.84413616	-0.44046395
C	-0.88231084	0.95823756	-0.72974162
H	-1.05641551	1.18116027	-1.81399818
C	1.14135126	-0.34403371	-0.86461222
H	1.34333643	-0.41147950	-1.94761875
H	1.73210491	-1.12185178	-0.36376171
C	1.48671366	1.06446974	-0.37311728
P	0.38695441	-5.66116969	0.20665326
C	-0.69672139	-6.73433411	1.20269641
C	0.59590369	-6.55213589	-1.36851987
H	-2.13938460	-1.32803037	-0.10951234
C	2.01496641	-5.78068385	1.01646046
H	1.95912084	-5.33839406	2.02196505
H	2.33187398	-6.83139704	1.09904220
H	2.76045324	-5.22273490	0.43103916
H	-0.81444975	-6.30579614	2.20876776
H	-0.27077795	-7.74567500	1.28640577
H	-1.68837148	-6.79627679	0.73104149
H	-0.37102882	-6.61239540	-1.88917560
H	0.97761119	-7.56947067	-1.19341971
H	1.30276942	-6.00475918	-2.00910066
H	-1.82710268	1.20361127	-0.22309517
H	1.68663304	1.05444226	0.72439625
H	2.35777301	1.55936788	-0.82640548
C	0.15409384	3.27474916	-0.23919974
C	-1.07617695	3.93616089	-0.38944320
C	1.29985559	4.00920549	0.10904112
C	-1.16149937	5.31713950	-0.21380424
H	-1.98136259	3.38906271	-0.65666096
C	1.22053273	5.38435641	0.32497752
H	2.26766413	3.52033685	0.22892813
C	-0.01043519	6.02511413	0.14889654
N	-0.09614426	7.48420182	0.34780630
O	-0.69676321	8.11006814	-0.48960047
O	0.44127508	7.93052245	1.33035096
C	-2.43963297	5.95940407	-0.38825148
N	-3.48866902	6.41834026	-0.53588449
C	2.41703141	6.09741462	0.69484313
N	3.40797522	6.61475076	0.98363358

*

D2

Au	-0.38606913	-2.54868760	-0.04146319
C	-1.05295239	-0.62080538	-0.27340064

C	-0.33321551	0.46193879	-0.60357300
C	0.25436552	2.77317065	-0.43544755
C	-0.87523754	1.87450781	-0.74980908
H	-1.08771269	2.08593052	-1.82294447
C	1.15561420	0.57557029	-0.86004917
H	1.36157999	0.51389676	-1.94291862
H	1.73892334	-0.21187378	-0.36446386
C	1.50404530	1.97904565	-0.35255314
P	0.36157736	-4.75357369	0.23014229
C	-0.90321483	-5.90621110	0.85566800
C	0.96340717	-5.52798604	-1.30540404
H	-2.12614948	-0.41277101	-0.12539614
C	1.74756154	-4.92630991	1.40073191
H	1.43550942	-4.57003584	2.39346970
H	2.06443800	-5.97761251	1.47606068
H	2.59722283	-4.31534951	1.06226811
H	-1.27155114	-5.55478206	1.83065751
H	-0.48482343	-6.91788942	0.96866987
H	-1.75075982	-5.94058342	0.15543453
H	0.15657046	-5.53802218	-2.05283897
H	1.29503685	-6.56025876	-1.11617341
H	1.80477060	-4.94502772	-1.70798445
H	-1.80553576	2.10039445	-0.20872742
H	1.72862713	1.95470300	0.73475066
H	2.36673025	2.46801206	-0.82724997
C	0.16053395	4.17491524	-0.23265827
C	-1.09206088	4.83496124	-0.32378845
C	1.31652946	4.94105748	0.06697975
C	-1.18747753	6.19852985	-0.12919866
H	-1.99722905	4.27062758	-0.55392773
C	1.22734408	6.30353987	0.27157925
H	2.29291008	4.45969633	0.14266451
C	-0.02559975	6.92143936	0.16915613
H	-2.14906334	6.70856069	-0.20203605
H	2.11409815	6.89403341	0.50632490
Cl	-0.14069540	8.62094347	0.41569909

*

D20

Au	-0.36575552	-4.32369681	0.03837458
C	-1.04871285	-2.39224276	-0.10701325
C	-0.35942877	-1.31155141	-0.49940667
C	0.23232967	0.99850117	-0.42747492
C	-0.91185137	0.10075304	-0.59190621
H	-1.19601659	0.31338562	-1.65389763
C	1.09606006	-1.19590860	-0.90655903
H	1.19650477	-1.28003891	-2.00257708
H	1.73530816	-1.96412900	-0.45234587
C	1.48070045	0.22105550	-0.47102810
P	0.40316434	-6.53197215	0.19782359
C	-0.37531540	-7.67959434	-0.98367233
C	2.19062387	-6.71007343	-0.10811385
H	-2.10228276	-2.18132604	0.14140552
C	0.14091920	-7.30721382	1.82543109
H	-0.93338013	-7.31623362	2.06099729
H	0.52131203	-8.33992772	1.82856510
H	0.66321178	-6.72661194	2.59992157
H	-1.46055089	-7.71025658	-0.80739403
H	0.03929593	-8.69291414	-0.87118341
H	-0.19852436	-7.32777395	-2.01064497
H	2.42969944	-6.34506424	-1.11780384
H	2.49671846	-7.76375845	-0.02264734
H	2.75248174	-6.10943490	0.62213271
H	-1.80439500	0.34221644	0.00324143
H	1.78615543	0.22986425	0.60132395
H	2.30471603	0.70638035	-1.01312209
C	0.15326163	2.42754968	-0.24709857
C	-1.09740483	3.06226869	-0.14634988
C	1.32588508	3.20078253	-0.18047973
C	-1.18336187	4.43777571	0.00686823
H	-2.01928449	2.48128374	-0.18469840
C	1.25881845	4.57539803	-0.01192404
H	2.30568998	2.72998173	-0.26621237
C	0.00067515	5.17582083	0.07690570
N	-0.07836045	6.64239750	0.24921946
O	-0.56321104	7.26624102	-0.65984910
O	0.35053791	7.08605710	1.28369149
C	-2.55525317	5.09026462	0.08077296
F	-3.48453622	4.19975712	0.41843388
F	-2.90751954	5.61630108	-1.08970542
F	-2.57706253	6.06545460	0.99249215
C	2.55064792	5.37420666	0.06737077
F	3.55757603	4.69620332	-0.47816645
F	2.88158890	5.64356172	1.32774324
F	2.44160456	6.53352341	-0.58632134

*

D21

Au	-0.35890892	-3.23174687	-0.11871200
C	-1.01290992	-1.30261536	-0.38331466
C	-0.27296044	-0.21557870	-0.64366709
C	0.28615454	2.08979106	-0.41408166
C	-0.80673174	1.19832144	-0.80652922
H	-0.90159900	1.43231150	-1.89681247
C	1.23013630	-0.09030465	-0.79167158
H	1.51715337	-0.14176374	-1.85628580
H	1.78415438	-0.87273551	-0.25687001
C	1.52614391	1.31368722	-0.25604297
P	0.36271450	-5.44044707	0.18831244
C	-0.78734147	-6.47277885	1.15293912
C	0.61752071	-6.36284187	-1.36182938
H	-2.09450371	-1.09820245	-0.31458002
C	1.95101248	-5.58583861	1.06942007
H	1.86316222	-5.12541432	2.06450270
H	2.23678784	-6.64257712	1.18311904
H	2.73551800	-5.05678514	0.50870544
H	-0.93407144	-6.02799856	2.14811421
H	-0.39125600	-7.49334852	1.26618017
H	-1.76062661	-6.51601337	0.64250623
H	-0.32106720	-6.39008820	-1.93448931
H	0.94405425	-7.39267603	-1.15171730
H	1.38204722	-5.85640766	-1.96904587
H	-1.78920359	1.42819486	-0.36935703
H	1.64335751	1.29090058	0.85209025
H	2.42622038	1.81725637	-0.63712970
C	0.16907383	3.51353132	-0.20485051
C	-1.07263456	4.14859081	-0.39838785
C	1.29823910	4.25766623	0.18803881
C	-1.16875464	5.51457394	-0.21271827
H	-1.96910783	3.60472590	-0.69841260
C	1.17189312	5.61814936	0.39353482
H	2.27730288	3.80235184	0.34156226
C	-0.05381596	6.25347899	0.18693464
N	-0.16793253	7.70139446	0.38266653

O -0.66379143 8.33328907 -0.51779221
O 0.24446210 8.14448885 1.42628707
F -2.32345918 6.12581650 -0.38911177
F 2.21891232 6.33210985 0.75727289
*

D22
Au -0.38868969 -3.46754731 -0.07555757
C -1.05611880 -1.54003107 -0.30403072
C -0.34123772 -0.45542972 -0.64542310
C 0.27605815 1.85981362 -0.48642753
C -0.88223286 0.95797837 -0.79069371
H -1.17394320 1.14868663 -1.84293400
C 1.14583306 -0.35606465 -0.91553067
H 1.33210882 -0.40089052 -2.00343396
H 1.72090992 -1.16747507 -0.44821097
C 1.52608866 1.03225005 -0.38520872
P 0.35315821 -5.67557343 0.19376532
C -0.98698721 -6.90286038 0.33181653
C 1.39134492 -6.28721298 -1.17348416
H -2.12813358 -1.33322749 -0.14282369
C 1.36566393 -5.94828400 1.68458990
H 0.78126701 -5.68625218 2.57878071
H 1.68165568 -7.00031470 1.75358926
H 2.25689706 -5.30453351 1.65015211
H -1.62271358 -6.65755450 1.19531768
H -0.57565208 -7.91564150 0.46028181
H -1.60721142 -6.87644530 -0.57615500
H 0.82562376 -6.24145997 -2.11564263
H 1.70495380 -7.32606237 -0.98947756
H 2.28354401 -5.65145672 -1.27083425
H -1.77219297 1.16808198 -0.17904378
H 1.78101066 0.97609540 0.68954034
H 2.38742771 1.49519854 -0.88748399
C 0.20497000 3.22656088 -0.31621722
C -1.04540965 3.93675384 -0.41637064
C 1.37564031 4.01501744 -0.02437190
C -1.12575477 5.28337133 -0.24946516
H -1.95999488 3.37997429 -0.63093818
C 1.31341452 5.36122337 0.15270371
H 2.34610654 3.52110857 0.05641821
C 0.05511262 6.05710695 0.04399473
H -2.09258681 5.78005899 -0.32530941
H 2.22454720 5.91935062 0.36546394
N -0.01307065 7.36777482 0.20783635
C -1.24325088 8.16034652 0.05302098
C 1.13344236 8.21758299 0.56872519
H -1.77127637 7.87669375 -0.86770439
H -1.91209327 7.96863331 0.90866963
H 1.69101374 7.77405424 1.40490231
H 1.81261983 8.30085500 -0.29604364
C -0.74463800 9.60153782 0.03182618
H -0.46732673 9.88598133 -0.99555055
H -1.51044457 10.30609715 0.38295556
C 0.49550735 9.55719348 0.92226167
H 0.20369981 9.56471921 1.98433857
H 1.18520069 10.39527997 0.75447154
*

D23
Au -0.38825290 -3.11091300 -0.15443021
C -1.09110678 -1.19861592 -0.40484787
C -0.38072630 -0.09041016 -0.67114440
C 0.16160945 2.23418535 -0.42889428
C -0.94581311 1.31197422 -0.83471965
H -1.15866665 1.51530877 -1.90358500
C 1.12013342 0.05093375 -0.82288667
H 1.39280484 0.03052483 -1.89310067
H 1.67862276 -0.75294341 -0.32373089
C 1.41966780 1.43893823 -0.24323222
P 0.40351340 -5.29730609 0.14618125
C -0.85743271 -6.48319795 0.71610529
C 1.09385555 -6.05501561 -1.36041426
H -2.17757832 -1.02221975 -0.32495029
C 1.74112033 -5.43849921 1.37605698
H 1.38068574 -5.08415338 2.35310227
H 2.07632390 -6.48259637 1.46982889
H 2.59163758 -4.81110265 1.07093470
H -1.27546837 -6.14158517 1.67434868
H -0.41922689 -7.48426389 0.84722451
H -1.67290622 -6.53881033 -0.01987060
H 0.32279395 -6.08676676 -2.14416215
H 1.44564403 -7.07775418 -1.15649240
H 1.93605597 -5.44792500 -1.72388864
H -1.88634643 1.49088010 -0.29262714
H 1.58755629 1.37246466 0.84829642
H 2.30552805 1.93440783 -0.66613947
C 0.04483661 3.59797684 -0.24197743
C -1.20448469 4.28308630 -0.41530956
C 1.17393196 4.40590655 0.13033500
C -1.32295926 5.62637529 -0.22597029
H -2.09421126 3.71563848 -0.69488895
C 1.05157818 5.73887616 0.32958649
H 2.16892759 3.97038949 0.23980006
C -0.20702618 6.43961232 0.18477040
H -2.29927014 6.09160104 -0.34865351
N -0.35233199 7.74270239 0.39841815
C -1.58675079 8.42518640 0.01871150
C 0.63998630 8.58158729 1.07328862
H -1.37415033 9.49655846 -0.06905971
H -1.94962736 8.07053571 -0.95373633
H -2.37130583 8.28107275 0.77841096
H 0.10711526 9.41650768 1.54286895
H 1.15801891 8.02382907 1.85831934
H 1.37254808 8.98588434 0.35938230
F 2.15527345 6.44230308 0.61562557
*

D24
Au -0.35596787 -3.75872144 -0.13623172
C -1.02760453 -1.83358145 -0.38084807
C -0.30132410 -0.74325221 -0.66657975
C 0.25681667 1.56678794 -0.44839845
C -0.84633478 0.66774100 -0.81816532
H -0.98958779 0.89618605 -1.90183167
C 1.19622195 -0.61386623 -0.85746889
H 1.45155973 -0.66347685 -1.93026114
H 1.76606487 -1.39768782 -0.34138680
C 1.50659614 0.78902978 -0.32596098
P 0.39453335 -5.96045719 0.15086057
C -0.88684415 -7.12058492 0.72667977
C 1.05573674 -6.72391207 -1.36543043
H -2.10767974 -1.63443520 -0.27918174
C 1.73875654 -6.13125747 1.36908977

H 1.39222471 -5.77580056 2.35063718
H 2.05504967 -7.18191631 1.45478053
H 2.59822076 -5.51828065 1.06019924
H -1.29014945 -6.77396068 1.68945664
H -0.46830777 -8.13090491 0.85073853
H -1.70895737 -7.15627952 -0.00318035
H 0.27875824 -6.73136147 -2.14385969
H 1.38303385 -7.75634686 -1.16976758
H 1.91047590 -6.13575616 -1.73065587
H -1.81125108 0.88589948 -0.33795378
H 1.67298407 0.76109482 0.77358842
H 2.38799969 1.29312146 -0.74778461
C 0.14094976 2.97629567 -0.22872058
C -1.11052286 3.61784841 -0.35758979
C 1.27687919 3.73953241 0.12015367
C -1.21329796 4.98206404 -0.14646307
H -1.99977816 3.04679202 -0.62693737
C 1.15539345 5.10115799 0.33798525
H 2.25270480 3.26353170 0.22328811
C -0.08530756 5.72822507 0.20319434
H -0.17360657 6.80596304 0.36970540
C -2.54096999 5.69101132 -0.28313288
F -2.90592527 6.25764416 0.87093679
F -2.47458945 6.66514895 -1.19569142
F -3.51622074 4.85876718 -0.65018122
C 2.35418265 5.93729785 0.72190904
F 2.54874685 6.93012171 -0.15131242
F 2.18605157 6.49709119 1.92373583
F 3.47342512 5.21337201 0.76655294
*

D3
Au -0.37695784 -2.89546251 -0.00657641
C -0.77110168 -0.88841442 -0.18478923
C 0.08175840 0.09512682 -0.50680231
C 0.96812751 2.30315817 -0.29769287
C -0.27049858 1.56998617 -0.61270570
H -0.45323449 1.83473720 -1.68077158
C 1.56909306 0.01962473 -0.78677335
H 1.75107986 -0.04286852 -1.87384649
H 2.05272669 -0.84699808 -0.31690658
C 2.10310721 1.35422801 -0.25563690
P 0.04104859 -5.19270947 0.20046246
C -1.37884494 -6.24844332 -0.23347009
C 1.39843075 -5.79664605 -0.85431891
H -1.80423058 -0.54107828 -0.01465827
C 0.50400938 -5.71722603 1.88274917
H -0.30786810 -5.46937332 2.58217071
H 0.69241521 -6.80106913 1.91525482
H 1.41131262 -5.18169425 2.19874902
H -2.23190219 -6.00350851 0.41614064
H -1.12492318 -7.31248077 -0.11276239
H -1.67014579 -6.06087215 -1.27734241
H 1.16179777 -5.59615576 -1.90962166
H 1.54898066 -6.87795359 -0.71518949
H 2.32714347 -5.26557386 -0.59878324
H -1.16221061 1.90734864 -0.06494923
H 2.32758067 1.27837099 0.83033315
H 3.02023810 1.73890126 -0.72400171
C 1.06191053 3.70587786 -0.05954080
C -0.10298278 4.51104872 -0.08153366
C 2.31477976 4.31079158 0.19939336
C -0.00330372 5.87092054 0.14194714
H -1.07925970 4.06456572 -0.27367437
C 2.40023754 5.67504404 0.42812513
H 3.22310818 3.70625316 0.21835377
C 1.24572410 6.45517851 0.39769768
H 3.36722806 6.14012649 0.62637502
H 1.31521688 7.53204261 0.57362027
C -1.22852673 6.75263637 0.12606292
F -1.41814212 7.34031278 1.31329454
F -1.10959587 7.73115086 -0.77857152
F -2.33727148 6.06914206 -0.16483931
*

D4
Au -0.37120814 -3.15441281 -0.07971521
C -1.02519665 -1.22329032 -0.32267357
C -0.29752224 -0.14066624 -0.63369579
C 0.28252945 2.16871069 -0.44727116
C -0.83600142 1.27273024 -0.78400595
H -1.00916075 1.49209271 -1.86450076
C 1.19607323 -0.02430623 -0.85991431
H 1.42544528 -0.08539854 -1.93802792
H 1.77132631 -0.80904840 -0.35108901
C 1.52942199 1.38057560 -0.34735951
P 0.34450050 -5.36773100 0.20865785
C -1.01237079 -6.57701873 0.33623725
C 1.39274383 -5.99666222 -1.14236123
H -2.10098752 -1.01408184 -0.19740610
C 1.33469453 -5.64079435 1.71381952
H 0.74169998 -5.36777291 2.59901713
H 1.63851864 -6.69569420 1.79259319
H 2.23306860 -5.00671603 1.68666571
H -1.65450409 -6.32062579 1.19172909
H -0.61437493 -7.59394182 0.47342256
H -1.62195808 -6.54723355 -0.57878153
H 0.84071336 -5.94688933 -2.09236972
H 1.68936672 -7.03893017 -0.94990158
H 2.29485339 -5.37345683 -1.22888816
H -1.78500079 1.50055489 -0.27761085
H 1.72419512 1.35966429 0.74722585
H 2.40307594 1.87486311 -0.79585544
C 0.18187599 3.57975349 -0.23813372
C -1.06873478 4.22987123 -0.34555488
C 1.33142848 4.34143401 0.08061127
C -1.16666824 5.59754572 -0.14647534
H -1.96831147 3.66281359 -0.58922152
C 1.22943519 5.70521521 0.28825790
H 2.30689287 3.86107468 0.16969769
C -0.01860579 6.32604439 0.16917295
H -2.13302528 6.09627307 -0.23109477
H 2.11599009 6.29010570 0.54033081
C -0.10013215 7.82684197 0.35699275
F 0.62083051 8.22338136 1.40857579
F 0.37916906 8.46288951 -0.71716954
F -1.35234800 8.24297643 0.54479997
*

D5
Au -0.37499830 -2.20493493 -0.02932992
C -1.04144830 -0.27549816 -0.24728153
C -0.31696643 0.81871983 -0.52895304
C 0.26340295 3.13114641 -0.28979294

C	-0.86045373	2.23173562	-0.66741449
H	-1.06845763	2.45481757	-1.73562985
C	1.17999391	0.93849244	-0.72812254
H	1.42214414	0.90858706	-1.80515916
H	1.74383335	0.13289056	-0.23845871
C	1.51349377	2.32535991	-0.16608619
P	0.36772098	-4.41339959	0.23030543
C	-0.91842130	-5.57614655	0.79081184
C	1.02787284	-5.16242148	-1.29382598
H	-2.12080589	-0.07655194	-0.13355446
C	1.71015897	-4.60582726	1.44762035
H	1.36464392	-4.26054591	2.43312445
H	2.02203779	-5.65876118	1.52094353
H	2.57246203	-3.99278949	1.14682386
H	-1.32176551	-5.23916833	1.75710084
H	-0.50414152	-6.58953005	0.90367965
H	-1.73995332	-5.59991815	0.05972379
H	0.25290078	-5.15456973	-2.07420749
H	1.34851264	-6.19927796	-1.11093869
H	1.88691636	-4.57480519	-1.64945839
H	-1.79584908	2.43248896	-0.12504578
H	1.70976365	2.26437149	0.92273673
H	2.39051136	2.81734523	-0.61016274
C	0.16544406	4.50960594	-0.07287326
C	-1.08828082	5.18493899	-0.19064228
C	1.31505403	5.28573909	0.27673966
C	-1.19036407	6.53565748	0.02234471
H	-1.98567274	4.62267202	-0.45557733
C	1.22045858	6.63433587	0.49569171
H	2.28829644	4.80117293	0.37382279
C	-0.03490755	7.27408196	0.36888874
H	-2.15113123	7.04885750	-0.06874147
H	2.09125765	7.23483567	0.76431867
O	-0.07258661	8.57228483	0.58607949
H	-0.97399469	8.92099402	0.48113708

*

D6

Au	-0.35922452	-2.56154120	-0.09756407
C	-0.97085262	-0.61817301	-0.34815489
C	-0.21692604	0.46322175	-0.60056828
C	0.38923552	2.76685551	-0.33941291
C	-0.73222200	1.88411646	-0.76363788
H	-0.89333391	2.10725665	-1.83974606
C	1.28848728	0.55914061	-0.73868042
H	1.57268940	0.52429748	-1.80520576
H	1.81971081	-0.25497474	-0.22709739
C	1.62104996	1.94166511	-0.16494195
P	0.30274471	-4.79162472	0.19458715
C	-1.06274722	-5.99337678	0.08520895
C	1.51748910	-5.37961532	-1.03010981
H	-2.05025597	-0.39909463	-0.28069947
C	1.08173552	-5.13298838	1.80633580
H	0.37702222	-4.88816086	2.61455620
H	1.36873934	-6.19268130	1.88409334
H	1.97753408	-4.50554103	1.92243307
H	-1.81394846	-5.77163402	0.85735530
H	-0.69031624	-7.01947736	0.22584764
H	-1.54348789	-5.91482560	-0.90102643
H	1.09151770	-5.29285534	-2.04045878
H	1.78496906	-6.42985700	-0.83836951
H	2.42464540	-4.75961894	-0.98019684
H	-1.68614930	2.09892038	-0.26029000
H	1.77593474	1.87964876	0.93025483
H	2.52173397	2.41926300	-0.57639705
C	0.30405844	4.14524160	-0.12588897
C	-0.93331321	4.83741052	-0.29179603
C	1.44962183	4.90982834	0.26713017
C	-1.03548992	6.19112904	-0.08651524
H	-1.82621858	4.28422639	-0.58932058
C	1.35837381	6.25715523	0.47854649
H	2.41349675	4.41542775	0.40142471
C	0.11469413	6.92000370	0.30358810
H	-1.99489598	6.69014787	-0.22154041
H	2.22347799	6.85124922	0.77818512
O	0.12099673	8.21320066	0.52481745
C	-1.06913203	8.98594557	0.38380544
H	-1.44505615	8.93922570	-0.64882989
H	-1.84155388	8.64270721	1.08769090
H	-0.78823095	10.01696192	0.62432595

*

D7

Au	-0.37675731	-2.47669634	0.00667986
C	-1.01153852	-0.53391692	-0.18749110
C	-0.28458975	0.53865872	-0.53575493
C	0.34967333	2.84024457	-0.37512208
C	-0.80712557	1.96031963	-0.66202288
H	-1.05924657	2.17592738	-1.72433417
C	1.19758744	0.62685347	-0.83626679
H	1.36821445	0.56980855	-1.92562915
H	1.77987688	-0.17671354	-0.36572800
C	1.58888983	2.01959188	-0.32874005
P	0.31945589	-4.70431998	0.22682603
C	-1.04155563	-5.88850546	0.48182165
C	1.21432089	-5.35129484	-1.22284597
H	-2.07610026	-0.30866482	-0.00536217
C	1.44878420	-5.00202421	1.62567436
H	0.95252097	-4.71911529	2.56558911
H	1.73825884	-6.06276470	1.67313332
H	2.35194751	-4.38515931	1.50931659
H	-1.60148727	-5.61932719	1.38943995
H	-0.65139649	-6.91232590	0.58616040
H	-1.72841301	-5.84849073	-0.37630890
H	0.56772500	-5.29569710	-2.11093658
H	1.51470110	-6.39738306	-1.05874789
H	2.11105243	-4.74149946	-1.40645553
H	-1.71141832	2.19631492	-0.08243502
H	1.85236734	1.98063534	0.74816042
H	2.44329086	2.49350256	-0.83260777
C	0.28668279	4.23514206	-0.16831043
C	-0.95696418	4.92497443	-0.22013258
C	1.46279911	4.98705057	0.09760344
C	-1.01800628	6.28545726	-0.01992940
H	-1.87741650	4.37548534	-0.42507908
C	1.39139409	6.34794386	0.30402908
H	2.43309123	4.48928626	0.14269777
C	0.15452886	7.02515271	0.25072532
H	-1.98494134	6.78816964	-0.07294948
H	2.30117073	6.91600633	0.51063241
C	0.14156201	8.47771056	0.47801466
C	-0.95001016	9.25092545	0.52293964
H	1.12567249	8.93478364	0.62481983
H	-0.85719955	10.32577668	0.70017763

H -1.96320144 8.85960667 0.39264079
*

D8
Au -0.36033100 -2.42057600 -0.03390500
C -1.00548900 -0.49061500 -0.30329300
C -0.27104900 0.59784000 -0.57717700
C 0.29121300 2.91043400 -0.35278200
C -0.81022800 2.00878000 -0.74691500
H -0.95281800 2.22784700 -1.83012500
C 1.23059700 0.72071500 -0.73619600
H 1.50613800 0.66855700 -1.80400300
H 1.78507600 -0.06783600 -0.21013600
C 1.53794900 2.12231500 -0.19783900
P 0.33568200 -4.63674100 0.28122300
C -1.00173000 -5.76893500 0.78013400
C 1.06010500 -5.40958600 -1.20118900
H -2.08708400 -0.28751600 -0.22544100
C 1.60902300 -4.84314400 1.56807000
H 1.22232300 -4.47672800 2.53039700
H 1.89241900 -5.90168900 1.67013300
H 2.49905100 -4.25347300 1.30347100
H -1.44268600 -5.42137800 1.72591600
H -0.61689200 -6.79145600 0.91300300
H -1.78724300 -5.77439100 0.01026300
H 0.32502800 -5.39880300 -2.01928900
H 1.35586100 -6.44901700 -0.99277200
H 1.94407100 -4.83747600 -1.51927600
H -1.77603600 2.22466000 -0.26747500
H 1.69685700 2.09181200 0.90074800
H 2.42490900 2.61957000 -0.61597300
C 0.17620400 4.31043500 -0.14354500
C -1.07475900 4.96208800 -0.29565700
C 1.30938500 5.08186300 0.22218100
C -1.18729000 6.32283600 -0.09452900
H -1.96152400 4.39126500 -0.57591200
C 1.19617500 6.44140200 0.43078800
H 2.28366200 4.60553300 0.34322000
C -0.05299600 7.07383300 0.27227200
H -2.15043200 6.82156700 -0.21407800
H 2.06862400 7.03229600 0.71424000
C -0.17058700 8.48743000 0.48413500
C -0.27411100 9.68048900 0.66209200
H -0.36139200 10.74489700 0.81860000
*

D9
Au -0.39236622 -2.41267647 -0.04055366
C -1.05712073 -0.48299681 -0.26507277
C -0.33253590 0.59849994 -0.58698637
C 0.25789847 2.90540095 -0.41078649
C -0.86888853 2.01391920 -0.72488153
H -1.05689676 2.23541306 -1.80318421
C 1.15679211 0.71074591 -0.84264319
H 1.36486154 0.64737342 -1.92490524
H 1.73978967 -0.07474696 -0.34394362
C 1.50411084 2.11484390 -0.33782551
P 0.35746325 -4.61770681 0.22167406
C -0.90337544 -5.77418577 0.84764999
C 0.95553229 -5.38553548 -1.31847242
H -2.12977606 -0.27221840 -0.11816996
C 1.74753765 -4.79111659 1.38723737
H 1.43756412 -4.43951802 2.38229233
H 2.06699129 -5.84198472 1.45761628
H 2.59484440 -4.17704881 1.04856522
H -1.26986875 -5.42578995 1.82441710
H -0.48260360 -6.78524805 0.95716400
H -1.75270787 -5.80858049 0.14959451
H 0.14620381 -5.39433344 -2.06321307
H 1.28944295 -6.41788839 -1.13378331
H 1.79468251 -4.79986215 -1.72170844
H -1.80940115 2.24646619 -0.20501183
H 1.72004695 2.09385328 0.75313032
H 2.37020049 2.60738917 -0.80267591
C 0.16556110 4.31863214 -0.19650114
C -1.08387215 4.97428230 -0.28985581
C 1.32245599 5.07042776 0.11359200
C -1.17580850 6.33966475 -0.08386669
H -1.98765028 4.41245806 -0.52982579
C 1.23446422 6.43414281 0.33096484
H 2.29585891 4.58388449 0.18976732
C -0.01540346 7.06300897 0.22884793
H -2.13719135 6.84976011 -0.15879154
H 2.12338215 7.01713755 0.57588401
C -0.10849230 8.48563940 0.45014785
N -0.18291471 9.62529632 0.62740290
*

TSCD0
Au 0.32023196 -1.56318286 -0.03818892
C 0.92675292 0.39086224 -0.47858386
C 0.11114604 1.04106859 -1.21856564
C -0.21382993 3.36919947 -1.28047365
C 0.87806497 2.92892842 -1.96799713
H 1.88869680 3.25000149 -1.70418868
C -1.18226353 1.25365298 -1.91827995
H -1.92322660 0.53284464 -1.54178293
H -1.05161639 1.07044720 -2.99520332
C -1.54156686 2.71450785 -1.61291114
P -0.27117573 -3.74009405 0.51663450
C -1.91345266 -3.87221858 1.28540440
C -0.32006357 -4.86399959 -0.91137089
H 1.88356685 0.75167371 -0.09009231
C 0.87617297 -4.51006577 1.69765820
H 0.89822184 -3.92230698 2.62677936
H 0.55656145 -5.53819607 1.92501923
H 1.88846885 -4.53058599 1.26879700
H -1.94008719 -3.27090211 2.20566603
H -2.13594855 -4.92203680 1.52921346
H -2.67661928 -3.49068199 0.59176581
H -1.05087070 -4.49565209 -1.64583453
H -0.60695740 -5.87639229 -0.58918511
H 0.67031907 -4.90074645 -1.38767870
H 0.78390738 2.41619350 -2.92865160
H -2.04945695 3.21005062 -2.45392096
H -2.20858039 2.76468151 -0.74307964
C -0.12197461 4.32684387 -0.15613661
C -1.24497364 5.08793851 0.20596446
C 1.07436041 4.50815134 0.55817443
C -1.16734478 6.01987863 1.23880972
H -2.18692850 4.96911527 -0.33480740
C 1.14268781 5.42363240 1.60343638
H 1.95727561 3.91413654 0.31144850
C 0.02415222 6.18699709 1.94323752

H	-2.04647996	6.61546987	1.49698710
H	2.07700635	5.54146649	2.15785591
H	0.08224071	6.91006139	2.76088097

*

TSCD1

Au	0.31373683	-1.87433378	0.03690006
C	1.04329876	0.03601531	-0.41314513
C	0.38356976	0.63986543	-1.32595081
C	0.12116238	2.98048147	-1.60412007
C	1.30819799	2.47900080	-2.04979584
H	2.26440634	2.81546560	-1.64199704
C	-0.75696534	0.82382369	-2.25878969
H	-1.56939027	0.13662081	-1.97899412
H	-0.44045969	0.56912499	-3.28127271
C	-1.13756229	2.30377232	-2.11483074
P	-0.42267261	-4.00071047	0.61147620
C	-2.18152735	-4.05962542	1.06801988
C	-0.23359828	-5.22029397	-0.72349906
H	1.92731018	0.40866140	0.11227383
C	0.46495462	-4.70595783	2.03244313
H	0.32889991	-4.05931773	2.91133240
H	0.08177633	-5.71254177	2.25800613
H	1.53861850	-4.76853232	1.80348264
H	-2.36600945	-3.38608881	1.91730627
H	-2.46723676	-5.08508157	1.34708586
H	-2.79531530	-3.73061554	0.21698172
H	-0.81561744	-4.90181794	-1.60046231
H	-0.58951446	-6.20657940	-0.38932428
H	0.82541893	-5.29329132	-1.01021840
H	1.37116460	1.88945341	-2.96775508
H	-1.49139915	2.74214881	-3.06015239
H	-1.94240177	2.41335365	-1.37698671
C	0.02641420	4.02604043	-0.56425239
C	-1.15110209	4.77997116	-0.42608748
C	1.09614111	4.30997141	0.29966920
C	-1.24473331	5.78947579	0.52533055
H	-2.00358911	4.59299611	-1.08336485
C	0.98794862	5.30674339	1.26341455
H	2.02220616	3.73374795	0.23889135
C	-0.18054320	6.06879221	1.39351333
H	-2.16836617	6.37089992	0.59917249
H	1.83171607	5.49814758	1.93234532
C	-0.30471228	7.13945260	2.44245206
H	-0.67381807	8.08139187	2.00914296
H	-1.02329752	6.83992857	3.22224064
H	0.65925645	7.33743443	2.93136195

*

TSCD10

Au	0.27222547	-1.73896944	0.11431626
C	0.99300409	0.21749878	-0.06431798
C	0.69401365	0.78959456	-1.16795106
C	0.44485095	3.08740552	-1.53899943
C	1.74215951	2.67001306	-1.51712192
H	2.44998029	3.04059002	-0.77166123
C	-0.02169397	0.91141782	-2.46433963
H	-0.84925950	0.18694851	-2.49375825
H	0.66653904	0.66986101	-3.28796581
C	-0.48800090	2.37444387	-2.50112462
P	-0.48271209	-3.91502996	0.41538514
C	-2.29230649	-4.07589746	0.35227985
C	0.14736371	-5.08093217	-0.82911429
H	1.58795630	0.63982378	0.75061848
C	0.00587968	-4.61744472	2.01937308
H	-0.39997326	-3.99852544	2.83259566
H	-0.37705938	-5.64437605	2.11792567
H	1.10253105	-4.62887066	2.09866498
H	-2.74545012	-3.45576304	1.13919848
H	-2.58580286	-5.12586351	0.50229183
H	-2.66105582	-3.73030233	-0.62429814
H	-0.17414225	-4.76169017	-1.83101825
H	-0.23469601	-6.09350484	-0.62955667
H	1.24644302	-5.09431167	-0.79892320
H	2.18205683	2.11625959	-2.35052268
H	-0.44830010	2.80491673	-3.51281286
H	-1.52283080	2.45165032	-2.14490149
C	-0.09568753	4.07707602	-0.58057796
C	-1.22960793	4.82813939	-0.91855140
C	0.51418627	4.29126829	0.66741420
C	-1.72042365	5.78594611	-0.03246734
H	-1.72223764	4.68424220	-1.88305831
C	0.01801123	5.23016419	1.57234747
H	1.38484063	3.69587029	0.95508725
C	-1.10730294	5.98227305	1.20127045
H	-2.59525646	6.38045421	-0.30790504
H	-1.50822245	6.72705311	1.89533278
C	0.66910607	5.43954674	2.91399366
H	1.08458172	6.45628882	2.99594593
H	-0.06001713	5.32053646	3.73018614
H	1.48719682	4.72484998	3.08036362

*

TSCD11

Au	0.26192707	-1.76392109	0.13458991
C	0.98317627	0.19296024	-0.03683280
C	0.66328586	0.77597911	-1.12941549
C	0.41767161	3.07616367	-1.49401888
C	1.70957508	2.64120534	-1.49282887
H	2.43492520	3.00538815	-0.76120964
C	-0.08292756	0.90319766	-2.40852981
H	-0.91496339	0.18351298	-2.41950630
H	0.58416268	0.65989096	-3.24890691
C	-0.54302037	2.36788970	-2.43084577
P	-0.49345064	-3.94219890	0.41839058
C	-2.30304857	-4.10239521	0.35204326
C	0.13689719	-5.09682666	-0.83653188
H	1.59774406	0.60653316	0.76803087
C	-0.00642125	-4.65995711	2.01600625
H	-0.41566832	-4.05076669	2.83486328
H	-0.38691003	-5.68885298	2.10290162
H	1.09008014	-4.66948307	2.09752859
H	-2.75713471	-3.49074699	1.14502985
H	-2.59670714	-5.15389801	0.49058233
H	-2.67100324	-3.74672717	-0.62122620
H	-0.18418925	-4.76814865	-1.83553149
H	-0.24575180	-6.11105370	-0.64671010
H	1.23595642	-5.11096685	-0.80610720
H	2.12862837	2.08256941	-2.33368772
H	-0.53031861	2.80069073	-3.44231909
H	-1.56697398	2.44883593	-2.04525239
C	-0.09071577	4.08607360	-0.53739750
C	-1.23633827	4.83183773	-0.85820612
C	0.56626246	4.32356711	0.67701423

C	-1.69436474	5.80956681	0.02200348
H	-1.75967000	4.66790009	-1.80222802
C	0.08846230	5.29029804	1.56396394
H	1.44858574	3.75094333	0.96990687
C	-1.04571168	6.04161842	1.23172161
H	-2.57676381	6.40070472	-0.23553258
H	-1.41622966	6.80115865	1.92680203
O	0.75576657	5.45810978	2.72212163
H	0.33867015	6.15794941	3.24406432

*

TSCD12

Au	0.30887613	-2.48016066	0.02798099
C	1.00339475	-0.64017500	-0.69291207
C	0.18374598	-0.05698607	-1.47989355
C	-0.07334184	2.30577486	-1.89010460
C	0.98686759	1.69772321	-2.49641753
H	2.01557814	2.02286410	-2.32525802
C	-1.12050026	0.12947959	-2.16330834
H	-1.87808951	-0.50064898	-1.67385964
H	-1.03522204	-0.20256021	-3.20892772
C	-1.42148993	1.62885969	-2.04822816
P	-0.37936185	-4.53070757	0.87383630
C	-1.71158818	-4.39474441	2.10339632
C	-1.01674184	-5.66305269	-0.39780643
H	1.99664408	-0.29091290	-0.39713933
C	0.95361855	-5.44342712	1.70713184
H	1.34325795	-4.84615933	2.54415662
H	0.57527250	-6.40330169	2.08979256
H	1.77121724	-5.63160862	0.99631907
H	-1.37390672	-3.77964721	2.95000811
H	-1.99552106	-5.39330704	2.46861355
H	-2.58774907	-3.91348030	1.64526080
H	-1.88016032	-5.20328521	-0.90008973
H	-1.32683822	-6.61465079	0.05970926
H	-0.23505060	-5.85607188	-1.14675217
H	0.85136937	1.02052767	-3.34338593
H	-1.97860490	2.01505693	-2.91544991
H	-2.03297609	1.81460463	-1.15626807
C	0.06296025	3.48099464	-1.00671421
C	-1.06887637	4.22452613	-0.63126853
C	1.31408862	3.91431921	-0.53498282
C	-0.95477123	5.35778691	0.16573315
H	-2.06152751	3.93024434	-0.97851041
C	1.42222245	5.03538092	0.27691565
H	2.22136117	3.36261748	-0.78956006
C	0.29332817	5.78548350	0.64069879
H	-1.86178627	5.91301547	0.42029104
H	2.40766127	5.34265347	0.63758293
C	0.45640755	6.98738708	1.50544901
C	-0.42939838	8.19989292	1.34009035
C	-0.57097785	7.34401488	2.55484401
H	1.49721740	7.19272568	1.77145914
H	-1.21298035	8.14888853	0.57805131
H	0.04154709	9.18117128	1.44306641
H	-0.19925024	7.72515859	3.50981439
H	-1.45260830	6.70044678	2.63305027

*

TSCD13

Au	0.28718708	-1.91928267	0.08396703
C	0.99285778	0.02391734	-0.24098320
C	0.61049028	0.54200745	-1.34692238
C	0.33615565	2.80364555	-1.82431740
C	1.62859671	2.37342804	-1.87648206
H	2.39284921	2.77393290	-1.20580638
C	-0.21236204	0.58783798	-2.58465105
H	-1.04145334	-0.12999462	-2.49834792
H	0.40217754	0.29117462	-3.44773926
C	-0.67414247	2.05009173	-2.66909213
P	-0.45465396	-4.07612907	0.52637476
C	-2.09254568	-4.43442242	-0.17692771
C	0.64414993	-5.36544896	-0.13314167
H	1.65009245	0.48550018	0.50166459
C	-0.60359640	-4.43468708	2.30208737
H	-1.31396578	-3.73268397	2.76214948
H	-0.95969016	-5.46485278	2.45343233
H	0.37618798	-4.31382482	2.78648923
H	-2.83181223	-3.72889694	0.22905539
H	-2.39551625	-5.46307771	0.07026547
H	-2.05745042	-4.31854313	-1.26985983
H	0.73103360	-5.25510108	-1.22369300
H	0.24403301	-6.36346858	0.10121491
H	1.64392068	-5.26111372	0.31269847
H	1.99994427	1.77438753	-2.71196165
H	-0.71152928	2.42443476	-3.70293214
H	-1.67706692	2.15727664	-2.23694143
C	-0.12023855	3.85048508	-0.88267088
C	-1.27220028	4.59810819	-1.17128486
C	0.57898502	4.12835688	0.30109674
C	-1.70357374	5.61192010	-0.31481569
H	-1.83536478	4.40159547	-2.08676777
C	0.13527194	5.12550845	1.16806274
H	1.46780965	3.55141674	0.57245790
C	-1.00630002	5.87585581	0.85767713
H	-2.59407657	6.19162526	-0.56841062
H	-1.33304594	6.65598359	1.54967703
C	0.88313633	5.38640141	2.42635547
O	0.57514052	6.23170185	3.23157026
H	1.77412881	4.73375956	2.58744975

*

TSCD14

Au	0.32734270	-2.19828256	0.13095970
C	1.01740447	-0.33420688	-0.51715239
C	0.39840295	0.14256830	-1.53308054
C	0.14494995	2.34955401	-2.06501314
C	1.35361958	1.83846132	-2.43538602
H	2.28670718	2.20255061	-1.99832659
C	-0.71461150	0.16286276	-2.52126706
H	-1.53271814	-0.48318579	-2.17041919
H	-0.36145823	-0.23175766	-3.48568923
C	-1.09189739	1.65042224	-2.59841066
P	-0.39362321	-4.26028238	0.92410378
C	-1.80267376	-4.13965747	2.06651641
C	-0.94162081	-5.39068081	-0.39007582
H	1.85719915	0.13654328	0.00274528
C	0.88982974	-5.16658354	1.83817633
H	1.21946811	-4.56846332	2.70010550
H	0.49527519	-6.13014176	2.19434519
H	1.75352662	-5.34672951	1.18205576
H	-1.52213874	-3.52897396	2.93692983
H	-2.10378408	-5.14205266	2.40656007
H	-2.65064429	-3.65875540	1.55768883

H -1.77374829 -4.93283036 -0.94423160
H -1.27590241 -6.34547295 0.04309992
H -0.11336333 -5.57779779 -1.08873788
H 1.45846364 1.19976765 -3.31637832
H -1.34444967 1.97458279 -3.61867917
H -1.95364607 1.86176634 -1.95329020
C 0.01401019 3.43734729 -1.06775984
C -1.10977358 4.27695776 -1.10452375
C 0.99670070 3.65091943 -0.08770193
C -1.24532271 5.32187512 -0.19811720
H -1.88446426 4.12848661 -1.85952851
C 0.86354983 4.67887605 0.83588687
H 1.86981956 2.99852101 -0.02780139
C -0.25571759 5.50151956 0.76064928
H -2.10928925 5.98602653 -0.22682945
H 1.61503055 4.84570359 1.60783670
N -0.39900320 6.59787087 1.73344974
O 0.46950083 6.73022502 2.56684480
O -1.37669754 7.30674297 1.64565059
*

TSCD15
Au 0.27635719 -2.23480980 0.20118374
C 1.10471036 -0.33409582 -0.07742942
C 0.79869370 0.21124835 -1.19498174
C 0.66886784 2.48258098 -1.67057053
C 1.93178114 1.96832336 -1.67075186
H 2.69400817 2.31624101 -0.96960901
C 0.02608496 0.30498751 -2.46293122
H -0.84461753 -0.36544376 -2.41513678
H 0.65634136 -0.01891652 -3.30460229
C -0.35263078 1.79036440 -2.55209739
P -0.62431342 -4.34409346 0.57456296
C -2.38954116 -4.30345965 1.00824659
C -0.51142691 -5.44126358 -0.87085014
H 1.75551917 0.09051363 0.69276166
C 0.18351822 -5.24809681 1.92881451
H 0.09674158 -4.67274449 2.86181972
H -0.28873911 -6.23296943 2.06286131
H 1.24976667 -5.38477608 1.69735178
H -2.52836100 -3.72585619 1.93351032
H -2.76965483 -5.32554861 1.15584705
H -2.95595321 -3.81760205 0.20066750
H -1.03604943 -4.98511081 -1.72277227
H -0.96478218 -6.41803081 -0.64404791
H 0.54432265 -5.58494847 -1.14286026
H 2.29816097 1.35447230 -2.49743746
H -0.33793928 2.17442264 -3.58295757
H -1.36164673 1.94638413 -2.15039782
C 0.24638940 3.56482737 -0.75400903
C -0.89209359 4.32257524 -1.06256853
C 0.97372366 3.85759694 0.41002538
C -1.27427490 5.36092945 -0.22902255
H -1.48850745 4.13911840 -1.95759873
C 0.55882290 4.88631352 1.23602957
H 1.85570067 3.28836410 0.70756877
C -0.56337078 5.65339316 0.93006339
F -2.34068266 6.09986620 -0.51878205
F -0.94510046 6.63905310 1.72791255
F 1.22690962 5.16856059 2.35025997
*

TSCD16
Au 0.32478427 -2.15830572 -0.05386556
C 0.84610950 -0.21150595 -0.62743850
C -0.05716334 0.38262069 -1.30521500
C -0.50405504 2.76500490 -1.45310011
C 0.56695933 2.31286094 -2.17018896
H 1.57605742 2.68795383 -1.98025246
C -1.40242334 0.54809004 -1.90580030
H -2.08936321 -0.18671960 -1.45966924
H -1.34524447 0.33970802 -2.98469616
C -1.80026910 1.99992068 -1.61370533
P -0.14884134 -4.32629631 0.63037768
C -1.12932023 -4.40776920 2.15882138
C -1.09007869 -5.28533983 -0.59393872
H 1.82964959 0.17606607 -0.34796741
C 1.34413654 -5.30741862 0.96619704
H 1.92764229 -4.82808543 1.76547039
H 1.06754586 -6.32599292 1.27743540
H 1.96350810 -5.35906698 0.05912679
H -0.58468426 -3.91121906 2.97487346
H -1.31884924 -5.45677347 2.43230674
H -2.08923006 -3.89204925 2.01126068
H -2.05414219 -4.79253733 -0.78606113
H -1.27037292 -6.30482763 -0.22083946
H -0.52558028 -5.33645110 -1.53616049
H 0.44549573 1.72402353 -3.08319571
H -2.42209747 2.43616472 -2.41071353
H -2.38857946 2.04878634 -0.68969819
C -0.39006493 3.86147415 -0.47599818
C -1.21554689 3.93814407 0.65481646
C 0.58052119 4.86972443 -0.66687698
C -1.07298844 4.97279228 1.57716304
H -1.96962834 3.17368827 0.84772335
C 0.71772935 5.90308939 0.24243094
H 1.21884651 4.86354079 -1.55469394
C -0.11251436 5.96055584 1.38337834
H -1.70647410 5.02490412 2.46545672
O 0.02246045 6.96314823 2.26282345
H 0.73355828 7.55080525 1.96288181
O 1.60677730 6.91760892 0.13481047
H 2.14032140 6.82829975 -0.66723686
*

TSCD17
Au 0.25547720 -2.73296400 0.16261912
C 1.28072528 -0.90650082 0.09659806
C 1.09383227 -0.25601849 -0.98516625
C 1.16670634 2.15821763 -1.34229160
C 2.36632921 1.51920673 -1.22516583
H 3.10371508 1.80582488 -0.47193383
C 0.45968182 0.02883073 -2.29479790
H -0.44455965 -0.58967593 -2.39720119
H 1.15121296 -0.25389026 -3.10247958
C 0.15704418 1.53182343 -2.28485182
P -0.83032494 -4.77905697 0.32800949
C -2.61674370 -4.68090976 0.00673588
C -0.19901790 -6.02198287 -0.83880815
H 1.91717576 -0.62382746 0.93978833
C -0.67816391 -5.54142225 1.97109082
H -1.09584456 -4.86619731 2.73177229
H -1.21999263 -6.49882431 1.99634414
H 0.38297808 -5.71738379 2.19949060

H	-3.08400485	-3.99561079	0.72865613
H	-3.07569206	-5.67677843	0.09962614
H	-2.78814098	-4.29426151	-1.00829322
H	-0.32373416	-5.65793306	-1.86882870
H	-0.74474887	-6.97005082	-0.71916322
H	0.87116491	-6.19239024	-0.65227977
H	2.74147926	0.85824117	-2.01006418
H	0.19826528	1.97583303	-3.29133641
H	-0.85593497	1.69700783	-1.89526808
C	0.77981940	3.31635827	-0.51538237
C	-0.35336518	4.08469650	-0.86731495
C	1.51351694	3.69534836	0.61094687
C	-0.72688746	5.19951644	-0.13126929
H	-0.93732310	3.81438903	-1.74659192
C	1.13519277	4.80057253	1.37465107
H	2.38749239	3.12316579	0.92675937
C	0.02553921	5.56521843	1.01847817
H	1.72112881	5.06171166	2.25671882
O	-0.40984182	6.64469154	1.68018746
C	0.28849424	7.06894844	2.83139087
H	0.27632501	6.29780855	3.61965652
H	1.33275434	7.33470722	2.59696625
H	-0.23161514	7.96178866	3.19951256
O	-1.77644773	5.99151832	-0.41535365
C	-2.56779614	5.68936544	-1.54109119
H	-3.04821072	4.70014934	-1.44909306
H	-3.34861038	6.45841661	-1.59055359
H	-1.97958039	5.72026707	-2.47418673

*

TSCD18

Au	0.34841718	-2.56903696	-0.04670270
C	0.87518309	-0.68470649	-0.80679745
C	-0.09993899	-0.10597944	-1.38567377
C	-0.54022221	2.33824745	-1.68053385
C	0.44023570	1.79373292	-2.46159381
H	1.46034337	2.18647064	-2.45029240
C	-1.50002753	0.08604272	-1.82314772
H	-2.14540908	-0.59717579	-1.25045177
H	-1.58511472	-0.18835590	-2.88538549
C	-1.83473152	1.56036576	-1.57520803
P	-0.13107063	-4.66810091	0.82037112
C	-1.83760586	-4.83185253	1.42471036
C	0.08198490	-6.00858395	-0.38882576
H	1.90509534	-0.33574990	-0.69625395
C	0.92097777	-5.12082630	2.23166027
H	0.78749551	-4.38756754	3.04016851
H	0.65105318	-6.12222469	2.59928824
H	1.97634821	-5.11832489	1.92300858
H	-2.02179758	-4.08909266	2.21435220
H	-2.00181364	-5.84213076	1.82898204
H	-2.54168245	-4.65081528	0.59972680
H	-0.59044564	-5.84419936	-1.24310275
H	-0.14835957	-6.97950106	0.07519304
H	1.11952451	-6.01627174	-0.75284769
H	0.21542001	1.09701667	-3.27327834
H	-2.58800240	1.94106354	-2.28263036
H	-2.25995891	1.67454942	-0.57037988
C	-0.34073225	3.56100648	-0.89602031
C	-1.09197013	3.85859130	0.25763347
C	0.62148419	4.51627936	-1.28431506
C	-0.89191886	5.01738517	0.98848298
H	-1.84797708	3.15777973	0.61800066
C	0.83338758	5.68262115	-0.57345016
H	1.20773175	4.35169890	-2.19197726
C	0.08100281	5.97332755	0.59621015
H	-1.49919637	5.18251136	1.87839321
H	1.58244814	6.38633722	-0.93629559
N	0.28032917	7.11990232	1.30390889
C	-0.50922286	7.39346664	2.48719376
C	1.28070215	8.07610556	0.87564352
H	-0.19851930	8.35541744	2.91048224
H	-1.58702781	7.45619245	2.25897479
H	-0.36832707	6.62229785	3.26380002
H	1.28214984	8.92944124	1.56357733
H	2.29440118	7.64017736	0.87597384
H	1.07512114	8.46143021	-0.13757869

*

TSCD19

Au	0.14038895	-2.77802358	0.23910470
C	1.05239144	-0.91917826	-0.03721639
C	0.98816590	-0.45660428	-1.23397443
C	1.14292944	1.68853996	-1.87034957
C	2.34862855	1.09704503	-1.63162985
H	2.99585262	1.42690769	-0.81468205
C	0.43267035	-0.45663553	-2.61781938
H	-0.50152246	-1.03572162	-2.64538637
H	1.14759227	-0.92692884	-3.30941475
C	0.23863676	1.04135606	-2.90144324
P	-0.84218505	-4.85404039	0.60227842
C	-2.47111786	-4.75457087	1.40281144
C	-1.11753511	-5.79044586	-0.93163480
H	1.57369778	-0.44386441	0.79952113
C	0.15287828	-5.94560819	1.66182352
H	0.29242041	-5.47670587	2.64665375
H	-0.35092060	-6.91570296	1.78831297
H	1.13974894	-6.10463859	1.20358802
H	-2.37135885	-4.27085688	2.38532354
H	-2.89396759	-5.76202524	1.53390666
H	-3.15006794	-4.15298280	0.78137751
H	-1.77505145	-5.21587951	-1.59993283
H	-1.58414665	-6.76157093	-0.70785668
H	-0.15648360	-5.95624759	-1.43964301
H	2.80636619	0.42053103	-2.35802629
H	0.50903461	1.32595209	-3.92872662
H	-0.80451328	1.33543377	-2.72883657
C	0.65815844	2.83345122	-1.06337360
C	-0.18591601	3.78054037	-1.64962888
C	1.03451031	2.99389013	0.27312090
C	-0.63713975	4.88653174	-0.92302493
H	-0.49376756	3.68374310	-2.69227078
C	0.59130317	4.09126840	1.01503985
H	1.66753455	2.25508235	0.76692839
C	-0.24221985	5.03733496	0.40917027
N	-0.70601437	6.19893082	1.18112181
O	0.07091150	6.68144990	1.97004200
O	-1.82966714	6.58853290	0.97064830
C	-1.47656904	5.84062450	-1.60389756
N	-2.12912433	6.56143076	-2.22753685
C	0.99340294	4.17516139	2.39719553
N	1.34414453	4.15541970	3.49761889

*

TSCD2
Au 0.34099157 -2.11320531 0.01670652
C 1.04825439 -0.23467913 -0.57709906
C 0.34390247 0.31794777 -1.49080392
C 0.05794681 2.61087702 -1.87321868
C 1.22341145 2.09568117 -2.35879435
H 2.19677186 2.44520989 -2.00604633
C -0.84733142 0.43376403 -2.37193044
H -1.63879928 -0.23787866 -2.00768575
H -0.58236197 0.11884485 -3.39221588
C -1.22436272 1.92004338 -2.29841652
P -0.39211717 -4.19028543 0.75496143
C -2.14778968 -4.20559835 1.22725332
C -0.21743667 -5.50832528 -0.48475351
H 1.95209463 0.17222942 -0.11446793
C 0.50552931 -4.78301320 2.22021879
H 0.38432254 -4.06202243 3.04163808
H 0.11718605 -5.76298751 2.53606599
H 1.57600127 -4.87420425 1.98591826
H -2.32070855 -3.47329767 2.02914158
H -2.43989956 -5.20673620 1.57854673
H -2.76574958 -3.93059655 0.36020103
H -0.79976350 -5.25027128 -1.38117500
H -0.58147230 -6.46382466 -0.07776909
H 0.83986666 -5.61201891 -0.76824004
H 1.24401795 1.47805303 -3.26040920
H -1.60790476 2.30879260 -3.25369358
H -2.00110455 2.07567216 -1.53914076
C 0.01545559 3.68946388 -0.86232157
C -1.14238854 4.46998374 -0.72304738
C 1.11535367 3.97000536 -0.03485719
C -1.19960736 5.51390230 0.19488202
H -2.01510735 4.28019516 -1.35152539
C 1.06373494 4.99585484 0.90075291
H 2.02475536 3.36887203 -0.09895806
C -0.09472711 5.76617779 1.00461953
H -2.10032908 6.12417975 0.28318843
H 1.91837451 5.19823254 1.54903180
Cl -0.16185187 7.05619066 2.16764157
*

TSCD20
Au 0.16981969 -3.47592290 0.13222136
C 1.21047953 -1.66403292 0.07659671
C 1.39528842 -1.18794007 -1.10072564
C 1.67258454 0.98268612 -1.65009196
C 2.81378230 0.38800757 -1.19821812
H 3.29387620 0.70168793 -0.26797249
C 1.13267468 -1.15175331 -2.56679649
H 0.22729986 -1.73155277 -2.79768158
H 1.97530794 -1.60620165 -3.10877403
C 0.99172335 0.35177901 -2.84982294
P -0.98968220 -5.48582698 0.27421581
C -1.26841496 -6.27346182 -1.34065485
C -0.13427339 -6.73806329 1.27653498
H 1.56749357 -1.21767788 1.00980891
C -2.63984161 -5.31826548 1.01802255
H -3.25304718 -4.64148845 0.40559166
H -3.13185847 -6.30077998 1.07756110
H -2.54915203 -4.89480572 2.02876341
H -1.86596553 -5.60620992 -1.97854982
H -1.80229728 -7.22714805 -1.21216291
H -0.30269813 -6.46036103 -1.83212823
H 0.85530987 -6.94440351 0.84371485
H -0.72142808 -7.66837262 1.30401018
H 0.00017538 -6.36188423 2.30104101
H 3.40360564 -0.27468936 -1.83686438
H 1.45591501 0.65934491 -3.79817025
H -0.06673479 0.63803085 -2.88161378
C 1.02486580 2.10164551 -0.92580103
C 0.20046336 2.99155530 -1.61978103
C 1.22598970 2.29809509 0.44427215
C -0.39466181 4.07142461 -0.97499137
H 0.02099627 2.85690792 -2.68730440
C 0.61908837 3.35383481 1.11427779
H 1.85499151 1.61319743 1.01392250
C -0.18290863 4.24001994 0.39282308
N -0.82303442 5.37131765 1.09143361
O -1.62113180 5.10003337 1.95476800
O -0.49903262 6.48060716 0.74506920
C -1.25636692 5.02127625 -1.78771287
F -1.67160565 4.44327164 -2.91504033
F -0.59049387 6.12382080 -2.12869479
F -2.34491081 5.39514152 -1.10753527
C 0.85008201 3.49756669 2.60842613
F 1.94975320 2.84475431 2.98641407
F -0.16591403 3.00324815 3.31533579
F 1.00663912 4.77772051 2.95916644
*

TSCD21
Au 0.25325019 -2.56496534 0.03109683
C 1.03194382 -0.72887600 -0.58991310
C 0.51442819 -0.24133199 -1.65792007
C 0.40717856 1.93925916 -2.19844161
C 1.61744855 1.38473175 -2.49282091
H 2.53021684 1.69219361 -1.97665316
C -0.52091713 -0.19206307 -2.72717096
H -1.39253515 -0.79240258 -2.42822263
H -0.11856619 -0.61643020 -3.65906759
C -0.81499195 1.31233817 -2.84439898
P -0.56375528 -4.59490331 0.81591327
C -2.19579519 -4.45449658 1.60435586
C -0.76431292 -5.85094001 -0.48298241
H 1.83909435 -0.27035869 -0.01006722
C 0.51216715 -5.35937062 2.06568736
H 0.62237356 -4.67911110 2.92261689
H 0.08165677 -6.31158397 2.41050432
H 1.50609337 -5.54436307 1.63313714
H -2.13636273 -3.75974974 2.45464987
H -2.53307930 -5.43900101 1.96220028
H -2.92356924 -4.06303374 0.87891840
H -1.47304028 -5.48685257 -1.24089986
H -1.14415591 -6.78925592 -0.05138528
H 0.20478917 -6.03835159 -0.96754508
H 1.75725637 0.75762193 -3.37722084
H -0.94965127 1.64453306 -3.88392169
H -1.72084959 1.57235659 -2.28348612
C 0.25357995 3.00499067 -1.17831113
C -0.83733389 3.87832432 -1.26085639
C 1.18569472 3.14920042 -0.14204100
C -0.97513842 4.89339641 -0.32891814
H -1.58875405 3.79785646 -2.04778264
C 1.01341314 4.14873299 0.79798642

H	2.04523651	2.48668586	-0.03251975
C	-0.06231053	5.03704919	0.71697200
N	-0.23418573	6.08858440	1.71413182
O	-0.07023324	5.78418583	2.87295897
O	-0.52984851	7.19024104	1.31306831
F	-2.01097108	5.71077537	-0.41098053
F	1.89801903	4.28156950	1.77189956

*

TSCD22

Au	0.37821668	-3.12681751	0.08757737
C	1.01150596	-1.29583536	-0.72131408
C	0.11827278	-0.75849141	-1.45191817
C	-0.30771702	1.68253037	-1.95249160
C	0.77555123	1.09681348	-2.54661536
H	1.78048590	1.51338289	-2.44186524
C	-1.21205455	-0.59986367	-2.07707732
H	-1.92308521	-1.26647001	-1.56575099
H	-1.15650391	-0.92039330	-3.12844524
C	-1.58995283	0.87713118	-1.93824486
P	-0.23151105	-5.16834856	1.00830979
C	-0.92662271	-5.03342617	2.68245702
C	-1.49104578	-6.04889444	0.03698808
H	2.02112082	-0.93834697	-0.50344942
C	1.15853189	-6.32977337	1.15944550
H	1.92561069	-5.90309362	1.82181787
H	0.80890393	-7.28646276	1.57592002
H	1.60435633	-6.50419109	0.16955808
H	-0.18471041	-4.58031580	3.35572708
H	-1.20094605	-6.02985632	3.06050577
H	-1.82140735	-4.39451925	2.66196075
H	-2.40267103	-5.43719416	-0.02560829
H	-1.73136876	-7.01203577	0.51206654
H	-1.11509100	-6.22839407	-0.98062283
H	0.66770858	0.33528083	-3.32292178
H	-2.28589172	1.20648508	-2.72558404
H	-2.10357570	1.02413516	-0.97973754
C	-0.24052239	2.97513977	-1.26501558
C	-1.21505671	3.39301498	-0.33594509
C	0.80730586	3.88429151	-1.52859344
C	-1.14698950	4.61814572	0.30392527
H	-2.05228680	2.73910083	-0.08369658
C	0.89482606	5.11270989	-0.90302950
H	1.56782638	3.63239056	-2.27171362
C	-0.08305301	5.51982841	0.04378100
H	-1.92119547	4.87983354	1.02628580
H	1.71687522	5.78121773	-1.16236575
N	-0.00684261	6.72071580	0.66563892
C	1.11120602	7.64678331	0.51358741
C	-1.03474886	7.23338527	1.56620352
H	2.07557632	7.12423122	0.61584117
H	1.09542590	8.12292453	-0.48464981
C	0.88156148	8.66857914	1.62566007
H	-2.03810272	7.12604714	1.12399488
H	-1.03199506	6.67808008	2.52278642
C	-0.64028462	8.69603432	1.76383380
H	1.33966999	8.31023269	2.56174603
H	1.31844999	9.64906467	1.39013207
H	-1.08207951	9.31301253	0.96473951
H	-0.98710353	9.09542627	2.72717008

*

TSCD23

Au	0.37136762	-2.67968351	0.12030607
C	1.12884315	-0.86367701	-0.60740141
C	0.36123058	-0.32304926	-1.46892969
C	0.07239606	2.06439897	-1.96142342
C	1.20813721	1.48987976	-2.45783286
H	2.19892802	1.85308326	-2.17258746
C	-0.87227082	-0.16437048	-2.27308497
H	-1.65655624	-0.81390117	-1.85561134
H	-0.67699730	-0.49908255	-3.30290308
C	-1.22671180	1.32566296	-2.20504819
P	-0.39069173	-4.69185279	0.99319418
C	-2.18095193	-4.93429753	0.78957349
C	0.38223983	-6.14974326	0.23155427
H	2.09148203	-0.50022574	-0.23821039
C	-0.08367883	-4.85924316	2.77690811
H	-0.58874903	-4.04437984	3.31548713
H	-0.46344728	-5.82604196	3.14025962
H	0.99643718	-4.79511415	2.97298214
H	-2.72198147	-4.12105251	1.29468089
H	-2.48686493	-5.89868082	1.22254488
H	-2.43740242	-4.91816887	-0.27957466
H	0.17689009	-6.15398328	-0.84869131
H	-0.01759742	-7.07101081	0.68146918
H	1.47062050	-6.11219546	0.38364383
H	1.18259677	0.79189635	-3.29885507
H	-1.72038355	1.68207291	-3.12245303
H	-1.92587007	1.50552087	-1.37932648
C	0.10264689	3.27420391	-1.13105451
C	1.13636283	4.21753159	-1.26457187
C	-0.88658211	3.54842070	-0.16693272
C	1.17947711	5.36979411	-0.49566360
H	1.91020409	4.06867681	-2.02138755
C	-0.82357027	4.68320298	0.60924864
H	-1.70557932	2.85707231	0.03978180
C	0.19250286	5.66326481	0.47588980
H	1.98345931	6.08519256	-0.67059384
F	-1.75751209	4.83261868	1.56926863
N	0.23842100	6.79434025	1.24057786
C	1.44990383	7.58585832	1.28783682
C	-0.95528841	7.42306416	1.78527579
H	1.44099094	8.19597670	2.20169618
H	2.33964062	6.94184068	1.33131251
H	1.55164744	8.26668198	0.42200781
H	-0.85103126	8.51439004	1.69620392
H	-1.84946981	7.13573286	1.21991402
H	-1.11579712	7.17960881	2.84880666

*

TSCD24

Au	0.12884194	-3.06559465	0.15630185
C	1.05256320	-1.20889866	0.41801332
C	1.51042780	-0.67915462	-0.65519182
C	1.85126203	1.54871572	-1.04486356
C	2.84679126	0.96684148	-0.31861236
H	3.05575215	1.26690150	0.71140273
C	1.64720348	-0.57456729	-2.13371681
H	0.85700651	-1.16552003	-2.61952093
H	2.61889139	-0.98316008	-2.44890024
C	1.53265886	0.93550832	-2.39575988
P	-0.90489120	-5.13858680	-0.03182948
C	-2.37004648	-5.12866786	-1.10787224

C	0.18130160	-6.42842642	-0.71095877
H	1.13973339	-0.79459635	1.42696174
C	-1.48647243	-5.78813194	1.56326675
H	-2.21251655	-5.08892394	2.00273041
H	-1.96454306	-6.76945815	1.42412580
H	-0.63580387	-5.89257957	2.25216610
H	-3.10852784	-4.41241004	-0.71956858
H	-2.82094529	-6.13176608	-1.14635896
H	-2.08064953	-4.82112408	-2.12314298
H	0.51127157	-6.13877351	-1.71909415
H	-0.35517182	-7.38778547	-0.76473878
H	1.06665651	-6.54160862	-0.06886428
H	3.59894470	0.32758001	-0.78809642
H	2.21819852	1.28563638	-3.18131420
H	0.51048431	1.19605360	-2.69773491
C	1.00359703	2.63650673	-0.50469622
C	0.42204874	3.55779051	-1.38389575
C	0.77906436	2.77568164	0.87115225
C	-0.34329663	4.61116075	-0.89160965
H	0.58070215	3.46737819	-2.45985695
C	-0.00758001	3.81778763	1.34922055
H	1.20101350	2.05821273	1.57669845
C	-0.56803056	4.74827492	0.47554783
H	-1.17809027	5.57069629	0.85838475
C	-0.27339002	3.96852186	2.82653178
F	0.16508588	5.14816495	3.28342372
F	0.32066443	3.01549108	3.54997179
F	-1.58438173	3.91319304	3.09494396
C	-0.95916671	5.61903193	-1.83039619
F	-0.62043279	6.86979611	-1.49396393
F	-0.58014695	5.43021946	-3.09709723
F	-2.29696711	5.55972557	-1.80231217

*

TSCD3

Au	0.26987288	-2.34027077	0.16733538
C	0.96830646	-0.39582661	-0.16463911
C	0.81602834	0.01596591	-1.36694270
C	0.56274657	2.21830318	-2.09982600
C	1.84649999	1.84749270	-1.82803091
H	2.43427898	2.34412320	-1.05232616
C	0.27786029	-0.08220042	-2.74979779
H	-0.52032779	-0.83848001	-2.78061519
H	1.07626982	-0.40657919	-3.43378010
C	-0.21558694	1.33825576	-3.05876459
P	-0.49186529	-4.48771939	0.62125749
C	-2.13414313	-4.83244272	-0.07869768
C	0.59220037	-5.79214991	-0.03300260
H	1.43440729	0.15407206	0.65813921
C	-0.64278774	-4.83577353	2.39888485
H	-1.34634150	-4.12481912	2.85567556
H	-1.00848858	-5.86174823	2.55568400
H	0.33840334	-4.72158286	2.88203845
H	-2.86576069	-4.11777331	0.32516691
H	-2.44733104	-5.85688865	0.17319569
H	-2.09933060	-4.72153469	-1.17214835
H	0.67944534	-5.68764638	-1.12410956
H	0.18109181	-6.78458848	0.20602845
H	1.59342292	-5.69690932	0.41159260
H	2.41213062	1.20064747	-2.50321641
H	-0.05303484	1.62530028	-4.10850271
H	-1.29025731	1.41911182	-2.85274130
C	-0.11574488	3.33062679	-1.39707661
C	-1.23195291	3.94881849	-1.97748355
C	0.34667701	3.79897152	-0.15693744
C	-1.86015682	5.02190986	-1.34742942
H	-1.61035788	3.60433570	-2.94248474
C	-0.29666884	4.85553460	0.47502950
H	1.20245155	3.32740231	0.32821224
C	-1.40000882	5.47799344	-0.11768802
H	-2.71930792	5.50196615	-1.82075777
H	-1.89645353	6.31309139	0.38267965
C	0.17283893	5.34350654	1.82120484
F	-0.73768332	5.10451432	2.77637551
F	0.38553513	6.66648623	1.81767076
F	1.31156203	4.76030990	2.20922054

*

TSCD4

Au	0.32995655	-2.58592266	0.07006666
C	1.00996369	-0.74788122	-0.66172720
C	0.29741530	-0.26906341	-1.61196820
C	0.02617598	1.95917939	-2.17575473
C	1.18023326	1.40081043	-2.64052159
H	2.16001382	1.76479768	-2.32120133
C	-0.90977873	-0.23156417	-2.48054885
H	-1.69921737	-0.85864670	-2.04074798
H	-0.66492980	-0.64159999	-3.47166139
C	-1.27093520	1.25970032	-2.53622301
P	-0.37479499	-4.62397202	0.93548584
C	-2.11884356	-4.62367272	1.44891258
C	-0.22259733	-5.99822849	-0.24504610
H	1.91552124	-0.30120931	-0.24094818
C	0.55884432	-5.14433723	2.40567092
H	0.44972901	-4.38832743	3.19675262
H	0.18346327	-6.11142446	2.77285300
H	1.62468877	-5.23966647	2.15278472
H	-2.27505997	-3.86185222	2.22642723
H	-2.40048966	-5.61104863	1.84491187
H	-2.75758312	-4.38262915	0.58690774
H	-0.82161690	-5.78130704	-1.14128632
H	-0.57779475	-6.93439130	0.21158030
H	0.82933819	-6.11450072	-0.54331038
H	1.18457842	0.72184820	-3.49738279
H	-1.65024453	1.57150688	-3.52078902
H	-2.04123352	1.49283034	-1.79028615
C	0.01546335	3.10726880	-1.24033926
C	-1.10945846	3.94571477	-1.18612881
C	1.11030449	3.39020358	-0.41116805
C	-1.12946489	5.05126380	-0.34484623
H	-1.97560945	3.74693802	-1.82073332
C	1.08576494	4.48497387	0.44628626
H	1.99001173	2.74345696	-0.41462272
C	-0.03110787	5.31872044	0.47443510
H	-2.00702376	5.70201299	-0.32441816
H	1.94045487	4.68740000	1.09399968
C	-0.07105894	6.52756414	1.37274527
F	-0.09480825	7.66510846	0.66289267
F	0.98637148	6.59338687	2.18697462
F	-1.16747969	6.53403086	2.14372103

*

TSCD5

Au	0.37232331	-1.89955834	0.01410497

C	0.95824091	0.02975434	-0.55687381
C	0.10012201	0.63074356	-1.28476806
C	-0.29358893	3.01812904	-1.46618232
C	0.80237319	2.54894005	-2.13218026
H	1.80682743	2.90719568	-1.89160170
C	-1.20983319	0.81636698	-1.95331119
H	-1.93080603	0.09601199	-1.53819666
H	-1.10240154	0.60042406	-3.02680921
C	-1.59490014	2.27726069	-1.68973998
P	-0.18428930	-4.04421624	0.70928246
C	-1.67484036	-4.70753892	-0.09254371
C	1.12178935	-5.26576557	0.38341974
H	1.93210040	0.40452613	-0.23009835
C	-0.51015446	-4.15924928	2.49409001
H	-1.34554047	-3.49594994	2.76101762
H	-0.76526629	-5.19433174	2.76718643
H	0.38234381	-3.84504066	3.05443608
H	-2.53388729	-4.05857186	0.13107916
H	-1.88183822	-5.72453670	0.27338773
H	-1.52743972	-4.73583870	-1.18183059
H	1.32889887	-5.30730536	-0.69566943
H	0.80647042	-6.26062450	0.73244110
H	2.04183229	-4.97073829	0.90851317
H	0.71605625	1.95913921	-3.04863559
H	-2.16432478	2.72068142	-2.52121738
H	-2.23085157	2.34319662	-0.79879480
C	-0.20323609	4.11332252	-0.48569610
C	-1.05740960	4.19499746	0.63048486
C	0.76228191	5.12487496	-0.64436823
C	-0.94307086	5.22369557	1.55328081
H	-1.81564505	3.42883906	0.80494664
C	0.87947786	6.16516410	0.26607394
H	1.42045893	5.11258090	-1.51655833
C	0.02760504	6.21939249	1.37802902
H	-1.59895003	5.27149752	2.42540584
H	1.62794291	6.94882314	0.11579846
O	0.09600496	7.19747419	2.29281671
H	0.80159176	7.81918308	2.06220424

*

TSCD6

Au	0.31049312	-2.29141128	0.20581510
C	1.14234930	-0.39574741	-0.12431750
C	0.69531848	0.17348628	-1.17502185
C	0.46834474	2.56408841	-1.63664850
C	1.72439713	2.03863304	-1.72587291
H	2.55968343	2.42862377	-1.13964844
C	-0.19586544	0.32842117	-2.34975012
H	-1.04005215	-0.37090998	-2.25426471
H	0.35706920	0.06010717	-3.26252012
C	-0.63432652	1.79747474	-2.34170981
P	-0.55615080	-4.40113811	0.64174869
C	-2.14659747	-4.36454814	1.52149218
C	-0.86210766	-5.37887564	-0.86010600
H	1.89662620	-0.01531110	0.56993222
C	0.53545039	-5.42897586	1.66973920
H	0.69856595	-4.93762712	2.63986384
H	0.08113121	-6.41771132	1.83404432
H	1.50647610	-5.55295176	1.16890316
H	-2.02240752	-3.85664176	2.48888780
H	-2.51002264	-5.38908608	1.69258895
H	-2.88632242	-3.81080696	0.92552444
H	-1.58157612	-4.85222136	-1.50351878
H	-1.26622781	-6.36706246	-0.59339264
H	0.07887059	-5.50746132	-1.41423501
H	2.00778717	1.37144444	-2.54339523
H	-0.82569751	2.18453547	-3.35437518
H	-1.56815806	1.89397228	-1.77321201
C	0.13117524	3.73079707	-0.80190010
C	-1.12739223	4.35552428	-0.91247684
C	1.04734859	4.27861631	0.11081256
C	-1.44594277	5.47318552	-0.16026452
H	-1.87225507	3.97607620	-1.61498432
C	0.73578617	5.38821578	0.88734804
H	2.03266789	3.82571014	0.23795294
C	-0.51957127	6.00099573	0.75377558
H	-2.41864463	5.95991251	-0.25958051
H	1.47632772	5.76854381	1.59178270
O	-0.91347238	7.07676980	1.44795650
C	-0.02977935	7.66194491	2.38283232
H	0.23781529	6.95393308	3.18427146
H	0.88862192	8.03022835	1.89670917
H	-0.56289814	8.51257757	2.82462083

*

TSCD7

Au	0.32860089	-2.04972287	0.01532579
C	1.05096710	-0.16408545	-0.53683171
C	0.41008029	0.37638309	-1.50186125
C	0.12671262	2.67899552	-1.89972296
C	1.33236507	2.18277735	-2.29894723
H	2.27263911	2.54563501	-1.87707562
C	-0.70729361	0.48782266	-2.47418050
H	-1.51767289	-0.19463913	-2.17745238
H	-0.35882242	0.18032968	-3.47143088
C	-1.10866609	1.96899119	-2.42319434
P	-0.42161744	-4.13349524	0.71564687
C	-2.23394959	-4.26108578	0.78492725
C	0.11267665	-5.49984641	-0.35787228
H	1.91204891	0.25518427	-0.00833292
C	0.15312174	-4.57887748	2.38143244
H	-0.20228086	-3.83243975	3.10646432
H	-0.23007827	-5.57114309	2.66340438
H	1.25239274	-4.59424375	2.40170173
H	-2.63075647	-3.50813220	1.48111353
H	-2.53205704	-5.26410592	1.12575788
H	-2.65622589	-4.07675244	-0.21351526
H	-0.26903919	-5.34015896	-1.37670233
H	-0.26937683	-6.45706466	0.02803097
H	1.21108800	-5.53449073	-0.39403460
H	1.42661444	1.56291559	-3.19403110
H	-1.42666882	2.35505520	-3.40322374
H	-1.94259037	2.10786071	-1.72387594
C	-0.00839958	3.75000103	-0.89072994
C	-1.18796011	4.50515795	-0.81904559
C	1.02806765	4.05220226	0.01130556
C	-1.31669221	5.53684495	0.10543513
H	-2.01384737	4.30279700	-1.50481003
C	0.88760158	5.06698486	0.94596045
H	1.95271326	3.47083498	0.00128120
C	-0.28755779	5.83640037	1.00863856
H	-2.24043122	6.12151229	0.13132018
H	1.70848342	5.25981423	1.64053951
C	-0.48347842	6.93239187	1.98086435

C 0.39340717 7.35438910 2.89751056
H -1.45736113 7.43144309 1.91700508
H 0.13991320 8.17980393 3.56847037
H 1.38501716 6.90784454 3.02050804
*

TSCD8
Au 0.34358965 -1.99958880 0.02154315
C 1.05207655 -0.11882866 -0.56310078
C 0.34794950 0.44174246 -1.47260077
C 0.05489488 2.73494635 -1.83941190
C 1.22158063 2.22257131 -2.32626334
H 2.19427385 2.57065284 -1.97035631
C -0.84551191 0.55946681 -2.35101222
H -1.63477788 -0.11593850 -1.98905889
H -0.58146161 0.25060584 -3.37341502
C -1.22578738 2.04434930 -2.26913533
P -0.39487641 -4.07693920 0.75380159
C -2.15692151 -4.09396325 1.20196084
C -0.20094000 -5.39759852 -0.48025010
H 1.95655276 0.28326130 -0.09762514
C 0.48303105 -4.66495351 2.23287497
H 0.34983978 -3.94189622 3.05061636
H 0.09132699 -5.64449830 2.54589026
H 1.55672662 -4.75567113 2.01363688
H -2.34134409 -3.36085622 2.00054150
H -2.45256502 -5.09498442 1.55061683
H -2.76348003 -3.82074163 0.32632960
H -0.77186040 -5.14297320 -1.38494189
H -0.56820436 -6.35291150 -0.07573663
H 0.86027173 -5.49965425 -0.74936367
H 1.24356930 1.61200600 -3.23268648
H -1.60920150 2.43791736 -3.22250801
H -2.00333938 2.19447260 -1.50962967
C 0.01078002 3.80936789 -0.82392242
C -1.15016350 4.58520030 -0.67973227
C 1.11149693 4.08957874 0.00317138
C -1.20685665 5.62104019 0.24503489
H -2.02163430 4.39449212 -1.30960755
C 1.05326760 5.10885995 0.94304433
H 2.02314455 3.49294986 -0.06767886
C -0.10648761 5.89101201 1.07173491
H -2.11297781 6.22406441 0.33359845
H 1.91192361 5.30569833 1.58844479
C -0.16549145 6.95062304 2.04469271
C -0.21103825 7.83860117 2.86730994
H -0.25543944 8.63085859 3.59802340
*

TSCD9
Au 0.32942807 -1.96027314 0.03727251
C 1.01111329 -0.06792581 -0.53660546
C 0.31675569 0.48379916 -1.46136551
C 0.03905461 2.73761019 -1.84252391
C 1.20674786 2.22769113 -2.32934169
H 2.17691628 2.56702480 -1.95818518
C -0.87454540 0.57861161 -2.34813312
H -1.66661980 -0.08696917 -1.97487670
H -0.60733356 0.25018729 -3.36358764
C -1.24542631 2.06771583 -2.29306471
P -0.37890910 -4.05522270 0.75077151
C -2.13171161 -4.09554563 1.23207253
C -0.19768209 -5.35397184 -0.50827973
H 1.90177582 0.35203320 -0.06000127
C 0.53277172 -4.65980188 2.20249158
H 0.40721498 -3.95321983 3.03568576
H 0.15765430 -5.64916188 2.50471050
H 1.60306982 -4.73452847 1.96168190
H -2.30838220 -3.37554969 2.04417655
H -2.41091117 -5.10436197 1.57170551
H -2.75714891 -3.81632938 0.37174553
H -0.78749030 -5.08958525 -1.39794761
H -0.54974307 -6.31870454 -0.11271530
H 0.85904693 -5.44275923 -0.79890089
H 1.23246919 1.62850817 -3.24332964
H -1.60024023 2.45656050 -3.25910790
H -2.03634605 2.23685687 -1.55155420
C 0.00156550 3.79565005 -0.80711068
C -1.12473014 4.62700362 -0.71130830
C 1.07376450 3.99678510 0.07709123
C -1.17524029 5.65033567 0.22694923
H -1.96978687 4.49059194 -1.38907450
C 1.02487668 5.00314575 1.03158336
H 1.95304903 3.35071413 0.03779875
C -0.09988662 5.83707482 1.10391995
H -2.04963503 6.30137346 0.28436957
H 1.85641659 5.14608107 1.72428407
C -0.15115685 6.88888970 2.08732468
N -0.19098654 7.73249346 2.87802155
*
